# Protein Intake and Resistance Training Across Clinical Populations: A PRISMA-Guided Rapid Review of Observational and Randomized Evidence

**DOI:** 10.3390/nu18193259

**Published:** 2026-10-02

**Authors:** Csilla Dézsi, Viktor Gulyás-Oldal, Ekaterine Paschali, Imre Gálóczi, Sándor Nagy, Károly Szili

**Affiliations:** 1 Department of Obstetrics and Gynecology, Faculty of Health and Sport Science, Széchenyi István University, Egyetem tér 1, 9026 Győr, Hungary; sziline.dezsi.csilla@szte.hu (C.D.); goviktor93@gmail.com (V.G.-O.); drpaschali@hotmail.com (E.P.); immer444@gmail.com (I.G.); 2 Doctoral School of Regional Sciences and Business Administration, Széchenyi István University, Egyetem tér 1, 9026 Győr, Hungary; 3 Department of Obstetrics and Gynecology, Albert Szent-Györgyi Medical School, University of Szeged, Semmelweis utca 1, 6725 Szeged, Hungary

**Keywords:** dietary protein, protein intake, sarcopenia, muscle mass, all-cause mortality, resistance training, kidney disease, body composition, GLP-1 receptor agonists, physical activity

## Abstract

**Background/Objectives:** Guidance on protein and physical activity rests on population-wide minima with little differentiation by age or disease. We examined how protein intake and resistance training relate to muscle, function and mortality across clinical populations. **Methods:** PRISMA-guided rapid review: Fifty-eight logged PubMed queries returned 7544 unique records for screening (the analytic corpus); Europe PMC, Semantic Scholar and ClinicalTrials.gov were tested for coverage only. Estimates were extracted by a deterministic script and adjudicated by the authors; random-effects models were fitted within strata sharing an effect measure and contrast, and randomized evidence was tabulated, not pooled. Screening was single-reviewer outside a duplicate-screened sample (889 records); risk of bias was rated in duplicate. **Results:** In randomized trials, added protein increased strength with resistance training (+2.01%) but not without it (+0.13%); no trial of protein with resistance training reported mortality. Meeting activity guidelines was associated with lower mortality (HR 0.76, 95% CI 0.72–0.79; k = 16; *I*^2^ = 64%; low certainty). Plant protein was associated with lower mortality (HR 0.76, 0.63–0.93; k = 4; very low certainty). After excluding studies at high risk of bias, the protein strata fell to single studies. The direction of protein–outcome associations differed between populations. **Conclusions:** Protein with resistance training is a plausible strategy for preserving muscle and function; effects on survival are untested, and population-specific protein ranges are provisional and hypothesis-generating.

## 1. Introduction

Two numbers dominate lifestyle guidance for healthy aging. The first is 150 min per week of moderate-intensity physical activity, the lower bound of the World Health Organization recommendation [1]. The second is about 0.8 g/kg body weight per day of dietary protein, the population reference intake derived from nitrogen-balance studies [2,3]. Both are widely communicated as minima and applied to adult populations with little differentiation. Expert groups have nonetheless proposed higher intakes for older adults, of 1.0–1.2 g/kg/day [4,5,6], and lower intakes in non-dialysis chronic kidney disease [7].

One growing population illustrates why undifferentiated minima may be inadequate. Glucagon-like peptide-1 receptor agonists produce large weight losses, a mean of 14.9% of body weight with semaglutide 2.4 mg over 68 weeks in the STEP 1 trial [8]. Across randomized trials, they also reduce lean body mass by about 1 kg more than control [9]. Because they act partly by suppressing appetite, they may also lower protein intake in people with obesity, many of whom are older or have metabolic disease. Services managing these patients typically record body weight, which does not distinguish loss of fat from loss of lean tissue. Where intake is recorded, it is often expressed as a percentage of energy, which does not identify a deficit in g/kg. How protein and activity guidance should apply to this population is therefore an open question (Section 3.11).

Each number carries an implicit model. The activity threshold implies that the benefit of movement accrues meaningfully only once a substantial weekly volume is reached. The protein floor implies that intake above the requirement is nutritionally uninteresting, and that the same requirement applies across the adult lifespan and across metabolic and renal disease states. Both implications have been questioned [10,11].

Physical activity is the shorter part of this review. Across sixteen prospective cohorts, meeting activity guidelines was associated with a pooled all-cause mortality HR of 0.76 (0.72–0.79). Lower mortality was observed in people with established chronic disease as well as in the general population (Section 3.2). Activity is included in a review about protein because the two exposures interact. Protein supplementation augments the gains in lean mass and strength produced by resistance training [12], whereas protein without training has shown little effect on strength (Section 3.9). The two exposures meet at sarcopenia—low muscle strength together with low muscle quantity or quality [13,14]—which is the axis of this review. The activity evidence is therefore presented once, as a pooled table and forest plot (Section 3.2), with its derivation in Document S4; the remaining sections concern protein.

Three developments motivate a reassessment. First, device-measured physical activity has made it possible to characterize the low end of the dose–response curve, where questionnaire-based cohorts were less informative [15]. Second, consensus definitions of sarcopenia have established functional endpoints [13], and expert groups have argued that 0.8 g/kg/day may be insufficient to preserve muscle function in older adults [4,5]. Third, protein is now presented in consumer nutrition messaging as a longevity intervention. Whether the outcome evidence supports that framing is one of the questions this review sets out to answer (Section 4.5 and Section 5.7).

A further, less discussed problem cuts across the protein literature: the exposure metric. Most mortality cohorts report protein as a percentage of total energy or as a quantile of intake. Neither can distinguish undernutrition from overnutrition. A person consuming 1400 kcal at 18% protein and a person consuming 2800 kcal at 18% protein occupy the same exposure category. They differ by roughly 63 g of protein per day. Quantile contrasts inherit the same defect, since the “highest quintile” denotes a different absolute intake in every cohort. For guideline work, where the deliverable is a number a clinician can prescribe, this metric is of limited use.

A recent perspective argues that both guidelines are anchored to deficiency prevention rather than to functional capacity and should be raised [16]. That argument is narrative, expresses protein per kilogram of body weight without addressing body size as a confounder, and proposes a single higher target for a broad population. This review examines the same question with a logged search, pooling where contrasts allow, risk-of-bias appraisal and population stratification (Document S4).

The central question of this review is whether protein recommendations should be population-specific and conditional on resistance training or clinical context. It is approached through five questions:

1. Where does the association between physical activity and mortality begin, and does it differ between generally healthy populations and those with established chronic disease?
2. Is the protein–mortality relationship better described by amount, by source, or by neither?
3. When protein is expressed in absolute units, and the population is specified, does a single recommendation remain tenable?
4. Does randomized evidence agree with the observational evidence on these questions, and where it does not, are the divergences systematic?
5. How far does the chain from protein, through muscle and sarcopenia, to mortality actually hold—and at which link does it stop being demonstrated?

We address them in a PRISMA-guided rapid review in which the search strategy was logged at execution time, and every effect estimate was extracted by deterministic script before human adjudication. We then consider the implications for policy, including whether the evidence on processed meat supports recommendations on protein source rather than on protein quantity.

## 2. Materials and Methods

### 2.1. Design, Article Type and Reporting Standard

This manuscript is a review, designed and reported as a PRISMA-guided rapid review; it is not a systematic review and should not be read as a guideline. It follows the reporting structure of PRISMA 2020: the search was logged at execution, identification and screening counts are reported in full, and the flow is auditable. It does not follow the conduct standards of a systematic review. There was no prospective registration. Screening was single-reviewer, outside a duplicate-screened sample of 889 records. The analytic corpus comes from a single database, with three further sources used only to test its coverage, and risk of bias was appraised after selection. Every deviation is declared in Section 2.8, and its implications for interpretation are carried into Section 4.7, Section 5 and Section 6.

### 2.2. Information Sources and Search Strategy

PubMed was searched via the NCBI E-utilities API. Fifty-eight queries were executed on 22 and 23 August 2026. They are reproduced verbatim in Table S1, with execution timestamps and true match counts. The further 153 executions against Europe PMC, Semantic Scholar and ClinicalTrials.gov are in Table S1b (Document S4).

Queries fell into ten tranches, grouped by tranche in Table S1 and described one by one, with the reasoning for each, in Document S3.

Search update at internal revision: Capped queries were re-executed without a retrieval cap. The nine core queries, bounded at 2023, were re-executed with a 1990 floor, which recovered two pooled analyses [10,17]. Europe PMC, Semantic Scholar, ClinicalTrials.gov and citation chasing were added to test coverage (Document S4, Table S15).

**Figure 1:**
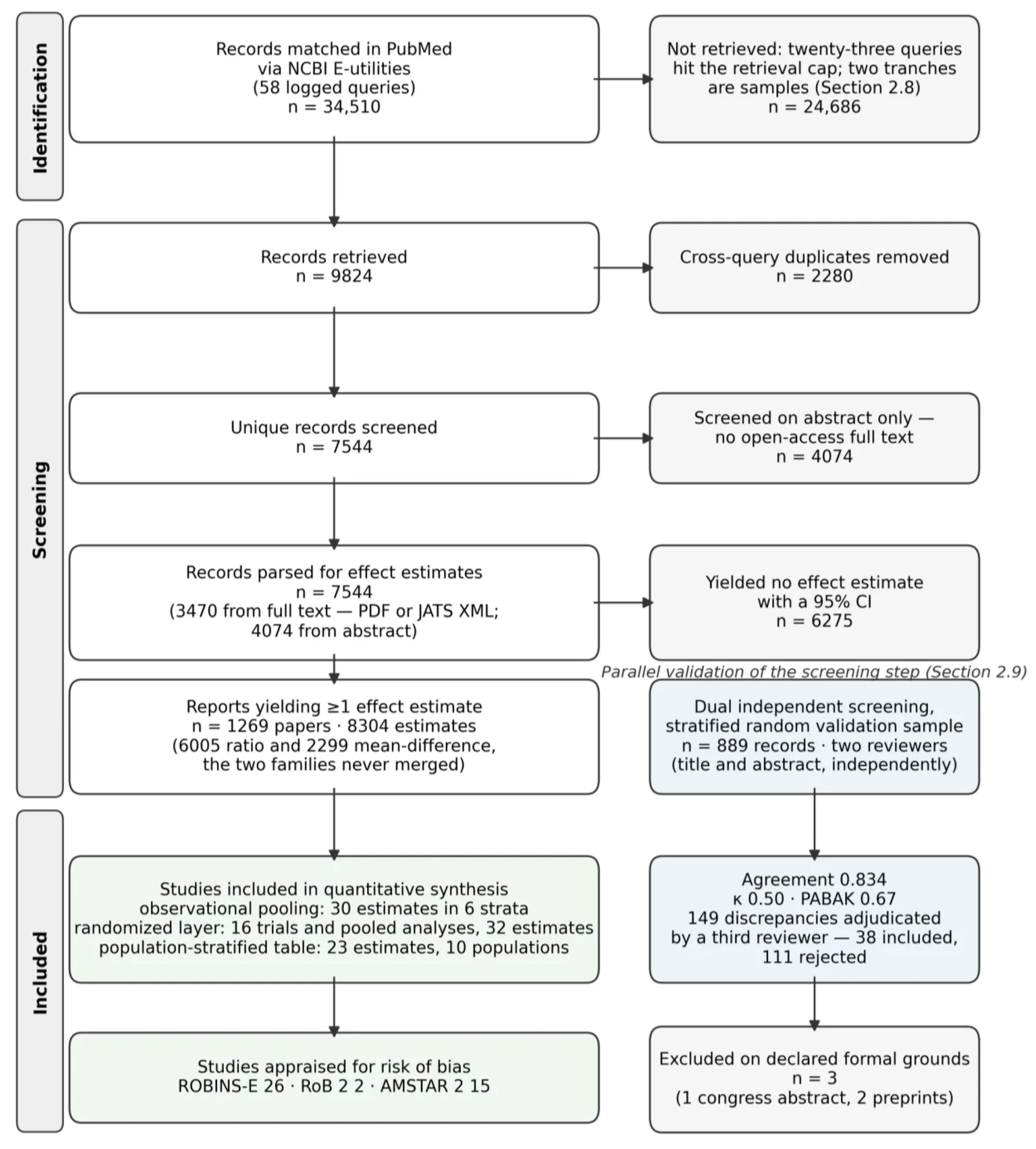
PRISMA 2020 flow of information across the ten search tranches. The right-hand panel below the screening phase shows the parallel duplicate screening of a stratified random sample (Section 2.9), not a step in the inclusion flow.

The syntheses in Section 3 rest on the originally screened PubMed corpus, together with the two pooled analyses recovered by the widened core queries. The counts in Figure 1 are those of that corpus. The enlarged multi-source corpus was under duplicate screening at the time of writing, and no count or inclusion from it is claimed here.

Every query was recorded at execution time in a machine-readable log capturing the database, timestamp, verbatim query string, PubMed’s true match count and the number of records retrieved. This log constitutes the PRISMA-S search-strategy appendix: Table S1 is its rendered form, and the log itself, with the query scripts as executed, is supplied in File S1.

### 2.3. Eligibility

We included human studies reporting a quantitative association between an exposure and an outcome of interest. The exposures were physical activity or resistance training, and dietary protein intake. The outcomes were all-cause or cause-specific mortality, incident chronic disease, sarcopenia, frailty, and physical function. Prospective cohorts, meta-analyses, umbrella reviews, randomized trials and Cochrane reviews were all eligible. Cross-sectional studies were admitted only to the descriptive population-stratified table and were never pooled; one such estimate is used, and its design is stated where it appears. Non-human studies and studies without a reported effect estimate with a 95% confidence interval were excluded at the extraction stage.

**Conference abstracts and meeting proceedings were excluded**, whether or not they reported an effect estimate. They carry no full methods and are not peer-reviewed to journal standard, and a large share are never published in full. An estimate taken from one therefore cannot be appraised with the instruments used here. The rule matters because two of the sources added at revision index them heavily where PubMed does not; applying the exclusion removed 3275 records. It is applied by an explicit, published rule rather than by hand. The rule keys on an indexed publication type or venue marker, or on a session code or an all-caps title in a record with no PubMed identifier. The excluded set is therefore reproducible by running the exclusion script supplied in File S1 over the retrieved corpus.

### 2.4. Data Extraction

Full texts were retrieved through the PMC Open Access Web Service and Europe PMC render endpoints; no web-interface scraping was used, in compliance with NCBI policy. Text was extracted from PDF and JATS XML. Effect estimates were then located by a deterministic regular-expression pipeline, which captured the measure (HR, RR, OR, IRR), the point estimate, both confidence limits, and a ±180-character provenance window.

The pipeline contains no language-model component. This is deliberate: separating mechanical retrieval from human judgment is what makes the harvest reproducible and the screening auditable. Every estimate admitted to an analysis was then read in its full-text context by at least one author before inclusion. No estimate entered a synthesis on the strength of an automated match.

Use of generative artificial intelligence: In accordance with MDPI policy, we disclose that a large language model (Claude Opus 5.0, Anthropic) was used as a technical assistant. Its roles were to write and debug the retrieval, extraction and meta-analysis code; to arrange candidate estimates in tables for author review; and to draft and edit manuscript text. At revision, it also checked cited numbers against their sources by script and drafted study-level moderator codings, which the authors verified before use. It made no screening, inclusion, risk-of-bias, certainty or interpretive decisions. Screening and adjudication of extracted estimates were performed by K.S., with independent duplicate screening of the validation sample by V.G.-O. and C.D. (Section 2.9). Risk of bias was rated by V.G.-O. and C.D., with disagreements resolved by K.S. Certainty grading and interpretation are the authors’ judgments. Every numerical result was produced by the deterministic R (4.6.2) and Python (3.12) code supplied as Supplementary Material. The authors reviewed and edited all output and take full responsibility for the content of this publication.

Validation of extraction: Automated extraction was used only to locate candidate estimates. Every estimate admitted to a pooled stratum (30), the population-stratified table (23) or the randomized table (32) was read by an author in its source text before admission. Two defects in the pipeline were found by comparing its output with sentences read by hand and were corrected before any analysis was finalized (Section 4.6; Document S1). The first was a silent failure of PDF text extraction, which left about a third of the corpus represented by abstracts only. Correcting it increased the ratio estimates extracted at that stage from 638 to 2566. The second was a regular expression that dropped minus signs from mean differences; its correction increased the mean-difference estimates from 841 to 1634, with signs correct. Because admitted estimates were read in their sources, both defects mainly affected which candidates were found rather than the values admitted. The sign error was itself detected during such reading. At revision, every numerical estimate cited in the final text was re-checked by script against the cited source, 93 estimate–citation pairs in all. Of these, 61 were matched in the abstract and a further 22 in the open-access full text (83 of 93, 89%). Each of the remaining ten was then checked by hand. Six are table cells in which the script paired an estimate with a neighboring citation in the same row rather than with the source it came from. All six were verified against the correct source. One is this review’s own pooled estimate, quoted in a row beside a cited estimate. Three are the same estimate from a report without an accessible full text at the time of the re-check [18], since verified by an author against the published article. The re-check found three confidence intervals transcribed incorrectly in Table 1, and one estimate reported with the wrong contrast (red meat and type 2 diabetes, Section 3.7). All were corrected, and none had entered a pooled estimate.

### 2.5. Synthesis

Estimates were pooled separately by measure. Hazard ratios, risk ratios and odds ratios were never converted into one another. The mortality literature is overwhelmingly prospective-cohort, and reports hazard ratios, and HR-to-OR conversion is invalid outside the rare-outcome limit. Genuine odds ratios were computed by us only where a published 2 × 2 table permitted it.

Primary cohorts and published meta-analyses were held in separate layers so that primary studies were not counted twice.

The randomized layer is reported as an evidence table and is not pooled, for a reason that is worth stating rather than leaving implicit. Most of the randomized evidence on exercise and mortality reaches us as successive Cochrane updates of overlapping trial sets. Pooling those pooled estimates would count the same trials four or five times and manufacture a precision that does not exist. Randomized and observational evidence are also never combined into a single estimate—they are compared, and where they disagree, the disagreement is the finding (Section 4.3).

Randomized trials report continuous outcomes, so the extraction pipeline was extended with a second measure family: standardized, weighted and unstandardized mean differences. These were written to a separate file from the ratio measures and never mixed with them.

### 2.6. Statistical Analysis

Analyses added at revision are marked as such and listed, with those planned at first draft, in Table S15.

Eligibility for pooling: An estimate entered a pooled stratum only when three conditions held. First, it shared the effect measure of the stratum: hazard ratios, risk ratios and odds ratios were pooled separately and never converted into one another (Section 2.5). Second, its exposure contrast matched the contrast definition of the stratum. For physical activity, this was a guideline-meeting or most-active category against an inactive or below-guideline reference; for protein intake, the highest against the lowest quantile of intake. Third, it was the covariate-adjusted estimate for the whole cohort. An unadjusted estimate was not used where an adjusted one was reported; in the COPD cohort, the adjusted HR of 0.53 was taken, not the unadjusted 0.35 [19]. Estimates reported only for a subgroup were not entered.

Contrast compatibility: The corpus reports per-standard-deviation, per-1000-step and other per-unit increments, highest-versus-lowest quantiles, meets-guideline-versus-not, and optimum-versus-zero contrasts. These are not interchangeable and were not merged. This restriction is the main reason several clinically important questions stop at k = 2 and are reported as evidence tables rather than pooled estimates. Estimates were also not admitted to the activity stratum when the exposure was an activity trajectory, a joint exposure category, an isotemporal-substitution estimate, occupational activity, sedentary time or bout length. Substitution estimates were not admitted to the protein strata. Contrasts judged compatible still use different cut-points, for example ≥600 MET-min/week, ≥150 min/week of moderate-to-vigorous activity, or the highest quintile of total activity. The pooled activity estimate therefore summarizes “guideline-meeting or most active versus least active” rather than the effect of a single dose. Whether this difference in exposure definition explains heterogeneity was tested directly (see below).

Multiple estimates and overlapping cohorts: One estimate per cohort per stratum was admitted. Where a paper reported several models or exposure categories, the adjusted whole-cohort estimate for the contrast matching the stratum was taken. Cohorts were analyzed as independent. At revision, we found that four estimates in the chronic-disease subgroup were drawn from overlapping cycles of the US National Health and Nutrition Examination Survey (NHANES) [20,21,22,23]. Because their participants may overlap, three sensitivity analyses were added: removing all four, retaining one at a time, and combining the four into a single fixed-effect estimate before random-effects pooling.

Effect size and model: Ratio estimates were analyzed on the natural-log scale. Standard errors were derived from the reported 95% confidence limits as (ln upper limit − ln lower limit)/(2 × 1.96). Random-effects models were fitted by restricted maximum likelihood (REML) using metafor 5.0.1 in R 4.6, with Wald-type confidence intervals. At revision, the activity stratum was refitted with the Knapp–Hartung adjustment as a sensitivity analysis [24].

Heterogeneity: Heterogeneity is reported as Cochran’s Q, τ^2^ and *I*^2^, with a 95% prediction interval for each pooled stratum. Confidence intervals for τ^2^ and *I*^2^ (95%, Q-profile method) were added at revision. Generally healthy and chronic-disease cohorts were compared with a mixed-effects moderator test (Q_M); this subgroup contrast is recorded in the protocol (Document S2), which is retrospective. At revision, heterogeneity in the activity stratum (k = 16) was explored further by univariable mixed-effects meta-regression. The moderators were exposure definition (extreme category versus guideline threshold), region (East Asia versus elsewhere), publication year, the standard error of each estimate, and length of follow-up. Each is reported with Q_M and the proportion of τ^2^ explained (R^2^); the follow-up model was also tested by permutation with 5000 iterations. The method used to measure activity could not be examined as a moderator, because it does not vary. Fifteen of the sixteen cohorts measured activity by self-report. The sixteenth does not name an instrument, but draws its exposure from the same questionnaire-based survey as several of the others. These analyses are exploratory, involve several tests at k = 16, and support no conclusion on their own (Table S14).

Influence, small-study effects and risk of bias: Influence was assessed by leave-one-out re-estimation. At revision, standardized influence diagnostics (Cook’s distance, DFFITS) and Baujat coordinates were added, reported per cohort in Table S14. Small-study effects were assessed with Egger’s regression test only where k ≥ 10, which in this review is the activity stratum alone. Trim-and-fill was added at revision as a sensitivity analysis. A non-significant Egger test is not evidence that publication bias is absent. With 16 studies, the test has low power, and funnel-plot asymmetry can also arise from heterogeneity rather than selective publication [25]. Each pooled stratum was also re-estimated after excluding studies rated at high overall risk of bias (Section 3.5).

Minimum number of studies and when not to pool: A stratum was pooled only when at least three estimates shared both the effect measure and the contrast definition. Otherwise, the estimates are presented in evidence tables. The minimum governs pooled results. Where a pooled stratum is split by a moderator, a side of the split may fall below it. Such a side is reported as a subgroup description, with its k stated and without being described as a pooled estimate. No conclusion rests on it. The randomized layer (Section 2.5) and the population-stratified estimates in absolute units (Section 2.7) were not pooled. The three-study minimum is a pragmatic convention of this review, not an accepted methodological standard. With few studies, τ^2^ is estimated imprecisely, and conventional random-effects confidence intervals tend to be too narrow [24,26]. *I*^2^ is biased [27], and a random-effects meta-analysis has little power [28]. Pooled estimates from strata with k ≤ 4 are therefore presented as exploratory and directional. They are reported with their k and prediction interval, and are not interpreted as estimates of effect size.

### 2.7. Population-Stratified Analysis

For the population-stratified component (Section 3.6), we admitted only estimates expressed in absolute units: g/kg actual body weight per day, g/kg ideal or adjusted body weight per day, or g/day. Percentage-of-energy contrasts were excluded entirely. Two rows of the population table are exceptions, admitted because the population has no estimate in absolute protein units and is stated as such in the table. One is an energy-intake contrast in maintenance hemodialysis. The other is a percentage-of-requirement contrast in disease-related malnutrition, where the requirement, which is a percentage of (1.5 g/kg/day), is given. Neither entered any pooled estimate, for the reason set out in the Introduction. Estimates were tabulated by clinical population with the direction of effect recorded explicitly, because the sign of the association is not constant across populations. No pooling was attempted within population strata.

### 2.8. Declared Deviations from PRISMA 2020

Six deviations are declared, each set out in full in Document S3. The first is no protocol registration; the protocol is supplied instead as Document S2, in PROSPERO field format. The second is single-reviewer screening with a duplicate-screened validation sample (Section 2.9). The third is a single-database analytic corpus: every pooled estimate comes from the PubMed search of Table S1, with Europe PMC, Semantic Scholar and ClinicalTrials.gov used only to test its coverage and to supply the validation sample (Table S1b). The fourth is risk of bias appraised after selection, so that no study was removed on the basis of its rating. The fifth is capped searches: twenty-three of fifty-eight queries were truncated by the retrieval cap, severely so for the microbiome tranche (1250 of 20,872 matched records retrieved) and the dietary-assessment tranche (597 of 1700). That is why Section 3.11 and Section 3.12 are orientations over a sample rather than syntheses of a field, and why Section 3.12 is excluded from the recommendations. The sixth is an open-access constraint: 4074 of 7544 records (54.0%) were screened on abstract alone, under-representing estimates reported only in paywalled full texts by an amount that cannot be quantified.

PRISMA 2020 is a reporting guideline rather than a conduct standard. The applicable conduct standard for a rapid review is the guidance of the Cochrane Rapid Reviews Methods Group [29], updated for reviews of effectiveness in 2024 [30]. Table S13 maps this review against it item by item. Four items are not met or only partly met: CENTRAL and Embase were not searched, no second reviewer screened the excluded records, full-text screening was not duplicated, and extraction was not independently checked. Risk of bias, by contrast, was rated in full duplicate rather than rated once and verified.

**Table 1:** Physical activity and all-cause mortality: pooled stratum, k = 16.

Cohort	Year	Population	Contrast	HR (95% CI)
Generally healthy				
Cho et al. [31]	2023	Korean adults (KNHANES 2007–2013)	≥50 vs. 0 MET-h/week MVPA	0.82 (0.70–0.95)
Wang et al. [32]	2025	Chinese adults (*n* = 109,407)	highest vs. inactive quartile, total PA	0.77 (0.69–0.85)
Moshkovits et al. [33]	2025	community-dwelling, 20-year follow-up	currently active vs. sedentary	0.72 (0.59–0.88)
Ke et al. [34]	2026	Chinese adults, 47,281 deaths, 12.1 y	highest vs. lowest quintile, total PA	0.69 (0.67–0.71)
Kim et al. [35]	2017	Korean adults, 8-year prospective	meets PA guidelines vs. inactive	0.68 (0.58–0.81)
Subtotal, general			k = 5, *I*^2^ = 51%	0.73 (0.68–0.78)
Established chronic disease				
Wu et al. [20]	2025	diabetes/prediabetes	≥600 vs. <600 MET-min/week	0.86 (0.77–0.95)
Bos et al. [36]	2025	cancer patients (Lifelines)	meets PA guidelines vs. no PA	0.84 (0.72–0.98)
Wu et al. [37]	2025	adults with diabetes	regularly active vs. inactive	0.83 (0.78–0.87)
Yu et al. [38]	2022	older adults with heart failure	500–999 MET-min/week (guideline range) vs. inactive	0.81 (0.70–0.95)
Zhu et al. [39]	2026	long-term cancer survivors	full adherence to PA recommendation	0.78 (0.70–0.86)
Kim et al. [22]	2023	non-alcoholic fatty liver disease	≥150 min/week total PA vs. below	0.74 (0.60–0.90)
Zhu et al. [40]	2025	stroke survivors	meets MVPA recommendation vs. not	0.73 (0.64–0.83)
Li et al. [21]	2026	cardiac patients	≥150 MET-min/week vs. below	0.71 (0.63–0.80)
Cao et al. [41]	2020	breast cancer survivors	>6.9 MET-h/week MVPA (active) vs. inactive	0.66 (0.43–0.99)
Xiao et al. [23]	2025	adults with depression	≥600 vs. <600 MET-min/week	0.60 (0.47–0.76)
Moy et al. [19]	2016	after COPD hospitalization	≥150 min/week MVPA vs. inactive	0.53 (0.34–0.84)
Subtotal, clinical			k = 11, *I*^2^ = 47%	0.77 (0.73–0.82)
Pooled (random effects)			k = 16, *I*^2^ = 64%	0.76 (0.72–0.79)

### 2.9. Risk of Bias and Certainty of Evidence

Each design was appraised with the instrument built for it: RoB 2 for randomized trials, ROBINS-E for observational exposure studies, and AMSTAR 2 for systematic reviews and meta-analyses. A Newcastle–Ottawa cross-check on the same observational set was added at revision; it was scored by a single rater and informs no grade and no conclusion. Certainty was rated with GRADE, with every up- and down-grade justified. An AMSTAR 2 rating of Critically low means that the pooled magnitude reported by that review is not treated as reliable and that no conclusion rests on it alone. The estimate is still reported, with its rating stated where it is used, and the certainty of any body of evidence to which it contributes is rated down accordingly. Overall AMSTAR 2 ratings follow the instrument’s rule mechanically from the critical-domain judgments recorded in Table S9. Five were recalculated at revision, when an internal audit found that they had been assigned as global impressions instead (Table S9). TRIPOD and PROBAST were considered and found inapplicable, because no included study reports a prediction model. Each study appraised with ROBINS-E, RoB 2 or AMSTAR 2 was rated independently from the published report by two authors (V.G.-O. and C.D.), with a third (K.S.) resolving disagreements. Ratings are the review team’s judgment of the published record, not of the underlying data. Document S3 gives the domain definitions, the instrument-selection reasoning and the full rationale for setting TRIPOD and PROBAST aside. The completed domain-level grids are supplied as Tables S7, S7b, S8 and S9, with the GRADE summary in Table S10, so that a reader who disagrees with a judgment can see which domain it turns on.

Reproducibility of the screening step: Because screening was otherwise single-reviewer (Section 2.8), a stratified random sample of 888 records was screened independently on title and abstract by V.G.-O. and C.D. The one record admitted by the top-up search after the sample had been drawn was added to it, giving 889 records in duplicate. The sample was drawn from the enlarged multi-source corpus rather than from the analytic corpus alone, and after a title-level exposure filter. It therefore describes screening decisions on records already naming an exposure in their title. Cohen’s κ was 0.50 (95% CI 0.42–0.57) with PABAK 0.67. Both are reported because the marginal distribution is heavily skewed, so κ is depressed by the prevalence paradox rather than by poor agreement. Raw agreement, the stability of κ across strata, the adjudication of the 149 discrepancies and their outcome are in Document S4.

## 3. Results

Six strata were defined, and three were pooled. Only two are large enough for between-study variance to be estimated with any confidence: meeting activity guidelines (k = 16) and total protein (k = 5). The other four have k ≤ 4 and are exploratory (Section 2.6). The activity stratum survives restriction to studies not rated high risk of bias, whereas each protein stratum falls to a single study (Section 3.5). No randomized estimate of protein with resistance training reports mortality (Section 3.9). The pooled mortality estimates below are therefore observational associations, reported with the k that produced them.

### 3.1. Study Selection

Fifty-eight logged queries across ten tranches matched 34,510 records. A total of 9824 were retrieved and screened. The shortfall is attributable entirely to the twenty-three capped queries in Section 2.8, and is dominated by the two exploratory tranches described there. After removal of 2280 cross-query duplicates, 7544 unique records entered screening. Open-access full text was obtained for 3470 records (46.0%); the remaining 4074 were screened on abstract.

Deterministic extraction recovered 8304 effect estimates with 95% confidence intervals from 1269 papers, in measure families that were never merged: 6005 ratio estimates and 2299 mean-difference estimates.

After contextual reading, 30 estimates across six strata met a stratum’s contrast definition, and the three strata with at least three estimates were pooled. A further 23 estimates across 10 clinical populations entered the absolute-intake stratified analysis, and 32 estimates from 16 trials and pooled analyses entered the randomized evidence table (Section 3.9). Twenty-six observational studies were appraised with ROBINS-E, two trials with RoB 2, and fifteen reviews with AMSTAR 2 (Section 3.10).

Figure 1 shows the flow.

### 3.2. Physical Activity and All-Cause Mortality

**Figure 2:**
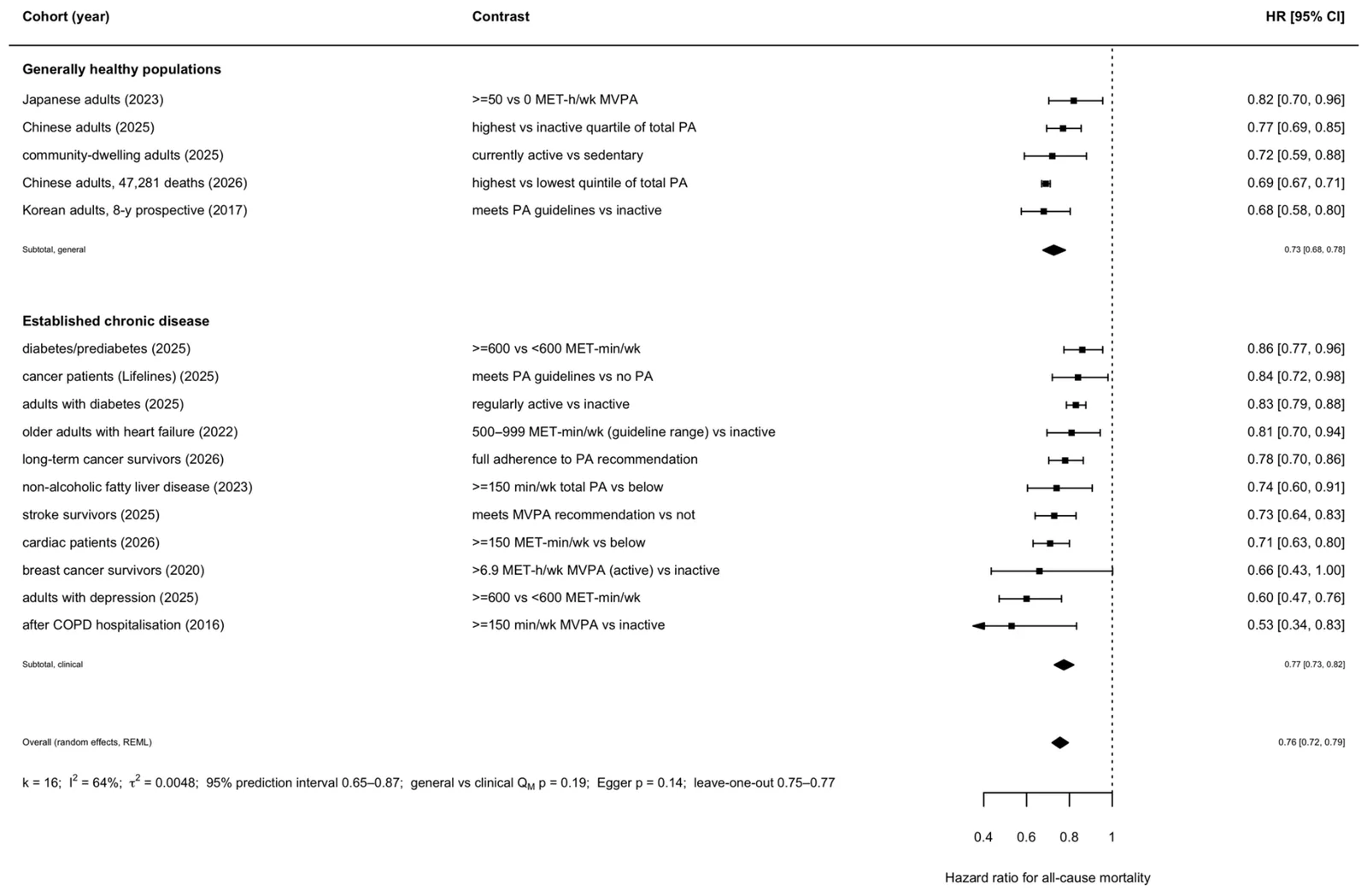
Physical activity and all-cause mortality: random-effects (REML) pooled estimate across sixteen prospective cohorts sharing a single contrast definition, namely the guideline-meeting or most-active category versus an inactive or below-guideline reference. Cohorts are stratified by whether they are generally healthy or have established chronic disease.

Sixteen prospective cohorts compared the most-active or guideline-meeting category with an inactive or below-guideline reference. The pooled association with all-cause mortality is HR 0.76 (0.72–0.79), k = 16, *I*^2^ = 64% (Table 1, Figure 2). The stratum, its leave-one-out robustness and the sources of its heterogeneity are given in Document S4.

Seven of the sixteen cohorts were added at internal revision (Table S15).

No single cohort determines the pooled estimate: leave-one-out estimates ranged from 0.747 to 0.770, and refitting with the Knapp–Hartung adjustment gave HR 0.76 (0.72–0.80). Egger’s regression was not significant (z = −1.46, *p* = 0.144), and trim-and-fill imputed three studies with an adjusted HR of 0.77 (0.73–0.81). With 16 studies, neither result excludes publication bias (Section 2.6).

Heterogeneity is substantial and imprecisely estimated: *I*^2^ = 64% (95% CI 29–89%), τ^2^ = 0.0048 (0.0011–0.0221), Q = 60.45 (*df* = 15, *p* < 0.001). The 95% prediction interval is 0.65–0.87. All sixteen point estimates and confidence intervals lie below one, so the heterogeneity concerns the size of the association rather than its direction. No measured study characteristic explained it in exploratory meta-regression (Table S14). All five moderators were non-significant: exposure definition (Q_M *p* = 0.77), region (*p* = 0.33), publication year (*p* = 0.34), the standard error of the estimate (*p* = 0.14) and length of follow-up (*p* = 0.24; permutation *p* = 0.29, 5000 iterations). The standard-error model is Egger’s regression restated as a moderator and is counted once. Omitting the largest and most precise cohort [34] lowered *I*^2^ to 39% and moved the pooled estimate to 0.770. Part of the heterogeneity therefore reflects one very precise estimate; the remainder is unexplained. Activity was self-reported in every cohort for which the measurement method could be established, so the measurement method could not be examined.

General versus clinical populations: General-population cohorts pooled to HR 0.73 (0.68–0.78), k = 5. Clinical cohorts pooled to HR 0.77 (0.73–0.82), k = 11 (Q_M = 1.71, *df* = 1, *p* = 0.19). This comparison depends on how overlapping cohorts are handled. Four of the clinical estimates come from overlapping NHANES cycles (Section 2.6). Removing them, or combining them into one estimate, left the overall pooled estimate unchanged (HR 0.76, 0.72–0.80, k = 12 and k = 13). The clinical subgroup, however, moved to 0.80 (0.76–0.84) and 0.79 (0.75–0.83), and the subgroup difference reached nominal significance (Q_M *p* = 0.049 and *p* = 0.039, unadjusted for multiple testing). Retaining one NHANES estimate at a time gave *p*-values from 0.013 to 0.21 (Table S14). Higher activity is therefore associated with lower mortality in people with established chronic disease as well as in the general population. These data do not show that the association is of the same magnitude: a modestly weaker association in clinical cohorts cannot be excluded (Section 5.4, Document S4).

### 3.3. Where the Activity Dose–Response Curve Bends

Lower mortality was observed at activity levels below the guideline threshold. In a pooled analysis of 661,137 adults, leisure-time activity below the recommended minimum was associated with HR 0.80 (0.78–0.82) compared with no leisure-time activity [10]. In individual analyses, a statistically significant association was detectable from 10 min/week of accelerometer-measured MVPA [15]. An umbrella review of step counts reported an inverse association from about 3143 steps/day, with HR 0.91 (0.87–0.95) per further 1000 steps [42]; a cohort of older adults reported age-specific cut-points [43]. A median of 5.3 bouts/day of vigorous intermittent activity was associated with HR 0.56 (0.39–0.82) [44]. Muscle-strengthening activity was associated with an additional reduction that did not increase above 120 min/week [45]. The shape is L-shaped rather than linear. These thresholds are properties of the source analyses—their models, reference categories, measurement methods and populations—and are not universal cut-points. Document S4 gives the cohorts one by one.

In the accelerometer analysis, the association flattened beyond about 500 min/week [15]. In older adults, optimal step cut-points were about 1824 steps/day above age 75 and 4128 below [43]. Resistance training was associated with HR 0.87 (0.81–0.95) at 90–119 min/week after adjustment for aerobic activity, over 30 years of follow-up [45].

The lowest level at which an association becomes detectable is not the level at which it is largest; the gradient continues above it (Document S4).

### 3.4. Odds Ratios Computed from Primary 2 × 2 Data

Of 688 papers carrying raw event counts, one reported a genuine 2 × 2 table for total physical activity and mortality. Crude odds ratios could therefore be computed and compared with the published adjusted hazard ratios. In that study, the crude estimates are more extreme than the adjusted ones, which is what confounding adjustment is expected to do. The comparison is reported as a check on the extraction pipeline rather than as a result (the crude-odds-ratio table in Document S4).

### 3.5. Protein: Amount Versus Source

Total protein intake shows no consistent association with all-cause mortality in published syntheses. Three syntheses addressed the general population. One reported RR 0.97 (0.93–1.02) across 25 cohorts and 666,129 participants [46]. A second reported a small inverse association, HR 0.94 (0.89–0.99), across 715,128 participants [47]. A third found no clear association across 12 cohorts [48]. Our pool of highest-versus-lowest quantile contrasts gave HR 0.91 (0.64–1.27), k = 5, *I*^2^ = 81%, τ^2^ = 0.111, Q = 14.19 (*p* = 0.007), *p* = 0.57. The 95% prediction interval is 0.43–1.89. The absence of a consistent association does not show that the amount of protein is unimportant. It may instead reflect associations in opposite directions in different populations, quantile contrasts that correspond to different absolute intakes in different cohorts, and measurement error in dietary instruments.

Read across populations in absolute intake units, the direction of association differs. Of the sixteen populations assembled in absolute intake units (Section 5.5), lower intake was associated with worse outcomes in nine and higher intake in two: community-dwelling adults free of chronic disease at baseline at habitual intakes ≥1.8 g/kg/day, and the acute phase of critical illness. Two showed higher risk at both ends of the range, and in three the association concerned protein source rather than amount. These are qualitative comparisons across separate observational studies with different outcomes, not a pooled estimate. They suggest that, in populations near or below adequacy, low intake may be the more common risk (Document S4).

**Figure 3:**
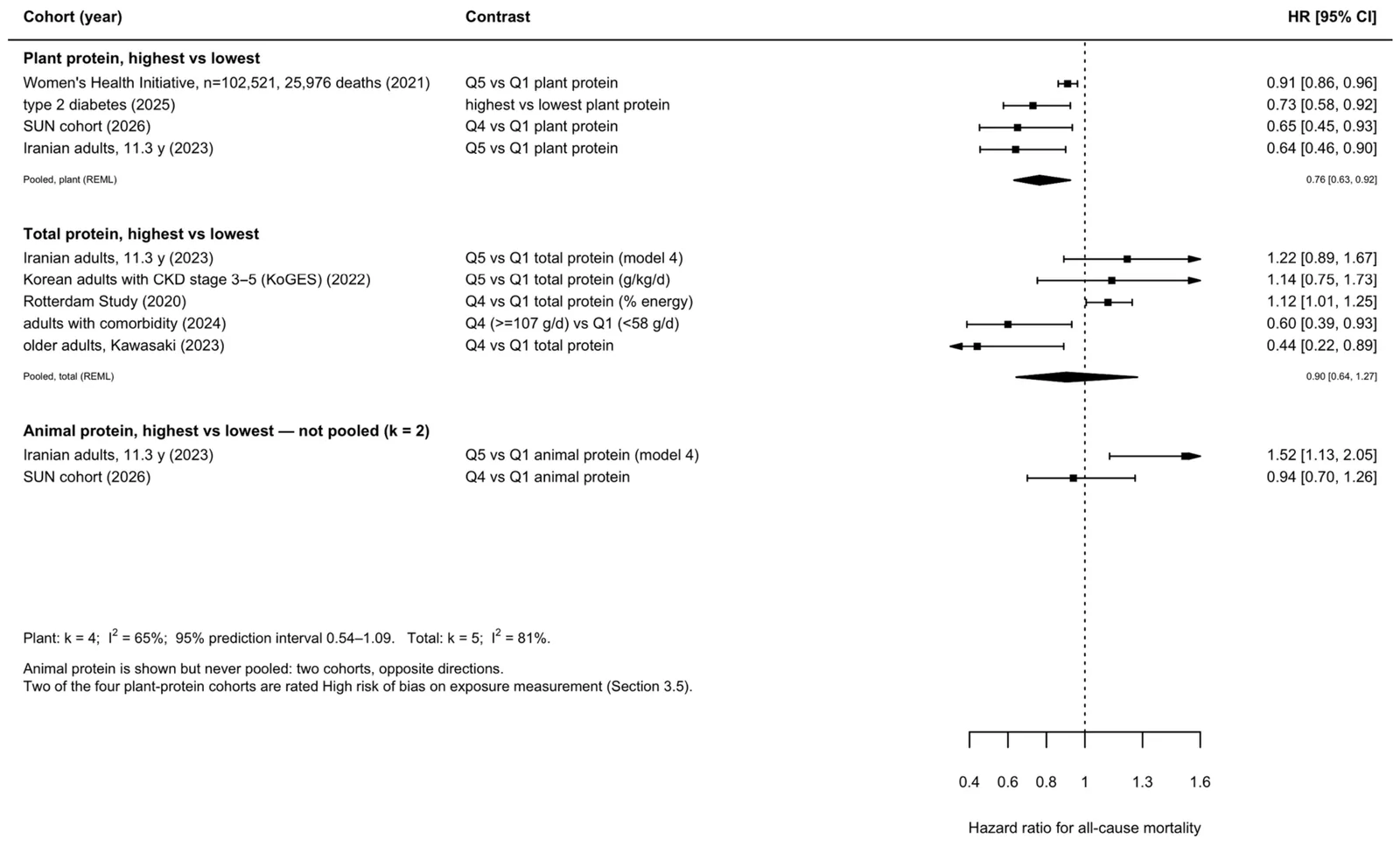
Dietary protein and all-cause mortality by protein source: random-effects (REML) pooled estimates, highest-versus-lowest quantile contrasts only. The diamonds should be read with the last two columns of Table 2 beside them. Every protein stratum shown here falls below the three-study minimum when cohorts rated high risk of bias are removed, so none of these pooled magnitudes survives the review’s own sensitivity restriction. The estimates therefore indicate direction rather than magnitude. An exploratory split of the total-protein stratum by setting shows no difference between generally healthy cohorts (0.93, 0.55–1.57) and chronic-disease cohorts (0.83, 0.44–1.56), Q_M *p* = 0.79 (Section 3.5). The single total-protein cohort not rated high risk shows HR 1.12 (1.01–1.25).

Plant protein: an inverse association of very low certainty. A top-up search at revision added the Women’s Health Initiative, the largest cohort in the stratum (102,521 women, 25,976 deaths; plant protein HR 0.91, 0.86–0.96, highest-versus-lowest quintile) [49]. The pooled estimate across four cohorts is HR 0.76 (0.63–0.93), *I*^2^ = 65.1%, with a prediction interval of 0.54–1.09 that includes one (Table 2, Figure 3). The earlier three-cohort estimate was 0.69 (0.58–0.82) with *I*^2^ = 0.0%. Certainty is very low (Section 3.10). All four cohorts used food-frequency questionnaires, confounding by overall diet quality is unresolved, and a single cohort remains when studies at high risk of bias are excluded. The pooled estimate indicates a direction of association, not a reliable magnitude (Document S4).

Animal protein shows no consistent association. The pooled 32-cohort estimate is RR.

Three independent meta-analyses report a pooled estimate of 0.92 for plant protein and all-cause mortality [46,47,48]. They pool graded or per-increment exposures across many cohorts, whereas we admitted only extreme-quantile contrasts from four, so the estimates describe different contrasts (Document S4). In 131,342 participants of the Nurses’ Health Study and the Health Professionals Follow-up Study, plant protein was associated with HR 0.90 (0.86–0.95) per 3% of energy, whereas animal protein was not associated with all-cause mortality. Both associations were confined to participants with a lifestyle risk factor [50] (Section 4.4).

1.04 (0.97–1.12), and 1.000 (0.950–1.053) after removal of two influential outliers [46]. Any case for shifting toward plant protein therefore rests on the inverse association with plant protein, not on an association with animal protein.

Pooled dose–response analyses do not identify an intake above which mortality rises. Non-linear dose–response modeling in [46] found an inverse, significantly non-linear association of total protein with all-cause mortality (*p* = 0.003 for non-linearity), and no significant non-linearity for animal (*p* = 0.79) or plant protein (*p* = 0.18).

Risk-of-bias sensitivity analysis: Because ROBINS-E rated thirteen of twenty-six observational studies at high risk overall, every pooled stratum was re-run restricted to studies not rated high. The activity estimate was essentially unchanged: 0.762 (0.716–0.811), k = 9, against 0.756 (0.720–0.794) with all sixteen. The protein strata each fell to a single study and could not be pooled under the three-study minimum. Measurement of the exposure (ROBINS-E domain D2) is the reason in most cases, covering three of the four high-rated cohorts in the total-protein stratum. The fourth, the KoGES cohort in chronic kidney disease, is rated high on confounding (D1), because protein restriction there is prescribed to those with worse disease. One plant-protein cohort, the Women’s Health Initiative, was admitted after the duplicate appraisal and has not been rated. Under either possible rating, the restricted plant-protein stratum is not poolable (k = 1 or k = 2, below the three-study minimum) (Document S1, Document S4).

Estimates remaining after restriction: For plant and animal protein, the remaining estimates point the same way as their pools (plant 0.65, 0.45–0.93; animal 0.94, 0.70–1.26, both from the SUN cohort). The direction therefore does not depend on the high-risk studies, although the precision does. Total protein differs. Its one remaining cohort is the Rotterdam Study, rated Some concerns and the best-characterized protein cohort in the set. It reports HR 1.12 (1.01–1.25) [51], an association with higher mortality, whereas the five-cohort pool returns 0.91 (0.64–1.27). The only well-measured cohort in the total-protein stratum therefore does not show a null association (Document S4).

Exploratory split of the total-protein stratum by setting: Applying the setting moderator recorded in the protocol for physical activity, the five cohorts gave 0.93 (0.55–1.57), *I*^2^ = 87.9%, in generally healthy cohorts and 0.83 (0.44–1.56), *I*^2^ = 76.9%, in chronic disease cohorts. There was no difference between them (Q_M = 0.07, *p* = 0.79). The chronic-disease side has k = 2 and is below the three-study minimum; it is reported as a subgroup description rather than as a pooled result (Section 2.6). The split was applied to the protein stratum only at revision (Table S15). One cohort was recoded during the revision audit after its source population was re-read, and one estimate was removed from the stratum because its contrast did not match the stratum definition (Document S1). It is therefore not evidence that the association reverses between healthy and chronically ill populations, and no recommendation in Section 5.5 depends on it. The population-specific windows are built from the per-population estimates of Table 3 and of the population-stratified table in Section 5.5, neither of which passes through the total-protein pool.

### 3.6. Population-Stratified Analysis in Absolute Units

Restricting to absolute intake and stratifying by population shows that the direction of the protein–outcome association differs across populations (Table 3).

The hemodialysis cohort ([52]; 45-month median follow-up, 354 deaths) is the only study in the corpus to report both low and high intake against a common reference. Risk was lowest at 1.0–1.4 g/kg ideal body weight per day, with a steeper association at low intake (HR 1.84) than at high intake (HR 1.49). As a single observational cohort, it supports only a provisional window (Section 5.5).

The critically ill rows are the only randomized mortality estimates in the table. Higher protein delivery showed no difference in mortality overall (RR 1.01) and higher mortality in the acute-kidney-injury subgroup (1.29, 1.05–1.58), so this evidence does not support raising protein in acute illness. In kidney transplant recipients, muscle-mass recovery in the first year tracked intake, with a break-even estimated at 0.72 g/kg ideal body weight per day.

In CKD patients over 65, moderate compared with low intake was associated with lower all-cause mortality (HR 0.14, 0.02–0.69) [59], in a single cohort with a wide interval. Protein-energy wasting may matter more than filtration burden in older patients.

### 3.7. Type 2 Diabetes: Protein Source and Amount

Findings in this section derive partly from a capped query (Section 2.8) and are recall-oriented.

The umbrella review [65] (Eur J Nutr 2024; eight systematic reviews, six with meta-analysis) reported three things. Six of eight reviews found risk increases with total and animal protein (animal protein RR 1.13, 1.08–1.19 to 1.19, 1.11–1.28). There was a U-shaped relationship with maximum risk reduction at approximately 5–6% of energy from plant protein (≈25–30 g/day on a 2000 kcal diet). And randomized trials of HbA1c, fasting glucose and fasting insulin “do not support a clear biological link with T2D risk.” All included meta-analyses received a low NutriGrade rating.

In one meta-analysis, associations differed more by food source than by protein amount [66]. Processed meat per 50 g/day gave RR 1.41 (1.24–1.60) and red meat per 50 g/day gave RR 1.11 (1.06–1.16). Total protein, highest versus lowest, gave RR 1.11 (1.05–1.16), plant protein RR 0.93 (0.87–0.99, *I*^2^ = 0%) and yogurt RR 0.83 (0.77–0.89).

Two modifiers may explain apparently discordant cohorts. Adiposity: Ref. [67] found moderate versus lower total protein associated with a lower risk of type 2 diabetes or impaired fasting glucose, HR 0.79 (0.66–0.94), the opposite direction to the meta-analyses. Higher animal protein combined with lower BMI gave HR 0.50 (0.40–0.62). Background dietary pattern: Ref. [68] found that the direction differs. Protein was inversely related to T2DM in a “legumes and seafood” pattern (OR 0.58, 0.33–0.96) and positively related in a “red meat” pattern.

Further datasets are consistent with a role for source. A healthful plant-based index was inversely and an unhealthful one positively associated with type 2 diabetes in the same cohorts [69], and protein from meat, but not from vegetables or cereals, was associated with diabetes (OR 1.34, 1.02–1.76) [70]. A single-amino-acid signal is hypothesis-generating [71]. Document S4 gives these in full, with the remaining source in this tranche [72].

### 3.8. Gestational Diabetes

In pregnancy, the association runs opposite to that in older adults. High total protein during pregnancy gave RR 2.24 (1.67–3.00) [73] and animal protein RR 2.31 (1.43–3.73). Each 5% of energy from total protein gave RR 1.20 (1.09–1.33) [74], and plant protein Q4 versus Q1 gave OR 0.43 (0.25–0.73) [75]. Ref. [76] used a middle quintile as reference and found Q5 aOR 1.48 (1.06–2.07) with Q1 aOR 0.69 (0.48–0.996).

These observational associations suggest that gestational diabetes requires its own guidance rather than the higher protein advice given to older adults.

### 3.9. The Randomized Layer

Sixteen trials and pooled analyses of trials entered a separate evidence table (Table 4; full version, Table S11), which is not pooled (Section 2.5).

Randomized exercise evidence on all-cause mortality is imprecise. In conventional pairwise meta-analyses of exercise-based cardiac rehabilitation, relative risks for all-cause mortality were 0.87–0.91 in coronary heart disease across short-, medium- and long-term follow-up [78] and 0.93 in heart failure [79]. An IPD meta-analysis in heart failure gave HR 0.83 (0.67–1.04) [80]. Every one of these intervals crosses the null. A network meta-analysis comparing modes of delivery found a reduction only for center-based cardiac rehabilitation, RR 0.76 (0.64–0.90) [81]. That estimate combines direct and indirect comparisons, comes from a review rated critically low on AMSTAR 2 (Table S9), and is not directly comparable with the pairwise estimates. The individual-participant estimate is from a review rated critically low as well. The two randomized estimates furthest from the null in this body of evidence are therefore also the two from the least reliable reviews. The one trial in generally healthy older adults was also imprecise, HR 0.63 (0.33–1.20) [77]. Table 4 and Document S4 give the estimates trial by trial.

The randomized point estimates lie below one, but they are not directly comparable with the pooled observational HR of 0.76. The trials estimate the effect of assignment to a structured program in selected cardiac populations, over limited follow-up and with exercise in control groups. The cohorts, by contrast, compare habitual activity levels in broader populations. Agreement in direction therefore does not exclude residual confounding in the observational estimate. Only the network estimate for center-based rehabilitation excludes the null, in a single disease and a single delivery format. The randomized evidence therefore does not establish an effect of exercise on all-cause mortality in the general population.

The same reviews report reductions in cardiovascular events: cardiovascular mortality RR 0.58 (0.43–0.78) at long-term follow-up and myocardial infarction RR 0.67 (0.50–0.90) [78]. All-cause hospital admission in heart failure gave RR 0.69 (0.56–0.86) [79]. Randomized evidence for exercise is therefore stronger for cardiovascular mortality and hospitalization than for all-cause mortality.

Protein supplementation increased strength with resistance training but not without it. Added protein changed strength by +0.13% (−1.53 to 1.79) without resistance training and by +2.01% (1.09–2.93) with it, and the effect scales with the increment above habitual intake [83]. Protein alone was associated with a small increase in muscle mass in community-dwelling older adults (SMD 0.116, 0.032–0.200) [93]. Document S4 gives the dose–response detail and the supporting syntheses.

This is the most consistent randomized finding in the review, and it is conditional: in these trials, the effect of added protein on strength depended on concurrent resistance training.

High-protein diets showed no evidence of renal harm in people without kidney disease over the duration of the trials. In 22 randomized trials in adults without kidney disease, high protein raised eGFR (SMD 0.39, 0.09–0.69) while serum creatinine was unchanged (SMD 0.08, −0.09 to 0.25) [86]. That pattern is consistent with hemodynamic adaptation rather than injury, although the trials were short and eGFR and creatinine are surrogate markers (Document S4).

In established kidney disease, the effects of protein restriction differ by endpoint. For kidney outcomes, low-protein diets reduced kidney failure (OR 0.59, 0.41–0.85) and end-stage renal disease (OR 0.64, 0.43–0.96) in a meta-analysis of 19 trials [87]. In non-diabetic CKD, very-low-protein diets reduced end-stage kidney disease compared with low-protein diets (RR 0.65, 0.49–0.85), whereas low-protein compared with normal-protein diets did not (RR 1.05, 0.73–1.53) [88]. For mortality, none of the four available estimates showed a difference, and all were imprecise. They were RR 0.77 (0.51–1.18) and 1.26 (0.62–2.54) [88], OR 1.17 (0.67–2.06) [87] and HR 0.95 (0.62–1.45) [89]. The trials differ in CKD stage, diabetes status, degree of restriction, adherence and monitoring of nutritional status, and few report nutritional harm, so their results cannot be summarized in a single statement. In diabetic kidney disease, the randomized evidence does not support restriction below 0.8 g/kg/day (Document S4).

### 3.10. Risk of Bias and Certainty

Twenty-six observational studies were appraised with ROBINS-E, two trials with RoB 2, and fifteen reviews with AMSTAR 2. The complete domain-level grids are Supplementary Tables S7–S9; the certainty ratings are in Supplementary Table S10 and are summarized in Table 5.

Thirteen of the twenty-six observational studies were rated High overall, and thirteen were rated Some concerns; none was rated Low. One overall rating was recalculated at revision. The UK Biobank cohort of very high intakes [56] had been rated High on the grounds of indirectness, which ROBINS-E does not assess and which is now carried in the certainty rating instead. No domain judgment changed, and no domain in that study is rated High. The reasons cluster into three recurring domains. These are D2 exposure measurement, where food-frequency instruments and single recalls stand in for habitual intake; D1 confounding by indication; and D7 selective reporting, where a large grid of exposure × outcome cells is reported and a review uses one. Document S4 gives the pattern study by study [93,94,95,96,97].

It is worth stating what this pattern does not do to the headline result. Removing any single cohort from the activity stratum moves the pooled hazard ratio by no more than 0.014 (leave-one-out range 0.747–0.770; Table S14).

On AMSTAR 2, eight reviews were rated High confidence, two Low and five Critically low, with none Moderate. Five of these ratings were recalculated at revision. Applying the instrument’s rule to the critical-domain judgments already recorded moved [80,81,87] to Critically low and [85,92] to Low, without changing any domain judgment (Table S9). Two of the five Critically low reviews are critically flawed in the pooling itself and support nothing [84,90]. The other three are flawed in protocol registration, justification of exclusions and assessment of publication bias. Their estimates are reported with the rating stated at each point of use, and the affected certainty ratings are lowered accordingly (Section 2.9, Document S4).

Of the two randomized trials appraised with RoB 2, one was rated Some concerns and one high risk of bias (Table S8, Document S4).

Newcastle–Ottawa and ROBINS-E disagree: Scored on the same 26 studies, NOS returns a median of 7.5 stars out of nine, which would ordinarily read as high quality. ROBINS-E rates thirteen of those studies at high risk. The two instruments ask different questions. NOS scores design features, whereas ROBINS-E assesses the risk of bias in a specific exposure effect estimate, domain by domain, against a hypothetical target experiment, with explicit domains for confounding and exposure measurement [98]. Because confounding and exposure measurement are the main threats in this literature, ROBINS-E was designated the primary instrument before appraisal began, and NOS was added at revision as a cross-check. The NOS scores informed no certainty rating: every GRADE judgment in Table 5 uses the ROBINS-E ratings, so the discordance changed no grade. We report it rather than reconcile it, because it shows that an appraisal result depends on the instrument as well as on the study (Table S7b, Document S4).

Table 5 shows every up- and down-grade with its justification.

### 3.11. Incretin Therapy and Lean Mass

The number of people treated with incretin-based therapies is increasing rapidly, and their muscle outcomes bear directly on the protein question.

Lean mass: Across 19 randomized trials, GLP-1 receptor-based agonists reduced lean body mass by 1.02 kg (95% CI 0.57–1.46) more than control [9]. Other syntheses give estimates of 0.86–1.68 kg [99,100,101].

Two qualifications apply: The lean-mass loss is measured against control rather than against weight loss of the same size by other means. A proportionate loss of lean tissue is expected with any substantial weight loss. Lean mass is also not the same as muscle function, which these trials did not measure, so the clinical significance of the loss is not known [9] (Document S4).

Protein intake: One study measured intake directly in patients on these drugs. In the same people, protein was within the acceptable macronutrient distribution range as a percentage of energy but below requirement in g/kg [102]. Monitoring based on percentage of energy would not detect this (Document S4).

Possible countermeasures and their limits: Evidence from other populations is relevant but indirect. Protein supplementation combined with resistance training increases fat-free mass (MD 0.30 kg, 0.09–0.52) and one-repetition-maximum strength (MD 2.49, 0.64–4.33). In community-dwelling older adults with sarcopenia, the combination raises muscle mass by SMD 0.95 (0.13–1.78) [103]. A published practical framework suggests 1.2–1.6 g/kg/day, distributed across meals, with resistance training started early [103]. Its authors note that randomized trials have not yet tested protein dose strategies in patients receiving GLP-1 receptor agonists. These targets are therefore extrapolated from the obesity, caloric-restriction and sarcopenia literature rather than derived from incretin trials.

Two considerations complicate the extrapolation. Many people receiving these drugs have obesity or metabolic disease, in which the muscle protein response to a given dose may be blunted [104]. And the appetite suppression through which the drugs act may make a target of 1.6 g/kg/day, roughly 25–30 g of protein per meal, difficult to achieve [103].

Ongoing trials: LEAN-PREP is a six-month randomized controlled trial in adults with obesity starting semaglutide or tirzepatide. It compares resistance exercise and/or increased dietary protein (1.6 g/kg/day, at least 25 g per meal) with usual care, with muscle mass and physical function as endpoints [105]. It has not been reported. Until trials in this population are reported, protein targets for people receiving incretin therapy are extrapolations and should be labeled as such.

### 3.12. The Gut Microbiome

Dietary scores designed to favor the gut microbiota were associated with lower odds of frailty and of sarcopenia in two NHANES analyses (OR 0.70 in each) [106,107]. Mendelian randomization effects were small, however [108,109], and sequencing studies have not identified a consistent microbial signature [110,111]. The area is hypothesis-generating, contributes to no pooled estimate, and is excluded from the recommendations in Section 5 (Document S4).

### 3.13. Sarcopenia as the Outcome That Joins the Two Exposures

Both exposures in this review act on the same tissue. The chain from protein to survival can be set out link by link, with the design at each step:

- Protein → muscle: Randomized: Added protein was associated with a small increase in muscle mass without training (SMD 0.116, 0.032–0.200) [93]. It increased strength only with resistance training (+2.01% versus +0.13%) [83,112].
- Resistance training → muscle: Randomized: Resistance training increased handgrip strength in sarcopenia by 2.69 kg (1.78–3.61) in a network meta-analysis of 42 trials summarized in [92], and protein with training outperformed either alone [113].
- Muscle → sarcopenia: Definitional: Low muscle mass alone, low mass with low function, and sarcopenic obesity are different exposures.
- Sarcopenia → mortality: Observational: Consistently associated with higher mortality, but open to reverse causation, because illness lowers both intake and muscle.
- Protein with resistance training → mortality: Untested: No randomized estimate of protein with resistance training reports mortality in any population (Section 4.5). The chain is therefore incomplete, and no recommendation in Section 5 assumes otherwise.

The association with mortality depends on how sarcopenia is defined. Low muscle mass alone was associated with all-cause mortality, RR 1.36 (1.28–1.44) across 49 studies [114]. Definitions that also required low function gave an OR of 3.64 (2.94–4.51) in adults aged 60 and over [115]. In type 2 diabetes, sarcopenia was associated with HR 1.70 (1.21–2.40) across 21 cohorts [116]. These estimates use different measures and are not directly comparable, so no single number should be quoted as the risk associated with muscle loss. The associations were larger for combined phenotypes. In older outpatients with CKD, osteosarcopenia, rather than sarcopenia or low bone density alone, was associated with the composite of death, kidney replacement therapy or major cardiovascular events (HR 3.28, 1.52–7.08). Low handgrip strength was the component most strongly associated with it (HR 2.44, 1.46–4.08) [117]. In type 2 diabetes, sarcopenic obesity was associated with incident CKD (HR 1.77, 1.24–2.51) [118]. Screening that measures muscle mass alone may therefore miss the highest-risk groups (Document S4).

These estimates are observational, and muscle mass in a cohort is partly an index of the illness that leads to death. Leucine alone did not improve muscle mass or strength in older adults, and improvements were seen only in combination with resistance training or microbiota-targeted co-intervention [119]. The data show that sarcopenia is associated with poor outcomes, not that improving muscle extends life (Section 4.5).

## 4. Discussion

### 4.1. Principal Findings

Meeting physical activity guidelines was associated with lower all-cause mortality across sixteen cohorts (HR 0.76), with substantial unexplained heterogeneity. The association was also present in people with established chronic disease, although whether it is of the same size is uncertain. Lower mortality was observed at activity levels below the 150 min/week threshold. For protein, total intake showed no consistent association with mortality in the largest syntheses, whereas protein source was more consistently associated with outcomes. In absolute units, the direction of the association with intake differed between clinical populations. These observational findings do not support a single population-wide protein reference value; the population-specific windows proposed in Section 5.5 are provisional.

The randomized layer qualifies these findings. It agrees with the direction of the activity–mortality association, but only a network estimate restricted to center-based cardiac rehabilitation excludes the null for all-cause mortality. The reductions that recur across randomized syntheses are in cardiovascular death and hospitalization. For protein, the randomized evidence makes the claim conditional. Added protein increased strength with resistance training but not without it, had a small effect on muscle mass alone, and had a larger effect when more protein was added above habitual intake. Whether these findings apply to people receiving incretin therapy, who lose lean mass on treatment, has not been tested (Section 3.11).

### 4.2. The Denominator Problem

A recurring artifact can generate apparent “high protein is harmful” findings and has entered the secondary literature.

When protein is expressed per kilogram of actual body weight, the denominator is itself on the causal path wherever body size predicts the outcome. In obesity, heart failure and dialysis, this is not a technicality. At a BMI of 35, the same instruction produces intakes differing by roughly 40%, and a cohort that reports intake per kilogram of actual weight will attribute to protein what belongs to body size. This can produce apparent associations between high protein intake and higher observed risk that reflect body size, which is why every window in Table 6 names its denominator [120] (Document S4).

Estimates using ideal or adjusted body weight [52,54,55] are less affected by this problem, and none of them shows higher risk at higher intakes up to 1.4 g/kg.

The same structure can also exaggerate an association in the direction of lower risk, and the clearest instance is the largest obesity estimate in this review. In EPIC-Norfolk, sarcopenia was defined as the sex-specific 20th percentile of fat-free mass divided by BMI and obesity as a BMI of at least 30, while the exposure was protein per kilogram of actual body weight. Body size therefore enters the outcome definition and the denominator of the exposure at once. The reported odds ratio of 0.14 is expressed per 1 g/kg/day, an increment wider than the intake distribution it was estimated in. Energy intake was omitted from that model for collinearity [18]. We report it because it is the only quantitative observational estimate available in this population. It is labeled in Table 3 and Table 7 rather than left to carry weight its magnitude cannot bear.

### 4.3. Where Observational and Randomized Evidence Diverge

Observational and randomized estimates diverged in four places in this corpus.

1. Chronic kidney disease: In observational cohorts, lower intake was associated with a lower risk of the composite of kidney failure, dialysis or death (HR 0.77, 0.62–0.97). In randomized trials, restriction reduced end-stage kidney disease in some comparisons (RR 0.65, 0.49–0.85, very-low versus low-protein). It showed no difference in mortality in any of the four imprecise estimates (RR 0.77, 0.51–1.18; OR 1.17, 0.67–2.06; RR 1.26, 0.62–2.54; HR 0.95, 0.62–1.45) [87,88,89]. The observational composite and the randomized endpoints are not the same outcome, and the trials differ in stage, diabetes status and adherence (Section 3.9). The Cochrane review in diabetic kidney disease is consistent with this [60] (Table 7).
2. Type 2 diabetes incidence: Observational meta-analyses report total protein RR ≈ 1.11. Randomized trials of glycemic traits do not support a biological link, and protein restriction in diabetic nephropathy leaves glycated hemoglobin unchanged (SMD 0.17, −0.18 to 0.51) [90].
3. Very high total protein and mortality: An association with higher mortality at habitual intakes ≥1.8 g/kg/day in a single cohort (HR 1.39) has no counterpart in three large syntheses. This divergence is between observational sources rather than between designs.
4. Physical activity and all-cause mortality: Cohorts give HR 0.76 (0.72–0.79) across sixteen studies. Randomized estimates range from RR 0.76 to RR 0.93, with HR 0.83 in the one individual-participant analysis, and most intervals cross the null [77,78,79,80,81]. The estimates agree in direction but measure different quantities. Trials test assignment to structured programs in selected patients over limited follow-up, with exercise in control groups, whereas cohorts compare habitual activity over long follow-up and remain open to confounding (Document S4).

Confounding by indication or reverse causation may explain part of these divergences, since patients with worse renal function tend to eat less protein and also progress and die more often. Differences between the designs in populations, contrasts, endpoints and follow-up could also contribute. For physical activity, the limitations of the trials mean that imprecise randomized estimates do not by themselves refute the observational association, and agreement in direction does not exclude confounding. Because the two designs have different limitations, the randomized tranche was added, and the two layers are reported side by side rather than merged.

### 4.4. Red Meat, the Food Matrix, and What the Cohorts Cannot Tell Us

The strongest single limitation of the protein literature is one that dietary-pattern research has long recognized but that protein-specific analyses rarely confront: a protein food is never eaten alone.

The evidence assembled here associates red and processed meat with higher risk. Red meat gives HR 1.12 (1.04–1.20) for all-cause mortality, with each 50 g/day associated with 4% higher cardiovascular and 2% higher cancer mortality [121]. But the exposure “red meat intake” as measured in a food-frequency questionnaire aggregates over radically different meals. A steak grilled and served with a large salad, and the same weight of beef fried in butter and served with chips, enter the same exposure category. They differ in total energy, in saturated fat, in cooking-derived heterocyclic amines and advanced glycation end-products, and in fiber and polyphenol co-consumption. Over years, they also differ in the probability of obesity, which is itself on the causal path to most of the measured outcomes.

The corpus quantifies this concern in one place. Ref. [122] found that a Western pattern (dairy, baked/fried food and white meat) was associated with gestational diabetes at aOR 4.40 (1.58–12.22) relative to a prudent pattern, and a traditional pattern that included red meat at aOR 4.88 (1.79–13.32). That is several times larger than any single protein-source estimate in this review. Ref. [68] similarly found the protein–T2DM association to reverse direction according to the background dietary pattern (OR 0.58 in a legumes-and-seafood pattern; positive in a red-meat pattern). The accompanying diet may matter as much as the meat itself.

Two consequences follow.

First, isocaloric-substitution analyses are the most interpretable design in this literature, because they specify what the protein replaces rather than leaving the comparator implicit. Substituting plant for animal protein is associated with HR 0.91 (0.87–0.94) across nine cohorts and 1,036,799 participants [123]. Replacing 3% of energy with plant protein is associated with a larger reduction when it replaces processed meat (0.54, 0.38–0.75) than unprocessed red meat (0.66, 0.55–0.80) [124]. Document S4 sets out what substitution does and does not resolve.

Second, the associations are stronger for processed meat. Processed meat is more strongly associated than unprocessed red meat with type 2 diabetes (RR 1.41 versus 1.11 per 50 g/day). The substitution association is likewise larger for processed meat (0.54) than for red meat (0.66). A recommendation targeting ultra-processed meat products may therefore be better supported and less affected by the food-matrix confounding described above. The processing itself—nitrite curing, high sodium, emulsified fat, extended shelf life—is a property of the product rather than of the meal it sits in.

The association between unprocessed red meat and mortality is small and sensitive to adjustment, which is one reason the recommendation in Section 5.7 is limited to processed products. Our corpus also contained almost no direct evidence on cooking method (Section 5.8; Document S4).

### 4.5. Combined Physical Activity and Protein: Tested on Function, Untested on Mortality

The interaction between physical activity and protein has not been tested against mortality, but it has been tested on muscle outcomes in randomized trials.

On muscle strength, the interaction has been tested in trials. Added protein raises muscle strength when resistance training is present and not when it is absent (+2.01% versus +0.13%). The effect scales with the increment above habitual intake [83,112,125] (Document S4).

Protein alone has a small effect on muscle mass. Where resistance training cannot be delivered—frailty, disease-related malnutrition, immobility, institutional care—protein supplementation was associated with a small increase in muscle mass (SMD 0.116, 0.032–0.200) [93]. The conditionality of the strength finding is therefore not in itself a reason to withhold protein [126] (Document S4).

Acute illness is different. In critical illness, higher protein delivery was not associated with lower mortality and was associated with higher mortality in acute kidney injury (Section 3.9; Document S4).

Trials would be most informative. The higher risks documented in Section 3.13 are concentrated in older, multimorbid and sarcopenic-obese populations. A trial of the combination would have the highest event rate there.

**Figure 4:**
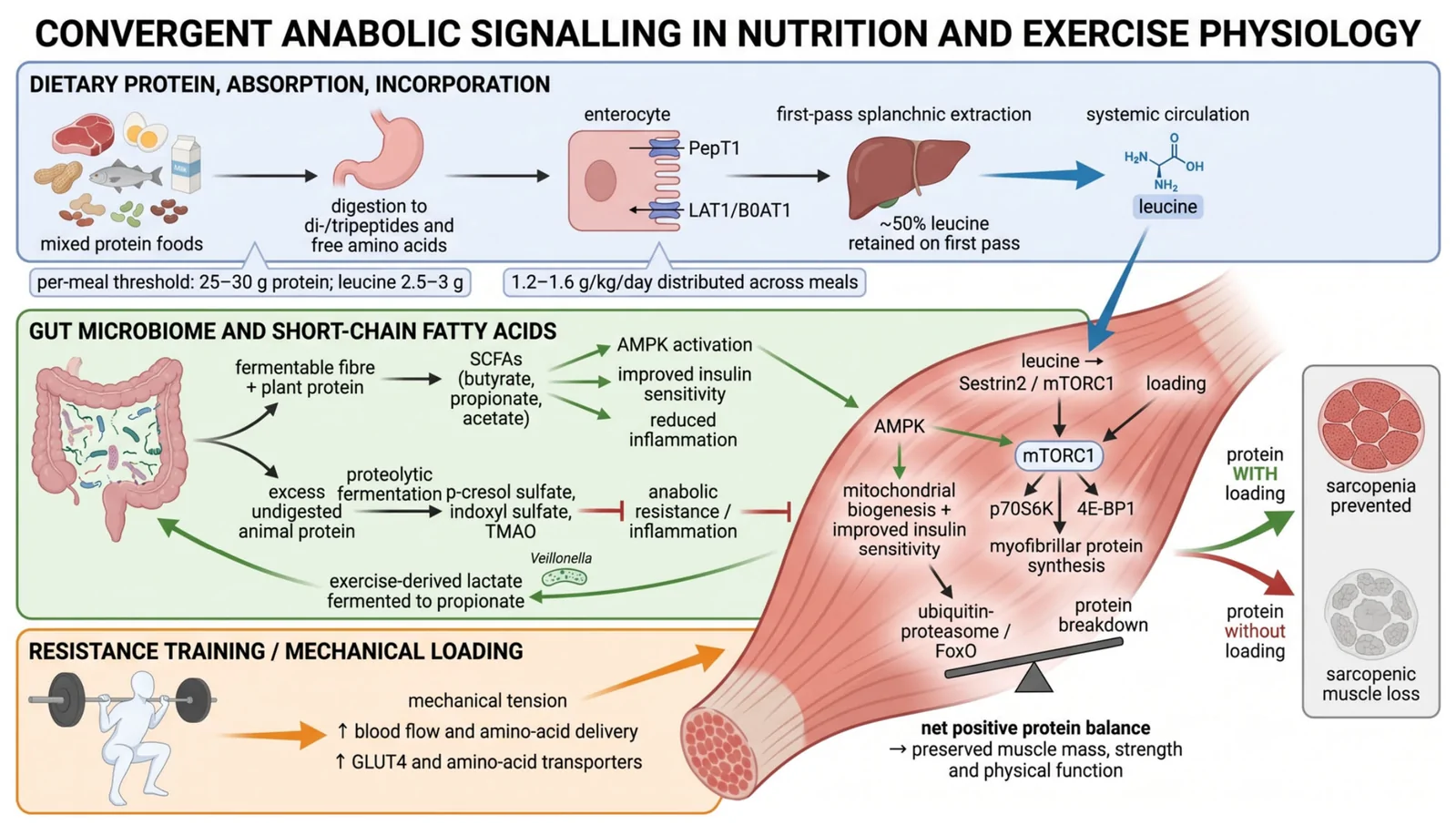
Convergent anabolic signaling (schematic). Dietary protein supplies amino acids whose leucine signal to mTORC1 depends on the per-meal dose after splanchnic extraction. The colonic microbiota ferments fiber and plant protein to short-chain fatty acids, and undigested protein to *p*-cresol sulfate, indoxyl sulfate and trimethylamine *N*-oxide, while exercise-derived lactate is fermented to propionate. Mechanical loading adds tension, blood flow and amino acid delivery. The inputs converge on the balance of muscle protein synthesis and breakdown. The schematic summarizes proposed mechanisms and is not quantitative. Created in BioRender. Károly, S. (2026) https://BioRender.com/yc3edyt (accessed on 1 September 2026).

Figure 4 summarizes the proposed mechanisms.

On mortality, the interaction remains untested. A dedicated query on combined resistance training and protein returned 128 records, none with an estimate meeting a mortality stratum’s contrast definition (Document S4).

What is missing is therefore evidence on survival: trials show effects of the combination on muscle strength and mass, but not on mortality or disability-free survival. The question is also relevant to incretin therapy, which produces rapid weight loss with lean-mass loss in a large population (Section 3.11).

A trial powered on disability-free survival in the populations at highest risk would be needed to answer the question (Document S4).

The evidence supports the claim that the combination changes muscle strength and mass; it does not support a claim about survival, and Section 5.10 applies that test to each recommendation. Document S4 states the inferential chain in full.

### 4.6. Errors Identified During This Review

Two defects in our own pipeline and one in the published literature were identified and are reported here because each could have distorted the results had it gone undetected.

Our pipeline: Two defects were found by checking output against sentences read by hand (Section 2.4). One was a silent extraction failure in which the PDF utility returned success on empty output; the other was a sign-absorbing regular expression in the mean-difference extractor. Both were corrected and the affected analyses re-run; Document S1 logs all eight findings, and Document S4 gives these two in full.

In the literature: One published 2026 review [127] cites a cohort [128] as showing higher sarcopenia incidence at an intake threshold that the source paper uses only as a descriptive cut-point, not as an exposure contrast. We verified this against the original and report it because the misattributed figure has begun to propagate (Document S1, Document S4).

### 4.7. Limitations

Beyond the declared PRISMA deviations (Section 2.8), all mortality estimates in the observational layer are observational. Residual confounding and reverse causation—early illness reducing both activity and intake while raising mortality—are not eliminated by covariate adjustment, and the associations reported should not be read as causal. Three of the six strata fell below the three-study minimum and were not pooled. Of those pooled, plant protein has k = 4, at which between-study variance is poorly estimated, and REML confidence intervals are optimistic. Heterogeneity in the activity stratum is substantial and unexplained, and four of its estimates come from overlapping NHANES cycles (Section 3.2). Exposures were almost all self-reported. Population labels are coarse: “CVD” spans hospitalized heart failure and community-dwelling adults with risk factors, and “CKD” spans stage 3 to dialysis. Regular-expression extraction recovers only what a text states within a bounded window: estimates reported solely in figures, Supplementary Files, or tables whose layout does not survive text extraction were missed.

These limitations determine how the findings should be used. Because the analytic corpus comes from one database and screening outside the validation sample was single-reviewer, relevant studies may have been missed. Pooled estimates summarize the evidence identified rather than all evidence. Because most observational studies were rated at high risk of bias and no protein stratum survived restriction, the protein findings indicate direction rather than magnitude. And because the randomized evidence stops at muscle and function, no recommendation in Section 5 should be read as a claim about survival.

One limitation is specific to the recommendations rather than to the evidence. The windows of Table 6 are assembled from separate studies rather than measured within one cohort; only the hemodialysis window derives both bounds from a common reference, and no window has been validated prospectively. Document S4 states this and the remaining limitations in full.

Three limitations are specific to the randomized layer. It is a layer of pooled estimates rather than of trials, so its unit of analysis is the review. Its quality is bounded by the quality of those reviews, five of which are critically low on AMSTAR 2. Two of those five are critically flawed in the pooling itself and support nothing. The estimates of the other three are reported with their rating stated, and the certainty of the affected bodies of evidence is rated down (Section 3.10). The exercise-and-mortality estimates come from overlapping trial sets and are therefore reported as a table rather than pooled, which means we present consistency rather than a summary estimate. And the randomized trials speak to horizons of months to a few years, whereas the guideline question is about decades. On that mismatch, the observational layer is not replaceable, only checkable.

## 5. Recommendations for Policy Makers

The recommendations are graded by the strength of the evidence assembled here. Where the evidence does not support a change, we say so.

Every number below is bound to the endpoint and the population in which it was measured. No randomized estimate of protein with resistance training reports mortality (Section 4.5), so no recommendation, whatever its grade, should be read as a demonstrated effect on survival or disability-free survival. Recommendations based on observational data inherit that design’s limits. All recommendations are provisional and hypothesis-generating rather than clinical targets; Section 5.10 sets out for each the population, the endpoint and what it does not claim.

**Figure 5:**
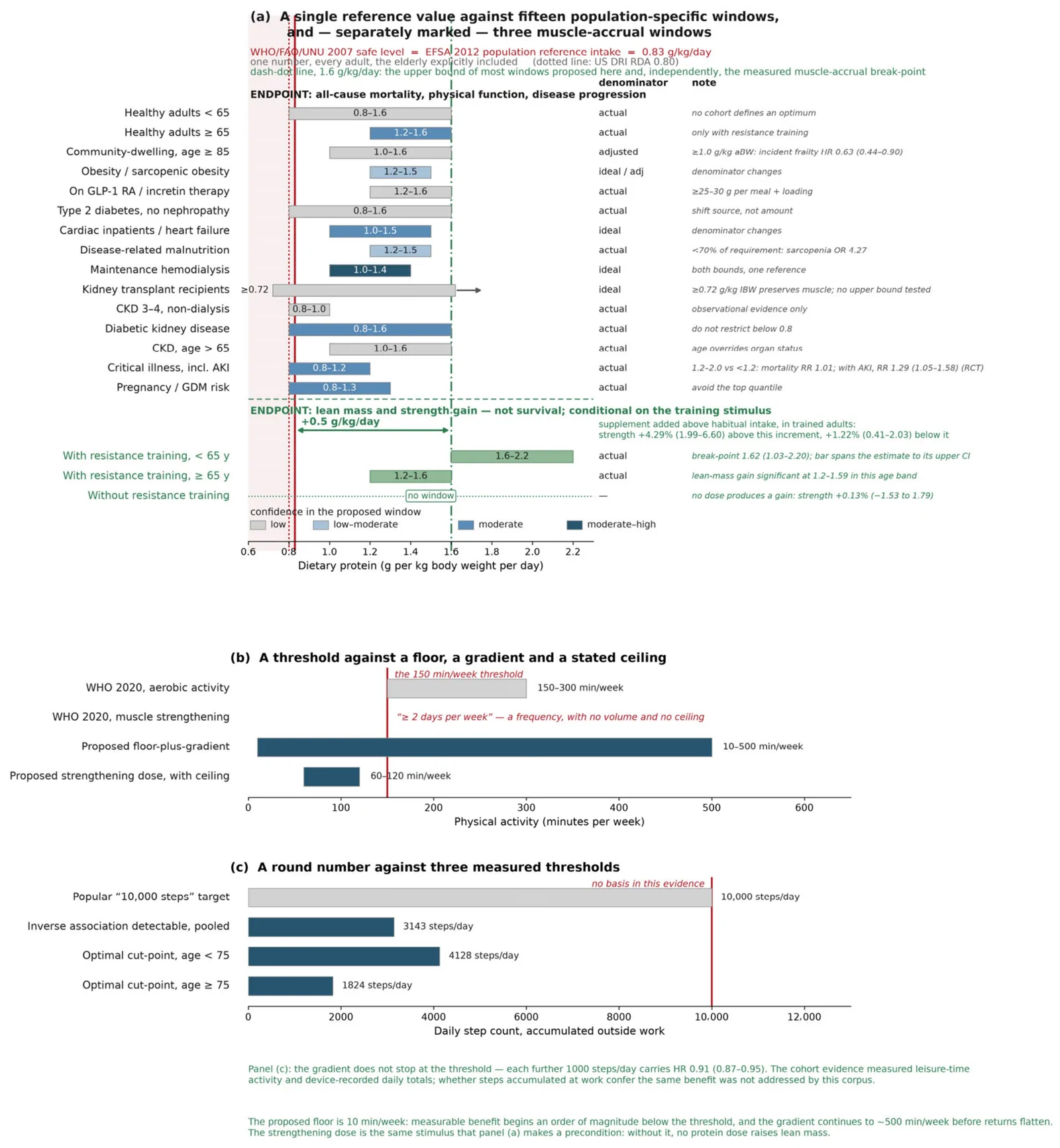
Current international reference values against the provisional, hypothesis-generating windows proposed in this review. (**a**) Dietary protein. The vertical red line is 0.83 g/kg/day, the WHO/FAO/UNU 2007 safe level of intake and the EFSA 2012 population reference intake, which applies to all adults [2,3]. The dotted line is the US RDA of 0.80 g/kg/day. Bars are the windows proposed here, shaded by confidence, with the denominator stated for each row. The green rows below the dashed rule are drawn on a different endpoint: lean-mass and strength gain rather than mortality, function or disease progression. They should not be read off the same axis. The green dash-dot line at 1.6 g/kg/day marks the break-point above which added protein was associated with no further lean-mass gain in meta-regression [12], within the International Society of Sports Nutrition range of 1.4–2.0 g/kg/day [129]. The double-headed arrow marks the +0.5 g/kg/day increment above habitual intake associated with a larger strength effect [83]. The third green row carries no window because, without resistance training, no dose was associated with a strength gain. The transplant row is open-ended because the evidence gives a lower bound (0.72 g/kg ideal body weight per day) and no upper one. Four rows were added at internal revision (Table S15). (**b**) Physical activity, minutes per week. (**c**) Daily step count. Gray bars are the WHO 2020 guideline [1]; dark bars are the proposal.

Figure 5 places what is currently recommended beside what this review proposes, in the units a clinician can act on.

Document S4 explains how Figure 5 should be read, including why the lean-mass windows in panel (a) are drawn on a different endpoint from the other rows.

### 5.1. Consider Replacing the Activity Threshold with a Floor Plus Gradient—Low Certainty, Observational

The 150 min/week figure can function as a pass/fail line and may discourage the least active. In a pooled analysis of 661,137 adults, leisure-time activity below the recommended minimum was associated with HR 0.80 (0.78–0.82) compared with none [10]. The same analysis found that most of the observed reduction was reached at the recommended minimum, so a floor is an entry point rather than a target. Public messaging could combine three elements. A floor: some activity is associated with lower mortality than none, and ten minutes a week is a reasonable start. A gradient: in device-measured data, the association continued to about 300–500 min weekly and then flattened. The pooled analysis places its optimum higher, at three to five times the recommendation, but the two are not in conflict. Self-reported leisure-time activity and device-measured MVPA are not the same exposure and are not interchangeable on a minutes axis. And a step message that does not depend on 10,000 steps a day, a figure not supported as a threshold by the evidence assembled here. In one umbrella review, an inverse association was detectable from about 3143 steps/day, with HR 0.91 per additional 1000 steps [42]. Age-specific cut-points reported for older adults (about 4100 steps/day under 75 and 1800 over 75) may be more appropriate for them [43]. In the largest single cohort, the lowest non-zero activity quartile was not associated with lower mortality (Document S4), which argues against presenting a very small dose as sufficient.

### 5.2. Consider Specifying a Muscle-Strengthening Dose and Its Upper Figure—Observational for Mortality; Randomized for Strength

Current guidance specifies frequency (“2 days per week”) without volume. We suggest 60–120 min per week, stating that no further reduction in mortality was observed above 120 min/week in cohort data. Stating the upper figure may reduce a perceived barrier for people who assume strength training requires a large time commitment.

The recommendation no longer rests only on the two cohorts that generated the ceiling. A pooled analysis of eleven population cohorts covered 80,306 adults, 5763 deaths and 736,463 person-years [17]. Any strength-promoting exercise carried an HR of 0.77 (0.69–0.87) for all-cause mortality and 0.69 (0.56–0.86) for cancer mortality. Meeting the strength guideline alone gave 0.79 (0.66–0.94), meeting the aerobic guideline alone 0.84 (0.78–0.90), and meeting both 0.71 (0.57–0.87). Meeting each guideline was independently associated with lower mortality, and the association for strength activity was of similar size to that for aerobic activity.

Two randomized findings concern outcomes other than mortality. Resistance training raises handgrip strength in older adults with sarcopenia by 2.69 kg (1.78–3.61) in a network meta-analysis of 42 trials summarized in [92]. Adding strength work to an aerobic program improves cardiorespiratory fitness in coronary artery disease (SMD 0.26, 0.02–0.49 overall; SMD 0.36, 0.05–0.68 where aerobic volume was preserved) [91]. Mechanistic trial work also indicates that useful adaptation does not require heavy loads: low external loads taken close to failure produce comparable metabolic and excitatory stimuli [130], which is relevant for older and deconditioned people. The upper figure is cohort-derived, and the randomized evidence concerns strength and fitness, not mortality.

Adherence to muscle-strengthening recommendations is low in some groups [131] (Document S4).

### 5.3. Consider Counting Incidental and Intermittent Activity Toward Guidelines—Observational, Single Device-Measured Cohort

Vigorous intermittent lifestyle physical activity was associated with HR 0.56 (0.39–0.82) at a median of 5.3 bouts/day [44]. In the same analysis, excluding participants with prevalent cardiovascular disease or cancer at baseline attenuated the association and widened its interval, HR 0.68 (0.41–1.11). The source attributes that in part to reverse causation. This finding may be relevant to equity. The classic barriers to structured exercise—cost, facility access, discretionary time, caring responsibilities—fall hardest on the populations with the worst activity profiles. Guidelines could count accumulated incidental bursts without a minimum bout duration. This does not extend to occupational activity: moderate and high occupational activity were associated with higher mortality (aHR 1.24 and 1.71; [132]). Guidelines should distinguish leisure-time from occupational activity.

### 5.4. Consider Activity Prescription Within Chronic-Disease Care—Low Certainty Observational

Lower mortality with higher activity is observed in clinical as well as general populations, although whether the association is as large in clinical cohorts is uncertain once overlapping NHANES cohorts are accounted for (Section 3.2, Table S14). Three changes follow. Activity counseling could be considered a reimbursable clinical service rather than a lifestyle adjunct. Physical activity status could be recorded in electronic health records in the same way as smoking status. And activity referral could serve as a quality indicator for diabetes, post-stroke and cardio-oncology care. None of these measures has itself been tested as an intervention.

### 5.5. Consider Population-Specific Protein Windows in Place of a Single Floor—Provisional and Hypothesis-Generating; Evidence Basis Stated per Population

The evidence in Section 3.6, Section 3.7, Section 3.8 and Section 3.9 points in different directions in different populations. The windows below are built from population-stratified evidence in absolute intake units (Table 3 and Table 7), read study by study. They are not built from the pooled protein strata of Table 2, which do not survive the risk-of-bias restriction (Section 3.5), and they do not depend on the exploratory setting split of Section 3.5 (Document S4).

Table 6 sets out the windows we propose, in absolute units, each with the denominator it should be read against, the type of evidence it rests on and our confidence in it. It is the tabular form of Figure 5a. All windows are provisional and hypothesis-generating rather than clinical targets. Several rest on a single cohort or are extrapolated from another population; those rows are marked in the status column of Table 6, and no window has been validated prospectively.

Randomized evidence bears on three rows. For the healthy-adult upper bound, trials in adults without kidney disease showed no renal harm over their duration [86] (Section 3.9). Guidelines can therefore state that no harm has been shown and that long-term data are lacking.

For the older-adult window, added protein increased strength only with resistance training and had a small effect on muscle mass without it. That is why this row carries a resistance-training prescription that is not a reason to withhold protein (Document S4).

For the kidney rows, the randomized evidence differs by endpoint (Section 3.9). Restriction reduced kidney failure or end-stage kidney disease in some comparisons but showed no difference in mortality, and few trials reported nutritional harm. In diabetic kidney disease, the randomized evidence does not support restriction below 0.8 g/kg/day. Above 65, or with protein-energy wasting, observational data suggest that age should weigh more than organ status [133,134]. Document S4 gives the trials row by row.

Two implementation points follow. Guidelines should state the denominator—ideal or adjusted body weight for obesity, heart failure and dialysis, actual weight elsewhere—to avoid the artifact described in Section 4.2. And in older patients, observational data suggest that the risk of protein-energy wasting may outweigh organ-specific concerns about higher intake.

Table 7 sets out the risks associated with too little and too much intake in each population; their size differs between populations.

Read down the fourth column. In most populations where protein intake is contested, resistance training is the component with randomized evidence on muscle endpoints, and protein appears to act mainly as a condition for its effect. Two settings differ: deficiency, where protein alone was associated with gains in muscle mass, and acute illness, where neither should be escalated. These are comparisons across different studies and endpoints, not estimates from within one trial.

### 5.6. Consider a Protein-And-Resistance-Training Prescription with Incretin Therapy—Provisional; Extrapolated from Other Populations

GLP-1 receptor agonists and dual incretin agonists are prescribed to a rapidly growing population. Across randomized trials, they reduce lean body mass by about 1 kg more than control (Section 3.11). Whether this loss affects function is not known, and no trial of protein or resistance training in this population has been reported. The following recommendations are therefore provisional.

1. A protein target could be issued with the prescription. A total of 1.2–1.6 g/kg/day is distributed as 25–30 g per meal [135]. The upper end applies to anyone with low baseline muscle mass or measured weakness. The dose is extrapolated from the obesity, caloric-restriction and sarcopenia literature [136]; no randomized trial has yet tested protein dose–response during incretin therapy, and the recommendation should say so.
2. The target should be paired with resistance training where feasible. In randomized trials in other populations, added protein increased strength only with resistance training. Its effect on muscle mass without training was small (Section 3.9).
3. Baseline and follow-up measurements of function should be considered, not only of weight. Handgrip strength and a chair-stand test are inexpensive and are part of the definition of sarcopenia. Body weight alone does not distinguish loss of fat from loss of lean tissue.
4. Protein intake should be recorded in grams per kilogram rather than only as a percentage of energy. In the one study that measured both, the same patients were adequate on the relative metric and below requirement on the absolute one.

Evidence behind this recommendation, by endpoint:
**Endpoint****Status in This Corpus**Lean body mass falls on incretin therapyDemonstrated. WMD −1.02 kg (−1.46 to −0.57), 19 randomized trials, Jiao et al. [9]Protein without resistance training changes muscleNo effect shown on strength (+0.13%, −1.53 to 1.79), Tagawa et al. [83]; small effect on muscle mass (SMD 0.116) [93]; not tested during incretin therapyProtein with resistance training changes muscleShown in other populations. Strength +2.01% (1.09–2.93), and lean-mass gain conditional on loading, Tagawa et al. [83], Nunes et al. [112]; not tested during incretin therapyThe same combination preserves function during incretin therapyUnder test, not reported, Alawadhi et al. [105]The same combination extends survival or disability-free survivalUntested, in any population (Section 4.5)

The recommendation rests on the first three rows, two of which come from populations not receiving incretin therapy. It does not claim that the combination preserves function or extends life in this population. Guidance issued now should be explicitly provisional and revisited when trials in this population report (Document S4).

### 5.7. Consider Policies on Processed Meat Products Rather than on Protein Quantity—Observational and Substitution Evidence

This recommendation rests on observational and isocaloric-substitution analyses, not on randomized evidence, and does not carry the certainty of the randomized findings in Section 3.9. The evidence does not support a population target for protein amount. It is consistent with the following:

- Processed meat is more strongly associated than unprocessed red meat with type 2 diabetes: RR 1.41 (1.24–1.60) versus RR 1.11 (1.06–1.16) per 50 g/day [66].
- In substitution analyses, replacing 3% of energy from processed meat with plant protein was associated with a larger reduction in all-cause mortality than replacing unprocessed red meat: HR 0.54 (0.38–0.75) versus HR 0.66 (0.55–0.80) [124].
- Higher plant protein intake is associated with lower all-cause mortality in three meta-analyses (pooled estimate 0.92) and in the largest primary analysis (HR 0.90, 0.86–0.95 per 3% of energy) [50]. Substituting plant for animal protein is associated with HR 0.91 (0.87–0.94) [123] (Section 3.5).

A measure targeted at processing may be less affected by the food-matrix confounding set out in Section 4.4 than one targeted at red meat. Nitrite curing, sodium load and emulsified fat are properties of the product rather than of the meal.

Within the limits of this evidence, policymakers could consider the following:

1. Target ultra-processed meat products (NOVA group 4 processed meats) through fiscal, reformulation and procurement instruments, rather than generic “eat less red meat” advice, which rests on weaker associations.
2. Set institutional procurement standards—schools, hospitals, military, public catering—that displace processed meat and raise the plant:animal protein ratio, consistent with the substitution analyses.
3. Do not frame this as a protein-reduction policy. The evidence supports changing the source, not lowering the amount. A protein-reduction message could be counterproductive in older adults, in heart failure and in dialysis, where low intake is associated with worse outcomes.
4. Hold longevity and healthspan claims for protein supplements to the same substantiation standard as food health claims, requiring human outcome data rather than mechanistic or biomarker inference. This corpus does not support claims that very high protein intakes extend life, and one cohort suggests higher risk at habitual intakes ≥1.8 g/kg/day in adults free of chronic disease at baseline.

### 5.8. Closing the Measurement and Evidence Gaps—Infrastructure

The synthesis was constrained less by the volume of literature than by its comparability. Priorities:

1. Standardize the exposure contrast: Sixteen cohorts out of 7544 records were poolable for physical activity because cohorts report per-SD, per-1000-step, quantile and meets-guideline contrasts interchangeably. Funders could require device-measured activity reported against a common reference metric, and protein reported in g/day alongside g/kg, so the denominator artifact can be measured rather than argued about.
2. Resistance-training dose–response with mortality endpoints—currently two cohorts.
3. Protein × age × renal-function interaction, powered for the interaction rather than the main effect. The direction differences documented in Section 3.6 may reflect an unmodeled interaction.
4. A combined activity-and-protein trial against hard endpoints, in the populations at highest risk, comparing the combination with each component and powered on disability-free survival; Document S4 gives the design parameters.
5. Cooking method, food matrix and co-consumption were found by our search to be almost unstudied in relation to protein outcomes.
6. Adopt image-based, AI-assisted dietary assessment as the exposure instrument for the next generation of protein cohorts—and fund the validation first. Every protein estimate in this review rests on a food-frequency questionnaire or a single recall [137,138]. Exposure measurement is the ROBINS-E domain on which most of these cohorts are rated high risk. Table S12 sets out the measurement error in current instruments and the five requirements such an instrument should meet before it is used for exposure classification. Document S4 gives the supporting evidence [139,140,141,142].
7. A randomized comparison of 0.8 versus 1.2 g/kg/day in CKD stages 3–4 with mortality endpoints, not eGFR slope alone: In existing trials, restriction reduced end-stage kidney disease in some comparisons without a detectable difference in mortality; whether a higher intake carries risk or benefit has not been tested.
8. Protein dose–response during incretin therapy: The targets now being recommended alongside GLP-1 receptor agonists are extrapolated from other studies, as their proponents acknowledge. A dose-ranging trial with function as the endpoint would reduce this reliance on extrapolation.
9. Long-horizon renal safety of high protein intake: The 22 randomized trials that found no renal harm from high-protein diets in people with normal kidneys lasted months [86]. The guideline question is about decades. A registry-linked cohort with repeated measured intake, or a long pragmatic trial, would address a question that is currently unresolved.

### 5.9. The General Adult Population

For a healthy adult under 65 with normal kidney function, the same evidence is consistent with three statements, each subject to the limits set out in Document S4. Some activity is associated with lower mortality than none, the association continues above the guideline minimum, and muscle-strengthening activity of 60–120 min/week is associated with lower mortality (Section 5.1, Section 5.2 and Section 5.3). Protein intake within 0.8–1.6 g/kg of actual body weight per day is consistent with adequacy in the evidence assembled here, and no position within that range has been shown to be better than another. For protein source, the evidence is consistent with eating less processed meat and more plant protein, at observational certainty (Section 5.7). Where a person belongs to a population in Table 6, that row takes precedence. A consultation-level summary and a checklist of features that identify recommendations this evidence does not support are given in Document S4.

### 5.10. What Each Recommendation Does and Does Not Claim

The recommendations above differ in what they are recommendations *about*. Some rest on mortality data, some on function, some on a surrogate, and one on nothing but the design of future studies. Reading them off a single page invites the error this review objects to elsewhere: treating an effect on muscle as an effect on survival (Document S4, red flag 16). Table 8 therefore binds each recommendation to its warrant. The last column states, for each recommendation, the claim a reader is not entitled to make from it.

The column that is the same in every row. No row of Table 8 rests on a randomized mortality estimate. The randomized mortality estimates in this corpus concern exercise programs, protein restriction in chronic kidney disease and protein delivery in critical illness (Table 3 and Table 4). None concerns the combination recommended here. This applies to every recommendation, whatever its grade. The combination of movement and protein has randomized evidence on strength and body composition, observational associations with survival, and no trial evidence on survival.

How to quote a recommendation from this section. A recommendation is quoted correctly when the number, the population and the endpoint travel together. “60–120 min/week of muscle strengthening, associated in cohort data with lower all-cause mortality, with no further reduction in mortality observed above 120 min/week” is a correct use of Section 5.2. “Strength training for 60–120 min a week extends life” is not. Table 8 is shown so that the difference is visible without reading the whole section.

## 6. Conclusions

This PRISMA-guided rapid review supports three conclusions, which differ in the strength of the evidence behind them.

First, the randomized evidence. In older adults, protein supplementation increases strength and appendicular lean mass mainly when it is combined with resistance training, with a larger effect at higher added doses. Without training, protein has at most a small effect on total muscle mass. In people with normal kidney function, trials of higher protein intake showed no renal harm over their duration, although the markers involved are surrogates measured over short follow-up. In cardiac populations, randomized exercise evidence shows reductions in cardiovascular death and hospitalization. No randomized estimate in this review reports all-cause mortality for protein supplementation, for resistance training, or for their combination. The randomized mortality estimates available concern exercise programs, protein restriction in chronic kidney disease and protein delivery in critical illness.

Second, the observational evidence. Meeting physical activity guidelines is associated with lower all-cause mortality across sixteen cohorts (HR 0.76), with substantial heterogeneity that no measured study characteristic explained. The association is present in people with chronic disease, but whether it is as large as in the general population is uncertain. For protein, total intake shows no consistent association with mortality in published syntheses, and higher plant protein intake is associated with lower mortality at very low certainty. When intake is expressed in absolute units, the direction of the association differs between clinical populations. These findings rest largely on self-reported exposures and small pooled strata, and none of the protein strata survives restriction to studies not at high risk of bias. Residual confounding and reverse causation cannot be excluded.

Third, the chain from protein and training to survival is incomplete. The link from protein with resistance training to muscle and function is supported by trials, and the link from sarcopenia to mortality is consistent but observational. The effect of the combination on mortality or disability-free survival has not been tested (Section 3.13). Protein with resistance training is therefore a plausible strategy for preserving muscle mass and function, not a demonstrated longevity intervention.

**Figure 6:**
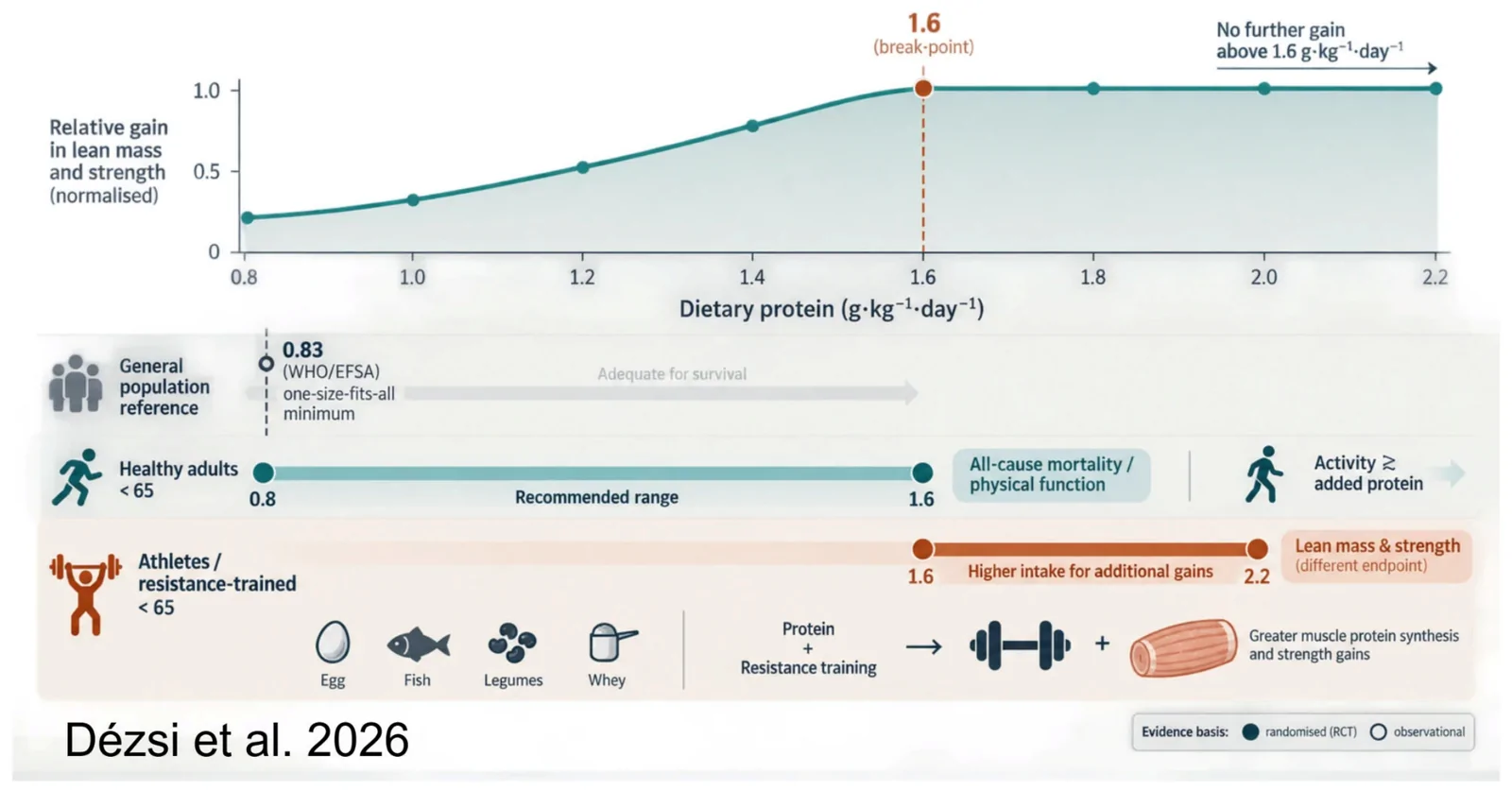
Visual summary of the population-specific dietary-protein reference windows on a single intake axis (g·kg^−1^·day^−1^). For healthy adults < 65 years, the recommended range is 0.8–1.6 g·kg^−1^·day^−1^, judged on all-cause mortality and physical function, where physical activity is at least as important as added protein. For athletes and resistance-trained adults < 65 years, a higher intake of 1.6–2.2 g·kg^−1^·day^−1^ targets lean mass and strength (a different endpoint), and added protein raises strength only in combination with resistance training. The normalized lean-mass and strength dose–response rises to a break-point at ≈ 1.6 g·kg^−1^·day^−1^, above which no further gain was observed. The 0.83 g·kg^−1^·day^−1^ WHO/EFSA value is the one-size-fits-all survival minimum. Filled markers denote randomized-trial (RCT) evidence; open markers denote observational evidence.

The population-specific protein ranges and activity recommendations in Section 5 are provisional and hypothesis-generating rather than clinical targets. Each is labeled with the type of evidence it rests on; several are extrapolated, and none has been validated prospectively. For people receiving GLP-1 receptor agonists, protein intake and resistance training may be a reasonable precaution against lean-mass loss, a suggestion extrapolated from trials in other populations; trials in this population are still pending. Recommendations on processed meat rest on observational and substitution analyses and carry lower certainty than the randomized findings. Trials with functional and hard clinical endpoints are needed to establish whether the combination improves survival. They should compare protein with resistance training against each component, in the populations at highest risk (Document S4) (Figure 6).

## Figures and Tables

**Table 2:** Protein and all-cause mortality by source, each pooled estimate shown beside what remains of it when studies rated high risk of bias are removed. The last two columns are the reason the protein layer of this review is presented as evidence of direction and not of magnitude. No protein stratum retains enough studies to be pooled, and in the total-protein stratum the single surviving cohort points the opposite way to the pool. The plant-protein row was re-estimated at revision after a top-up search added the Women’s Health Initiative cohort; the superseded three-cohort value was 0.69 (0.58–0.82) with *I*^2^ = 0.0%. The activity row is included for contrast, not as a protein result.

Stratum	k	Pooled HR (95% CI)	*I* ^2^	k Not Rated High	What Survives the Restriction
Plant protein, highest vs. lowest	4	0.76 (0.63–0.93)	65.1%	1	SUN cohort 0.65 (0.45–0.93); WHI unrated (Section 3.5). Not poolable
Total protein, highest vs. lowest	5	0.91 (0.64–1.27)	81.0%	1	Rotterdam Study 1.12 (1.01–1.25)—higher mortality, where the pool is null. Not poolable
— generally healthy cohorts (exploratory)	3	0.93 (0.55–1.57)	87.9%	1	Rotterdam carries 26.7% of the weight in the stratum; the setting moderator is not significant, Q_M *p* = 0.79
— chronic-disease cohorts (exploratory)	2	0.83 (0.44–1.56)	76.9%	0	below the three-study minimum (Section 2.6); reported as a subgroup description, not as a pooled result
Animal protein, highest vs. lowest	2	not pooled—0.94 (0.70–1.26); 1.52 (1.13–2.05)	—	1	SUN cohort 0.94 (0.70–1.26). Not poolable
Very high (≥1.8 g/kg/d) vs. adequate	1	singleton—1.39 (1.17–1.65)	—	1	the single cohort itself, 1.39 (1.17–1.65), rated Some concerns after the recalculation of Section 3.10; its contrast is very high versus adequate rather than highest versus lowest, and mortality is a secondary outcome in the source
For contrast: meeting activity guidelines	16	0.76 (0.72–0.79)	64%	9	0.762 (0.716–0.811)—pooled estimate essentially unchanged

**Table 3:** Protein and outcomes by clinical population, absolute intake units only. Rows are single studies with different contrasts, outcomes and designs (observational unless marked RCT); they are descriptive and not directly comparable across rows.

Population	Contrast	Outcome	Estimate	Direction
Maintenance hemodialysis, Wang et al. [52]	<1.0 vs. 1.0–<1.4 g/kg IBW/d	all-cause mortality	HR 1.84 (1.42–2.38)	higher risk at both ends
	≥1.4 vs. 1.0–<1.4 g/kg IBW/d	all-cause mortality	HR 1.49 (1.00–2.22)	
Healthy older adults, Han et al. [53]	<0.8 vs. ≥1.2 g/kg/d	sarcopenia	OR 1.87 (1.46–2.38)	higher intake, better outcome
	0.8–1.2 vs. ≥1.2 g/kg/d	sarcopenia	OR 1.30 (1.11–1.52)	
Age 85+, Mendonça et al. [54]	≥1.0 g/kg aBW/d	incident frailty, age 85–90	HR 0.63 (0.44–0.90)	higher intake, better outcome
Obesity, Baabbad et al. [18]	total protein, per 1 g/kg BW/d (continuous)	prevalent sarcopenic obesity at the follow-up examination	OR 0.14 (0.08–0.20); energy intake omitted from this model for collinearity, unlike the animal (0.58, 0.53–0.62) and plant (0.46, 0.41–0.52) models	higher intake, better outcome—but see Section 4.2: body size enters both the outcome definition and the denominator of the exposure
Hospitalized cardiac/HF, Katano et al. [55]	lowest vs. upper quartiles (g/kg IBW/d)	death + HF readmission	HR 2.03 (1.08–3.82)	lower intake, higher risk
Community adults free of chronic disease, Huang et al. [56]	≥1.8 vs. <1.8 g/kg BW/d	all-cause mortality	HR 1.39 (1.17–1.65)	higher intake, higher risk (single cohort)
CKD non-dialysis, Beberashvili et al. [57]	<1.0 vs. ≥1.0 g/kg/d	kidney failure/dialysis/death	HR 0.77 (0.62–0.97)	lower intake, lower risk
CKD + T2DM, Liu et al. [58]	>1.0 g/kg/d (urea/creatinine-estimated; PSM subgroup model, reference category not stated)	composite renal and cardiovascular endpoint	HR 3.53 (1.54–8.09)	higher intake, higher risk
CKD, age > 65, Watanabe et al. [59]	moderate vs. low intake	all-cause mortality	HR 0.14 (0.02–0.69)	higher intake, lower risk
Diabetic kidney disease, Jiang et al. [60]	LPD 0.6–0.8 g/kg/d vs. usual	kidney failure	RR 1.16 (0.38–3.59)	no difference (RCT)
Critically ill (randomized), Summers et al. [61]	enteral 1.2–2.0 vs. <1.2 g/kg/d	all-cause mortality	RR 1.01 (0.92–1.12), *I*^2^ = 0%, from the 12 trials reporting mortality of the 14 included (*n* = 6553 overall)	no difference (RCT)
Critically ill with acute kidney injury, Summers et al. [61]	same contrast, subgroup of the same review	all-cause mortality	RR 1.29 (1.05–1.58), *p* = 0.015, *I*^2^ = 0%, 3 trials, *n* = 755; the source rates the credibility of the effect modification as moderate on ICEMAN and notes that the subgroup definition varied between trials	higher intake, higher mortality (randomized subgroup; hypothesis-generating)
Disease-related malnutrition, Pérez-López et al. [62]	<70% vs. ≥70% of requirement (target 1.5 g/kg/d)	sarcopenia	OR 4.27 (1.30–14.03)	lower intake, higher risk
Kidney transplant recipients, Kosoku et al. [63]	protein intake, continuous (Maroni)	1-year change in muscle mass	positive, *p* = 0.015; break-even 0.72 g/kg IBW/d	higher intake, better outcome
Maintenance hemodialysis, 10-year, Kang et al. [64]	energy < 25 kcal/kg/d	all-cause mortality	HR 1.86 (1.02–3.40)	lower energy intake, higher risk

**Table 4:** Randomized evidence layer: 16 trials and pooled analyses, 32 estimates, reported by domain and not pooled (Section 2.5). Within each domain, individual trials, conventional pairwise meta-analyses, individual-participant-data (IPD) meta-analyses and network meta-analyses are listed separately. These designs estimate different quantities, and their results should not be compared as if interchangeable. Full version with per-row notes: Supplementary Table S11.

Domain	Design	Population	Contrast	Estimate (95% CI)	Source
PA → all-cause mortality	RCT (5 y, *n* = 1567)	community-dwelling adults aged 70–77, generally healthy	HIIT vs. control	HR 0.63 (0.33 to 1.2)	Stensvold et al. [77]
PA → all-cause mortality	Cochrane MA (25 trials)	coronary heart disease	exercise-based cardiac rehabilitation vs. usual care, 6–12 mo	RR 0.87 (0.73 to 1.04)	Dibben et al. [78]
PA → all-cause mortality	Cochrane MA (15 trials)	coronary heart disease	same, medium-term follow-up	RR 0.9 (0.8 to 1.02)	Dibben et al. [78]
PA → all-cause mortality	Cochrane MA (long-term)	coronary heart disease	same, long-term follow-up	RR 0.91 (0.75 to 1.1)	Dibben et al. [78]
PA → all-cause mortality	Cochrane MA (34 trials)	heart failure	exercise-based cardiac rehabilitation vs. usual care, ≤12 mo	RR 0.93 (0.71 to 1.21)	Molloy et al. [79]
PA → all-cause mortality	IPD meta-analysis	heart failure	exercise cardiac rehabilitation vs. control	HR 0.83 (0.67 to 1.04)	Taylor et al. [80]
PA → all-cause mortality	network MA (*n* = 19,411)	coronary heart disease	center-based cardiac rehabilitation vs. usual care	RR 0.76 (0.64 to 0.9)	Xia et al. [81]
Protein → muscle (with/without training)	MA of RCTs	older adults	protein vs. lower-protein control—total lean body mass	WMD (kg) 0.34 (−0.21 to 0.89)	Kirwan et al. [82]
Protein → muscle (with/without training)	MA of RCTs	older adults	protein + resistance exercise—appendicular lean mass	WMD (kg) 0.54 (0.03 to 1.05)	Kirwan et al. [82]
Protein → muscle (with/without training)	MA of RCTs	older adults	protein + resistance exercise—handgrip strength	WMD (kg) 1.71 (0.12 to 3.3)	Kirwan et al. [82]
Protein → muscle (with/without training)	MA of RCTs	sarcopenic/frail only	protein ± exercise—appendicular lean mass	WMD (kg) 0.88 (0.51 to 1.25)	Kirwan et al. [82]
Protein → muscle (with/without training)	dose–response MA (69 articles)	adults	added protein WITH resistance training—muscle strength	MD (%) 2.01 (1.09 to 2.93)	Tagawa et al. [83]
Protein → muscle (with/without training)	dose–response MA (69 articles)	adults	added protein WITHOUT resistance training—muscle strength	MD (%) 0.13 (−1.53 to 1.79)	Tagawa et al. [83]
Protein → muscle (with/without training)	dose–response MA	adults	added protein <0.5 g/kg/day + resistance training—strength	MD (%) 1.22 (0.41 to 2.03)	Tagawa et al. [83]
Protein → muscle (with/without training)	dose–response MA	adults	added protein >0.5 g/kg/day + resistance training—strength	MD (%) 4.29 (1.99 to 6.6)	Tagawa et al. [83]
Protein → muscle (with/without training)	MA of RCTs	older adults with sarcopenia	protein supplementation—appendicular skeletal muscle mass	SMD 0.41 (0.24 to 0.58)	Kwon et al. [84]
Protein → muscle (with/without training)	MA of RCTs	older adults with sarcopenia	protein supplementation—handgrip strength	SMD 0.37 (−0.32 to 1.07)	Kwon et al. [84]
Protein → muscle (with/without training)	MA of RCTs	older adults, low physical activity	protein supplementation alone—total lean body mass	MD (kg) −0.01 (−3.23 to 3.2)	Zhang et al. [85]
High protein → kidney (no CKD)	MA of 22 RCTs (RoB 2 + GRADE)	adults without chronic kidney disease	high-protein vs. control diet—eGFR	SMD 0.39 (0.09 to 0.69)	Teixeira et al. [86]
High protein → kidney (no CKD)	MA of 19 RCTs	adults without chronic kidney disease	high-protein vs. control diet—serum creatinine	SMD 0.08 (−0.09 to 0.25)	Teixeira et al. [86]
Protein restriction in CKD	MA of 19 RCTs (*n* = 2492)	chronic kidney disease	low-protein vs. control—kidney failure	OR 0.59 (0.41 to 0.85)	Yan et al. [87]
Protein restriction in CKD	MA of 19 RCTs	chronic kidney disease	low-protein vs. control—end-stage renal disease	OR 0.64 (0.43 to 0.96)	Yan et al. [87]
Protein restriction in CKD	MA of 19 RCTs	chronic kidney disease	low-protein vs. control—all-cause death	OR 1.17 (0.67 to 2.06)	Yan et al. [87]
Protein restriction in CKD	Cochrane MA (5 trials, *n* = 1680)	non-diabetic CKD	low-protein vs. normal-protein—death	RR 0.77 (0.51 to 1.18)	Hahn et al. [88]
Protein restriction in CKD	Cochrane MA (6 trials, *n* = 1814)	non-diabetic CKD	low-protein vs. normal-protein—end-stage kidney disease	RR 1.05 (0.73 to 1.53)	Hahn et al. [88]
Protein restriction in CKD	Cochrane MA (10 trials, *n* = 1010)	non-diabetic CKD	very-low-protein vs. low-protein—end-stage kidney disease	RR 0.65 (0.49 to 0.85)	Hahn et al. [88]
Protein restriction in CKD	Cochrane MA (6 trials, *n* = 681)	non-diabetic CKD	very-low-protein vs. low-protein—death	RR 1.26 (0.62 to 2.54)	Hahn et al. [88]
Protein restriction in CKD	RCT (median 74 months)	advanced CKD	supplemented very-low-protein vs. low-protein—mortality	HR 0.95 (0.62 to 1.45)	Bellizzi et al. [89]
Protein restriction in CKD	MA of RCTs	diabetic nephropathy	low-protein diet—proteinuria	SMD 0.69 (0.22 to 1.16)	Li et al. [90]
Protein restriction in CKD	MA of RCTs	diabetic nephropathy	low-protein diet—glycated hemoglobin	SMD 0.17 (−0.18 to 0.51)	Li et al. [90]
Training composition and dose	MA of 23 RCTs (*n* = 916)	coronary artery disease	strength + aerobic vs. aerobic alone—cardiorespiratory fitness	SMD 0.26 (0.02 to 0.49)	Terada et al. [91]
Training composition and dose	network MA of 42 RCTs (*n* = 3728), as summarized in [92]	older adults with sarcopenia	resistance training vs. control—handgrip strength	MD (kg) 2.69 (1.78 to 3.61)	Shen et al. [92]

**Table 5:** Certainty of the evidence (abbreviated GRADE summary; full version, Supplementary Table S10).

Outcome	Effect	Certainty	Principal Reason
Meeting activity guidelines → all-cause mortality (observational)	HR 0.76 (0.72–0.79), k = 16	Low	starts Low as observational and is not rated up: the dose–response gradient comes from other analyses (Section 3.3) rather than from this contrast; exposure was self-reported in fifteen of the sixteen contributing cohorts; *I*^2^ = 64% (95% CI 29–89%) is unexplained (Table S14); seven of the sixteen cohorts contributing to this estimate are at high risk of bias; and four estimates share NHANES cycles
Structured exercise → all-cause mortality (randomized)	RR 0.87–0.93 in two pairwise syntheses; HR 0.83 (0.67–1.04) in an individual-participant meta-analysis; RR 0.76 (0.64–0.90) in a network meta-analysis restricted to center-based cardiac rehabilitation	Low	rated down for imprecision—three of four intervals cross the null, and the one that does not is restricted to center-based cardiac rehabilitation—and down again because the two estimates furthest from the null come from reviews rated Critically low on AMSTAR 2
Exercise → cardiovascular mortality (randomized)	RR 0.58 (0.43–0.78)	Moderate	rated down for imprecision: eight trials, wide interval
Exercise → all-cause hospitalization in heart failure (randomized)	RR 0.69 (0.56–0.86)	High	precise, consistent across Cochrane updates, directly measured
Total protein → all-cause mortality (observational)	null across three syntheses; our pool 0.91 (0.64–1.27), k = 5	Very low	inconsistency (*I*^2^ = 81%, prediction interval 0.43–1.89) plus indirectness of the exposure metric; an exploratory split by setting shows no difference (Q_M *p* = 0.79), and the only cohort not rated high risk reports 1.12 (1.01–1.25) (Table 2)
Plant protein → all-cause mortality (observational)	HR 0.76 (0.63–0.93), k = 4	Very low	downgraded from Low at revision: k = 4, all FFQ, *I*^2^ = 65%, prediction interval crosses 1, diet-quality confounding unresolved, and the stratum falls to one appraised cohort when studies rated high risk are removed (Table 2). The direction is consistent; the magnitude is not defensible
Protein alone → lean mass in older adults (randomized)	WMD 0.34 kg (−0.21 to 0.89)	Moderate	rated down for imprecision; consistent null
Protein with resistance training → strength and lean mass (randomized)	ALM +0.54 kg (0.03–1.05); strength +2.01% (1.09–2.93)	Moderate	rated down for *I*^2^ of 89–95%; dose–response monotonic
High protein → kidney function without CKD (randomized)	eGFR SMD 0.39 (0.09–0.69); creatinine SMD 0.08 (−0.09 to 0.25)	Moderate	rated down for indirectness—short trials, filtration, not outcomes
Protein restriction in CKD → mortality (randomized)	RR 0.77 (0.51–1.18); RR 1.26 (0.62–2.54); OR 1.17 (0.67–2.06); HR 0.95 (0.62–1.45)	Low	rated down for imprecision (four estimates, no signal) and down again because one contributing review is rated Critically low on AMSTAR 2 and the contributing trial is at high risk of bias on RoB 2
Protein restriction in CKD → end-stage kidney disease (randomized)	very-low vs. low RR 0.65 (0.49–0.85)	Moderate	rated down for risk of bias—unblinded, adherence incomplete
Incretin therapy → lean body mass (randomized)	WMD −1.02 kg (−1.46 to −0.57), 19 trials	Moderate	rated down for indirectness—the outcome is tissue mass, not function, and no trial measured strength
Protein + resistance training during weight loss → fat-free mass (randomized, extrapolated to incretin therapy)	MD +0.30 kg (0.09–0.52)	Low	rated down twice: imprecision and indirectness—no trial has tested this during incretin therapy

**Table 6:** Proposed provisional reference windows in absolute units, with the evidence basis and confidence for each. Evidence basis: randomized = randomized trials on the stated endpoint; observational = cohort or cross-sectional associations; surrogate = biomarker or tissue endpoint rather than a clinical outcome; extrapolated = derived from another population. The windows are provisional, hypothesis-generating reference points intended to frame research and guideline discussion; they are not validated clinical targets. Rows marked hypothesis-generating rest on a single cohort or on extrapolation from another population.

Population	Proposed Window	Denominator	Evidence Basis	Confidence and Status
Healthy adults < 65	0.8–1.6 g/kg/d	actual BW	observational (mortality); randomized surrogate (renal markers)	low; provisional
Healthy adults ≥ 65	1.2–1.6 g/kg/d	actual BW	randomized (muscle mass, strength, with training); observational (sarcopenia)	moderate (randomized function endpoints); provisional
Obesity/sarcopenic obesity	1.2–1.5 g/kg/d	ideal or adjusted BW	observational (sarcopenic obesity); randomized surrogate (fat-free mass during weight loss)	low; provisional
Type 2 diabetes, no nephropathy	general adult range; shift source	actual BW	observational (incidence; source)	low; provisional
Pregnancy/gestational diabetes mellitus (GDM) risk	avoid the top quantile of total and animal protein	actual BW	observational (GDM incidence)	low (observational only); provisional
Cardiac inpatients/heart failure	≥1.0, target 1.0–1.5 g/kg IBW/d	ideal BW	observational (death and readmission)	low (single cohort); provisional—hypothesis-generating
Community adults free of chronic disease, upper bound	avoid habitual ≥1.8 g/kg/d	actual BW	observational (mortality), single cohort	low—one disputed cohort; provisional—hypothesis-generating
CKD 3–4, non-dialysis	0.8–1.0 g/kg/d, individualized	actual BW	observational (kidney failure, dialysis or death)	low—observational only; provisional
Diabetic kidney disease	do not restrict below 0.8 g/kg/d	actual BW	randomized (kidney failure)	low (randomized but imprecise: RR 1.16, 0.38–3.59); provisional
Adults on GLP-1 RA or dual incretin therapy	1.2–1.6 g/kg/d, ≥25–30 g per meal, with resistance training	actual BW (adjusted if BMI > 30)	extrapolated (dose); randomized surrogate (lean-mass loss on therapy)	low for the dose; low for the co-prescription, which is extrapolated from randomized trials in other populations (Table 5); provisional—hypothesis-generating (extrapolated)
Maintenance hemodialysis	1.0–1.4 g/kg IBW/d	ideal BW	observational (mortality), single cohort; provisional	low (single cohort); provisional—hypothesis-generating
CKD, age > 65	do not restrict	actual BW	observational (mortality)	low; provisional

**Table 7:** Observed risk at lower and at higher intake, by population, with the randomized muscle-endpoint evidence alongside. Entries in the first two columns are observational associations unless a row states otherwise, and “direction indicated” names the direction those associations point in, not a demonstrated effect. Estimates are those of Section 3.5, Section 3.6, Section 3.7, Section 3.13 and Section 4.5; the units and denominators are those of Table 6.

Population	Observed Risk at Lower Intake	Observed Risk at Higher Intake	Randomized Evidence on Muscle Endpoints	Direction Indicated
Healthy adults < 65	no deficiency signal within the adequacy range	no benefit shown above adequacy; no renal harm over trial horizons (eGFR SMD 0.39, creatinine unchanged)	grip +2.69 kg; strength guideline alone HR 0.79	the evidence here favors activity over added protein
Healthy adults ≥ 65	sarcopenia OR 1.87 (1.46–2.38) at <0.8 vs. ≥1.2 g/kg/d	none demonstrated	mass SMD 0.22 and grip MD 4.19 kg with training [113]; mass SMD 0.116 without [93]	raised to 1.2–1.6, with loading
Age 85+	incident frailty (85–90 y) HR 0.63 (0.44–0.90) at ≥1.0 g/kg aBW/d	not measured	not measured in this age band	raise
Obesity/sarcopenic obesity	prevalent sarcopenic obesity OR 0.14 (0.08–0.20) per 1 g/kg/d of total protein, an increment wider than the intake distribution it was estimated in, not adjusted for energy, and exposed to the denominator problem of Section 4.2 [18]; sarcopenic obesity → incident CKD HR 1.77 [118]	over-prescription if actual rather than ideal weight is used	the only intervention in this review with randomized evidence on lean mass during weight loss	higher intake, calculated on ideal or adjusted weight
Type 2 diabetes, no nephropathy	no amount signal	source association: processed meat RR 1.41, animal protein RR 1.13–1.19	not tested in this population; relevant to the sarcopenic-obesity pathway	change source, not amount
CKD 3–4, non-dialysis	protein-energy wasting; osteosarcopenia HR 3.28 (1.52–7.08) for death/KRT/MACE	<1.0 vs. ≥1.0 g/kg/d, HR 0.77 (0.62–0.97) for kidney failure, dialysis or death	rising grip strength HR 0.93 (0.89–0.98) [117], the only measure whose improvement tracked lower risk, and a functional rather than a body-composition measure	individualize 0.8–1.0 and monitor muscle, not weight
CKD, age > 65	moderate vs. low intake, all-cause mortality HR 0.14 (0.02–0.69)	not demonstrated at moderate intake	PA associated with lower mortality in end-stage disease	do not restrict
Diabetic kidney disease	restriction below 0.8 g/kg/d risks wasting	low-protein diet vs. usual, kidney failure RR 1.16 (0.38–3.59)—no benefit, randomized	not tested	do not restrict below 0.8
Maintenance hemodialysis	<1.0 vs. 1.0–<1.4 g/kg IBW/d, HR 1.84 (1.42–2.38)	≥1.4 vs. 1.0–<1.4, HR 1.49 (1.00–2.22)	not tested	window 1.0–1.4 IBW; higher mortality at both ends (single cohort)
Kidney transplant recipients	below ≈0.72 g/kg IBW/d, muscle recovery incomplete in year 1	not demonstrated	not tested	release the restriction after transplant
Cardiac inpatients/heart failure	lowest vs. upper quartiles, death + HF readmission HR 2.03 (1.08–3.82)	not demonstrated	not tested	≥1.0, target 1.0–1.5 g/kg IBW
On incretin therapy	lean-mass loss 1.02 kg (0.57–1.46) more than control across 19 trials	not demonstrated	in randomized trials, protein raised strength only when combined with resistance training	1.2–1.6 g/kg/d with resistance training
Disease-related malnutrition	<70% of requirement: sarcopenia OR 4.27 (1.30–14.03), severe malnutrition OR 5.17 (1.63–16.42) [62]	not demonstrated	often not deliverable—protein alone still raises mass	raise, even where loading is impossible
Critical illness, incl. acute kidney injury	not demonstrated	1.2–2.0 vs. <1.2 g/kg/d: mortality RR 1.01 (0.92–1.12); with AKI, 1.29 (1.05–1.58)	contraindicated in the acute phase	do not raise
Community adults free of chronic disease	not demonstrated	habitual ≥1.8 vs. <1.8 g/kg/d, all-cause mortality HR 1.39 (1.17–1.65), one disputed cohort	not tested	avoid habitual ≥1.8
Pregnancy/GDM risk	not demonstrated	top quantile of total and animal protein; Western pattern (dairy, baked/fried food, white meat) aOR 4.40 (1.58–12.22)	not tested	the signal concerns dietary pattern rather than protein quantity

**Table 8:** Recommendation-level warrant. For each recommendation: the number recommended, the population and design the number was measured in, the endpoint it was measured on, and the claim the recommendation does not license. No row of this table rests on a randomized mortality estimate: the combination of protein and resistance training has randomized evidence on strength and body composition, observational associations with survival, and no trial evidence on survival.

Rec.	What Is Recommended	Measured in	Endpoint the Number Was Measured on	What This Does Not Claim
5.1	Floor at 10 min/week; gradient to ~300–500 min/week; step floor ~3143/day; step message independent of the 10,000-step figure	661,137 adults, self-reported leisure-time activity, Arem et al. [10]; device-measured step cohorts, Rodríguez-Gutiérrez et al. [42], Shimoda et al. [43]; accelerometer analysis for the 10 min/week figure, Xu et al. [15]	All-cause mortality, observational. HR 0.80 (0.78–0.82) below the minimum versus none; HR 0.91 (0.87–0.95) per additional 1000 steps; a detectable association from 10 min/week of device-measured MVPA	That moving the floor has been shown *in a trial* to reduce mortality. No trial has randomized a floor. Self-reported and device-measured exposures are not interchangeable on a minutes axis, and occupational steps were not tested (Section 5.3)
5.2	60–120 min/week of muscle strengthening, with the ceiling published	80,306 adults, 5763 deaths, Stamatakis et al. [17]; ceiling from two cohorts, Zhang et al. [45]; floor from a network meta-analysis of 42 randomized trials, as summarized by Shen et al. [92]	Mortality for the dose (observational): HR 0.79 (0.66–0.94) for the strength guideline alone; HR 0.87 (0.81–0.95) at 90–119 min/week. Strength for the floor (randomized): +2.69 kg handgrip (1.78–3.61)	That 60–120 min/week has been shown to extend life. The upper figure is cohort-derived; the randomized layer under this recommendation reaches strength and fitness, not survival
5.3	Count incidental and intermittent activity, no minimum bout duration	Device-measured non-exercisers, Koemel et al. [44]	All-cause mortality, observational. HR 0.56 (0.39–0.82) at a median 5.3 bouts/day. The source also reports that the association attenuates to HR 0.68 (0.41–1.11) when participants with prevalent cardiovascular disease or cancer are excluded	That bouts are causally equivalent to structured exercise, or that *occupational* activity qualifies—moderate and high occupational activity ran the other way (aHR 1.24 and 1.71), Quinn et al. [132]
5.4	Consider activity prescription within chronic-disease care	16 cohorts; 5 general-population, 11 clinical	All-cause mortality, observational. General 0.73 (0.68–0.78); clinical 0.77 (0.73–0.82); Q_M = 1.71, *p* = 0.19 as reported; *p* = 0.013–0.21 depending on the handling of overlapping NHANES cohorts (Table S14)	That the association is the same size in the two settings. A non-significant moderator test does not show equivalence, and the result depends on how overlapping cohorts are handled. Nor has reimbursement itself been tested as an intervention
5.5	Population-specific windows in place of one floor, each with its denominator (Table 6)	Twelve populations, mostly observational; three rows carry randomized evidence. The pooled protein estimates of Table 2 do not survive restriction to studies not rated high risk, and the setting moderator of Section 3.5 rests on one cohort—neither is load-bearing for this recommendation (Section 5.5)	Mixed, and stated per row. Mortality (observational) for most rows; *function* for the ≥65 row; *surrogate* (eGFR SMD 0.39, 0.09–0.69, with creatinine unchanged) for the healthy upper bound, Teixeira et al. [86]; randomized for the diabetic-kidney-disease non-restriction row	That any window has been validated against a mortality endpoint in a trial. None has. The healthy-adult upper bound rests on a renal surrogate over trial horizons, so the appropriate statement is that no harm was shown over trial durations, not that long-term intake is safe
5.6	1.2–1.6 g/kg/day, 25–30 g per meal, paired with resistance training, on incretin therapy	Lean-mass loss measured in 19 randomized incretin trials, Jiao et al. [9]; the dose extrapolated from obesity, caloric-restriction and sarcopenia literature; the pairing from randomized protein × training trials, Tagawa et al. [83], Nunes et al. [112]	Lean body mass and strength, randomized. WMD −1.02 kg (−1.46 to −0.57) lean mass on incretins; strength +2.01% (1.09–2.93) with training versus +0.13% (−1.53 to 1.79) without	That the combination preserves function *during incretin therapy*—that trial is underway and has not reported, Alawadhi et al. [105]—or that it extends survival. No incretin-specific protein dose–response trial has reported; the recommendation is provisional
5.7	Consider policies on ultra-processed meat products rather than on protein quantity	Pooled cohorts, Fan et al. [66], Budhathoki et al. [124]	Type 2 diabetes incidence and all-cause mortality, observational. Processed meat RR 1.41 (1.24–1.60) versus red meat 1.11 (1.06–1.16) per 50 g/day; substitution HR 0.54 (0.38–0.75) versus 0.66 (0.55–0.80)	That processing has been randomized. It has not, in any study here. The recommendation carries observational certainty only; targeting processing may be less affected by food-matrix confounding, but the design is no stronger than that of the other observational rows
5.8	Measurement and trial-design infrastructure	—	No clinical endpoint. This recommendation is about how future evidence should be generated	Nothing about outcomes. It is included so that the gaps named in the rows above become answerable

## Data Availability

The original contributions presented in this study are included in the article and Supplementary Material. The complete search log is machine-readable and permits exact reproduction of the identification step; the extraction and analysis code is provided in full. No new primary data were created. Further inquiries can be directed to the corresponding authors.

## Supplementary Materials

The following supporting information can be downloaded at: https://www.mdpi.com/article/10.3390/nu18193259/s1. References [143,144,145,146] are cited in the Supplementary Materials. Table S1: PRISMA-S search strategy—the 58 query strings of the original PubMed search, verbatim, with execution timestamps, true match counts and retrieved counts, grouped by tranche and with every capped query flagged; Table S1b: the remaining 153 logged executions that complete the PRISMA-S record—the nine date-floor-removed PubMed re-runs and every execution against Europe PMC (67), Semantic Scholar (67) and ClinicalTrials.gov (10), with the same fields; Table S2: all 8304 extracted effect estimates with provenance context and source PMID, in the two measure families kept separate throughout (6005 ratio; 2299 mean-difference); Table S3: the curated analysis set—every estimate admitted to a pooled stratum, with its contrast, population and source; the rejected candidates and the reason for each rejection are in the extraction code supplied as File S1; Table S4: population-stratified evidence table in absolute intake units; Table S5: comparator table of published meta-analyses; Table S6: reference number to PMID/DOI mapping for all cited studies; Table S7: ROBINS-E domain grid for the twenty-six appraised observational studies (the Women’s Health Initiative, admitted at revision, is unrated—Section 3.5, Document S1); Table S7b: Newcastle–Ottawa star grid for the same twenty-six, with the scoring conventions and the cross-tabulation against ROBINS-E; Table S8: RoB 2 grid for the randomized trials; Table S9: AMSTAR 2 critical-domain grid for the fifteen included reviews; Table S10: full GRADE summary of findings; Table S11: the complete randomized evidence table (32 estimates, 16 sources); Table S12: measurement error in dietary exposure instruments and in automated image-based assessment, with the five requirements of Section 5.8 item 6 in full; Table S13: conduct of this review mapped item by item against the Cochrane Rapid Reviews Methods Group recommendations, including the four on which it does not meet them; Table S14: heterogeneity and sensitivity analyses of the physical activity stratum—overall model with confidence intervals for τ^2^ and *I*^2^, subgroups, exploratory meta-regression, influence diagnostics, small-study effects and parent-cohort (NHANES) overlap; Table S15: analyses planned at first draft distinguished from those added at internal revision and in response to peer review; File S1: the analysis code and the machine-readable search record—the extraction pipeline (Python, both measure families), the conference-abstract exclusion script, the curated analysis set, the population and comparator tables, the pooling and moderator scripts and the heterogeneity script (R, metafor 5.0.1), the forest and PRISMA flow renderers, the reference builder, the six-dimension reference verifier and the in-text estimate verifier, the search log itself and the query scripts as executed; Document S1: log of citation and pipeline errors identified during this review; Document S2: retrospective protocol in PROSPERO field format, with PRISMA 2020 and PRISMA-S checklists; Document S3: methods in full—the ten search tranches one by one, the declared PRISMA deviations at length, and the risk-of-bias domain definitions and instrument-selection reasoning. Document S4: extended methods and results—the passages moved out of the main text to keep it within the length a review is normally granted. They are reproduced unshortened under the section each came from, and share the main manuscript’s reference numbering.

## Abbreviations

The following abbreviations are used in this manuscript:

aBW	adjusted body weight
BMI	body mass index
BW	body weight
CI	confidence interval
CKD	chronic kidney disease
CVD	cardiovascular disease
eGFR	estimated glomerular filtration rate
GDM	gestational diabetes mellitus
HF	heart failure
HR	hazard ratio
IBW	ideal body weight
IPD	individual-participant data
IRR	incidence rate ratio
LPD	low-protein diet
MACE	major adverse cardiovascular events
MD	mean difference
MET	metabolic equivalent of task
MVPA	moderate-to-vigorous physical activity
NOS	Newcastle–Ottawa Scale
OR	odds ratio
PA	physical activity
RCT	randomized controlled trial
REML	restricted maximum likelihood
RR	risk ratio
SMD	standardized mean difference
T2DM	type 2 diabetes mellitus
WMD	weighted mean difference
