# Supplementary material for: Protein Intake and Resistance Training Across Clinical Populations: A PRISMA-Guided Rapid Review of Observational and Randomized Evidence
Source: Nutrients. 2026 Oct 2;18(19):3259. doi: 10.3390/nu18193259 (PMC13666671; doi:10.3390/nu18193259)
Supplement: Supplementary file 1 [file nutrients-18-03259-s001.zip › Supplementary_Material.pdf]

Table of Contents

Supplementary Material 2

Table S1. PRISMA-S search strategy — the 58 query strings of the original PubMed search 2

Table S1b. The remaining 153 logged executions that complete the PRISMA-S record — Europe PMC, Semantic Scholar and ClinicalTrials.gov 35

Table S2. All extracted effect estimates 110

Table S3. Curated analysis set 110

Table S4. Population-stratified evidence, absolute intake units 116

Table S5. Comparator table of published meta-analyses 126

Table S6. Reference number → PMID mapping 129

Table S7. ROBINS-E — observational exposure studies entering a synthesis 152

Table S7b. Newcastle–Ottawa Scale (cohort form) — the same 26 observational studies 179

Table S8. RoB 2 — randomized trials 238

Table S9. AMSTAR 2 — included systematic reviews and meta-analyses 241

Table S10. GRADE summary of findings 249

Table S11. Randomized evidence table (full) 256

Table S12. Measurement error in dietary exposure instruments, and what image-based assessment does and does not fix 264

Table S13. Conduct against the Cochrane Rapid Reviews Methods Group recommendations 266

Table S14. Heterogeneity and sensitivity analyses of the physical activity stratum (k = 16) 272

Table S15. Analyses planned at first draft, added at internal revision, and added in response to peer review 276

Document S1. Log of citation and pipeline errors identified during this review 281

Document S2. Protocol — PROSPERO registration form (all fields) 286

PRISMA-S checklist (search reporting) 301

Document S3. Methods in full — search architecture, declared deviations and appraisal 304

Document S4. Extended methods and results 309

Supplementary Material

Protein Intake and Resistance Training Across Clinical Populations: A PRISMA-Guided Rapid Review of Observational and Randomized Evidence

Dézsi C., Gulyás-Oldal V., Paschali E., Gálóczi I., Nagy S., Szili K.

Every table below is generated from the analysis files by the scripts in File S1 and can be regenerated from them; none was assembled by hand. Two items ship alongside this document rather than inside it because they are data rather than documents: `TableS2_all_estimates.csv` (8,304 rows) and File S1 itself (the extraction, pooling and figure code).

Table S1. PRISMA-S search strategy — the 58 query strings of the original PubMed search

The **58** queries of the original PubMed search, as executed through the NCBI E-utilities `esearch` endpoint. Nine further queries — the date-floor-removed re-runs identified at revision — and every execution against Europe PMC, Semantic Scholar and ClinicalTrials.gov are in **Table S1b**, which completes the PRISMA-S record. The table below repeats its header row once per tranche, so it carries 68 lines for 58 queries through the NCBI E-utilities `esearch` endpoint, with the UTC execution time, PubMed's true match count and the number of records retrieved. A query whose *retrieved* is below its *matched* was truncated by the retrieval cap; those are flagged **†** and are the queries enumerated in Section 2.8 item 5. Query strings are reproduced verbatim and are runnable as given. No language filter, no publication-type exclusion and no narrative-review filter was applied to any query.

Core tranche (8 queries)

Query name	Executed (UTC)	Matched	Retrieved	Query string
`pa-dose-mortality`	2026-08-22T09:12:11	172	172	`(("physical activity"[tiab] OR "exercise"[tiab] OR "aerobic exercise"[tiab] OR "resistance training"[tiab] OR "muscle-strengthening"[tiab]) AND ("dose-response"[tiab] OR "minimal dose"[tiab] OR "minimum dose"[tiab] OR "minimum effective`

Query name	Executed (UTC)	Matched	Retrieved	Query string
				dose"[tiab] OR "weekly volume"[tiab] OR "MET-minutes"[tiab]) AND ("all-cause mortality"[tiab] OR "longevity"[tiab] OR "lifespan"[tiab] OR "life expectancy"[tiab] OR "healthspan"[tiab])) AND ("2023/01/01"[dp] : "2026/12/31"[dp])
pa-guidelines-healthspan	2026-08-22T09:12:17	283	283	(("physical activity guidelines"[tiab] OR "exercise guidelines"[tiab] OR "physical activity recommendation*"[tiab] OR "WHO guidelines"[tiab] OR "consensus statement"[tiab]) AND ("physical activity"[tiab] OR "exercise"[tiab]) AND ("healthy ageing"[tiab] OR "healthy aging"[tiab] OR "healthspan"[tiab] OR "longevity"[tiab] OR "mortality"[tiab] OR "older adults"[tiab])) AND ("2023/01/01"[dp] : "2026/12/31"[dp])
protein-longevity	2026-08-22T09:12:23	191	191	(("protein intake"[tiab] OR "dietary protein"[tiab] OR "protein consumption"[tiab] OR "protein requirement*"[tiab]) AND ("longevity"[tiab] OR "healthspan"[tiab] OR "lifespan"[tiab] OR "life expectancy"[tiab] OR "all-cause mortality"[tiab] OR "healthy ageing"[tiab] OR "healthy

Query name	Executed (UTC)	Matched	Retrieved	Query string
				aging"[tiab])) AND ("2023/01/01"[dp] : "2026/12/31"[dp])
protein-plus-training	2026-08-22T09:12:30	128	128	(("resistance training"[tiab] OR "resistance exercise"[tiab] OR "physical activity"[tiab] OR "exercise"[tiab]) AND ("protein supplementation"[tiab] OR "protein intake"[tiab] OR "dietary protein"[tiab]) AND ("combined"[tiab] OR "adjunct"[tiab] OR "synerg*"[tiab] OR "multimodal"[tiab] OR "co-ingestion"[tiab] OR "interaction"[tiab]) AND ("older adult*"[tiab] OR "ageing"[tiab] OR "aging"[tiab] OR "sarcopenia"[tiab] OR "healthspan"[tiab])) AND ("2023/01/01"[dp] : "2026/12/31"[dp])
protein-restriction-mtor	2026-08-22T09:12:42	468	468	(("protein restriction"[tiab] OR "low-protein diet"[tiab] OR "mTOR"[tiab] OR "IGF-1"[tiab] OR "amino acid restriction"[tiab] OR "methionine restriction"[tiab]) AND ("longevity"[tiab] OR "lifespan"[tiab] OR "healthspan"[tiab] OR "ageing"[tiab] OR "aging"[tiab]) AND ("human*"[tiab] OR "older adult*"[tiab] OR "protein intake"[tiab] OR "dietary protein"[tiab])) AND ("2023/01/01"[dp] :

Query name	Executed (UTC)	Matched	Retrieved	Query string
				"2026/12/31"[dp])
protein-sarcopenia-dose	2026-08-22T09:12:26	190	190	(("protein intake"[tiab] OR "dietary protein"[tiab] OR "protein supplementation"[tiab] OR "leucine"[tiab] OR "essential amino acid*"[tiab]) AND ("sarcopenia"[tiab] OR "muscle mass"[tiab] OR "lean mass"[tiab] OR "muscle protein synthesis"[tiab] OR "frailty"[tiab]) AND ("older adult*"[tiab] OR "elderly"[tiab] OR "ageing"[tiab] OR "aging"[tiab]) AND ("recommendation*"[tiab] OR "requirement*"[tiab] OR "optimal"[tiab] OR "g/kg"[tiab] OR "dose"[tiab] OR "distribution"[tiab])) AND ("2023/01/01"[dp] : "2026/12/31"[dp])
sport-nutrition-longevity	2026-08-22T09:12:34	222	222	(("sports nutrition"[tiab] OR "sport nutrition"[tiab] OR "athlete*"[tiab] OR "masters athlete*"[tiab] OR "exercise nutrition"[tiab]) AND ("longevity"[tiab] OR "healthspan"[tiab] OR "lifespan"[tiab] OR "healthy ageing"[tiab] OR "healthy aging"[tiab] OR "biological age"[tiab])) AND ("2023/01/01"[dp] : "2026/12/31"[dp])
steps-vilpa-mortality	2026-08-22T09:12:39	108	108	(("steps per day"[tiab] OR "step count"[tiab] OR "daily steps"[tiab] OR

Query name	Executed (UTC)	Matched	Retrieved	Query string
				"accelerometer-measured"[tiab] OR "device-measured physical activity"[tiab] OR "vigorous intermittent lifestyle physical activity"[tiab] OR "VILPA"[tiab]) AND ("all-cause mortality"[tiab] OR "longevity"[tiab] OR "life expectancy"[tiab] OR "healthspan"[tiab] OR "cardiovascular mortality"[tiab])) AND ("2023/01/01"[dp] : "2026/12/31"[dp])

Supplementary tranche (4 queries)

Query name	Executed (UTC)	Matched	Retrieved	Query string
highprotein-safety-cvd	2026-08-22T10:31:55	121	121	(("high-protein diet"[tiab] OR "high protein intake"[tiab] OR "protein overconsumption"[tiab]) AND ("cardiovascular"[tiab] OR "mortality"[tiab] OR "safety"[tiab] OR "renal"[tiab] OR "kidney"[tiab]) AND ("randomized"[tiab] OR "trial"[tiab] OR "cohort"[tiab] OR "meta-analysis"[tiab])) AND ("2015/01/01"[dp] : "2026/12/31"[dp])
protein-mortality-meta	2026-08-22T10:30:34	17	17	((("protein intake"[tiab] OR "dietary protein"[tiab] OR "protein consumption"[tiab]) AND ("all-cause mortality"[tiab] OR "total

Query name	Executed (UTC)	Matched	Retrieved	Query string
				mortality"[tiab])) AND ("meta-analysis"[pt] OR "systematic review"[pt] OR "dose-response"[tiab] OR "pooled analysis"[tiab])) AND ("2015/01/01"[dp] : "2026/12/31"[dp])
protein-older-mortality	2026-08-22T10:41:46	85	85	(("protein intake"[tiab] OR "dietary protein"[tiab]) AND ("older adult*"[tiab] OR "elderly"[tiab] OR "aged"[tiab] OR "ageing"[tiab] OR "aging"[tiab]) AND ("all-cause mortality"[tiab] OR "survival"[tiab] OR "mortality risk"[tiab])) AND ("2015/01/01"[dp] : "2026/12/31"[dp])
protein-ukbiobank	2026-08-22T10:43:49	55	55	(("UK Biobank"[tiab] OR "NHANES"[tiab] OR "prospective cohort"[tiab]) AND ("protein intake"[tiab] OR "dietary protein"[tiab]) AND ("mortality"[tiab] OR "cardiovascular"[tiab])) AND ("2023/01/01"[dp] : "2026/12/31"[dp])

Population tranche (7 queries)

Query name	Executed (UTC)	Matched	Retrieved	Query string
cardiovascular	2026-08-22T17:44:06	112	112	(("protein intake"[tiab] OR "dietary protein"[tiab]) AND ("heart failure"[tiab] OR "coronary"[tiab] OR "myocardial"[tiab] OR "stroke"[tiab] OR

Query name	Executed (UTC)	Matched	Retrieved	Query string
				"cardiovascular disease"[tiab] OR "cardiac cachexia"[tiab]) AND ("mortality"[tiab] OR "prognosis"[tiab] OR "survival"[tiab] OR "g/kg"[tiab] OR "protein requirement*"[tiab])) AND ("2018/01/01"[dp] : "2026/12/31"[dp])
ckd	2026-08-23T02:09:30	96	96	(("protein intake"[tiab] OR "dietary protein"[tiab] OR "low-protein diet"[tiab]) AND ("g/kg"[tiab] OR "g per kg"[tiab] OR "protein requirement*"[tiab] OR "optimal protein"[tiab] OR "protein target"[tiab] OR "protein dose"[tiab]) AND ("chronic kidney disease"[tiab] OR "CKD"[tiab] OR "dialysis"[tiab] OR "eGFR"[tiab] OR "renal"[tiab]) AND ("mortality"[tiab] OR "progression"[tiab] OR "survival"[tiab])) AND ("2018/01/01"[dp] : "2026/12/31"[dp])
diabetes	2026-08-22T17:42:00	30	30	(("protein intake"[tiab] OR "dietary protein"[tiab]) AND ("g/kg"[tiab] OR "g per kg"[tiab] OR "grams per kilogram"[tiab] OR "protein requirement*"[tiab] OR "optimal protein"[tiab] OR "protein target"[tiab] OR

Query name	Executed (UTC)	Matched	Retrieved	Query string
				"protein dose"[tiab]) AND ("type 2 diabetes"[tiab] OR "diabetic"[tiab] OR "diabetes mellitus"[tiab]) AND ("mortality"[tiab] OR "nephropathy"[tiab] OR "glycemic"[tiab] OR "outcome*"[tiab])) AND ("2018/01/01"[dp] : "2026/12/31"[dp])
diabetes2†	2026-08-23T02:16:11	940	300	(("protein intake"[tiab] OR "dietary protein"[tiab] OR "high-protein diet"[tiab] OR "protein supplementation"[tiab]) AND ("type 2 diabetes"[tiab] OR "diabetes mellitus"[tiab] OR "diabetic"[tiab] OR "insulin resistance"[tiab] OR "glycaemic"[tiab] OR "glycemic"[tiab] OR "HbA1c"[tiab])) AND ("2015/01/01"[dp] : "2026/12/31"[dp])
healthy	2026-08-22T11:08:53	91	91	(("protein intake"[tiab] OR "dietary protein"[tiab]) AND ("g/kg"[tiab] OR "g per kg"[tiab] OR "grams per kilogram"[tiab] OR "protein requirement*"[tiab] OR "optimal protein"[tiab] OR "protein target"[tiab] OR "protein dose"[tiab]) AND ("healthy adult*"[tiab] OR "general population"[tiab] OR

Query name	Executed (UTC)	Matched	Retrieved	Query string
				"community-dwelling"[tiab] OR "healthy older"[tiab]) AND ("mortality"[tiab] OR "sarcopenia"[tiab] OR "muscle mass"[tiab] OR "healthspan"[tiab] OR "requirement*"[tiab])) AND ("2018/01/01"[dp] : "2026/12/31"[dp])
highprotein-upper†	2026-08-23T02:30:53	1,023	300	(("high-protein diet"[tiab] OR "high protein intake"[tiab] OR "1.6 g/kg"[tiab] OR "2.0 g/kg"[tiab] OR "2.2 g/kg"[tiab] OR "upper limit"[tiab] OR "safe upper"[tiab] OR "tolerable upper intake"[tiab]) AND ("protein"[tiab]) AND ("safety"[tiab] OR "renal function"[tiab] OR "kidney function"[tiab] OR "adverse"[tiab] OR "upper limit"[tiab] OR "randomized"[tiab] OR "randomised"[tiab])) AND ("2015/01/01"[dp] : "2026/12/31"[dp])
obesity	2026-08-22T11:13:22	110	110	(("protein intake"[tiab] OR "dietary protein"[tiab] OR "high-protein diet"[tiab]) AND ("g/kg"[tiab] OR "g per kg"[tiab] OR "grams per kilogram"[tiab] OR "protein requirement*"[tiab] OR "optimal protein"[tiab] OR "protein target"[tiab] OR "protein dose"[tiab]) AND ("obesity"[tiab] OR

Query name	Executed (UTC)	Matched	Retrieved	Query string
				"obese"[tiab] OR "weight loss"[tiab] OR "bariatric"[tiab] OR "sarcopenic obesity"[tiab]) AND ("mortality"[tiab] OR "lean mass"[tiab] OR "muscle"[tiab] OR "outcome*"[tiab])) AND ("2018/01/01"[dp] : "2026/12/31"[dp])

Randomised tranche (12 queries)

Query name	Executed (UTC)	Matched	Retrieved	Query string
rct-exercise-clinical-pooled	2026-08-23T09:55:17	91	91	(("exercise training"[tiab] OR "cardiac rehabilitation"[tiab] OR "exercise intervention"[tiab] OR "supervised exercise"[tiab]) AND ("heart failure"[tiab] OR "coronary artery disease"[tiab] OR "type 2 diabetes"[tiab] OR "chronic kidney disease"[tiab] OR "cancer survivor*"[tiab]) AND ("mortality"[tiab] OR "all-cause"[tiab] OR "hospitalisation"[tiab] OR "hospitalization"[tiab] OR "MACE"[tiab]) AND ("randomized controlled trial"[pt] OR "controlled clinical trial"[pt] OR "randomized"[tiab] OR "randomised"[tiab] OR "randomly assigned"[tiab] OR "placebo"[tiab] OR

Query name	Executed (UTC)	Matched	Retrieved	Query string
				"trial"[ti]) AND ("meta-analysis"[pt] OR "systematic review"[pt] OR "meta-analysis"[tiab] OR "pooled analysis"[tiab])) AND ("2015/01/01"[dp] : "2026/12/31"[dp])
rct-exercise-dose-pooled	2026-08-23T10:01:16	63	63	(("exercise dose"[tiab] OR "exercise volume"[tiab] OR "dose-response"[tiab] OR "minimal dose"[tiab] OR "training frequency"[tiab] OR "weekly sets"[tiab]) AND ("randomized"[tiab] OR "randomised"[tiab]) AND ("muscle strength"[tiab] OR "cardiorespiratory fitness"[tiab] OR "VO2"[tiab] OR "functional capacity"[tiab]) AND ("meta-analysis"[pt] OR "systematic review"[pt] OR "meta-analysis"[tiab] OR "pooled analysis"[tiab])) AND ("2015/01/01"[dp] : "2026/12/31"[dp])
rct-exercise-older-outcomes†	2026-08-23T09:38:38	551	250	(("exercise training"[tiab] OR "physical activity intervention"[tiab] OR "structured exercise"[tiab] OR "resistance training"[tiab] OR "aerobic training"[tiab]) AND ("mortality"[tiab] OR "major mobility disability"[tiab] OR "disability"[tiab] OR "frailty"[tiab] OR "falls"[tiab] OR

Query name	Executed (UTC)	Matched	Retrieved	Query string
				"hospitalisation"[tiab] OR "hospitalization"[tiab]) AND ("older adult*"[tiab] OR "elderly"[tiab] OR "aged"[tiab]) AND ("randomized controlled trial"[pt] OR "controlled clinical trial"[pt] OR "randomized"[tiab] OR "randomised"[tiab] OR "randomly assigned"[tiab] OR "placebo"[tiab] OR "trial"[ti])) AND ("2015/01/01"[dp] : "2026/12/31"[dp])
rct-highprotein-renal-safety	2026-08-23T08:37:46	102	102	(("high-protein diet"[tiab] OR "high protein intake"[tiab] OR "protein supplementation"[tiab]) AND ("renal function"[tiab] OR "kidney function"[tiab] OR "glomerular filtration"[tiab] OR "eGFR"[tiab] OR "albuminuria"[tiab] OR "creatinine"[tiab] OR "safety"[tiab] OR "adverse event*"[tiab]) AND ("randomized controlled trial"[pt] OR "controlled clinical trial"[pt] OR "randomized"[tiab] OR "randomised"[tiab] OR "randomly assigned"[tiab] OR "placebo"[tiab] OR "trial"[ti])) AND ("2015/01/01"[dp] : "2026/12/31"[dp])

Query name	Executed (UTC)	Matched	Retrieved	Query string
rct-lpd-ckd	2026-08-23T08:44:49	61	61	(("low-protein diet"[tiab] OR "protein restriction"[tiab] OR "very low protein diet"[tiab] OR "ketoanalogue*"[tiab] OR "keto-analogue*"[tiab]) AND ("chronic kidney disease"[tiab] OR "CKD"[tiab] OR "dialysis"[tiab] OR "diabetic nephropathy"[tiab] OR "diabetic kidney disease"[tiab]) AND ("mortality"[tiab] OR "progression"[tiab] OR "end-stage"[tiab] OR "eGFR"[tiab] OR "proteinuria"[tiab]) AND ("randomized controlled trial"[pt] OR "controlled clinical trial"[pt] OR "randomized"[tiab] OR "randomised"[tiab] OR "randomly assigned"[tiab] OR "placebo"[tiab] OR "trial"[ti])) AND ("2015/01/01"[dp] : "2026/12/31"[dp])
rct-multidomain †	2026-08-23T10:05:15	309	250	(("multidomain"[tiab] OR "multicomponent"[tiab] OR "lifestyle intervention"[tiab]) AND ("exercise"[tiab] OR "physical activity"[tiab]) AND ("protein"[tiab] OR "nutrition"[tiab] OR "diet"[tiab]) AND ("frailty"[tiab] OR "sarcopenia"[tiab] OR "cognitive"[tiab] OR "disability"[tiab] OR "healthy ageing"[tiab]

Query name	Executed (UTC)	Matched	Retrieved	Query string
				OR "healthy aging"[tiab]) AND ("randomized controlled trial"[pt] OR "controlled clinical trial"[pt] OR "randomized"[tiab] OR "randomised"[tiab] OR "randomly assigned"[tiab] OR "placebo"[tiab] OR "trial"[ti])) AND ("2015/01/01"[dp] : "2026/12/31"[dp])
rct-protein-cvd	2026-08-23T09:27:01	207	207	(("protein intake"[tiab] OR "dietary protein"[tiab] OR "protein supplementation"[tiab]) AND ("heart failure"[tiab] OR "cardiovascular"[tiab] OR "blood pressure"[tiab] OR "LDL cholesterol"[tiab] OR "cardiac"[tiab]) AND ("randomized controlled trial"[pt] OR "controlled clinical trial"[pt] OR "randomized"[tiab] OR "randomised"[tiab] OR "randomly assigned"[tiab] OR "placebo"[tiab] OR "trial"[ti])) AND ("2015/01/01"[dp] : "2026/12/31"[dp])
rct-protein-dose-response	2026-08-23T09:14:51	183	183	(("protein distribution"[tiab] OR "per-meal protein"[tiab] OR "protein dose"[tiab] OR "dose-response"[tiab] OR "g/kg"[tiab] OR "protein timing"[tiab]) AND ("muscle protein synthesis"[tiab] OR

Query name	Executed (UTC)	Matched	Retrieved	Query string
				"myofibrillar"[tiab] OR "nitrogen balance"[tiab] OR "protein requirement*"[tiab] OR "indicator amino acid oxidation"[tiab]) AND ("randomized controlled trial"[pt] OR "controlled clinical trial"[pt] OR "randomized"[tiab] OR "randomised"[tiab] OR "randomly assigned"[tiab] OR "placebo"[tiab] OR "trial"[ti])) AND ("2015/01/01"[dp] : "2026/12/31"[dp])
rct-protein-obesity-leanmass†	2026-08-23T09:00:45	261	250	(("high-protein diet"[tiab] OR "protein intake"[tiab] OR "protein supplementation"[tiab] OR "energy-restricted"[tiab]) AND ("obesity"[tiab] OR "obese"[tiab] OR "weight loss"[tiab] OR "bariatric"[tiab] OR "GLP-1"[tiab] OR "semaglutide"[tiab] OR "tirzepatide"[tiab]) AND ("lean mass"[tiab] OR "fat-free mass"[tiab] OR "muscle mass"[tiab] OR "body composition"[tiab] OR "sarcopenic obesity"[tiab]) AND ("randomized controlled trial"[pt] OR "controlled clinical trial"[pt] OR "randomized"[tiab] OR "randomised"[tiab] OR "randomly assigned"[tiab] OR "placebo"[tiab] OR

Query name	Executed (UTC)	Matched	Retrieved	Query string
				"trial"[ti])) AND ("2015/01/01"[dp] : "2026/12/31"[dp])
rct-protein-older-function†	2026-08-23T08:22:59	503	250	(("protein supplementation"[tiab] OR "dietary protein"[tiab] OR "protein intake"[tiab] OR "whey protein"[tiab] OR "leucine"[tiab] OR "essential amino acid*"[tiab]) AND ("older adult*"[tiab] OR "elderly"[tiab] OR "sarcopenia"[tiab] OR "frailty"[tiab]) AND ("lean mass"[tiab] OR "muscle mass"[tiab] OR "muscle strength"[tiab] OR "grip strength"[tiab] OR "physical function"[tiab] OR "gait speed"[tiab] OR "SPPB"[tiab]) AND ("randomized controlled trial"[pt] OR "controlled clinical trial"[pt] OR "randomized"[tiab] OR "randomised"[tiab] OR "randomly assigned"[tiab] OR "placebo"[tiab] OR "trial"[ti])) AND ("2015/01/01"[dp] : "2026/12/31"[dp])
rct-protein-plus-rt-pooled	2026-08-23T09:22:08	48	48	(("protein supplementation"[tiab] OR "dietary protein"[tiab]) AND ("resistance training"[tiab] OR "resistance exercise"[tiab] OR "strength training"[tiab]) AND ("lean mass"[tiab] OR

Query name	Executed (UTC)	Matched	Retrieved	Query string
				"muscle mass"[tiab] OR "strength"[tiab] OR "hypertrophy"[tiab]) AND ("meta-analysis"[pt] OR "systematic review"[pt] OR "meta-analysis"[tiab] OR "pooled analysis"[tiab]) AND ("randomized controlled trial"[pt] OR "controlled clinical trial"[pt] OR "randomized"[tiab] OR "randomised"[tiab] OR "randomly assigned"[tiab] OR "placebo"[tiab] OR "trial"[ti])) AND ("2015/01/01"[dp] : "2026/12/31"[dp])
rct-protein-t2dm	2026-08-23T08:47:13	227	227	(("high-protein diet"[tiab] OR "protein intake"[tiab] OR "dietary protein"[tiab] OR "protein supplementation"[tiab]) AND ("type 2 diabetes"[tiab] OR "insulin resistance"[tiab] OR "HbA1c"[tiab] OR "glycaemic control"[tiab] OR "glycemic control"[tiab] OR "insulin sensitivity"[tiab]) AND ("randomized controlled trial"[pt] OR "controlled clinical trial"[pt] OR "randomized"[tiab] OR "randomised"[tiab] OR "randomly assigned"[tiab] OR "placebo"[tiab] OR "trial"[ti])) AND ("2015/01/01"[dp] :

Query name	Executed (UTC)	Matched	Retrieved	Query string
				"2026/12/31"[dp])

Incretin tranche (6 queries)

Query name	Executed (UTC)	Matched	Retrieved	Query string
glp1-guidance†	2026-08-23T12:39:36	267	250	(("GLP-1"[tiab] OR "GLP1"[tiab] OR "glucagon-like peptide-1"[tiab] OR "glucagon like peptide 1"[tiab] OR "incretin"[tiab] OR "semaglutide"[tiab] OR "tirzepatide"[tiab] OR "liraglutide"[tiab] OR "dulaglutide"[tiab] OR "exenatide"[tiab] OR "retatrutide"[tiab] OR "GIP/GLP-1"[tiab]) AND ("consensus"[tiab] OR "position statement"[tiab] OR "guideline*"[tiab] OR "recommendation*"[tiab] OR "practical guidance"[tiab] OR "expert opinion"[tiab]) AND ("nutrition"[tiab] OR "protein"[tiab] OR "muscle"[tiab] OR "lean mass"[tiab] OR "sarcopenia"[tiab])) AND ("2015/01/01"[dp] : "2026/12/31"[dp])
glp1-leanmass†	2026-08-23T11:48:47	1,120	250	(("GLP-1"[tiab] OR "GLP1"[tiab] OR "glucagon-like peptide-1"[tiab] OR "glucagon like peptide 1"[tiab] OR "incretin"[tiab] OR "semaglutide"[tiab] OR "tirzepatide"[tiab] OR "liraglutide"[tiab] OR "dulaglutide"[tiab] OR "exenatide"[tiab] OR "retatrutide"[tiab] OR "GIP/GLP-1"[tiab]) AND

Query name	Executed (UTC)	Matched	Retrieved	Query string
				("lean mass"[tiab] OR "fat-free mass"[tiab] OR "fat free mass"[tiab] OR "muscle mass"[tiab] OR "skeletal muscle"[tiab] OR "sarcopenia"[tiab] OR "sarcopenic obesity"[tiab] OR "muscle strength"[tiab] OR "grip strength"[tiab] OR "body composition"[tiab] OR "appendicular"[tiab] OR "muscle loss"[tiab])) AND ("2015/01/01"[dp] : "2026/12/31"[dp])
glp1-preservation	2026-08-23T12:32:10	121	121	((("GLP-1"[tiab] OR "GLP1"[tiab] OR "glucagon-like peptide-1"[tiab] OR "glucagon like peptide 1"[tiab] OR "incretin"[tiab] OR "semaglutide"[tiab] OR "tirzepatide"[tiab] OR "liraglutide"[tiab] OR "dulaglutide"[tiab] OR "exenatide"[tiab] OR "retatrutide"[tiab] OR "GIP/GLP-1"[tiab]) OR "weight loss"[tiab] OR "caloric restriction"[tiab] OR "bariatric"[tiab]) AND ("muscle-preserving"[tiab] OR "muscle preservation"[tiab] OR "preserve lean"[tiab] OR "preserving lean"[tiab] OR "iatrogenic sarcopenia"[tiab] OR "sarcopenic obesity"[tiab]) AND ("protein"[tiab] OR "resistance training"[tiab])) AND ("2015/01/01"[dp] :

Query name	Executed (UTC)	Matched	Retrieved	Query string
				"2026/12/31"[dp])
glp1-protein†	2026-08-23T12:05:10	497	250	(("GLP-1"[tiab] OR "GLP1"[tiab] OR "glucagon-like peptide-1"[tiab] OR "glucagon like peptide 1"[tiab] OR "incretin"[tiab] OR "semaglutide"[tiab] OR "tirzepatide"[tiab] OR "liraglutide"[tiab] OR "dulaglutide"[tiab] OR "exenatide"[tiab] OR "retatrutide"[tiab] OR "GIP/GLP-1"[tiab]) AND ("protein intake"[tiab] OR "dietary protein"[tiab] OR "protein supplementation"[tiab] OR "high-protein"[tiab] OR "whey protein"[tiab] OR "leucine"[tiab] OR "essential amino acid*"[tiab] OR "protein requirement*"[tiab] OR "g/kg"[tiab])) AND ("2015/01/01"[dp] : "2026/12/31"[dp])
glp1-randomised†	2026-08-23T12:50:34	267	250	(("GLP-1"[tiab] OR "GLP1"[tiab] OR "glucagon-like peptide-1"[tiab] OR "glucagon like peptide 1"[tiab] OR "incretin"[tiab] OR "semaglutide"[tiab] OR "tirzepatide"[tiab] OR "liraglutide"[tiab] OR "dulaglutide"[tiab] OR "exenatide"[tiab] OR "retatrutide"[tiab] OR "GIP/GLP-1"[tiab]) AND ("lean mass"[tiab] OR "fat-free mass"[tiab] OR "fat free mass"[tiab] OR "muscle mass"[tiab] OR "skeletal muscle"[tiab] OR "sarcopenia"[tiab] OR

Query name	Executed (UTC)	Matched	Retrieved	Query string
				"sarcopenic obesity"[tiab] OR "muscle strength"[tiab] OR "grip strength"[tiab] OR "body composition"[tiab] OR "appendicular"[tiab] OR "muscle loss"[tiab]) AND ("randomized controlled trial"[pt] OR "randomized"[tiab] OR "randomised"[tiab] OR "placebo"[tiab] OR "meta-analysis"[pt] OR "systematic review"[pt])) AND ("2015/01/01"[dp] : "2026/12/31"[dp])
glp1-training	2026-08-23T12:21:19	155	155	(("GLP-1"[tiab] OR "GLP1"[tiab] OR "glucagon-like peptide-1"[tiab] OR "glucagon like peptide 1"[tiab] OR "incretin"[tiab] OR "semaglutide"[tiab] OR "tirzepatide"[tiab] OR "liraglutide"[tiab] OR "dulaglutide"[tiab] OR "exenatide"[tiab] OR "retatrutide"[tiab] OR "GIP/GLP-1"[tiab]) AND ("resistance training"[tiab] OR "resistance exercise"[tiab] OR "strength training"[tiab] OR "exercise training"[tiab] OR "physical activity"[tiab]) AND ("lean mass"[tiab] OR "fat-free mass"[tiab] OR "fat free mass"[tiab] OR "muscle mass"[tiab] OR "skeletal muscle"[tiab] OR "sarcopenia"[tiab] OR

Query name	Executed (UTC)	Matched	Retrieved	Query string
				"sarcopenic obesity"[tiab] OR "muscle strength"[tiab] OR "grip strength"[tiab] OR "body composition"[tiab] OR "appendicular"[tiab] OR "muscle loss"[tiab])) AND ("2015/01/01"[dp] : "2026/12/31"[dp])

Microbiome tranche (5 queries)

Query name	Executed (UTC)	Matched	Retrieved	Query string
mb-exercise†	2026-08-23T15:48:26	7,253	250	(("gut microbiota"[tiab] OR "gut microbiome"[tiab] OR "intestinal microbiota"[tiab] OR "microbiome"[tiab] OR "microbiota"[tiab] OR "short-chain fatty acid*"[tiab] OR "SCFA"[tiab] OR "butyrate"[tiab] OR "propionate"[tiab] OR "faecal microbiota"[tiab] OR "fecal microbiota"[tiab]) AND ("exercise"[tiab] OR "physical activity"[tiab] OR "resistance training"[tiab] OR "endurance training"[tiab] OR "athlete*"[tiab] OR "lactate"[tiab] OR "Veillonella"[tiab])) AND ("2015/01/01"[dp] : "2026/12/31"[dp])
mb-gutmuscle-axis†	2026-08-23T16:35:29	743	250	(("gut-muscle axis"[tiab] OR "gut muscle axis"[tiab] OR "microbiota-gut-

Query name	Executed (UTC)	Matched	Retrieved	Query string
				muscle"[tiab] OR "muscle-gut"[tiab]) OR (("gut microbiota"[tiab] OR "gut microbiome"[tiab] OR "intestinal microbiota"[tiab] OR "microbiome"[tiab] OR "microbiota"[tiab] OR "short-chain fatty acid*"[tiab] OR "SCFA"[tiab] OR "butyrate"[tiab] OR "propionate"[tiab] OR "faecal microbiota"[tiab] OR "fecal microbiota"[tiab]) AND ("sarcopenia"[tiab] OR "muscle mass"[tiab] OR "skeletal muscle"[tiab] OR "muscle strength"[tiab] OR "grip strength"[tiab] OR "physical function"[tiab] OR "frailty"[tiab] OR "lean mass"[tiab] OR "muscle protein synthesis"[tiab]) AND ("randomized"[tiab] OR "randomised"[tiab] OR "probiotic*"[tiab] OR "prebiotic*"[tiab] OR "synbiotic*"[tiab] OR "fibre"[tiab] OR "fiber"[tiab]))) AND ("2015/01/01"[dp] : "2026/12/31"[dp])
mb-longevity†	2026-08-23T16:14:08	1,797	250	(("gut microbiota"[tiab] OR "gut microbiome"[tiab] OR "intestinal microbiota"[tiab] OR "microbiome"[tiab] OR "microbiota"[tiab] OR "short-chain fatty

Query name	Executed (UTC)	Matched	Retrieved	Query string
				acid*"[tiab] OR "SCFA"[tiab] OR "butyrate"[tiab] OR "propionate"[tiab] OR "faecal microbiota"[tiab] OR "fecal microbiota"[tiab]) AND ("longevity"[tiab] OR "healthspan"[tiab] OR "ageing"[tiab] OR "aging"[tiab] OR "all-cause mortality"[tiab] OR "centenarian*"[tiab]) AND ("human*"[tiab] OR "older adult*"[tiab] OR "cohort"[tiab])) AND ("2015/01/01"[dp] : "2026/12/31"[dp])
mb-muscle†	2026-08-23T14:33:10	1,815	250	(("gut microbiota"[tiab] OR "gut microbiome"[tiab] OR "intestinal microbiota"[tiab] OR "microbiome"[tiab] OR "microbiota"[tiab] OR "short-chain fatty acid*"[tiab] OR "SCFA"[tiab] OR "butyrate"[tiab] OR "propionate"[tiab] OR "faecal microbiota"[tiab] OR "fecal microbiota"[tiab]) AND ("sarcopenia"[tiab] OR "muscle mass"[tiab] OR "skeletal muscle"[tiab] OR "muscle strength"[tiab] OR "grip strength"[tiab] OR "physical function"[tiab] OR "frailty"[tiab] OR "lean mass"[tiab] OR "muscle protein synthesis"[tiab])) AND

Query name	Executed (UTC)	Matched	Retrieved	Query string
				("2015/01/01"[dp] : "2026/12/31"[dp])
mb-protein†	2026-08-23T15:23:57	9,264	250	(("gut microbiota"[tiab] OR "gut microbiome"[tiab] OR "intestinal microbiota"[tiab] OR "microbiome"[tiab] OR "microbiota"[tiab] OR "short-chain fatty acid*"[tiab] OR "SCFA"[tiab] OR "butyrate"[tiab] OR "propionate"[tiab] OR "faecal microbiota"[tiab] OR "fecal microbiota"[tiab]) AND ("dietary protein"[tiab] OR "protein intake"[tiab] OR "protein fermentation"[tiab] OR "plant protein"[tiab] OR "animal protein"[tiab] OR "amino acid"[tiab] OR "putrefaction"[tiab] OR "p-cresol"[tiab] OR "indoxyl sulfate"[tiab] OR "TMAO"[tiab] OR "trimethylamine"[tiab])) AND ("2015/01/01"[dp] : "2026/12/31"[dp])

Dietary assessment tranche (4 queries)

Query name	Executed (UTC)	Matched	Retrieved	Query string
ai-diet-assessment†	2026-08-23T15:10:05	1,018	150	(("artificial intelligence"[tiab] OR "machine learning"[tiab] OR "deep learning"[tiab] OR "computer vision"[tiab] OR "convolutional neural network*"[tiab] OR "image-based"[tiab] OR

Query name	Executed (UTC)	Matched	Retrieved	Query string
				"image based"[tiab] OR "smartphone"[tiab] OR "large language model*"[tiab] OR "automated"[tiab]) AND ("dietary assessment"[tiab] OR "dietary intake"[tiab] OR "food record*"[tiab] OR "24-hour recall"[tiab] OR "food frequency questionnaire"[tiab] OR "food photograph*"[tiab] OR "portion size"[tiab] OR "food recognition"[tiab] OR "nutrient estimation"[tiab] OR "energy intake"[tiab])) AND ("2018/01/01"[dp] : "2026/12/31"[dp])
ai-diet-validation†	2026-08-23T15:24:02	380	150	(("artificial intelligence"[tiab] OR "machine learning"[tiab] OR "deep learning"[tiab] OR "computer vision"[tiab] OR "convolutional neural network*"[tiab] OR "image-based"[tiab] OR "image based"[tiab] OR "smartphone"[tiab] OR "large language model*"[tiab] OR "automated"[tiab]) AND ("dietary assessment"[tiab] OR "dietary intake"[tiab] OR "food record*"[tiab] OR "24-hour recall"[tiab] OR "food frequency questionnaire"[tiab] OR "food photograph*"[tiab] OR "portion size"[tiab] OR "food

Query name	Executed (UTC)	Matched	Retrieved	Query string
				recognition"[tiab] OR "nutrient estimation"[tiab] OR "energy intake"[tiab]) AND ("validity"[tiab] OR "validation"[tiab] OR "accuracy"[tiab] OR "agreement"[tiab] OR "reliability"[tiab] OR "weighed food record"[tiab] OR "doubly labelled water"[tiab] OR "doubly labeled water"[tiab])) AND ("2018/01/01"[dp] : "2026/12/31"[dp])
ai-protein-monitoring	2026-08-23T15:36:51	147	147	(("artificial intelligence"[tiab] OR "machine learning"[tiab] OR "deep learning"[tiab] OR "computer vision"[tiab] OR "convolutional neural network*"[tiab] OR "image-based"[tiab] OR "image based"[tiab] OR "smartphone"[tiab] OR "large language model*"[tiab] OR "automated"[tiab]) AND ("protein intake"[tiab] OR "dietary protein"[tiab] OR "macronutrient*"[tiab]) AND ("assessment"[tiab] OR "monitoring"[tiab] OR "tracking"[tiab] OR "estimation"[tiab])) AND ("2018/01/01"[dp] : "2026/12/31"[dp])
selfreport-error†	2026-08-23T15:49:35	155	150	(("self-report*"[tiab] AND ("misreporting"[tiab] OR "under-reporting"[tiab] OR "underreporting"[tiab] OR "measurement

Query name	Executed (UTC)	Matched	Retrieved	Query string
				error"[tiab] OR "bias"[tiab])) AND ("dietary intake"[tiab] OR "energy intake"[tiab] OR "food frequency questionnaire"[tiab] OR "24-hour recall"[tiab])) AND ("2018/01/01"[dp] : "2026/12/31"[dp])

Physical-activity expansion tranche (5 queries)

Query name	Executed (UTC)	Matched	Retrieved	Query string
pa-clinical-populations	2026-08-23T13:41:56	182	182	(("physical activity"[tiab] OR "exercise"[tiab]) AND ("cancer survivor*"[tiab] OR "coronary"[tiab] OR "heart failure"[tiab] OR "chronic kidney disease"[tiab] OR "COPD"[tiab] OR "type 2 diabetes"[tiab] OR "stroke"[tiab] OR "hypertension"[tiab]) AND ("meeting"[tiab] OR "guideline*"[tiab] OR "recommend*"[tiab] OR "active vs"[tiab] OR "inactive"[tiab]) AND ("all-cause mortality"[tiab] OR "all cause mortality"[tiab] OR "total mortality"[tiab]) AND ("prospective cohort"[tiab] OR "cohort study"[tiab] OR "prospective study"[tiab] OR "follow-up study"[tiab] OR "hazard ratio*"[tiab])) AND ("2010/01/01"[dp] : "2026/12/31"[dp])

Query name	Executed (UTC)	Matched	Retrieved	Query string
pa-device-measured	2026-08-23T13:41:48	169	169	(("accelerometer"[tiab] OR "accelerometry"[tiab] OR "device-measured"[tiab] OR "device measured"[tiab] OR "wrist-worn"[tiab] OR "wearable"[tiab] OR "actigraph*"[tiab]) AND ("physical activity"[tiab] OR "MVPA"[tiab] OR "steps"[tiab]) AND ("all-cause mortality"[tiab] OR "all cause mortality"[tiab] OR "total mortality"[tiab]) AND ("prospective cohort"[tiab] OR "cohort study"[tiab] OR "prospective study"[tiab] OR "follow-up study"[tiab] OR "hazard ratio*"[tiab])) AND ("2010/01/01"[dp] : "2026/12/31"[dp])
pa-guideline-adherence	2026-08-23T13:41:31	130	130	(("physical activity guideline*"[tiab] OR "meeting the guidelines"[tiab] OR "guideline-concordant"[tiab] OR "guideline adherence"[tiab] OR "WHO recommendation*"[tiab] OR "150 min"[tiab] OR "150 minutes"[tiab] OR "500-1000 MET"[tiab] OR "600 MET"[tiab]) AND ("physical activity"[tiab] OR "exercise"[tiab] OR "MVPA"[tiab]) AND ("all-cause mortality"[tiab] OR "all cause mortality"[tiab] OR "total mortality"[tiab])

Query name	Executed (UTC)	Matched	Retrieved	Query string
				AND ("prospective cohort"[tiab] OR "cohort study"[tiab] OR "prospective study"[tiab] OR "follow-up study"[tiab] OR "hazard ratio*"[tiab])) AND ("2010/01/01"[dp] : "2026/12/31"[dp])
pa-pooled-cohorts	2026-08-23T13:42:03	71	71	(("pooled analysis"[tiab] OR "pooled cohort"[tiab] OR "consortium"[tiab] OR "harmonised"[tiab] OR "harmonized"[tiab] OR "individual participant data"[tiab]) AND ("physical activity"[tiab] OR "exercise"[tiab]) AND ("all-cause mortality"[tiab] OR "all cause mortality"[tiab] OR "total mortality"[tiab])) AND ("2010/01/01"[dp] : "2026/12/31"[dp])
pa-quantile-contrast	2026-08-23T13:41:39	144	144	(("leisure-time physical activity"[tiab] OR "leisure time physical activity"[tiab] OR "total physical activity"[tiab] OR "moderate-to-vigorous physical activity"[tiab] OR "MVPA"[tiab]) AND ("most active"[tiab] OR "highest quartile"[tiab] OR "highest quintile"[tiab] OR "highest tertile"[tiab] OR "inactive"[tiab] OR "sedentary"[tiab] OR "physically inactive"[tiab]) AND ("all-cause mortality"[tiab] OR "all

Query name	Executed (UTC)	Matched	Retrieved	Query string
				cause mortality"[tiab] OR "total mortality"[tiab]) AND ("prospective cohort"[tiab] OR "cohort study"[tiab] OR "prospective study"[tiab] OR "follow-up study"[tiab] OR "hazard ratio*"[tiab])) AND ("2010/01/01"[dp] : "2026/12/31"[dp])

Hypertrophy tranche (4 queries)

Query name	Executed (UTC)	Matched	Retrieved	Query string
hyp-athlete-req†	2026-08-23	260	150	(("athlete*"[tiab] OR "resistance-trained"[tiab] OR "resistance trained"[tiab] OR "bodybuilder*"[tiab] OR "strength athlete*"[tiab]) AND ("protein requirement*"[tiab] OR "protein intake"[tiab] OR "protein recommendation*"[tiab] OR "g/kg"[tiab]) AND ("position stand"[tiab] OR "consensus"[tiab] OR "recommendation*"[tiab] OR "requirement*"[tiab] OR "hypertrophy"[tiab] OR "lean mass"[tiab])) AND ("2010/01/01"[dp] : "2026/12/31"[dp])
hyp-dose-response†	2026-08-23	184	150	(("protein supplementation"[tiab] OR "protein intake"[tiab] OR "dietary protein"[tiab]) AND ("resistance training"[tiab] OR "resistance

Query name	Executed (UTC)	Matched	Retrieved	Query string
				exercise"[tiab] OR "strength training"[tiab]) AND ("dose-response"[tiab] OR "plateau"[tiab] OR "breakpoint"[tiab] OR "optimal"[tiab] OR "g/kg"[tiab] OR "threshold"[tiab]) AND ("fat-free mass"[tiab] OR "lean body mass"[tiab] OR "muscle mass"[tiab] OR "hypertrophy"[tiab] OR "1RM"[tiab] OR "strength"[tiab])) AND ("2010/01/01"[dp] : "2026/12/31"[dp])
hyp-iaao-nbal	2026-08-23	118	118	(("indicator amino acid oxidation"[tiab] OR "nitrogen balance"[tiab] OR "protein requirement*"[tiab]) AND ("resistance training"[tiab] OR "athlete*"[tiab] OR "exercise"[tiab] OR "endurance"[tiab])) AND ("2010/01/01"[dp] : "2026/12/31"[dp])
hyp-veryhigh†	2026-08-23	308	150	(("high protein"[tiab] OR "2 g/kg"[tiab] OR "2.2 g/kg"[tiab] OR "3 g/kg"[tiab] OR "supraphysiological"[tiab] OR "very high protein"[tiab]) AND ("resistance training"[tiab] OR "body composition"[tiab] OR "lean mass"[tiab]) AND ("randomized"[tiab] OR "randomised"[tiab] OR "trial"[tiab])) AND ("2010/01/01"[dp] : "2026/12/31"[dp])

Mediator tranche (3 queries)

Query name	Executed (UTC)	Matched	Retrieved	Query string
med-grip-mortality†	2026-08-23	558	150	(("handgrip strength"[tiab] OR "grip strength"[tiab] OR "muscle strength"[tiab]) AND ("all-cause mortality"[tiab] OR "mortality"[tiab]) AND ("meta-analysis"[pt] OR "systematic review"[pt] OR "dose-response"[tiab] OR "per 5 kg"[tiab] OR "per kg"[tiab] OR "hazard ratio"[tiab])) AND ("2010/01/01"[dp] : "2026/12/31"[dp])
med-musclemass-mortality	2026-08-23	76	76	(("muscle mass"[tiab] OR "lean body mass"[tiab] OR "fat-free mass"[tiab] OR "skeletal muscle index"[tiab]) AND ("mortality"[tiab] OR "survival"[tiab]) AND ("cohort"[tiab] OR "prospective"[tiab]) AND ("meta-analysis"[pt] OR "dose-response"[tiab])) AND ("2010/01/01"[dp] : "2026/12/31"[dp])
med-sarcopenia-mortality†	2026-08-23	263	150	(("sarcopenia"[tiab] OR "low muscle mass"[tiab] OR "appendicular lean mass"[tiab] OR "muscle wasting"[tiab]) AND ("all-cause mortality"[tiab] OR "cardiovascular mortality"[tiab] OR "major adverse cardiovascular"[tiab] OR "MACE"[tiab]) AND ("meta-analysis"[pt] OR "systematic review"[pt] OR "pooled"[tiab] OR "hazard ratio"[tiab])) AND ("2010/01/01"[dp] :

Query name	Executed (UTC)	Matched	Retrieved	Query string
				"2026/12/31"[dp])

Totals. 58 queries · 34,510 records matched · 9,824 retrieved · 23 queries truncated by the retrieval cap (†) · 35 retrieved every matching record.

Table S1b. The remaining 153 logged executions that complete the PRISMA-S record — Europe PMC, Semantic Scholar and ClinicalTrials.gov

Table S1 lists the **58** queries of the original PubMed search. This table lists the remaining **153** logged executions: the **9** date-floor-removed PubMed re-runs (suffix `-1990`, added at revision after the 2023 floor was found to have excluded two pivotal cohorts), and every execution against **Europe PMC**, **Semantic Scholar** and **ClinicalTrials.gov**. Across the two tables the search comprises **67 distinct query strings** run against three bibliographic databases, plus 10 trial-register queries. Together the executions below matched **146,240** records and retrieved **146,233**; **3** were truncated by a retrieval cap. Query strings are reproduced verbatim as sent to each API. Times are UTC.

PubMed — 9 queries, 5,013 matched, 5,006 retrieved

Query name	Executed (UTC)	Matched	Retrieved	Query string
pa-dose-mortality-1990	2026-08-24T12:32:40 +00:00	344	344	(("physical activity"[tiab] OR "exercise"[tiab] OR "aerobic exercise"[tiab] OR "resistance training"[tiab] OR "muscle-strengthening"[tiab]) AND ("dose-response"[tiab] OR "minimal dose"[tiab] OR "minimum dose"[tiab] OR "minimum effective dose"[tiab] OR "weekly volume"[tiab] OR "MET-minutes"[tiab]) AND ("all-cause mortality"[tiab] OR "longevity"[tiab] OR "lifespan"[tiab] OR

Query name	Executed (UTC)	Matched	Retrieved	Query string
				"life expectancy"[tiab] OR "healthspan"[tiab])) AND ("1990/01/01"[dp] : "2026/12/31"[dp])
pa-guidelines-healthspan-1990†	2026-08-24T12:32:50 +00:00	834	829	(("physical activity guidelines"[tiab] OR "exercise guidelines"[tiab] OR "physical activity recommendation*"[tiab] OR "WHO guidelines"[tiab] OR "consensus statement"[tiab]) AND ("physical activity"[tiab] OR "exercise"[tiab]) AND ("healthy ageing"[tiab] OR "healthy aging"[tiab] OR "healthspan"[tiab] OR "longevity"[tiab] OR "mortality"[tiab] OR "older adults"[tiab])) AND ("1990/01/01"[dp] : "2026/12/31"[dp])
protein-longevity-1990	2026-08-24T12:32:57 +00:00	520	520	(("protein intake"[tiab] OR "dietary protein"[tiab] OR "protein consumption"[tiab] OR "protein requirement*"[tiab]) AND ("longevity"[tiab] OR "healthspan"[tiab] OR "lifespan"[tiab] OR "life expectancy"[tiab] OR "all-cause mortality"[tiab] OR "healthy ageing"[tiab] OR "healthy aging"[tiab])) AND ("1990/01/01"[dp] : "2026/12/31"[dp])
protein-plus-training-	2026-08-24T12:33:03 +00:00	300	300	(("resistance training"[tiab] OR "resistance

Query name	Executed (UTC)	Matched	Retrieved	Query string
1990				exercise"[tiab] OR "physical activity"[tiab] OR "exercise"[tiab]) AND ("protein supplementation"[tiab] OR "protein intake"[tiab] OR "dietary protein"[tiab]) AND ("combined"[tiab] OR "adjunct"[tiab] OR "synerg*"[tiab] OR "multimodal"[tiab] OR "co-ingestion"[tiab] OR "interaction"[tiab]) AND ("older adult*"[tiab] OR "ageing"[tiab] OR "aging"[tiab] OR "sarcopenia"[tiab] OR "healthspan"[tiab])) AND ("1990/01/01"[dp] : "2026/12/31"[dp])
protein-restriction-mtor-1990	2026-08-24T12:33:35 +00:00	1,577	1,577	(("protein restriction"[tiab] OR "low-protein diet"[tiab] OR "mTOR"[tiab] OR "IGF-1"[tiab] OR "amino acid restriction"[tiab] OR "methionine restriction"[tiab]) AND ("longevity"[tiab] OR "lifespan"[tiab] OR "healthspan"[tiab] OR "ageing"[tiab] OR "aging"[tiab]) AND ("human*"[tiab] OR "older adult*"[tiab] OR "protein intake"[tiab] OR "dietary protein"[tiab])) AND ("1990/01/01"[dp] : "2026/12/31"[dp])
protein-sarcopenia-dose-1990†	2026-08-24T12:33:43 +00:00	564	563	(("protein intake"[tiab] OR "dietary protein"[tiab] OR "protein supplementation"[tiab]

Query name	Executed (UTC)	Matched	Retrieved	Query string
				OR "leucine"[tiab] OR "essential amino acid*"[tiab]) AND ("sarcopenia"[tiab] OR "muscle mass"[tiab] OR "lean mass"[tiab] OR "muscle protein synthesis"[tiab] OR "frailty"[tiab]) AND ("older adult*"[tiab] OR "elderly"[tiab] OR "ageing"[tiab] OR "aging"[tiab]) AND ("recommendation*"[tiab] OR "requirement*"[tiab] OR "optimal"[tiab] OR "g/kg"[tiab] OR "dose"[tiab] OR "distribution"[tiab])) AND ("1990/01/01"[dp] : "2026/12/31"[dp])
protein-ukbiobank-1990	2026-08-24T12:33:45 +00:00	132	132	(("UK Biobank"[tiab] OR "NHANES"[tiab] OR "prospective cohort"[tiab]) AND ("protein intake"[tiab] OR "dietary protein"[tiab]) AND ("mortality"[tiab] OR "cardiovascular"[tiab])) AND ("1990/01/01"[dp] : "2026/12/31"[dp])
sport-nutrition-longevity-1990	2026-08-24T12:33:58 +00:00	556	556	(("sports nutrition"[tiab] OR "sport nutrition"[tiab] OR "athlete*"[tiab] OR "masters athlete*"[tiab] OR "exercise nutrition"[tiab]) AND ("longevity"[tiab] OR "healthspan"[tiab] OR "lifespan"[tiab] OR "healthy ageing"[tiab] OR "healthy aging"[tiab] OR "biological age"[tiab])) AND ("1990/01/01"[dp] :

Query name	Executed (UTC)	Matched	Retrieved	Query string
				"2026/12/31"[dp])
steps-vilpa-mortality-1990†	2026-08-24T12:34:00 +00:00	186	185	(("steps per day"[tiab] OR "step count"[tiab] OR "daily steps"[tiab] OR "accelerometer-measured"[tiab] OR "device-measured physical activity"[tiab] OR "vigorous intermittent lifestyle physical activity"[tiab] OR "VILPA"[tiab]) AND ("all-cause mortality"[tiab] OR "longevity"[tiab] OR "life expectancy"[tiab] OR "healthspan"[tiab] OR "cardiovascular mortality"[tiab])) AND ("1990/01/01"[dp] : "2026/12/31"[dp])

Europe PMC — 67 queries, 36,285 matched, 36,287 retrieved

In two rows (`mb-exercise`, `mb-protein`) the number of records retrieved exceeds the match count the API reported for the same query by one. Both figures are logged as returned at execution time and are printed here unaltered; the discrepancy is a property of the live index, which changed between the count call and the final page of results.

Query name	Executed (UTC)	Matched	Retrieved	Query string
ai-diet-assessment	2026-08-24T11:54:43 +00:00	1,046	1,046	((TITLE_ABS:"artificial intelligence" OR TITLE_ABS:"machine learning" OR TITLE_ABS:"deep learning" OR TITLE_ABS:"computer vision" OR TITLE_ABS:"convolutional neural network*" OR TITLE_ABS:"image-based" OR TITLE_ABS:"image based" OR TITLE_ABS:"smartphone"

Query name	Executed (UTC)	Matched	Retrieved	Query string
				OR TITLE_ABS:"large language model*" OR TITLE_ABS:"automated") AND (TITLE_ABS:"dietary assessment" OR TITLE_ABS:"dietary intake" OR TITLE_ABS:"food record*" OR TITLE_ABS:"24-hour recall" OR TITLE_ABS:"food frequency questionnaire" OR TITLE_ABS:"food photograph*" OR TITLE_ABS:"portion size" OR TITLE_ABS:"food recognition" OR TITLE_ABS:"nutrient estimation" OR TITLE_ABS:"energy intake")) AND (FIRST_PDATE:[2018-01-01 TO 2026-12-31])
ai-diet-validation	2026-08-24T11:54:45 +00:00	383	383	((TITLE_ABS:"artificial intelligence" OR TITLE_ABS:"machine learning" OR TITLE_ABS:"deep learning" OR TITLE_ABS:"computer vision" OR TITLE_ABS:"convolutional neural network*" OR TITLE_ABS:"image-based" OR TITLE_ABS:"image based" OR TITLE_ABS:"smartphone" OR TITLE_ABS:"large language model*" OR TITLE_ABS:"automated") AND (TITLE_ABS:"dietary assessment" OR TITLE_ABS:"dietary intake" OR TITLE_ABS:"food record*" OR TITLE_ABS:"24-hour recall" OR

Query name	Executed (UTC)	Matched	Retrieved	Query string
				TITLE_ABS:"food frequency questionnaire" OR TITLE_ABS:"food photograph*" OR TITLE_ABS:"portion size" OR TITLE_ABS:"food recognition" OR TITLE_ABS:"nutrient estimation" OR TITLE_ABS:"energy intake") AND (TITLE_ABS:"validity" OR TITLE_ABS:"validation" OR TITLE_ABS:"accuracy" OR TITLE_ABS:"agreement" OR TITLE_ABS:"reliability" OR TITLE_ABS:"weighed food record" OR TITLE_ABS:"doubly labelled water" OR TITLE_ABS:"doubly labeled water")) AND (FIRST_PDATE:[2018-01-01 TO 2026-12-31])
ai-protein-monitoring	2026-08-24T11:54:46 +00:00	98	98	((TITLE_ABS:"artificial intelligence" OR TITLE_ABS:"machine learning" OR TITLE_ABS:"deep learning" OR TITLE_ABS:"computer vision" OR TITLE_ABS:"convolutional neural network*" OR TITLE_ABS:"image-based" OR TITLE_ABS:"image based" OR TITLE_ABS:"smartphone" OR TITLE_ABS:"large language model*" OR TITLE_ABS:"automated") AND (TITLE_ABS:"protein intake" OR TITLE_ABS:"dietary protein" OR TITLE_ABS:"macronutrient

Query name	Executed (UTC)	Matched	Retrieved	Query string
				*") AND (TITLE_ABS:"assessment" OR TITLE_ABS:"monitoring" OR TITLE_ABS:"tracking" OR TITLE_ABS:"estimation")) AND (FIRST_PDATE:[2018-01-01 TO 2026-12-31])
cardiovascular	2026-08-24T11:54:48 +00:00	115	115	((TITLE_ABS:"protein intake" OR TITLE_ABS:"dietary protein") AND (TITLE_ABS:"heart failure" OR TITLE_ABS:"coronary" OR TITLE_ABS:"myocardial" OR TITLE_ABS:"stroke" OR TITLE_ABS:"cardiovascular disease" OR TITLE_ABS:"cardiac cachexia") AND (TITLE_ABS:"mortality" OR TITLE_ABS:"prognosis" OR TITLE_ABS:"survival" OR TITLE_ABS:"g/kg" OR TITLE_ABS:"protein requirement*")) AND (FIRST_PDATE:[2018-01-01 TO 2026-12-31])
ckd	2026-08-24T11:54:50 +00:00	92	92	((TITLE_ABS:"protein intake" OR TITLE_ABS:"dietary protein" OR TITLE_ABS:"low-protein diet") AND (TITLE_ABS:"g/kg" OR TITLE_ABS:"g per kg" OR TITLE_ABS:"protein requirement*" OR TITLE_ABS:"optimal protein" OR TITLE_ABS:"protein target" OR TITLE_ABS:"protein dose") AND (TITLE_ABS:"chronic

Query name	Executed (UTC)	Matched	Retrieved	Query string
				kidney disease" OR TITLE_ABS:"CKD" OR TITLE_ABS:"dialysis" OR TITLE_ABS:"eGFR" OR TITLE_ABS:"renal") AND (TITLE_ABS:"mortality" OR TITLE_ABS:"progression" OR TITLE_ABS:"survival")) AND (FIRST_PDATE:[2018-01-01 TO 2026-12-31])
diabetes	2026-08-24T11:54:51 +00:00	22	22	((TITLE_ABS:"protein intake" OR TITLE_ABS:"dietary protein") AND (TITLE_ABS:"g/kg" OR TITLE_ABS:"g per kg" OR TITLE_ABS:"grams per kilogram" OR TITLE_ABS:"protein requirement*" OR TITLE_ABS:"optimal protein" OR TITLE_ABS:"protein target" OR TITLE_ABS:"protein dose") AND (TITLE_ABS:"type 2 diabetes" OR TITLE_ABS:"diabetic" OR TITLE_ABS:"diabetes mellitus") AND (TITLE_ABS:"mortality" OR TITLE_ABS:"nephropathy" OR TITLE_ABS:"glycemic" OR TITLE_ABS:"outcome*")) AND (FIRST_PDATE:[2018-01-01 TO 2026-12-31])
diabetes2	2026-08-24T11:54:58 +00:00	913	913	((TITLE_ABS:"protein intake" OR TITLE_ABS:"dietary protein" OR TITLE_ABS:"high-protein diet" OR

Query name	Executed (UTC)	Matched	Retrieved	Query string
				TITLE_ABS:"protein supplementation") AND (TITLE_ABS:"type 2 diabetes" OR TITLE_ABS:"diabetes mellitus" OR TITLE_ABS:"diabetic" OR TITLE_ABS:"insulin resistance" OR TITLE_ABS:"glycaemic" OR TITLE_ABS:"glycemic" OR TITLE_ABS:"HbA1c")) AND (FIRST_PDATE:[2015-01-01 TO 2026-12-31])
glp1-guidance	2026-08-24T11:54:59 +00:00	91	91	((TITLE_ABS:"GLP-1" OR TITLE_ABS:"GLP1" OR TITLE_ABS:"glucagon-like peptide-1" OR TITLE_ABS:"glucagon like peptide 1" OR TITLE_ABS:"incretin" OR TITLE_ABS:"semaglutide" OR TITLE_ABS:"tirzepatide" OR TITLE_ABS:"liraglutide" OR TITLE_ABS:"dulaglutide" OR TITLE_ABS:"exenatide" OR TITLE_ABS:"retatrutide" OR TITLE_ABS:"GIP/GLP-1") AND (TITLE_ABS:"consensus" OR TITLE_ABS:"position statement" OR TITLE_ABS:"guideline*" OR TITLE_ABS:"recommendation*" OR TITLE_ABS:"practical guidance" OR TITLE_ABS:"expert opinion") AND (TITLE_ABS:"nutrition" OR TITLE_ABS:"protein" OR TITLE_ABS:"muscle" OR

Query name	Executed (UTC)	Matched	Retrieved	Query string
				TITLE_ABS:"lean mass" OR TITLE_ABS:"sarcopenia")) AND (FIRST_PDATE:[2015-01-01 TO 2026-12-31])
glp1-leanmass	2026-08-24T11:55:10+00:00	1,130	1,130	((TITLE_ABS:"GLP-1" OR TITLE_ABS:"GLP1" OR TITLE_ABS:"glucagon-like peptide-1" OR TITLE_ABS:"glucagon like peptide 1" OR TITLE_ABS:"incretin" OR TITLE_ABS:"semaglutide" OR TITLE_ABS:"tirzepatide" OR TITLE_ABS:"liraglutide" OR TITLE_ABS:"dulaglutide" OR TITLE_ABS:"exenatide" OR TITLE_ABS:"retatrutide" OR TITLE_ABS:"GIP/GLP-1") AND (TITLE_ABS:"lean mass" OR TITLE_ABS:"fat-free mass" OR TITLE_ABS:"fat free mass" OR TITLE_ABS:"muscle mass" OR TITLE_ABS:"skeletal muscle" OR TITLE_ABS:"sarcopenia" OR TITLE_ABS:"sarcopenic obesity" OR TITLE_ABS:"muscle strength" OR TITLE_ABS:"grip strength" OR TITLE_ABS:"body composition" OR TITLE_ABS:"appendicular" OR TITLE_ABS:"muscle loss")) AND (FIRST_PDATE:[2015-01-01 TO 2026-12-31])
glp1-preservation	2026-08-24T11:55:12	116	116	(((TITLE_ABS:"GLP-1" OR TITLE_ABS:"GLP1" OR TITLE_ABS:"glucagon-like

Query name	Executed (UTC)	Matched	Retrieved	Query string
	+00:00			peptide-1" OR TITLE_ABS:"glucagon like peptide 1" OR TITLE_ABS:"incretin" OR TITLE_ABS:"semaglutide" OR TITLE_ABS:"tirzepatide" OR TITLE_ABS:"liraglutide" OR TITLE_ABS:"dulaglutide" OR TITLE_ABS:"exenatide" OR TITLE_ABS:"retatrutide" OR TITLE_ABS:"GIP/GLP-1") OR TITLE_ABS:"weight loss" OR TITLE_ABS:"caloric restriction" OR TITLE_ABS:"bariatric") AND (TITLE_ABS:"muscle-preserving" OR TITLE_ABS:"muscle preservation" OR TITLE_ABS:"preserve lean" OR TITLE_ABS:"preserving lean" OR TITLE_ABS:"iatrogenic sarcopenia" OR TITLE_ABS:"sarcopenic obesity") AND (TITLE_ABS:"protein" OR TITLE_ABS:"resistance training")) AND (FIRST_PDATE:[2015-01-01 TO 2026-12-31])
glp1-protein	2026-08-24T11:55:16 +00:00	386	386	((TITLE_ABS:"GLP-1" OR TITLE_ABS:"GLP1" OR TITLE_ABS:"glucagon-like peptide-1" OR TITLE_ABS:"glucagon like peptide 1" OR TITLE_ABS:"incretin" OR TITLE_ABS:"semaglutide" OR TITLE_ABS:"tirzepatide"

Query name	Executed (UTC)	Matched	Retrieved	Query string
				OR TITLE_ABS:"liraglutide" OR TITLE_ABS:"dulaglutide" OR TITLE_ABS:"exenatide" OR TITLE_ABS:"retatrutide" OR TITLE_ABS:"GIP/GLP-1") AND (TITLE_ABS:"protein intake" OR TITLE_ABS:"dietary protein" OR TITLE_ABS:"protein supplementation" OR TITLE_ABS:"high-protein" OR TITLE_ABS:"whey protein" OR TITLE_ABS:"leucine" OR TITLE_ABS:"essential amino acid*" OR TITLE_ABS:"protein requirement*" OR TITLE_ABS:"g/kg")) AND (FIRST_PDATE:[2015-01-01 TO 2026-12-31])
glp1-randomised	2026-08-24T11:55:18 +00:00	263	263	((TITLE_ABS:"GLP-1" OR TITLE_ABS:"GLP1" OR TITLE_ABS:"glucagon-like peptide-1" OR TITLE_ABS:"glucagon like peptide 1" OR TITLE_ABS:"incretin" OR TITLE_ABS:"semaglutide" OR TITLE_ABS:"tirzepatide" OR TITLE_ABS:"liraglutide" OR TITLE_ABS:"dulaglutide" OR TITLE_ABS:"exenatide" OR TITLE_ABS:"retatrutide" OR TITLE_ABS:"GIP/GLP-1") AND (TITLE_ABS:"lean mass" OR TITLE_ABS:"fat-free mass" OR

Query name	Executed (UTC)	Matched	Retrieved	Query string
				TITLE_ABS:"fat free mass" OR TITLE_ABS:"muscle mass" OR TITLE_ABS:"skeletal muscle" OR TITLE_ABS:"sarcopenia" OR TITLE_ABS:"sarcopenic obesity" OR TITLE_ABS:"muscle strength" OR TITLE_ABS:"grip strength" OR TITLE_ABS:"body composition" OR TITLE_ABS:"appendicular" OR TITLE_ABS:"muscle loss") AND (PUB_TYPE:"randomized controlled trial" OR TITLE_ABS:"randomized" OR TITLE_ABS:"randomised" OR TITLE_ABS:"placebo" OR PUB_TYPE:"meta-analysis" OR PUB_TYPE:"systematic review")) AND (FIRST_PDATE:[2015-01-01 TO 2026-12-31])
glp1-training	2026-08-24T11:55:19 +00:00	146	146	((TITLE_ABS:"GLP-1" OR TITLE_ABS:"GLP1" OR TITLE_ABS:"glucagon-like peptide-1" OR TITLE_ABS:"glucagon like peptide 1" OR TITLE_ABS:"incretin" OR TITLE_ABS:"semaglutide" OR TITLE_ABS:"tirzepatide" OR TITLE_ABS:"liraglutide" OR TITLE_ABS:"dulaglutide" OR TITLE_ABS:"exenatide" OR TITLE_ABS:"retatrutide" OR TITLE_ABS:"GIP/GLP-

Query name	Executed (UTC)	Matched	Retrieved	Query string
				1") AND (TITLE_ABS:"resistance training" OR TITLE_ABS:"resistance exercise" OR TITLE_ABS:"strength training" OR TITLE_ABS:"exercise training" OR TITLE_ABS:"physical activity") AND (TITLE_ABS:"lean mass" OR TITLE_ABS:"fat-free mass" OR TITLE_ABS:"fat free mass" OR TITLE_ABS:"muscle mass" OR TITLE_ABS:"skeletal muscle" OR TITLE_ABS:"sarcopenia" OR TITLE_ABS:"sarcopenic obesity" OR TITLE_ABS:"muscle strength" OR TITLE_ABS:"grip strength" OR TITLE_ABS:"body composition" OR TITLE_ABS:"appendicular" OR TITLE_ABS:"muscle loss")) AND (FIRST_PDATE:[2015-01-01 TO 2026-12-31])
healthy	2026-08-24T11:55:20 +00:00	63	63	((TITLE_ABS:"protein intake" OR TITLE_ABS:"dietary protein") AND (TITLE_ABS:"g/kg" OR TITLE_ABS:"g per kg" OR TITLE_ABS:"grams per kilogram" OR TITLE_ABS:"protein requirement*" OR TITLE_ABS:"optimal protein" OR TITLE_ABS:"protein target" OR TITLE_ABS:"protein

Query name	Executed (UTC)	Matched	Retrieved	Query string
				dose") AND (TITLE_ABS:"healthy adult*" OR TITLE_ABS:"general population" OR TITLE_ABS:"community-dwelling" OR TITLE_ABS:"healthy older") AND (TITLE_ABS:"mortality" OR TITLE_ABS:"sarcopenia" OR TITLE_ABS:"muscle mass" OR TITLE_ABS:"healthspan" OR TITLE_ABS:"requirement*")) AND (FIRST_PDATE:[2018-01-01 TO 2026-12-31])
highprotein-safety-cvd	2026-08-24T11:55:22+00:00	117	117	((TITLE_ABS:"high-protein diet" OR TITLE_ABS:"high protein intake" OR TITLE_ABS:"protein overconsumption") AND (TITLE_ABS:"cardiovascular" OR TITLE_ABS:"mortality" OR TITLE_ABS:"safety" OR TITLE_ABS:"renal" OR TITLE_ABS:"kidney") AND (TITLE_ABS:"randomized" OR TITLE_ABS:"trial" OR TITLE_ABS:"cohort" OR TITLE_ABS:"meta-analysis")) AND (FIRST_PDATE:[2015-01-01 TO 2026-12-31])
highprotein-upper	2026-08-24T11:55:29+00:00	1,010	1,010	((TITLE_ABS:"high-protein diet" OR TITLE_ABS:"high protein intake" OR TITLE_ABS:"1.6 g/kg" OR TITLE_ABS:"2.0 g/kg" OR TITLE_ABS:"2.2 g/kg" OR TITLE_ABS:"upper limit"

Query name	Executed (UTC)	Matched	Retrieved	Query string
				OR TITLE_ABS:"safe upper" OR TITLE_ABS:"tolerable upper intake") AND (TITLE_ABS:"protein") AND (TITLE_ABS:"safety" OR TITLE_ABS:"renal function" OR TITLE_ABS:"kidney function" OR TITLE_ABS:"adverse" OR TITLE_ABS:"upper limit" OR TITLE_ABS:"randomized" OR TITLE_ABS:"randomised")) AND (FIRST_PDATE:[2015-01-01 TO 2026-12-31])
hyp-athlete-req	2026-08-24T11:55:31+00:00	41	41	((TITLE_ABS:"athlete*" OR TITLE_ABS:"resistance-trained" OR TITLE_ABS:"resistance trained" OR TITLE_ABS:"bodybuilder*" OR TITLE_ABS:"strength athlete*") AND (TITLE_ABS:"protein requirement*" OR TITLE_ABS:"protein intake" OR TITLE_ABS:"protein recommendation*" OR TITLE_ABS:"g/kg") AND (TITLE_ABS:"position stand" OR TITLE_ABS:"consensus" OR TITLE_ABS:"recommendation*" OR TITLE_ABS:"requirement*" OR TITLE_ABS:"hypertrophy" OR TITLE_ABS:"lean mass")) AND (FIRST_PDATE:[2010-01-01 TO 2026-12-31])

Query name	Executed (UTC)	Matched	Retrieved	Query string
hyp-dose-response	2026-08-24T11:55:33 +00:00	170	170	((TITLE_ABS:"protein supplementation" OR TITLE_ABS:"protein intake" OR TITLE_ABS:"dietary protein") AND (TITLE_ABS:"resistance training" OR TITLE_ABS:"resistance exercise" OR TITLE_ABS:"strength training") AND (TITLE_ABS:"dose-response" OR TITLE_ABS:"plateau" OR TITLE_ABS:"breakpoint" OR TITLE_ABS:"optimal" OR TITLE_ABS:"g/kg" OR TITLE_ABS:"threshold") AND (TITLE_ABS:"fat-free mass" OR TITLE_ABS:"lean body mass" OR TITLE_ABS:"muscle mass" OR TITLE_ABS:"hypertrophy" OR TITLE_ABS:"1RM" OR TITLE_ABS:"strength")) AND (FIRST_PDATE:[2010-01-01 TO 2026-12-31])
hyp-iaao-nbal	2026-08-24T11:55:34 +00:00	75	75	((TITLE_ABS:"indicator amino acid oxidation" OR TITLE_ABS:"nitrogen balance" OR TITLE_ABS:"protein requirement*") AND (TITLE_ABS:"resistance training" OR TITLE_ABS:"athlete*" OR TITLE_ABS:"exercise" OR TITLE_ABS:"endurance")) AND (FIRST_PDATE:[2010-01-01 TO 2026-12-31])
hyp-veryhigh	2026-08-24T11:55:36 +00:00	284	284	((TITLE_ABS:"high protein" OR TITLE_ABS:"2 g/kg" OR TITLE_ABS:"2.2 g/kg" OR TITLE_ABS:"3 g/kg" OR

Query name	Executed (UTC)	Matched	Retrieved	Query string
				TITLE_ABS:"supraphysiological" OR TITLE_ABS:"very high protein") AND (TITLE_ABS:"resistance training" OR TITLE_ABS:"body composition" OR TITLE_ABS:"lean mass") AND (TITLE_ABS:"randomized" OR TITLE_ABS:"randomised" OR TITLE_ABS:"trial")) AND (FIRST_PDATE:[2010-01-01 TO 2026-12-31])
mb-exercise	2026-08-24T11:56:32+00:00	7,530	7,531	((TITLE_ABS:"gut microbiota" OR TITLE_ABS:"gut microbiome" OR TITLE_ABS:"intestinal microbiota" OR TITLE_ABS:"microbiome" OR TITLE_ABS:"microbiota" OR TITLE_ABS:"short-chain fatty acid*" OR TITLE_ABS:"SCFA" OR TITLE_ABS:"butyrate" OR TITLE_ABS:"propionate" OR TITLE_ABS:"faecal microbiota" OR TITLE_ABS:"fecal microbiota") AND (TITLE_ABS:"exercise" OR TITLE_ABS:"physical activity" OR TITLE_ABS:"resistance training" OR TITLE_ABS:"endurance training" OR TITLE_ABS:"athlete*" OR TITLE_ABS:"lactate" OR TITLE_ABS:"Veillonella")) AND (FIRST_PDATE:[2015-01-01 TO 2026-12-31])

Query name	Executed (UTC)	Matched	Retrieved	Query string
mb-gutmuscle-axis	2026-08-24T11:56:35 +00:00	663	663	((TITLE_ABS:"gut-muscle axis" OR TITLE_ABS:"gut muscle axis" OR TITLE_ABS:"microbiota-gut-muscle" OR TITLE_ABS:"muscle-gut") OR ((TITLE_ABS:"gut microbiota" OR TITLE_ABS:"gut microbiome" OR TITLE_ABS:"intestinal microbiota" OR TITLE_ABS:"microbiome" OR TITLE_ABS:"microbiota" OR TITLE_ABS:"short-chain fatty acid*" OR TITLE_ABS:"SCFA" OR TITLE_ABS:"butyrate" OR TITLE_ABS:"propionate" OR TITLE_ABS:"faecal microbiota" OR TITLE_ABS:"fecal microbiota") AND (TITLE_ABS:"sarcopenia" OR TITLE_ABS:"muscle mass" OR TITLE_ABS:"skeletal muscle" OR TITLE_ABS:"muscle strength" OR TITLE_ABS:"grip strength" OR TITLE_ABS:"physical function" OR TITLE_ABS:"frailty" OR TITLE_ABS:"lean mass" OR TITLE_ABS:"muscle protein synthesis") AND (TITLE_ABS:"randomized" OR TITLE_ABS:"randomised" OR TITLE_ABS:"probiotic*" OR TITLE_ABS:"prebiotic*" OR

Query name	Executed (UTC)	Matched	Retrieved	Query string
				TITLE_ABS:"synbiotic*" OR TITLE_ABS:"fibre" OR TITLE_ABS:"fiber"))) AND (FIRST_PDATE:[2015-01-01 TO 2026-12-31])
mb-longevity	2026-08-24T11:56:45 +00:00	1,330	1,330	((TITLE_ABS:"gut microbiota" OR TITLE_ABS:"gut microbiome" OR TITLE_ABS:"intestinal microbiota" OR TITLE_ABS:"microbiome" OR TITLE_ABS:"microbiota" OR TITLE_ABS:"short-chain fatty acid*" OR TITLE_ABS:"SCFA" OR TITLE_ABS:"butyrate" OR TITLE_ABS:"propionate" OR TITLE_ABS:"faecal microbiota" OR TITLE_ABS:"fecal microbiota") AND (TITLE_ABS:"longevity" OR TITLE_ABS:"healthspan" OR TITLE_ABS:"ageing" OR TITLE_ABS:"aging" OR TITLE_ABS:"all-cause mortality" OR TITLE_ABS:"centenarian*") AND (TITLE_ABS:"human*" OR TITLE_ABS:"older adult*" OR TITLE_ABS:"cohort")) AND (FIRST_PDATE:[2015-01-01 TO 2026-12-31])
mb-muscle	2026-08-24T11:56:54 +00:00	1,838	1,838	((TITLE_ABS:"gut microbiota" OR TITLE_ABS:"gut microbiome" OR TITLE_ABS:"intestinal microbiota" OR TITLE_ABS:"microbiome" OR TITLE_ABS:"microbiota" OR TITLE_ABS:"short-

Query name	Executed (UTC)	Matched	Retrieved	Query string
				chain fatty acid*" OR TITLE_ABS:"SCFA" OR TITLE_ABS:"butyrate" OR TITLE_ABS:"propionate" OR TITLE_ABS:"faecal microbiota" OR TITLE_ABS:"fecal microbiota") AND (TITLE_ABS:"sarcopenia" OR TITLE_ABS:"muscle mass" OR TITLE_ABS:"skeletal muscle" OR TITLE_ABS:"muscle strength" OR TITLE_ABS:"grip strength" OR TITLE_ABS:"physical function" OR TITLE_ABS:"frailty" OR TITLE_ABS:"lean mass" OR TITLE_ABS:"muscle protein synthesis")) AND (FIRST_PDATE:[2015-01-01 TO 2026-12-31])
mb-protein	2026-08-24T11:58:13 +00:00	9,667	9,668	((TITLE_ABS:"gut microbiota" OR TITLE_ABS:"gut microbiome" OR TITLE_ABS:"intestinal microbiota" OR TITLE_ABS:"microbiome" OR TITLE_ABS:"microbiota" OR TITLE_ABS:"short-chain fatty acid*" OR TITLE_ABS:"SCFA" OR TITLE_ABS:"butyrate" OR TITLE_ABS:"propionate" OR TITLE_ABS:"faecal microbiota" OR TITLE_ABS:"fecal microbiota") AND (TITLE_ABS:"dietary protein" OR TITLE_ABS:"protein intake" OR

Query name	Executed (UTC)	Matched	Retrieved	Query string
				TITLE_ABS:"protein fermentation" OR TITLE_ABS:"plant protein" OR TITLE_ABS:"animal protein" OR TITLE_ABS:"amino acid" OR TITLE_ABS:"putrefaction" OR TITLE_ABS:"p-cresol" OR TITLE_ABS:"indoxyl sulfate" OR TITLE_ABS:"TMAO" OR TITLE_ABS:"trimethylamine")) AND (FIRST_PDATE:[2015-01-01 TO 2026-12-31])
med-grip-mortality	2026-08-24T11:58:17 +00:00	568	568	((TITLE_ABS:"handgrip strength" OR TITLE_ABS:"grip strength" OR TITLE_ABS:"muscle strength") AND (TITLE_ABS:"all-cause mortality" OR TITLE_ABS:"mortality") AND (PUB_TYPE:"meta-analysis" OR PUB_TYPE:"systematic review" OR TITLE_ABS:"dose-response" OR TITLE_ABS:"per 5 kg" OR TITLE_ABS:"per kg" OR TITLE_ABS:"hazard ratio")) AND (FIRST_PDATE:[2010-01-01 TO 2026-12-31])
med-musclemass-mortality	2026-08-24T11:54:26 +00:00	73	73	((TITLE_ABS:"muscle mass" OR TITLE_ABS:"lean body mass" OR TITLE_ABS:"fat-free mass" OR TITLE_ABS:"skeletal muscle index") AND (TITLE_ABS:"mortality" OR TITLE_ABS:"survival")

Query name	Executed (UTC)	Matched	Retrieved	Query string
				AND (TITLE_ABS:"cohort" OR TITLE_ABS:"prospective") AND (PUB_TYPE:"meta-analysis" OR TITLE_ABS:"dose-response")) AND (FIRST_PDATE:[2010-01-01 TO 2026-12-31])
med-sarcopenia-mortality	2026-08-24T11:58:20 +00:00	238	238	((TITLE_ABS:"sarcopenia" OR TITLE_ABS:"low muscle mass" OR TITLE_ABS:"appendicular lean mass" OR TITLE_ABS:"muscle wasting") AND (TITLE_ABS:"all-cause mortality" OR TITLE_ABS:"cardiovascular mortality" OR TITLE_ABS:"major adverse cardiovascular" OR TITLE_ABS:"MACE") AND (PUB_TYPE:"meta-analysis" OR PUB_TYPE:"systematic review" OR TITLE_ABS:"pooled" OR TITLE_ABS:"hazard ratio")) AND (FIRST_PDATE:[2010-01-01 TO 2026-12-31])
obesity	2026-08-24T11:58:21 +00:00	83	83	((TITLE_ABS:"protein intake" OR TITLE_ABS:"dietary protein" OR TITLE_ABS:"high-protein diet") AND (TITLE_ABS:"g/kg" OR TITLE_ABS:"g per kg" OR TITLE_ABS:"grams per kilogram" OR TITLE_ABS:"protein requirement*" OR TITLE_ABS:"optimal protein" OR TITLE_ABS:"protein

Query name	Executed (UTC)	Matched	Retrieved	Query string
				target" OR TITLE_ABS:"protein dose") AND (TITLE_ABS:"obesity" OR TITLE_ABS:"obese" OR TITLE_ABS:"weight loss" OR TITLE_ABS:"bariatric" OR TITLE_ABS:"sarcopenic obesity") AND (TITLE_ABS:"mortality" OR TITLE_ABS:"lean mass" OR TITLE_ABS:"muscle" OR TITLE_ABS:"outcome*")) AND (FIRST_PDATE:[2018-01-01 TO 2026-12-31])
pa-clinical-populations	2026-08-24T11:58:23 +00:00	72	72	((TITLE_ABS:"physical activity" OR TITLE_ABS:"exercise") AND (TITLE_ABS:"cancer survivor*" OR TITLE_ABS:"coronary" OR TITLE_ABS:"heart failure" OR TITLE_ABS:"chronic kidney disease" OR TITLE_ABS:"COPD" OR TITLE_ABS:"type 2 diabetes" OR TITLE_ABS:"stroke" OR TITLE_ABS:"hypertension") AND (TITLE_ABS:"meeting" OR TITLE_ABS:"guideline*" OR TITLE_ABS:"recommend*" OR TITLE_ABS:"active vs" OR TITLE_ABS:"inactive") AND (TITLE_ABS:"all-cause mortality" OR TITLE_ABS:"all cause mortality" OR TITLE_ABS:"total mortality") AND (TITLE_ABS:"prospective cohort" OR TITLE_ABS:"cohort study" OR

Query name	Executed (UTC)	Matched	Retrieved	Query string
				TITLE_ABS:"prospective study" OR TITLE_ABS:"follow-up study" OR TITLE_ABS:"hazard ratio*")) AND (FIRST_PDATE:[2010-01-01 TO 2026-12-31])
pa-device-measured	2026-08-24T11:58:25 +00:00	150	150	((TITLE_ABS:"accelerometer" OR TITLE_ABS:"accelerometry" OR TITLE_ABS:"device-measured" OR TITLE_ABS:"device measured" OR TITLE_ABS:"wrist-worn" OR TITLE_ABS:"wearable" OR TITLE_ABS:"actigraph*") AND (TITLE_ABS:"physical activity" OR TITLE_ABS:"MVPA" OR TITLE_ABS:"steps") AND (TITLE_ABS:"all-cause mortality" OR TITLE_ABS:"all cause mortality" OR TITLE_ABS:"total mortality") AND (TITLE_ABS:"prospective cohort" OR TITLE_ABS:"cohort study" OR TITLE_ABS:"prospective study" OR TITLE_ABS:"follow-up study" OR TITLE_ABS:"hazard ratio*")) AND (FIRST_PDATE:[2010-01-01 TO 2026-12-31])
pa-dose-mortality	2026-08-24T11:58:27 +00:00	173	173	((TITLE_ABS:"physical activity" OR TITLE_ABS:"exercise" OR TITLE_ABS:"aerobic exercise" OR TITLE_ABS:"resistance

Query name	Executed (UTC)	Matched	Retrieved	Query string
				training" OR TITLE_ABS:"muscle-strengthening") AND (TITLE_ABS:"dose-response" OR TITLE_ABS:"minimal dose" OR TITLE_ABS:"minimum dose" OR TITLE_ABS:"minimum effective dose" OR TITLE_ABS:"weekly volume" OR TITLE_ABS:"MET-minutes") AND (TITLE_ABS:"all-cause mortality" OR TITLE_ABS:"longevity" OR TITLE_ABS:"lifespan" OR TITLE_ABS:"life expectancy" OR TITLE_ABS:"healthspan")) AND (FIRST_PDATE:[2023-01-01 TO 2026-12-31])
pa-dose-mortality-1990	2026-08-24T12:35:49 +00:00	342	342	((TITLE_ABS:"physical activity" OR TITLE_ABS:"exercise" OR TITLE_ABS:"aerobic exercise" OR TITLE_ABS:"resistance training" OR TITLE_ABS:"muscle-strengthening") AND (TITLE_ABS:"dose-response" OR TITLE_ABS:"minimal dose" OR TITLE_ABS:"minimum dose" OR TITLE_ABS:"minimum effective dose" OR TITLE_ABS:"weekly volume" OR TITLE_ABS:"MET-minutes") AND (TITLE_ABS:"all-cause mortality" OR TITLE_ABS:"longevity" OR TITLE_ABS:"lifespan" OR TITLE_ABS:"life expectancy" OR

Query name	Executed (UTC)	Matched	Retrieved	Query string
				TITLE_ABS:"healthspan")) AND (FIRST_PDATE:[1990-01-01 TO 2026-12-31])
pa-guideline-adherence	2026-08-24T11:58:29 +00:00	94	94	((TITLE_ABS:"physical activity guideline*" OR TITLE_ABS:"meeting the guidelines" OR TITLE_ABS:"guideline-concordant" OR TITLE_ABS:"guideline adherence" OR TITLE_ABS:"WHO recommendation*" OR TITLE_ABS:"150 min" OR TITLE_ABS:"150 minutes" OR TITLE_ABS:"500-1000 MET" OR TITLE_ABS:"600 MET") AND (TITLE_ABS:"physical activity" OR TITLE_ABS:"exercise" OR TITLE_ABS:"MVPA") AND (TITLE_ABS:"all-cause mortality" OR TITLE_ABS:"all cause mortality" OR TITLE_ABS:"total mortality") AND (TITLE_ABS:"prospective cohort" OR TITLE_ABS:"cohort study" OR TITLE_ABS:"prospective study" OR TITLE_ABS:"follow-up study" OR TITLE_ABS:"hazard ratio*")) AND (FIRST_PDATE:[2010-01-01 TO 2026-12-31])
pa-guidelines-healthspan	2026-08-24T11:58:30 +00:00	228	228	((TITLE_ABS:"physical activity guidelines" OR TITLE_ABS:"exercise guidelines" OR TITLE_ABS:"physical activity recommendation*" OR

Query name	Executed (UTC)	Matched	Retrieved	Query string
				TITLE_ABS:"WHO guidelines" OR TITLE_ABS:"consensus statement") AND (TITLE_ABS:"physical activity" OR TITLE_ABS:"exercise") AND (TITLE_ABS:"healthy ageing" OR TITLE_ABS:"healthy aging" OR TITLE_ABS:"healthspan" OR TITLE_ABS:"longevity" OR TITLE_ABS:"mortality" OR TITLE_ABS:"older adults")) AND (FIRST_PDATE:[2023-01-01 TO 2026-12-31])
pa-guidelines-healthspan-1990	2026-08-24T12:35:55 +00:00	661	661	((TITLE_ABS:"physical activity guidelines" OR TITLE_ABS:"exercise guidelines" OR TITLE_ABS:"physical activity recommendation*" OR TITLE_ABS:"WHO guidelines" OR TITLE_ABS:"consensus statement") AND (TITLE_ABS:"physical activity" OR TITLE_ABS:"exercise") AND (TITLE_ABS:"healthy ageing" OR TITLE_ABS:"healthy aging" OR TITLE_ABS:"healthspan" OR TITLE_ABS:"longevity" OR TITLE_ABS:"mortality" OR TITLE_ABS:"older adults")) AND (FIRST_PDATE:[1990-01-01 TO 2026-12-31])
pa-pooled-cohorts	2026-08-24T11:58:32 +00:00	72	72	((TITLE_ABS:"pooled analysis" OR TITLE_ABS:"pooled cohort" OR

Query name	Executed (UTC)	Matched	Retrieved	Query string
				TITLE_ABS:"consortium" OR TITLE_ABS:"harmonised" OR TITLE_ABS:"harmonized" OR TITLE_ABS:"individual participant data") AND (TITLE_ABS:"physical activity" OR TITLE_ABS:"exercise") AND (TITLE_ABS:"all-cause mortality" OR TITLE_ABS:"all cause mortality" OR TITLE_ABS:"total mortality")) AND (FIRST_PDATE:[2010-01-01 TO 2026-12-31])
pa-quantile-contrast	2026-08-24T11:58:34 +00:00	127	127	((TITLE_ABS:"leisure-time physical activity" OR TITLE_ABS:"leisure time physical activity" OR TITLE_ABS:"total physical activity" OR TITLE_ABS:"moderate-to-vigorous physical activity" OR TITLE_ABS:"MVPA") AND (TITLE_ABS:"most active" OR TITLE_ABS:"highest quartile" OR TITLE_ABS:"highest quintile" OR TITLE_ABS:"highest tertile" OR TITLE_ABS:"inactive" OR TITLE_ABS:"sedentary" OR TITLE_ABS:"physically inactive") AND (TITLE_ABS:"all-cause mortality" OR TITLE_ABS:"all cause mortality" OR TITLE_ABS:"total mortality") AND (TITLE_ABS:"prospective cohort" OR

Query name	Executed (UTC)	Matched	Retrieved	Query string
				TITLE_ABS:"cohort study" OR TITLE_ABS:"prospective study" OR TITLE_ABS:"follow-up study" OR TITLE_ABS:"hazard ratio*")) AND (FIRST_PDATE:[2010-01-01 TO 2026-12-31])
protein-longevity	2026-08-24T11:58:36 +00:00	192	192	((TITLE_ABS:"protein intake" OR TITLE_ABS:"dietary protein" OR TITLE_ABS:"protein consumption" OR TITLE_ABS:"protein requirement*") AND (TITLE_ABS:"longevity" OR TITLE_ABS:"healthspan" OR TITLE_ABS:"lifespan" OR TITLE_ABS:"life expectancy" OR TITLE_ABS:"all-cause mortality" OR TITLE_ABS:"healthy ageing" OR TITLE_ABS:"healthy aging")) AND (FIRST_PDATE:[2023-01-01 TO 2026-12-31])
protein-longevity-1990	2026-08-24T12:35:59 +00:00	526	526	((TITLE_ABS:"protein intake" OR TITLE_ABS:"dietary protein" OR TITLE_ABS:"protein consumption" OR TITLE_ABS:"protein requirement*") AND (TITLE_ABS:"longevity" OR TITLE_ABS:"healthspan" OR TITLE_ABS:"lifespan" OR TITLE_ABS:"life expectancy" OR TITLE_ABS:"all-cause

Query name	Executed (UTC)	Matched	Retrieved	Query string
				mortality" OR TITLE_ABS:"healthy ageing" OR TITLE_ABS:"healthy aging")) AND (FIRST_PDATE:[1990-01-01 TO 2026-12-31])
protein-mortality-meta	2026-08-24T11:58:36 +00:00	15	15	(((TITLE_ABS:"protein intake" OR TITLE_ABS:"dietary protein" OR TITLE_ABS:"protein consumption") AND (TITLE_ABS:"all-cause mortality" OR TITLE_ABS:"total mortality")) AND (PUB_TYPE:"meta-analysis" OR PUB_TYPE:"systematic review" OR TITLE_ABS:"dose-response" OR TITLE_ABS:"pooled analysis")) AND (FIRST_PDATE:[2015-01-01 TO 2026-12-31])
protein-older-mortality	2026-08-24T11:58:37 +00:00	73	73	((TITLE_ABS:"protein intake" OR TITLE_ABS:"dietary protein") AND (TITLE_ABS:"older adult*" OR TITLE_ABS:"elderly" OR TITLE_ABS:"aged" OR TITLE_ABS:"ageing" OR TITLE_ABS:"aging") AND (TITLE_ABS:"all-cause mortality" OR TITLE_ABS:"survival" OR TITLE_ABS:"mortality risk")) AND (FIRST_PDATE:[2015-01-01 TO 2026-12-31])
protein-plus-	2026-08-24T11:58:38	86	86	((TITLE_ABS:"resistance training" OR

Query name	Executed (UTC)	Matched	Retrieved	Query string
training	+00:00			TITLE_ABS:"resistance exercise" OR TITLE_ABS:"physical activity" OR TITLE_ABS:"exercise") AND (TITLE_ABS:"protein supplementation" OR TITLE_ABS:"protein intake" OR TITLE_ABS:"dietary protein") AND (TITLE_ABS:"combined" OR TITLE_ABS:"adjunct" OR TITLE_ABS:"synerg*" OR TITLE_ABS:"multimodal" OR TITLE_ABS:"co-ingestion" OR TITLE_ABS:"interaction") AND (TITLE_ABS:"older adult*" OR TITLE_ABS:"ageing" OR TITLE_ABS:"aging" OR TITLE_ABS:"sarcopenia" OR TITLE_ABS:"healthspan")) AND (FIRST_PDATE:[2023-01-01 TO 2026-12-31])
protein-plus-training-1990	2026-08-24T12:36:01 +00:00	195	195	((TITLE_ABS:"resistance training" OR TITLE_ABS:"resistance exercise" OR TITLE_ABS:"physical activity" OR TITLE_ABS:"exercise") AND (TITLE_ABS:"protein supplementation" OR TITLE_ABS:"protein intake" OR TITLE_ABS:"dietary protein") AND (TITLE_ABS:"combined" OR TITLE_ABS:"adjunct" OR TITLE_ABS:"synerg*" OR TITLE_ABS:"multimodal" OR TITLE_ABS:"co-ingestion" OR TITLE_ABS:"interaction")

Query name	Executed (UTC)	Matched	Retrieved	Query string
				AND (TITLE_ABS:"older adult*" OR TITLE_ABS:"ageing" OR TITLE_ABS:"aging" OR TITLE_ABS:"sarcopenia" OR TITLE_ABS:"healthspan")) AND (FIRST_PDATE:[1990-01-01 TO 2026-12-31])
protein-restriction-mtor	2026-08-24T11:58:42+00:00	364	364	((TITLE_ABS:"protein restriction" OR TITLE_ABS:"low-protein diet" OR TITLE_ABS:"mTOR" OR TITLE_ABS:"IGF-1" OR TITLE_ABS:"amino acid restriction" OR TITLE_ABS:"methionine restriction") AND (TITLE_ABS:"longevity" OR TITLE_ABS:"lifespan" OR TITLE_ABS:"healthspan" OR TITLE_ABS:"ageing" OR TITLE_ABS:"aging") AND (TITLE_ABS:"human*" OR TITLE_ABS:"older adult*" OR TITLE_ABS:"protein intake" OR TITLE_ABS:"dietary protein")) AND (FIRST_PDATE:[2023-01-01 TO 2026-12-31])
protein-restriction-mtor-1990	2026-08-24T12:36:11+00:00	1,199	1,199	((TITLE_ABS:"protein restriction" OR TITLE_ABS:"low-protein diet" OR TITLE_ABS:"mTOR" OR TITLE_ABS:"IGF-1" OR TITLE_ABS:"amino acid restriction" OR TITLE_ABS:"methionine restriction") AND (TITLE_ABS:"longevity" OR TITLE_ABS:"lifespan" OR TITLE_ABS:"healthspan"

Query name	Executed (UTC)	Matched	Retrieved	Query string
				OR TITLE_ABS:"ageing" OR TITLE_ABS:"aging") AND (TITLE_ABS:"human*" OR TITLE_ABS:"older adult*" OR TITLE_ABS:"protein intake" OR TITLE_ABS:"dietary protein")) AND (FIRST_PDATE:[1990-01-01 TO 2026-12-31])
protein-sarcopenia-dose	2026-08-24T11:58:43 +00:00	78	78	((TITLE_ABS:"protein intake" OR TITLE_ABS:"dietary protein" OR TITLE_ABS:"protein supplementation" OR TITLE_ABS:"leucine" OR TITLE_ABS:"essential amino acid*") AND (TITLE_ABS:"sarcopenia" OR TITLE_ABS:"muscle mass" OR TITLE_ABS:"lean mass" OR TITLE_ABS:"muscle protein synthesis" OR TITLE_ABS:"frailty") AND (TITLE_ABS:"older adult*" OR TITLE_ABS:"elderly" OR TITLE_ABS:"ageing" OR TITLE_ABS:"aging") AND (TITLE_ABS:"recommendation*" OR TITLE_ABS:"requirement*" OR TITLE_ABS:"optimal" OR TITLE_ABS:"g/kg" OR TITLE_ABS:"dose" OR TITLE_ABS:"distribution")) AND (FIRST_PDATE:[2023-01-01 TO 2026-12-31])
protein-sarcopenia-dose-1990	2026-08-24T12:36:14 +00:00	285	285	((TITLE_ABS:"protein intake" OR TITLE_ABS:"dietary protein" OR TITLE_ABS:"protein supplementation" OR

Query name	Executed (UTC)	Matched	Retrieved	Query string
				TITLE_ABS:"leucine" OR TITLE_ABS:"essential amino acid*") AND (TITLE_ABS:"sarcopenia" OR TITLE_ABS:"muscle mass" OR TITLE_ABS:"lean mass" OR TITLE_ABS:"muscle protein synthesis" OR TITLE_ABS:"frailty") AND (TITLE_ABS:"older adult*" OR TITLE_ABS:"elderly" OR TITLE_ABS:"ageing" OR TITLE_ABS:"aging") AND (TITLE_ABS:"recommendation*" OR TITLE_ABS:"requirement*" OR TITLE_ABS:"optimal" OR TITLE_ABS:"g/kg" OR TITLE_ABS:"dose" OR TITLE_ABS:"distribution")) AND (FIRST_PDATE:[1990-01-01 TO 2026-12-31])
protein-ukbiobank	2026-08-24T11:58:44 +00:00	54	54	((TITLE_ABS:"UK Biobank" OR TITLE_ABS:"NHANES" OR TITLE_ABS:"prospective cohort") AND (TITLE_ABS:"protein intake" OR TITLE_ABS:"dietary protein") AND (TITLE_ABS:"mortality" OR TITLE_ABS:"cardiovascular")) AND (FIRST_PDATE:[2023-01-01 TO 2026-12-31])
protein-ukbiobank-1990	2026-08-24T12:36:15 +00:00	131	131	((TITLE_ABS:"UK Biobank" OR TITLE_ABS:"NHANES" OR TITLE_ABS:"prospective cohort") AND (TITLE_ABS:"protein intake" OR TITLE_ABS:"dietary protein") AND

Query name	Executed (UTC)	Matched	Retrieved	Query string
				(TITLE_ABS:"mortality" OR TITLE_ABS:"cardiovascular")) AND (FIRST_PDATE:[1990-01-01 TO 2026-12-31])
rct-exercise-clinical-pooled	2026-08-24T11:58:45 +00:00	78	78	((TITLE_ABS:"exercise training" OR TITLE_ABS:"cardiac rehabilitation" OR TITLE_ABS:"exercise intervention" OR TITLE_ABS:"supervised exercise") AND (TITLE_ABS:"heart failure" OR TITLE_ABS:"coronary artery disease" OR TITLE_ABS:"type 2 diabetes" OR TITLE_ABS:"chronic kidney disease" OR TITLE_ABS:"cancer survivor*") AND (TITLE_ABS:"mortality" OR TITLE_ABS:"all-cause" OR TITLE_ABS:"hospitalisation" OR TITLE_ABS:"hospitalization" OR TITLE_ABS:"MACE") AND (PUB_TYPE:"randomized controlled trial" OR PUB_TYPE:"controlled clinical trial" OR TITLE_ABS:"randomized" OR TITLE_ABS:"randomised" OR TITLE_ABS:"randomly assigned" OR TITLE_ABS:"placebo" OR TITLE:"trial") AND (PUB_TYPE:"meta-analysis" OR PUB_TYPE:"systematic review" OR

Query name	Executed (UTC)	Matched	Retrieved	Query string
				TITLE_ABS:"meta-analysis" OR TITLE_ABS:"pooled analysis")) AND (FIRST_PDATE:[2015-01-01 TO 2026-12-31])
rct-exercise-dose-pooled	2026-08-24T11:58:46 +00:00	55	55	((TITLE_ABS:"exercise dose" OR TITLE_ABS:"exercise volume" OR TITLE_ABS:"dose-response" OR TITLE_ABS:"minimal dose" OR TITLE_ABS:"training frequency" OR TITLE_ABS:"weekly sets") AND (TITLE_ABS:"randomized" OR TITLE_ABS:"randomised") AND (TITLE_ABS:"muscle strength" OR TITLE_ABS:"cardiorespiratory fitness" OR TITLE_ABS:"VO2" OR TITLE_ABS:"functional capacity") AND (PUB_TYPE:"meta-analysis" OR PUB_TYPE:"systematic review" OR TITLE_ABS:"meta-analysis" OR TITLE_ABS:"pooled analysis")) AND (FIRST_PDATE:[2015-01-01 TO 2026-12-31])
rct-exercise-older-outcomes	2026-08-24T11:58:49 +00:00	317	317	((TITLE_ABS:"exercise training" OR TITLE_ABS:"physical activity intervention" OR TITLE_ABS:"structured exercise" OR TITLE_ABS:"resistance training" OR TITLE_ABS:"aerobic training") AND

Query name	Executed (UTC)	Matched	Retrieved	Query string
				(TITLE_ABS:"mortality" OR TITLE_ABS:"major mobility disability" OR TITLE_ABS:"disability" OR TITLE_ABS:"frailty" OR TITLE_ABS:"falls" OR TITLE_ABS:"hospitalisation" OR TITLE_ABS:"hospitalization") AND (TITLE_ABS:"older adult*" OR TITLE_ABS:"elderly" OR TITLE_ABS:"aged") AND (PUB_TYPE:"randomized controlled trial" OR PUB_TYPE:"controlled clinical trial" OR TITLE_ABS:"randomized" OR TITLE_ABS:"randomised" OR TITLE_ABS:"randomly assigned" OR TITLE_ABS:"placebo" OR TITLE:"trial")) AND (FIRST_PDATE:[2015-01-01 TO 2026-12-31])
rct-highprotein-renal-safety	2026-08-24T11:58:51+00:00	77	77	((TITLE_ABS:"high-protein diet" OR TITLE_ABS:"high protein intake" OR TITLE_ABS:"protein supplementation") AND (TITLE_ABS:"renal function" OR TITLE_ABS:"kidney function" OR TITLE_ABS:"glomerular filtration" OR TITLE_ABS:"eGFR" OR TITLE_ABS:"albuminuria" OR TITLE_ABS:"creatinine" OR TITLE_ABS:"safety" OR TITLE_ABS:"adverse event*") AND (PUB_TYPE:"randomized

Query name	Executed (UTC)	Matched	Retrieved	Query string
				controlled trial" OR PUB_TYPE:"controlled clinical trial" OR TITLE_ABS:"randomized" OR TITLE_ABS:"randomised" OR TITLE_ABS:"randomly assigned" OR TITLE_ABS:"placebo" OR TITLE:"trial")) AND (FIRST_PDATE:[2015-01-01 TO 2026-12-31])
rct-lpd-ckd	2026-08-24T11:58:52 +00:00	57	57	((TITLE_ABS:"low-protein diet" OR TITLE_ABS:"protein restriction" OR TITLE_ABS:"very low protein diet" OR TITLE_ABS:"ketoanalogue*" OR TITLE_ABS:"keto-analogue*") AND (TITLE_ABS:"chronic kidney disease" OR TITLE_ABS:"CKD" OR TITLE_ABS:"dialysis" OR TITLE_ABS:"diabetic nephropathy" OR TITLE_ABS:"diabetic kidney disease") AND (TITLE_ABS:"mortality" OR TITLE_ABS:"progression" OR TITLE_ABS:"end-stage" OR TITLE_ABS:"eGFR" OR TITLE_ABS:"proteinuria") AND (PUB_TYPE:"randomized controlled trial" OR PUB_TYPE:"controlled clinical trial" OR TITLE_ABS:"randomized" OR TITLE_ABS:"randomised" OR TITLE_ABS:"randomly assigned" OR TITLE_ABS:"placebo" OR TITLE:"trial")) AND

Query name	Executed (UTC)	Matched	Retrieved	Query string
				(FIRST_PDATE:[2015-01-01 TO 2026-12-31])
rct-multidomain	2026-08-24T11:58:54+00:00	255	255	((TITLE_ABS:"multidomain" OR TITLE_ABS:"multicomponent" OR TITLE_ABS:"lifestyle intervention") AND (TITLE_ABS:"exercise" OR TITLE_ABS:"physical activity") AND (TITLE_ABS:"protein" OR TITLE_ABS:"nutrition" OR TITLE_ABS:"diet") AND (TITLE_ABS:"frailty" OR TITLE_ABS:"sarcopenia" OR TITLE_ABS:"cognitive" OR TITLE_ABS:"disability" OR TITLE_ABS:"healthy ageing" OR TITLE_ABS:"healthy aging") AND (PUB_TYPE:"randomized controlled trial" OR PUB_TYPE:"controlled clinical trial" OR TITLE_ABS:"randomized" OR TITLE_ABS:"randomised" OR TITLE_ABS:"randomly assigned" OR TITLE_ABS:"placebo" OR TITLE:"trial")) AND (FIRST_PDATE:[2015-01-01 TO 2026-12-31])
rct-protein-cvd	2026-08-24T11:58:56+00:00	200	200	((TITLE_ABS:"protein intake" OR TITLE_ABS:"dietary protein" OR TITLE_ABS:"protein supplementation") AND (TITLE_ABS:"heart failure" OR TITLE_ABS:"cardiovascular" OR TITLE_ABS:"blood pressure" OR

Query name	Executed (UTC)	Matched	Retrieved	Query string
				TITLE_ABS:"LDL cholesterol" OR TITLE_ABS:"cardiac") AND (PUB_TYPE:"randomized controlled trial" OR PUB_TYPE:"controlled clinical trial" OR TITLE_ABS:"randomized" OR TITLE_ABS:"randomised" OR TITLE_ABS:"randomly assigned" OR TITLE_ABS:"placebo" OR TITLE:"trial")) AND (FIRST_PDATE:[2015-01-01 TO 2026-12-31])
rct-protein-dose-response	2026-08-24T11:58:58 +00:00	162	162	((TITLE_ABS:"protein distribution" OR TITLE_ABS:"per-meal protein" OR TITLE_ABS:"protein dose" OR TITLE_ABS:"dose-response" OR TITLE_ABS:"g/kg" OR TITLE_ABS:"protein timing") AND (TITLE_ABS:"muscle protein synthesis" OR TITLE_ABS:"myofibrillar" OR TITLE_ABS:"nitrogen balance" OR TITLE_ABS:"protein requirement*" OR TITLE_ABS:"indicator amino acid oxidation") AND (PUB_TYPE:"randomized controlled trial" OR PUB_TYPE:"controlled clinical trial" OR TITLE_ABS:"randomized" OR TITLE_ABS:"randomised" OR TITLE_ABS:"randomly assigned" OR TITLE_ABS:"placebo" OR TITLE:"trial")) AND

Query name	Executed (UTC)	Matched	Retrieved	Query string
				(FIRST_PDATE:[2015-01-01 TO 2026-12-31])
rct-protein-obesity-leanmass	2026-08-24T11:59:00 +00:00	242	242	((TITLE_ABS:"high-protein diet" OR TITLE_ABS:"protein intake" OR TITLE_ABS:"protein supplementation" OR TITLE_ABS:"energy-restricted") AND (TITLE_ABS:"obesity" OR TITLE_ABS:"obese" OR TITLE_ABS:"weight loss" OR TITLE_ABS:"bariatric" OR TITLE_ABS:"GLP-1" OR TITLE_ABS:"semaglutide" OR TITLE_ABS:"tirzepatide") AND (TITLE_ABS:"lean mass" OR TITLE_ABS:"fat-free mass" OR TITLE_ABS:"muscle mass" OR TITLE_ABS:"body composition" OR TITLE_ABS:"sarcopenic obesity") AND (PUB_TYPE:"randomized controlled trial" OR PUB_TYPE:"controlled clinical trial" OR TITLE_ABS:"randomized" OR TITLE_ABS:"randomised" OR TITLE_ABS:"randomly assigned" OR TITLE_ABS:"placebo" OR TITLE:"trial")) AND (FIRST_PDATE:[2015-01-01 TO 2026-12-31])
rct-protein-older-function	2026-08-24T11:59:02 +00:00	333	333	((TITLE_ABS:"protein supplementation" OR TITLE_ABS:"dietary protein" OR TITLE_ABS:"protein intake" OR TITLE_ABS:"whey protein" OR TITLE_ABS:"leucine"

Query name	Executed (UTC)	Matched	Retrieved	Query string
				OR TITLE_ABS:"essential amino acid*") AND (TITLE_ABS:"older adult*" OR TITLE_ABS:"elderly" OR TITLE_ABS:"sarcopenia" OR TITLE_ABS:"frailty") AND (TITLE_ABS:"lean mass" OR TITLE_ABS:"muscle mass" OR TITLE_ABS:"muscle strength" OR TITLE_ABS:"grip strength" OR TITLE_ABS:"physical function" OR TITLE_ABS:"gait speed" OR TITLE_ABS:"SPPB") AND (PUB_TYPE:"randomized controlled trial" OR PUB_TYPE:"controlled clinical trial" OR TITLE_ABS:"randomized" OR TITLE_ABS:"randomised" OR TITLE_ABS:"randomly assigned" OR TITLE_ABS:"placebo" OR TITLE:"trial")) AND (FIRST_PDATE:[2015-01-01 TO 2026-12-31])
rct-protein-plus-rt-pooled	2026-08-24T11:59:03 +00:00	46	46	((TITLE_ABS:"protein supplementation" OR TITLE_ABS:"dietary protein") AND (TITLE_ABS:"resistance training" OR TITLE_ABS:"resistance exercise" OR TITLE_ABS:"strength training") AND (TITLE_ABS:"lean mass" OR TITLE_ABS:"muscle mass" OR TITLE_ABS:"strength" OR TITLE_ABS:"hypertrophy") AND (PUB_TYPE:"meta-

Query name	Executed (UTC)	Matched	Retrieved	Query string
				analysis" OR PUB_TYPE:"systematic review" OR TITLE_ABS:"meta-analysis" OR TITLE_ABS:"pooled analysis") AND (PUB_TYPE:"randomized controlled trial" OR PUB_TYPE:"controlled clinical trial" OR TITLE_ABS:"randomized" OR TITLE_ABS:"randomised" OR TITLE_ABS:"randomly assigned" OR TITLE_ABS:"placebo" OR TITLE:"trial")) AND (FIRST_PDATE:[2015-01-01 TO 2026-12-31])
rct-protein-t2dm	2026-08-24T11:59:06 +00:00	217	217	((TITLE_ABS:"high-protein diet" OR TITLE_ABS:"protein intake" OR TITLE_ABS:"dietary protein" OR TITLE_ABS:"protein supplementation") AND (TITLE_ABS:"type 2 diabetes" OR TITLE_ABS:"insulin resistance" OR TITLE_ABS:"HbA1c" OR TITLE_ABS:"glycaemic control" OR TITLE_ABS:"glycemic control" OR TITLE_ABS:"insulin sensitivity") AND (PUB_TYPE:"randomized controlled trial" OR PUB_TYPE:"controlled clinical trial" OR TITLE_ABS:"randomized" OR TITLE_ABS:"randomised" OR TITLE_ABS:"randomly

Query name	Executed (UTC)	Matched	Retrieved	Query string
				assigned" OR TITLE_ABS:"placebo" OR TITLE:"trial")) AND (FIRST_PDATE:[2015-01-01 TO 2026-12-31])
selfreport-error	2026-08-24T11:59:07 +00:00	37	37	((TITLE_ABS:"self-report*" AND (TITLE_ABS:"misreporting" OR TITLE_ABS:"under-reporting" OR TITLE_ABS:"underreporting" OR TITLE_ABS:"measurement error" OR TITLE_ABS:"bias")) AND (TITLE_ABS:"dietary intake" OR TITLE_ABS:"energy intake" OR TITLE_ABS:"food frequency questionnaire" OR TITLE_ABS:"24-hour recall")) AND (FIRST_PDATE:[2018-01-01 TO 2026-12-31])
sport-nutrition-longevity	2026-08-24T11:59:08 +00:00	73	73	((TITLE_ABS:"sports nutrition" OR TITLE_ABS:"sport nutrition" OR TITLE_ABS:"athlete*" OR TITLE_ABS:"masters athlete*" OR TITLE_ABS:"exercise nutrition") AND (TITLE_ABS:"longevity" OR TITLE_ABS:"healthspan" OR TITLE_ABS:"lifespan" OR TITLE_ABS:"healthy ageing" OR TITLE_ABS:"healthy aging" OR TITLE_ABS:"biological age")) AND (FIRST_PDATE:[2023-01-01 TO 2026-12-31])

Query name	Executed (UTC)	Matched	Retrieved	Query string
sport-nutrition-longevity-1990	2026-08-24T12:36:17 +00:00	173	173	((TITLE_ABS:"sports nutrition" OR TITLE_ABS:"sport nutrition" OR TITLE_ABS:"athlete*" OR TITLE_ABS:"masters athlete*" OR TITLE_ABS:"exercise nutrition") AND (TITLE_ABS:"longevity" OR TITLE_ABS:"healthspan" OR TITLE_ABS:"lifespan" OR TITLE_ABS:"healthy ageing" OR TITLE_ABS:"healthy aging" OR TITLE_ABS:"biological age")) AND (FIRST_PDATE:[1990-01-01 TO 2026-12-31])
steps-vilpa-mortality	2026-08-24T11:59:09 +00:00	105	105	((TITLE_ABS:"steps per day" OR TITLE_ABS:"step count" OR TITLE_ABS:"daily steps" OR TITLE_ABS:"accelerometer-measured" OR TITLE_ABS:"device-measured physical activity" OR TITLE_ABS:"vigorous intermittent lifestyle physical activity" OR TITLE_ABS:"VILPA") AND (TITLE_ABS:"all-cause mortality" OR TITLE_ABS:"longevity" OR TITLE_ABS:"life expectancy" OR TITLE_ABS:"healthspan" OR TITLE_ABS:"cardiovascular mortality")) AND (FIRST_PDATE:[2023-01-01 TO 2026-12-31])

Query name	Executed (UTC)	Matched	Retrieved	Query string
steps-vilpa-mortality-1990	2026-08-24T12:36:19 +00:00	190	190	((TITLE_ABS:"steps per day" OR TITLE_ABS:"step count" OR TITLE_ABS:"daily steps" OR TITLE_ABS:"accelerometer-measured" OR TITLE_ABS:"device-measured physical activity" OR TITLE_ABS:"vigorous intermittent lifestyle physical activity" OR TITLE_ABS:"VILPA") AND (TITLE_ABS:"all-cause mortality" OR TITLE_ABS:"longevity" OR TITLE_ABS:"life expectancy" OR TITLE_ABS:"healthspan" OR TITLE_ABS:"cardiovascular mortality")) AND (FIRST_PDATE:[1990-01-01 TO 2026-12-31])

Semantic Scholar — 67 queries, 73,703 matched, 73,701 retrieved

Query name	Executed (UTC)	Matched	Retrieved	Query string
ai-diet-assessment	2026-08-24T12:12:45 +00:00	2,156	2,156	(("artificial intelligence" \\| "machine learning" \\| "deep learning" \\| "computer vision" \\| ("convolutional neural network" \\| "convolutional neural networks") \\| "image-based" \\| "image based" \\| "smartphone" \\| ("large language model" \\| "large language models") \\| "automated") + ("dietary assessment" \\| "dietary intake" \\| ("food record" \\| "food

Query name	Executed (UTC)	Matched	Retrieved	Query string
				records") | "24-hour recall" | "food frequency questionnaire" | ("food photograph" | "food photographs" | "food photography") | "portion size" | "food recognition" | "nutrient estimation" | "energy intake"))
ai-diet-validation	2026-08-24T12:12:48 +00:00	997	997	(("artificial intelligence" | "machine learning" | "deep learning" | "computer vision" | ("convolutional neural network" | "convolutional neural networks") | "image-based" | "image based" | "smartphone" | ("large language model" | "large language models") | "automated") + ("dietary assessment" | "dietary intake" | ("food record" | "food records") | "24-hour recall" | "food frequency questionnaire" | ("food photograph" | "food photographs" | "food photography") | "portion size" | "food recognition" | "nutrient estimation" | "energy intake") + ("validity" | "validation" | "accuracy" | "agreement" | "reliability" | "weighed food record" | "doubly labelled water" | "doubly labeled water"))

Query name	Executed (UTC)	Matched	Retrieved	Query string
ai-protein-monitoring	2026-08-24T12:12:51 +00:00	340	340	(("artificial intelligence" | "machine learning" | "deep learning" | "computer vision" | ("convolutional neural network" | "convolutional neural networks") | "image-based" | "image based" | "smartphone" | ("large language model" | "large language models") | "automated") + ("protein intake" | "dietary protein" | ("macronutrient" | "macronutrients")) + ("assessment" | "monitoring" | "tracking" | "estimation"))
cardiovascular	2026-08-24T12:12:54 +00:00	176	176	(("protein intake" | "dietary protein") + ("heart failure" | "coronary" | "myocardial" | "stroke" | "cardiovascular disease" | "cardiac cachexia") + ("mortality" | "prognosis" | "survival" | "g/kg" | ("protein requirement" | "protein requirements")))
ckd	2026-08-24T12:12:55 +00:00	121	121	(("protein intake" | "dietary protein" | "low-protein diet") + ("g/kg" | "g per kg" | ("protein requirement" | "protein requirements") | "optimal protein" | "protein target" | "protein dose") + ("chronic kidney

Query name	Executed (UTC)	Matched	Retrieved	Query string
				disease" | "CKD" | "dialysis" | "eGFR" | "renal") + ("mortality" | "progression" | "survival"))
diabetes	2026-08-24T12:12:58 +00:00	48	48	(("protein intake" | "dietary protein") + ("g/kg" | "g per kg" | "grams per kilogram" | ("protein requirement" | "protein requirements") | "optimal protein" | "protein target" | "protein dose") + ("type 2 diabetes" | "diabetic" | "diabetes mellitus") + ("mortality" | "nephropathy" | "glycemic" | ("outcome" | "outcomes")))
diabetes2	2026-08-24T12:13:05 +00:00	1,573	1,573	(("protein intake" | "dietary protein" | "high-protein diet" | "protein supplementation") + ("type 2 diabetes" | "diabetes mellitus" | "diabetic" | "insulin resistance" | "glycaemic" | "glycemic" | "HbA1c"))
glp1-guidance	2026-08-24T12:13:08 +00:00	438	438	(("GLP-1" | "GLP1" | "glucagon-like peptide-1" | "glucagon like peptide 1" | "incretin" | "semaglutide" | "tirzepatide" | "liraglutide" | "dulaglutide" | "exenatide" | "retatrutide" | "GIP/GLP-1") + ("consensus" | "position statement" |

Query name	Executed (UTC)	Matched	Retrieved	Query string
				("guideline" | "guidelines") | ("recommendation" | "recommendations") | "practical guidance" | "expert opinion") + ("nutrition" | "protein" | "muscle" | "lean mass" | "sarcopenia"))
glp1-leanmass	2026-08-24T12:13:14 +00:00	1,581	1,581	(("GLP-1" | "GLP1" | "glucagon-like peptide-1" | "glucagon like peptide 1" | "incretin" | "semaglutide" | "tirzepatide" | "liraglutide" | "dulaglutide" | "exenatide" | "retatrutide" | "GIP/GLP-1") + ("lean mass" | "fat-free mass" | "fat free mass" | "muscle mass" | "skeletal muscle" | "sarcopenia" | "sarcopenic obesity" | "muscle strength" | "grip strength" | "body composition" | "appendicular" | "muscle loss"))
glp1-preservation	2026-08-24T12:13:17 +00:00	185	185	((("GLP-1" | "GLP1" | "glucagon-like peptide-1" | "glucagon like peptide 1" | "incretin" | "semaglutide" | "tirzepatide" | "liraglutide" | "dulaglutide" | "exenatide" | "retatrutide" | "GIP/GLP-1") | "weight loss" | "caloric restriction" | "bariatric") + ("muscle-preserving" | "muscle

Query name	Executed (UTC)	Matched	Retrieved	Query string
				preservation" | "preserve lean" | "preserving lean" | "iatrogenic sarcopenia" | "sarcopenic obesity") + ("protein" | "resistance training"))
glp1-protein	2026-08-24T12:13:20 +00:00	524	524	(("GLP-1" | "GLP1" | "glucagon-like peptide-1" | "glucagon like peptide 1" | "incretin" | "semaglutide" | "tirzepatide" | "liraglutide" | "dulaglutide" | "exenatide" | "retatrutide" | "GIP/GLP-1") + ("protein intake" | "dietary protein" | "protein supplementation" | "high-protein" | "whey protein" | "leucine" | ("essential amino acid" | "essential amino acids") | ("protein requirement" | "protein requirements") | "g/kg"))
glp1-randomised	2026-08-24T12:13:23 +00:00	368	368	(("GLP-1" | "GLP1" | "glucagon-like peptide-1" | "glucagon like peptide 1" | "incretin" | "semaglutide" | "tirzepatide" | "liraglutide" | "dulaglutide" | "exenatide" | "retatrutide" | "GIP/GLP-1") + ("lean mass" | "fat-free mass" | "fat free mass" | "muscle mass" | "skeletal muscle" | "sarcopenia" | "sarcopenic obesity" | "muscle strength" |

Query name	Executed (UTC)	Matched	Retrieved	Query string
				"grip strength" | "body composition" | "appendicular" | "muscle loss") + ("randomized controlled trial" | "randomized" | "randomised" | "placebo" | "meta-analysis" | "systematic review"))
glp1-training	2026-08-24T12:13:25 +00:00	203	203	(("GLP-1" | "GLP1" | "glucagon-like peptide-1" | "glucagon like peptide 1" | "incretin" | "semaglutide" | "tirzepatide" | "liraglutide" | "dulaglutide" | "exenatide" | "retatrutide" | "GIP/GLP-1") + ("resistance training" | "resistance exercise" | "strength training" | "exercise training" | "physical activity") + ("lean mass" | "fat-free mass" | "fat free mass" | "muscle mass" | "skeletal muscle" | "sarcopenia" | "sarcopenic obesity" | "muscle strength" | "grip strength" | "body composition" | "appendicular" | "muscle loss"))
healthy	2026-08-24T12:13:28 +00:00	93	93	(("protein intake" | "dietary protein") + ("g/kg" | "g per kg" | "grams per kilogram" | ("protein requirement" | "protein requirements") | "optimal protein" | "protein target" | "protein dose") +

Query name	Executed (UTC)	Matched	Retrieved	Query string
				(("healthy adult" | "healthy adults") | "general population" | "community-dwelling" | "healthy older") + ("mortality" | "sarcopenia" | "muscle mass" | "healthspan" | ("requirement" | "requirements")))
highprotein-safety-cvd	2026-08-24T12:13:31 +00:00	249	249	(("high-protein diet" | "high protein intake" | "protein overconsumption") + ("cardiovascular" | "mortality" | "safety" | "renal" | "kidney") + ("randomized" | "trial" | "cohort" | "meta-analysis"))
highprotein-upper	2026-08-24T12:13:41 +00:00	2,221	2,221	(("high-protein diet" | "high protein intake" | "1.6 g/kg" | "2.0 g/kg" | "2.2 g/kg" | "upper limit" | "safe upper" | "tolerable upper intake") + ("protein") + ("safety" | "renal function" | "kidney function" | "adverse" | "upper limit" | "randomized" | "randomised"))
hyp-athlete-req	2026-08-24T12:13:44 +00:00	473	473	((("athlete" | "athletes") | "resistance-trained" | "resistance trained" | ("bodybuilder" | "bodybuilders") | ("strength athlete" | "strength athletes")) + (("protein requirement" | "protein requirements") | "protein intake" | ("protein

Query name	Executed (UTC)	Matched	Retrieved	Query string
				recommendation" | "protein recommendations") | "g/kg") + ("position stand" | "consensus" | ("recommendation" | "recommendations") | ("requirement" | "requirements") | "hypertrophy" | "lean mass"))
hyp-dose-response	2026-08-24T12:13:47 +00:00	272	272	(("protein supplementation" | "protein intake" | "dietary protein") + ("resistance training" | "resistance exercise" | "strength training") + ("dose-response" | "plateau" | "breakpoint" | "optimal" | "g/kg" | "threshold") + ("fat-free mass" | "lean body mass" | "muscle mass" | "hypertrophy" | "1RM" | "strength"))
hyp-iaao-nbal	2026-08-24T12:13:50 +00:00	228	228	(("indicator amino acid oxidation" | "nitrogen balance" | ("protein requirement" | "protein requirements")) + ("resistance training" | ("athlete" | "athletes") | "exercise" | "endurance"))
hyp-veryhigh	2026-08-24T12:13:53 +00:00	455	455	(("high protein" | "2 g/kg" | "2.2 g/kg" | "3 g/kg" | "supraphysiological" | "very high protein") + ("resistance training" | "body composition" | "lean mass") + ("randomized" |

Query name	Executed (UTC)	Matched	Retrieved	Query string
				"randomised" | "trial"))
mb-exercise	2026-08-24T12:14:42 +00:00	10,875	10,875	(("gut microbiota" | "gut microbiome" | "intestinal microbiota" | "microbiome" | "microbiota" | ("short-chain fatty acid" | "short-chain fatty acids") | "SCFA" | "butyrate" | "propionate" | "faecal microbiota" | "fecal microbiota") + ("exercise" | "physical activity" | "resistance training" | "endurance training" | ("athlete" | "athletes") | "lactate" | "Veillonella"))
mb-gutmuscle-axis	2026-08-24T12:14:49 +00:00	1,045	1,045	(("gut-muscle axis" | "gut muscle axis" | "microbiota-gut-muscle" | "muscle-gut") | (("gut microbiota" | "gut microbiome" | "intestinal microbiota" | "microbiome" | "microbiota" | ("short-chain fatty acid" | "short-chain fatty acids") | "SCFA" | "butyrate" | "propionate" | "faecal microbiota" | "fecal microbiota") + ("sarcopenia" | "muscle mass" | "skeletal muscle" | "muscle strength" | "grip strength" | "physical function" | "frailty" | "lean mass" | "muscle protein synthesis") + ("randomized" |

Query name	Executed (UTC)	Matched	Retrieved	Query string
				"randomised" | ("probiotic" | "probiotics") | ("prebiotic" | "prebiotics") | ("synbiotic" | "synbiotics") | "fibre" | "fiber")))
mb-longevity	2026-08-24T12:15:41 +00:00	10,297	10,297	(("gut microbiota" | "gut microbiome" | "intestinal microbiota" | "microbiome" | "microbiota" | ("short-chain fatty acid" | "short-chain fatty acids") | "SCFA" | "butyrate" | "propionate" | "faecal microbiota" | "fecal microbiota") + ("longevity" | "healthspan" | "ageing" | "aging" | "all-cause mortality" | ("centenarian" | "centenarians")) + (("human" | "humans") | ("older adult" | "older adults") | "cohort"))
mb-muscle	2026-08-24T12:15:54 +00:00	2,318	2,318	(("gut microbiota" | "gut microbiome" | "intestinal microbiota" | "microbiome" | "microbiota" | ("short-chain fatty acid" | "short-chain fatty acids") | "SCFA" | "butyrate" | "propionate" | "faecal microbiota" | "fecal microbiota") + ("sarcopenia" | "muscle mass" | "skeletal muscle" | "muscle strength" | "grip strength" | "physical

Query name	Executed (UTC)	Matched	Retrieved	Query string
				function" | "frailty" | "lean mass" | "muscle protein synthesis"))
mb-protein	2026-08-24T12:17:12 +00:00	15,039	15,039	(("gut microbiota" | "gut microbiome" | "intestinal microbiota" | "microbiome" | "microbiota" | ("short-chain fatty acid" | "short-chain fatty acids") | "SCFA" | "butyrate" | "propionate" | "faecal microbiota" | "fecal microbiota") + ("dietary protein" | "protein intake" | "protein fermentation" | "plant protein" | "animal protein" | "amino acid" | "putrefaction" | "p-cresol" | "indoxyl sulfate" | "TMAO" | "trimethylamine"))
med-grip-mortality	2026-08-24T12:17:17 +00:00	845	845	(("handgrip strength" | "grip strength" | "muscle strength") + ("all-cause mortality" | "mortality") + ("meta-analysis" | "systematic review" | "dose-response" | "per 5 kg" | "per kg" | "hazard ratio"))
med-musclemass-mortality	2026-08-24T12:12:25 +00:00	135	135	(("muscle mass" | "lean body mass" | "fat-free mass" | "skeletal muscle index") + ("mortality" | "survival") + ("cohort" | "prospective") + ("meta-analysis" | "dose-response"))
med-sarcopenia-	2026-08-24T12:17:21	319	319	(("sarcopenia" | "low muscle mass" |

Query name	Executed (UTC)	Matched	Retrieved	Query string
mortality	+00:00			"appendicular lean mass" | "muscle wasting") + ("all-cause mortality" | "cardiovascular mortality" | "major adverse cardiovascular" | "MACE") + ("meta-analysis" | "systematic review" | "pooled" | "hazard ratio"))
obesity	2026-08-24T12:17:24 +00:00	157	157	(("protein intake" | "dietary protein" | "high-protein diet") + ("g/kg" | "g per kg" | "grams per kilogram" | ("protein requirement" | "protein requirements") | "optimal protein" | "protein target" | "protein dose") + ("obesity" | "obese" | "weight loss" | "bariatric" | "sarcopenic obesity") + ("mortality" | "lean mass" | "muscle" | ("outcome" | "outcomes")))
pa-clinical-populations	2026-08-24T12:17:27 +00:00	304	304	(("physical activity" | "exercise") + (("cancer survivor" | "cancer survivors") | "coronary" | "heart failure" | "chronic kidney disease" | "COPD" | "type 2 diabetes" | "stroke" | "hypertension") + ("meeting" | ("guideline" | "guidelines") | ("recommendation" | "recommendations" | "recommended") | "active vs" | "inactive") + ("all-

Query name	Executed (UTC)	Matched	Retrieved	Query string
				cause mortality" | "all cause mortality" | "total mortality") + ("prospective cohort" | "cohort study" | "prospective study" | "follow-up study" | ("hazard ratio" | "hazard ratios")))
pa-device-measured	2026-08-24T12:17:31 +00:00	177	177	(("accelerometer" | "accelerometry" | "device-measured" | "device measured" | "wrist-worn" | "wearable" | ("actigraph" | "actigraphy" | "actigraphic")) + ("physical activity" | "MVPA" | "steps") + ("all-cause mortality" | "all cause mortality" | "total mortality") + ("prospective cohort" | "cohort study" | "prospective study" | "follow-up study" | ("hazard ratio" | "hazard ratios")))
pa-dose-mortality	2026-08-24T12:17:34 +00:00	181	181	(("physical activity" | "exercise" | "aerobic exercise" | "resistance training" | "muscle-strengthening") + ("dose-response" | "minimal dose" | "minimum dose" | "minimum effective dose" | "weekly volume" | "MET-minutes") + ("all-cause mortality" | "longevity" | "lifespan" | "life expectancy" | "healthspan"))
pa-dose-	2026-08-	460	460	(("physical activity" |

Query name	Executed (UTC)	Matched	Retrieved	Query string
mortality-1990	24T12:36:30 +00:00			"exercise" | "aerobic exercise" | "resistance training" | "muscle-strengthening") + ("dose-response" | "minimal dose" | "minimum dose" | "minimum effective dose" | "weekly volume" | "MET-minutes") + ("all-cause mortality" | "longevity" | "lifespan" | "life expectancy" | "healthspan"))
pa-guideline-adherence	2026-08-24T12:17:37 +00:00	173	173	((("physical activity guideline" | "physical activity guidelines") | "meeting the guidelines" | "guideline-concordant" | "guideline adherence" | ("WHO recommendation" | "WHO recommendations") | "150 min" | "150 minutes" | "500-1000 MET" | "600 MET") + ("physical activity" | "exercise" | "MVPA") + ("all-cause mortality" | "all cause mortality" | "total mortality") + ("prospective cohort" | "cohort study" | "prospective study" | "follow-up study" | ("hazard ratio" | "hazard ratios")))
pa-guidelines-healthspan	2026-08-24T12:17:40 +00:00	390	390	(("physical activity guidelines" | "exercise guidelines" | ("physical activity recommendation" | "physical activity recommendations") | "WHO guidelines" | "consensus statement") +

Query name	Executed (UTC)	Matched	Retrieved	Query string
				("physical activity" | "exercise") + ("healthy ageing" | "healthy aging" | "healthspan" | "longevity" | "mortality" | "older adults"))
pa-guidelines-healthspan-1990	2026-08-24T12:36:37 +00:00	1,409	1,407	(("physical activity guidelines" | "exercise guidelines" | ("physical activity recommendation" | "physical activity recommendations") | "WHO guidelines" | "consensus statement") + ("physical activity" | "exercise") + ("healthy ageing" | "healthy aging" | "healthspan" | "longevity" | "mortality" | "older adults"))
pa-pooled-cohorts	2026-08-24T12:17:44 +00:00	105	105	(("pooled analysis" | "pooled cohort" | "consortium" | "harmonised" | "harmonized" | "individual participant data") + ("physical activity" | "exercise") + ("all-cause mortality" | "all cause mortality" | "total mortality"))
pa-quantile-contrast	2026-08-24T12:17:46 +00:00	177	177	(("leisure-time physical activity" | "leisure time physical activity" | "total physical activity" | "moderate-to-vigorous physical activity" | "MVPA") + ("most active" | "highest quartile" | "highest quintile" | "highest tertile" | "inactive" |

Query name	Executed (UTC)	Matched	Retrieved	Query string
				"sedentary" | "physically inactive") + ("all-cause mortality" | "all cause mortality" | "total mortality") + ("prospective cohort" | "cohort study" | "prospective study" | "follow-up study" | ("hazard ratio" | "hazard ratios")))
protein-longevity	2026-08-24T12:17:50 +00:00	233	233	(("protein intake" | "dietary protein" | "protein consumption" | ("protein requirement" | "protein requirements")) + ("longevity" | "healthspan" | "lifespan" | "life expectancy" | "all-cause mortality" | "healthy ageing" | "healthy aging"))
protein-longevity-1990	2026-08-24T12:36:40 +00:00	779	779	(("protein intake" | "dietary protein" | "protein consumption" | ("protein requirement" | "protein requirements")) + ("longevity" | "healthspan" | "lifespan" | "life expectancy" | "all-cause mortality" | "healthy ageing" | "healthy aging"))
protein-mortality-meta	2026-08-24T12:17:53 +00:00	23	23	((("protein intake" | "dietary protein" | "protein consumption") + ("all-cause mortality" | "total mortality")) + ("meta-analysis" | "systematic review" | "dose-response" | "pooled analysis"))

Query name	Executed (UTC)	Matched	Retrieved	Query string
protein-older-mortality	2026-08-24T12:17:56 +00:00	259	259	(("protein intake" | "dietary protein") + (("older adult" | "older adults") | "elderly" | "aged" | "ageing" | "aging") + ("all-cause mortality" | "survival" | "mortality risk"))
protein-plus-training	2026-08-24T12:17:59 +00:00	222	222	(("resistance training" | "resistance exercise" | "physical activity" | "exercise") + ("protein supplementation" | "protein intake" | "dietary protein") + ("combined" | "adjunct" | ("synergy" | "synergistic" | "synergism") | "multimodal" | "co-ingestion" | "interaction") + (("older adult" | "older adults") | "ageing" | "aging" | "sarcopenia" | "healthspan"))
protein-plus-training-1990	2026-08-24T12:36:43 +00:00	674	674	(("resistance training" | "resistance exercise" | "physical activity" | "exercise") + ("protein supplementation" | "protein intake" | "dietary protein") + ("combined" | "adjunct" | ("synergy" | "synergistic" | "synergism") | "multimodal" | "co-ingestion" | "interaction") + (("older adult" | "older adults") | "ageing" | "aging" |

Query name	Executed (UTC)	Matched	Retrieved	Query string
				"sarcopenia" | "healthspan"))
protein-restriction-mtor	2026-08-24T12:18:06 +00:00	994	994	(("protein restriction" | "low-protein diet" | "mTOR" | "IGF-1" | "amino acid restriction" | "methionine restriction") + ("longevity" | "lifespan" | "healthspan" | "ageing" | "aging") + (("human" | "humans") | ("older adult" | "older adults") | "protein intake" | "dietary protein"))
protein-restriction-mtor-1990	2026-08-24T12:37:00 +00:00	4,599	4,599	(("protein restriction" | "low-protein diet" | "mTOR" | "IGF-1" | "amino acid restriction" | "methionine restriction") + ("longevity" | "lifespan" | "healthspan" | "ageing" | "aging") + (("human" | "humans") | ("older adult" | "older adults") | "protein intake" | "dietary protein"))
protein-sarcopenia-dose	2026-08-24T12:18:10 +00:00	332	332	(("protein intake" | "dietary protein" | "protein supplementation" | "leucine" | ("essential amino acid" | "essential amino acids")) + ("sarcopenia" | "muscle mass" | "lean mass" | "muscle protein synthesis" | "frailty") + (("older adult" | "older adults") | "elderly" |

Query name	Executed (UTC)	Matched	Retrieved	Query string
				"ageing" | "aging") + (("recommendation" | "recommendations") | ("requirement" | "requirements") | "optimal" | "g/kg" | "dose" | "distribution"))
protein-sarcopenia-dose-1990	2026-08-24T12:37:06 +00:00	1,098	1,098	(("protein intake" | "dietary protein" | "protein supplementation" | "leucine" | ("essential amino acid" | "essential amino acids")) + ("sarcopenia" | "muscle mass" | "lean mass" | "muscle protein synthesis" | "frailty") + (("older adult" | "older adults") | "elderly" | "ageing" | "aging") + (("recommendation" | "recommendations") | ("requirement" | "requirements") | "optimal" | "g/kg" | "dose" | "distribution"))
protein-ukbiobank	2026-08-24T12:18:13 +00:00	56	56	(("UK Biobank" | "NHANES" | "prospective cohort") + ("protein intake" | "dietary protein") + ("mortality" | "cardiovascular"))
protein-ukbiobank-1990	2026-08-24T12:37:09 +00:00	155	155	(("UK Biobank" | "NHANES" | "prospective cohort") + ("protein intake" | "dietary protein") + ("mortality" | "cardiovascular"))
rct-exercise-clinical-pooled	2026-08-24T12:18:16 +00:00	177	177	(("exercise training" | "cardiac rehabilitation" | "exercise intervention" |

Query name	Executed (UTC)	Matched	Retrieved	Query string
				"supervised exercise") + ("heart failure" | "coronary artery disease" | "type 2 diabetes" | "chronic kidney disease" | ("cancer survivor" | "cancer survivors")) + ("mortality" | "all-cause" | "hospitalisation" | "hospitalization" | "MACE") + ("randomized controlled trial" | "controlled clinical trial" | "randomized" | "randomised" | "randomly assigned" | "placebo" | "trial") + ("meta-analysis" | "systematic review" | "meta-analysis" | "pooled analysis"))
rct-exercise-dose-pooled	2026-08-24T12:18:19 +00:00	97	97	(("exercise dose" | "exercise volume" | "dose-response" | "minimal dose" | "training frequency" | "weekly sets") + ("randomized" | "randomised") + ("muscle strength" | "cardiorespiratory fitness" | "VO2" | "functional capacity") + ("meta-analysis" | "systematic review" | "meta-analysis" | "pooled analysis"))
rct-exercise-older-outcomes	2026-08-24T12:18:28 +00:00	1,314	1,314	(("exercise training" | "physical activity intervention" | "structured exercise" | "resistance training" | "aerobic training") + ("mortality" | "major mobility disability" |

Query name	Executed (UTC)	Matched	Retrieved	Query string
				"disability" | "frailty" | "falls" | "hospitalisation" | "hospitalization") + (("older adult" | "older adults") | "elderly" | "aged") + ("randomized controlled trial" | "controlled clinical trial" | "randomized" | "randomised" | "randomly assigned" | "placebo" | "trial"))
rct-highprotein-renal-safety	2026-08-24T12:18:32+00:00	209	209	(("high-protein diet" | "high protein intake" | "protein supplementation") + ("renal function" | "kidney function" | "glomerular filtration" | "eGFR" | "albuminuria" | "creatinine" | "safety" | ("adverse event" | "adverse events")) + ("randomized controlled trial" | "controlled clinical trial" | "randomized" | "randomised" | "randomly assigned" | "placebo" | "trial"))
rct-lpd-ckd	2026-08-24T12:18:35+00:00	124	124	(("low-protein diet" | "protein restriction" | "very low protein diet" | ("ketoanalogue" | "ketoanalogues") | ("keto-analogue" | "keto-analogues")) + ("chronic kidney disease" | "CKD" | "dialysis" | "diabetic nephropathy" | "diabetic kidney disease") + ("mortality" | "progression" |

Query name	Executed (UTC)	Matched	Retrieved	Query string
				"end-stage" | "eGFR" | "proteinuria") + ("randomized controlled trial" | "controlled clinical trial" | "randomized" | "randomised" | "randomly assigned" | "placebo" | "trial"))
rct-multidomain	2026-08-24T12:18:39 +00:00	545	545	(("multidomain" | "multicomponent" | "lifestyle intervention") + ("exercise" | "physical activity") + ("protein" | "nutrition" | "diet") + ("frailty" | "sarcopenia" | "cognitive" | "disability" | "healthy ageing" | "healthy aging") + ("randomized controlled trial" | "controlled clinical trial" | "randomized" | "randomised" | "randomly assigned" | "placebo" | "trial"))
rct-protein-cvd	2026-08-24T12:18:43 +00:00	404	404	(("protein intake" | "dietary protein" | "protein supplementation") + ("heart failure" | "cardiovascular" | "blood pressure" | "LDL cholesterol" | "cardiac") + ("randomized controlled trial" | "controlled clinical trial" | "randomized" | "randomised" | "randomly assigned" | "placebo" | "trial"))
rct-protein-	2026-08-24T12:18:47	354	354	(("protein distribution" | "per-meal protein" |

Query name	Executed (UTC)	Matched	Retrieved	Query string
dose-response	+00:00			"protein dose" | "dose-response" | "g/kg" | "protein timing") + ("muscle protein synthesis" | "myofibrillar" | "nitrogen balance" | ("protein requirement" | "protein requirements") | "indicator amino acid oxidation") + ("randomized controlled trial" | "controlled clinical trial" | "randomized" | "randomised" | "randomly assigned" | "placebo" | "trial"))
rct-protein-obesity-leanmass	2026-08-24T12:18:50 +00:00	397	397	(("high-protein diet" | "protein intake" | "protein supplementation" | "energy-restricted") + ("obesity" | "obese" | "weight loss" | "bariatric" | "GLP-1" | "semaglutide" | "tirzepatide") + ("lean mass" | "fat-free mass" | "muscle mass" | "body composition" | "sarcopenic obesity") + ("randomized controlled trial" | "controlled clinical trial" | "randomized" | "randomised" | "randomly assigned" | "placebo" | "trial"))
rct-protein-older-function	2026-08-24T12:18:54 +00:00	691	691	(("protein supplementation" | "dietary protein" | "protein intake" | "whey protein" | "leucine" | ("essential amino acid" |

Query name	Executed (UTC)	Matched	Retrieved	Query string
				"essential amino acids")) + (("older adult" | "older adults") | "elderly" | "sarcopenia" | "frailty") + ("lean mass" | "muscle mass" | "muscle strength" | "grip strength" | "physical function" | "gait speed" | "SPPB") + ("randomized controlled trial" | "controlled clinical trial" | "randomized" | "randomised" | "randomly assigned" | "placebo" | "trial"))
rct-protein-plus-rt-pooled	2026-08-24T12:18:57 +00:00	64	64	(("protein supplementation" | "dietary protein") + ("resistance training" | "resistance exercise" | "strength training") + ("lean mass" | "muscle mass" | "strength" | "hypertrophy") + ("meta-analysis" | "systematic review" | "meta-analysis" | "pooled analysis") + ("randomized controlled trial" | "controlled clinical trial" | "randomized" | "randomised" | "randomly assigned" | "placebo" | "trial"))
rct-protein-t2dm	2026-08-24T12:19:01 +00:00	393	393	(("high-protein diet" | "protein intake" | "dietary protein" | "protein supplementation") + ("type 2 diabetes" | "insulin resistance" | "HbA1c" | "glycaemic

Query name	Executed (UTC)	Matched	Retrieved	Query string
				control" | "glycemic control" | "insulin sensitivity") + ("randomized controlled trial" | "controlled clinical trial" | "randomized" | "randomised" | "randomly assigned" | "placebo" | "trial"))
selfreport-error	2026-08-24T12:19:04 +00:00	253	253	((("self-report" | "self-reported" | "self-reporting") + ("misreporting" | "under-reporting" | "underreporting" | "measurement error" | "bias")) + ("dietary intake" | "energy intake" | "food frequency questionnaire" | "24-hour recall"))
sport-nutrition-longevity	2026-08-24T12:19:07 +00:00	446	446	(("sports nutrition" | "sport nutrition" | ("athlete" | "athletes") | ("masters athlete" | "masters athletes") | "exercise nutrition") + ("longevity" | "healthspan" | "lifespan" | "healthy ageing" | "healthy aging" | "biological age"))
sport-nutrition-longevity-1990	2026-08-24T12:37:14 +00:00	1,325	1,325	(("sports nutrition" | "sport nutrition" | ("athlete" | "athletes") | ("masters athlete" | "masters athletes") | "exercise nutrition") + ("longevity" | "healthspan" | "lifespan" | "healthy ageing" | "healthy

Query name	Executed (UTC)	Matched	Retrieved	Query string
				aging" | "biological age"))
steps-vilpa-mortality	2026-08-24T12:19:10 +00:00	128	128	(("steps per day" | "step count" | "daily steps" | "accelerometer-measured" | "device-measured physical activity" | "vigorous intermittent lifestyle physical activity" | "VILPA") + ("all-cause mortality" | "longevity" | "life expectancy" | "healthspan" | "cardiovascular mortality"))
steps-vilpa-mortality-1990	2026-08-24T12:37:16 +00:00	251	251	(("steps per day" | "step count" | "daily steps" | "accelerometer-measured" | "device-measured physical activity" | "vigorous intermittent lifestyle physical activity" | "VILPA") + ("all-cause mortality" | "longevity" | "life expectancy" | "healthspan" | "cardiovascular mortality"))

ClinicalTrials.gov — 10 queries, 31,239 matched, 31,239 retrieved

Query name	Executed (UTC)	Matched	Retrieved	Query string
ctg-cardiac-rehab	2026-08-24T12:05:44 +00:00	952	952	"cardiac rehabilitation" AND (exercise OR training)
ctg-exercise-mortality	2026-08-24T12:07:12 +00:00	14,742	14,742	(exercise OR "physical activity" OR training) AND ("all-cause mortality" OR survival OR mortality)

Query name	Executed (UTC)	Matched	Retrieved	Query string
ctg-highprotein-renal	2026-08-24T12:07:12+00:00	70	70	("high protein" OR "high-protein diet") AND ("kidney function" OR "renal function" OR "glomerular filtration" OR creatinine)
ctg-incretin-leanmass	2026-08-24T12:07:23+00:00	1,819	1,819	(semaglutide OR tirzepatide OR liraglutide OR "GLP-1" OR incretin) AND ("lean mass" OR "muscle mass" OR "fat-free mass" OR protein OR sarcopenia)
ctg-lpd-ckd	2026-08-24T12:07:24+00:00	80	80	("low protein diet" OR "protein restriction" OR "very low protein") AND ("chronic kidney disease" OR "renal insufficiency" OR nephropathy)
ctg-pa-dose	2026-08-24T12:07:37+00:00	2,453	2,453	("physical activity" OR exercise) AND ("dose response" OR "exercise dose" OR "steps per day" OR "moderate to vigorous")
ctg-protein-older	2026-08-24T12:08:02+00:00	2,855	2,855	(protein OR "amino acid") AND ("older adults" OR elderly OR sarcopenia OR frailty) AND ("lean mass" OR "muscle mass" OR "muscle strength" OR "physical function")
ctg-protein-rt	2026-08-24T12:08:04+00:00	454	454	(protein OR "protein supplementation") AND ("resistance training" OR "resistance exercise" OR "strength training") AND ("lean body mass" OR hypertrophy OR "muscle mass")
ctg-protein-t2dm	2026-08-24T12:08:37+00:00	6,233	6,233	(protein OR "high protein diet") AND ("type 2 diabetes" OR

Query name	Executed (UTC)	Matched	Retrieved	Query string
				`"diabetes mellitus")`
`ctg-sarcopenia-intervention`	2026-08-24T12:08:42+00:00	1,581	1,581	`sarcopenia AND (exercise OR protein OR nutrition OR supplementation)`

Table S2. All extracted effect estimates

All **8,304** effect estimates with 95% confidence intervals recovered by the deterministic extraction pipeline, each with its ±180-character provenance window, source PMID, journal, year, DOI, the query that retrieved it and whether the source text was full text or abstract. The two measure families are kept separate throughout and are distinguished by the `measure_family` column: **6,005 ratio** estimates (HR, RR, OR, IRR) and **2,299 mean-difference** estimates (MD, SMD, WMD).

Supplied as **`TableS2_all_estimates.csv`** rather than as a Word table: at 8,304 rows it belongs in a spreadsheet, and rendering it here would produce several hundred unusable pages. Note that this is the full extraction output, not the analysis set — only the estimates in Table S3 entered a pooled stratum, and each of those was read in its source context by an author first (Section 2.4).

Table S3. Curated analysis set

Every estimate admitted to a pooled stratum, with the contrast definition it satisfies, the population and the setting. Each was read in its full-text context before admission; the rejected candidates and the reason for each rejection are recorded in the comment block of `notes/build_curated.py`, supplied in File S1.

stratum	measure	estimate	ci low	ci high	pmid	year	contrast	population	setting
PA_guideline_vs_inactive	HR	0.86	0.77	0.95	41165888	2025	>=600 vs <600 MET-min/wk	diabetes/prediabetes	clinical
PA_guideline_vs_inactiv	HR	0.84	0.72	0.98	40638069	2025	meets PA guidelines	cancer patients	clinical

stratum	measure	estimate	ci low	ci high	pmid	year	contrast	population	setting
e							vs no PA	(Lifelines)	
PA_guideline_vs_inactive	HR	0.83	0.78	0.87	40690774	2025	regularly active vs inactive	adults with diabetes	clinical
PA_guideline_vs_inactive	HR	0.82	0.7	0.95	37316812	2023	>=50 vs 0 MET-h/wk MVPA	Korean adults (KNHANES)	general
PA_guideline_vs_inactive	HR	0.81	0.7	0.95	39077609	2022	500-999 MET-min/wk (guideline range) vs inactive	older adults with heart failure	clinical
PA_guideline_vs_inactive	HR	0.78	0.7	0.86	41579289	2026	full adherence to PA recommendation	long-term cancer survivors	clinical
PA_guideline_vs_inactive	HR	0.77	0.69	0.85	41360828	2025	highest vs inactive quartile of total PA	Chinese adults	general
PA_gu	HR	0.74	0.6	0.9	36902	2023	>=150	non-	clinic

stratum	measure	estimate	ci low	ci high	pmid	year	contrast	population	setting
ideline_vs_inactive					707		min/wk total PA vs below	alcoholic fatty liver disease	al
PA_guideline_vs_inactive	HR	0.729	0.64	0.831	40269864	2025	meets MVPA recommendation vs not	stroke survivors	clinical
PA_guideline_vs_inactive	HR	0.72	0.59	0.88	39283728	2025	currently active vs sedentary	community-dwelling adults	general
PA_guideline_vs_inactive	HR	0.71	0.63	0.8	42296133	2026	>=150 MET-min/wk vs below	cardiac patients	clinical
PA_guideline_vs_inactive	HR	0.69	0.67	0.71	39968650	2026	highest vs lowest quintile of total PA	Chinese adults, 47,281 deaths	general
PA_guideline_vs_inactive	HR	0.68	0.58	0.81	28715883	2017	meets PA guidelines vs inactive	Korean adults, 8-y prospective	general
PA_guideline	HR	0.66	0.43	0.99	33083530	2020	>6.9 MET-	breast cance	clinical

stratum	measure	estimate	ci low	ci high	pmid	year	contrast	population	setting
e_vs_inactive							h/wk MVPA (active) vs inactive	r survivors	
PA_guideline_vs_inactive	HR	0.6	0.47	0.76	40308924	2025	>=600 vs <600 MET-min/wk	adults with depression	clinical
PA_guideline_vs_inactive	HR	0.53	0.34	0.84	27730174	2016	>=150 min/wk MVPA vs inactive	after COPD hospitalization	clinical
RT_vs_none	HR	0.87	0.81	0.95	42230125	2026	90-119 min/wk RT vs none	US adults, 30-y follow-up	general
RT_vs_none	HR	0.67	0.48	0.94	37316812	2023	>=1 vs 0 d/wk muscle-strengthening	Korean adults (KNHANES)	general
protein_total_high_vs_low	HR	1.22	0.89	1.67	36854962	2023	Q5 vs Q1 total protein (model 4)	Iranian adults, 11.3 y	general

stratum	measure	estimate	ci low	ci high	pmid	year	contrast	population	setting
protein_total_high_vs_low	HR	1.14	0.75	1.72	35445069	2022	Q5 vs Q1 total protein (g/kg/d)	Korean adults with CKD stage 3-5 (KoGES)	clinical
protein_total_high_vs_low	HR	1.12	1.01	1.25	32076944	2020	Q4 vs Q1 total protein (% energy)	Rotterdam Study	general
protein_total_high_vs_low	HR	0.6	0.39	0.94	38363105	2024	Q4 (>=107 g/d) vs Q1 (<58 g/d)	adults with comorbidity	clinical
protein_total_high_vs_low	HR	0.44	0.22	0.9	37558986	2023	Q4 vs Q1 total protein	older adults, Kawasaki	general
protein_plant_high_vs_low	HR	0.91	0.86	0.96	33624505	2021	Q5 vs Q1 plant protein	Women's Health Initiative, n=102,521, 25,976 deaths	general
protei	HR	0.73	0.58	0.93	39543	2025	highe	type 2	clinic

stratum	measure	estimate	ci low	ci high	pmid	year	contrast	population	setting
n_plant_high_vs_low					271		st vs lowest plant protein	diabetes	al
protein_plant_high_vs_low	HR	0.65	0.45	0.93	42541632	2026	Q4 vs Q1 plant protein	SUN cohort	general
protein_plant_high_vs_low	HR	0.64	0.46	0.91	36854962	2023	Q5 vs Q1 plant protein	Iranian adults, 11.3 y	general
protein_animal_high_vs_low	HR	1.52	1.13	2.05	36854962	2023	Q5 vs Q1 animal protein (model 4)	Iranian adults, 11.3 y	general
protein_animal_high_vs_low	HR	0.94	0.7	1.26	42541632	2026	Q4 vs Q1 animal protein	SUN cohort	general
protein_veryhigh_vs_adequate	HR	1.39	1.17	1.65	41260173	2026	>=1.8 vs <1.8 g/kg BW/d	UK Biobank subset, PSM	general

30 rows. Generated from the curated analysis set supplied in File S1; the rejected candidates and the reason for each rejection are recorded in the extraction code in the same file.

Table S4. Population-stratified evidence, absolute intake units

The 23 estimates across 10 clinical populations that entered the stratified analysis of Section 3.6.

How this table relates to Table 3 of the main text. The two are not the same set and are not intended to be. This table is the population tranche of the extraction, restricted to estimates in absolute intake units and admitted by the rule of Section 2.7; it carries every admitted estimate, including several per study. Table 3 is the main text's population-by-population summary, which also draws on the randomized, mediator and malnutrition tranches and therefore contains studies absent from here — among them the maintenance-hemodialysis cohort, the randomized low-protein-diet and enteral-protein trials, and the disease-related-malnutrition cohort — while three studies admitted here (PMIDs 35445069, 37178773, 37558986) are not carried into Table 3 because their populations are represented there by a better-matched estimate. Neither table enters a pooled estimate. Only estimates expressed in g/kg actual body weight per day, g/kg ideal or adjusted body weight per day, or g/day were admitted; percentage-of-energy contrasts are excluded throughout (Section 2.7). The direction of association is recorded explicitly because it is not constant across populations, and no pooling was attempted within a stratum.

population	pmid	year	design	n	contrast absolute	outcome	measure	estimate	ci low	ci high	direction	note
healthy older adults	39770971	2024	meta-analysis	—	<0.8 vs >=1.2 g/kg/d	sarcopenia	OR	1.87	1.46	2.38	higher intake, lower risk	low intake is the risk state
healthy older adults	39770971	2024	meta-analysis	—	0.8-1.2 vs >=1.2 g/kg/d	sarcopenia	OR	1.3	1.11	1.52	higher intake, lower	even 'adequate' 0.8-

population	pmid	year	design	n	contrast absolute	outcome	measure	estimate	ci low	ci high	direction	note
											risk	1.2 is worse than n >= 1.2
healthy older adults	39770971	2024	meta-analysis	—	<0.8 vs >=1.2 g/kg/d (men)	sarcopenia	OR	2.01	1.36	2.99	higher intake, lower risk	larger in men
healthy older adults	39770971	2024	meta-analysis	—	<0.8 vs 0.8-1.2 g/kg/d	low handgrip strength	OR	1.31	1.03	1.65	higher intake, lower risk	
healthy older adults	41204398	2025	multi-cohort	—	>=1.0 g/kg adj BW/d, age 85+	pre-frail -> frail	HR	0.63	0.44	0.9	higher intake, lower risk	highest category HR 0.44 (0.25-0.77)
healthy older adul	41204398	2025	multi-cohort	—	per 1 g/kg adj BW/d	non-frail -> pre-	HR	0.96	0.94	0.98	higher intake, lower er	

population	pmid	year	design	n	contrast absolute	outcome	measure	estimate	ci low	ci high	direction	note
ts					increment	frail					risk	
healthy older adults	41204398	2025	multi-cohort	—	per 1 g/kg adj BW/d increment	recovery pre-frail -> non-frail	HR	1.03	1.01	1.06	higher intake, higher recovery rate	HR>1 is GOOD here (recovery)
healthy older adults	37558986	2023	cohort	—	Q4 vs Q1 total protein (g/d)	all-cause mortality	HR	0.44	0.22	0.9	higher intake, lower risk	Kawasaki Aging & Wellbeing
CKD stage 3–5 (g/kg)	35445069	2022	cohort	—	>1.2 vs <0.8 g/kg/d	all-cause mortality	HR	1.06	0.71	1.57	no association	no mortality signal on the g/kg metric
obesity	42535295	2026	cohort, prevalen	14,816	higher total	sarcopenic obe	OR	0.14	0.08	0.2	higher intake,	EPIC-Norfolk,

population	pmid	year	design	n	contrast absolute	outcome	measure	estimate	ci low	ci high	direction	note
			t outcome at follow-up		protein (g/kg BW/d), continuous, per 1 g/kg/d; energy omitted for collinearity	sity					lower risk	UK, 39–79 y; sarcopenia = sex-specific 20th percentile of FFM/BMI, obesity = BMI ≥30, so body size is in both the outcome and

population	pmid	year	design	n	contrast absolute	outcome	measure	estimate	ci low	ci high	direction	note
												the exposure denominator (Section 4.2)
obesity	42535295	2026	cohort, prevalent outcome at follow-up	14,816	higher animal protein (g/kg BW/d), continuous, per 1 g/kg/d; energy-adjusted	sarcopenic obesity	OR	0.58	0.53	0.62	higher intake, lower risk	EPIC-Norfolk, UK, 39–79 y; sarcopenia = sex-specific 20th percentile of FFM/BMI, obesity

population	pmid	year	design	n	contrast absolute	outcome	measure	estimate	ci low	ci high	direction	note
												= BMI ≥30, so body size is in both the outcome and the exposure denominator (Section 4.2)
obesity	42535295	2026	cohort, prevalent outcome at follow-up	14,816	higher plant protein (g/kg BW/d), con	sarcopenic obesity	OR	0.46	0.41	0.52	higher intake, lower risk	EPIC-Norfolk, UK, 39–79 y; sarcopenia

population	pmid	year	design	n	contrast absolute	outcome	measure	estimate	ci low	ci high	direction	note
					tinuous, per 1 g/kg/d; energy-adjusted							= sex-specific 20th percentile of FFM/BMI, obesity = BMI ≥30, so body size is in both the outcome and the exposure denominat

population	pmid	year	design	n	contrast absolute	outcome	measure	estimate	ci low	ci high	direction	note
												or (Section 4.2)
CVD - hospitalized HF/cardiac	38705583	2024	cohort	—	lowest vs upper quartiles of DPI (g/kg IBW/d)	death + HF readmission (2 y)	HR	2.03	1.08	3.82	lower intake, higher risk	the deficiency end is the danger
CVD - hospitalized HF/cardiac	38705583	2024	cohort	—	per 0.26 g/kg IBW/d DECREASE	death + HF readmission	HR	1.32	1.1	1.71	lower intake, higher risk	linear, p-nonlinearity 0.90
CVD - community, very high intake	41260173	2026	cohort (PSM)	19420	>=1.8 vs <1.8 g/kg BW/d (ref mean 1.27)	all-cause mortality	HR	1.39	1.17	1.65	very high intake, higher risk	disputed; see Section 4.2 (denominator) and

population	pmid	year	design	n	contrast absolute	outcome	measure	estimate	ci low	ci high	direction	note
												Table S7
CVD - community, very high intake	41260173	2026	cohort (PSM)	19420	>=1.8 vs <1.8 g/kg BW/d	MACE	HR	1.21	1.02	1.44	very high intake, higher risk	primary endpoint
CVD - community, very high intake	41260173	2026	cohort (PSM)	19420	>=1.8 vs <1.8 g/kg BW/d, age <55	MACE	HR	0.75	0.51	1.11	no association under 55	p-interaction 0.003
CKD non-dialysis	42048078	2026	cohort (PSM), 15 y	1441	<1.0 vs >=1.0 g/kg/d nDPI	kidney failure/dialysis/death	HR	0.77	0.62	0.97	lower intake, lower risk	lower intake associated with lower risk
CKD non-dial	42048078	2026	cohort (PSM), 15 y	1441	<1.0 vs >=1.0 g/kg/d nDP	dialysis initiation	HR	0.65	0.42	0.99	lower intake, lower	

population	pmid	year	design	n	contrast absolute	outcome	measure	estimate	ci low	ci high	direction	note
ysis					l						risk	
CKD stage 4-5	37178773	2023	cohort	—	non-supplemented LPD <=0.5 g/kg/d	RRT initiation + all-cause mortality	HR	0.656	0.438	0.984	lower intake, lower risk	
CKD + T2DM	40618176	2025	cohort (PSM) + NHANES	820 / 1723	DPI sUCR <=1.0 g/kg/d	composite renal progression	HR	0.29	0.13	0.67	lower intake, lower risk	CKD 3-4: <0.8 g/kg/d best
CKD, age >65	30428524	2018	cohort	—	moderate vs low protein intake	all-cause mortality	HR	0.14	0.02	0.69	higher intake, lower risk	age reverses the sign within CKD
CKD, all ages	30428524	2018	cohort	—	moderate vs low protein inta	all-cause mortality	HR	0.29	0.07	0.94	higher intake, lower risk	whole cohort; in age <=65 no

population	pmid	year	design	n	contrast absolute	outcome	measure	estimate	ci low	ci high	direction	note
					ke							significant association

23 rows across 10 populations. The 285 candidate estimates the extraction pipeline located in absolute units, before contextual reading and admission, are in `notes/protein_by_population.csv` and ship with File S1.

Table S5. Comparator table of published meta-analyses

Published pooled analyses bearing on the same questions, held in a separate layer from our primary-cohort pooling so that no study is counted twice (Section 2.5).

pmid	year	journal	exposure	outcome	measure	estimate	ci low	ci high	i2	cohorts	participants
38201983	2024	Nutrients	total protein highest vs lowest	all-cause mortality	RR	0.97	0.93	1.02	68.7	25	666129
38201983	2024	Nutrients	total protein highest vs lowest	CVD morbidity	RR	0.88	0.82	0.94	38.8	15	286075

pmid	year	journal	exposure	outcome	measure	estimate	ci low	ci high	i2	cohorts	participants
38201983	2024	Nutrients	animal protein highest vs lowest	all-cause mortality	RR	1.04	0.97	1.12	68.9	32	411303
38201983	2024	Nutrients	plant protein highest vs lowest	all-cause mortality	RR	0.92	0.87	0.98	43.8	37	962646
38201983	2024	Nutrients	plant protein highest vs lowest	CVD mortality	RR	0.85	0.78	0.93	19.5	37	962646
32699048	2020	BMJ	total protein highest vs lowest	all-cause mortality	HR	0.94	0.89	0.99	58.4	NA	715128
32699048	2020	BMJ	plant protein	all-cause mort	HR	0.92	0.87	0.97	57.5	NA	715128

pmid	year	journal	exposure	outcome	measure	estimate	ci low	ci high	i2	cohorts	participants
			highest vs lowest	ality							
32699048	2020	BMJ	plant protein highest vs lowest	CVD mortality	HR	0.88	0.80	0.96	63.7	NA	715128
32451273	2020	NMCD	plant protein highest vs lowest	all-cause mortality	RR	0.92	0.88	0.96	NA	12	NA
32451273	2020	NMCD	plant protein per 3% energy increment	all-cause mortality	RR	0.97	0.94	0.99	NA	12	NA

10 rows. Generated from the comparator table supplied in File S1.

Table S6. Reference number → PMID mapping

Every citation resolves to a PubMed record, except the two reports without one (WHO/FAO/UNU 2007, identified by its WHO repository handle, and EFSA 2012, identified by its DOI), which carry their key in place of a PMID. Each reference was verified against its PubMed record, Crossref record or repository record.

Ref #	PMID	Citation
1	33239350	Bull, F.C.; Al-Ansari, S.S.; Biddle, S.; Borodulin, K.; Buman, M.P.; Cardon, G.; Carty, C.; Chaput, J.P.; Chastin, S.; Chou, R.; e
2	WHO_FAO_UNU_2007	World Health Organization; Food and Agriculture Organization of the United Nations; United Nations University. *Protein and Amino
3	EFSA_NDA_2012_protein	EFSA Panel on Dietetic Products, Nutrition and Allergies (NDA). Scientific Opinion on Dietary Reference Values for protein. *EFSA
4	23867520	Bauer, J.; Biolo, G.; Cederholm, T.; Cesari, M.; Cruz-Jentoft, A.J.; Morley, J.E.; Phillips, S.; Sieber, C.; Stehle, P.; Teta, D.;
5	24814383	Deutz, N.E.; Bauer, J.M.; Barazzoni, R.; Biolo, G.; Boirie, Y.; Bosy-Westphal, A.; Cederholm, T.; Cruz-Jentoft, A.; Krznariç, Z.;
6	30005900	Volkert, D.; Beck, A.M.; Cederholm, T.; Cruz-

Ref #	PMID	Citation
		Jentoft, A.; Goisser, S.; Hooper, L.; Kiesswetter, E.; Maggio, M.; Raynaud-Simon, A.;
7	32829751	Ikizler, T.A.; Burrowes, J.D.; Byham-Gray, L.D.; Campbell, K.L.; Carrero, J.J.; Chan, W.; Fouque, D.; Friedman, A.N.; Ghaddar, S.;
8	33567185	Wilding, J.P.H.; Batterham, R.L.; Calanna, S.; Davies, M.; Van Gaal, L.F.; Lingvay, I.; McGowan, B.M.; Rosenstock, J.; Tran, M.T.D
9	39431379	Jiao, R.; Lin, C.; Cai, X.; Wang, J.; Wang, Y.; Lv, F.; Yang, W.; Ji, L. Characterizing body composition modifying effects of a gl
10	25844730	Arem, H.; Moore, S.C.; Patel, A.; Hartge, P.; Berrington de Gonzalez, A.; Visvanathan, K.; Campbell, P.T.; Freedman, M.; Weiderpas
11	26960445	Phillips, S.M.; Chevalier, S.; Leidy, H.J. Protein “requirements” beyond the RDA: implications for optimizing health. *Appl Physio
12	28698222	Morton, R.W.; Murphy, K.T.; McKellar, S.R.;

Ref #	PMID	Citation
		Schoenfeld, B.J.; Henselmans, M.; Helms, E.; Aragon, A.A.; Devries, M.C.; Banfield, L.
13	30312372	Cruz-Jentoft, A.J.; Bahat, G.; Bauer, J.; Boirie, Y.; Bruyère, O.; Cederholm, T.; Cooper, C.; Landi, F.; Rolland, Y.; Sayer, A.A.;
14	31171417	Cruz-Jentoft, A.J.; Sayer, A.A. Sarcopenia. *Lancet* **2019**, *393*, 2636-2646. https://doi.org/10.1016/S0140-6736(19)31138-9
15	39389333	Polo-López, A.; Calatayud, J.; López-Bueno, L.; Núñez-Cortés, R.; Andersen, L.L.; López-Bueno, R. Dose-response association of an
16	42389704	Macdonald, C. Beyond the bare minimum: the case for revised physical activity guidelines and protein intake recommendations that m
17	29099919	Stamatakis, E.; Lee, I.M.; Bennie, J.; Freeston, J.; Hamer, M.; O’Donovan, G.; Ding, D.; Bauman, A.; Mavros, Y. Does Strength-Prom
18	42535295	Baabbad, T.; Aceves-

Ref #	PMID	Citation
		Martins, M.; Horgan, G.; Myint, P.; de Roos, B. Dietary intake of total protein, animal- and plant-based prote
19	27730174	Moy, M.L.; Gould, M.K.; Liu, I.A.; Lee, J.S.; Nguyen, H.Q. Physical activity assessed in routine care predicts mortality after a C
20	41165888	Wu, N.; Fu, J. Association of biological age acceleration with mortality in adults with diabetes or prediabetes: a mediating role
21	42296133	Li, Y.; Yang, L.; Lu, J.; Wang, Y.; Cheng, L.; Zuo, Z.; Fu, Z.; Chen, P. Association Between Comprehensive Health Assessment and C
22	36902707	Kim, D.; Wijarnpreecha, K.; Dennis, B.B.; Cholankeril, G.; Ahmed, A. Types of Physical Activity in Nonalcoholic Fatty Liver Diseas
23	40308924	Xiao, J.; Dong, X.; Ding, M.; Yang, Q.; Kong, T. Association between physical activity and all-cause mortality in patients with de
24	24548571	IntHout, J.; Ioannidis, J.P.; Borm, G.F. The

Ref #	PMID	Citation
		Hartung-Knapp-Sidik-Jonkman method for random effects meta-analysis is straightforwar
25	21784880	Sterne, J.A.; Sutton, A.J.; Ioannidis, J.P.; Terrin, N.; Jones, D.R.; Lau, J.; Carpenter, J.; Rücker, G.; Harbord, R.M.; Schmid, C
26	30067315	Langan, D.; Higgins, J.P.T.; Jackson, D.; Bowden, J.; Veroniki, A.A.; Kontopantelis, E.; Viechtbauer, W.; Simmonds, M. A compariso
27	25880989	von Hippel, P.T. The heterogeneity statistic I(2) can be biased in small meta-analyses. *BMC Med Res Methodol* **2015**, *15*, 35.
28	28378395	Jackson, D.; Turner, R. Power analysis for random-effects meta-analysis. *Res Synth Methods* **2017**, *8*, 290-302. https://doi.o
29	33068715	Garritty, C.; Gartlehner, G.; Nussbaumer-Streit, B.; King, V.J.; Hamel, C.; Kamel, C.; Affengruber, L.; Stevens, A. Cochrane Rapid
30	38320771	Garritty, C.; Hamel, C.; Trivella, M.; Gartlehner, G.; Nussbaumer-Streit, B.; Devane, D.; Kamel,

Ref #	PMID	Citation
		C.; Griebler, U.; King, V.J. Upda
31	37316812	Cho, Y.; Jang, H.; Kwon, S.; Oh, H. Aerobic, muscle-strengthening, and flexibility physical activity and risks of all-cause and ca
32	41360828	Wang, F.; Wang, Y.; Wang, K.; Wu, S.; Chen, Y.; Li, Y.; Chen, S.; Gao, X. Dose–response relationship between physical activity and
33	39283728	Moshkovits, Y.; Chetrit, A.; Dankner, R. Self-reported physical activity properties and 20-year all-cause and cardiovascular morta
34	39968650	Ke, Y.; Shi, K.; Bennett, D.A.; Lv, J.; Sun, D.; Pei, P.; Du, H.; Chen, Y.; Yang, L.; Zheng, X.; et al. Associations of total, dom
35	28715883	Kim, J. Association between Meeting Physical Activity Guidelines and Mortality in Korean Adults: An 8-year Prospective Study. *J E
36	40638069	Bos, M.D.; Sidorenkov, G.; Vinke, P.C.; Ho, C.S.L.; Meyer, N.H.; Bockhorn, M.; de Groot, D.J.A.; Klaase, J.M.; Hoogwater, F.J.H.;
37	40690774	Wu, Z.; Sheng, C.; Guo,

Ref #	PMID	Citation
		Z.; Zheng, Y.; Zheng, D.; Li, X.; Guo, X.; Li, H. Association of Weekend Warrior and Other Physical Activi
38	39077609	Yu, G.I.; Yang, P.S.; Kim, M.H.; Jin, M.N.; Jang, E.; Yu, H.T.; Kim, T.H.; Pak, H.N.; Lee, M.H.; Joung, B. Association between Phy
39	41579289	Zhu, C.; Thong, M.S.Y.; Doege, D.; Koch-Gallenkamp, L.; Bertram, H.; Eberle, A.; Holleczek, B.; Nennecke, A.; Waldmann, A.; Zeißig
40	40269864	Zhu, Y.; Chen, B.; Qin, M.; Yang, J.; Hu, M.; Zeng, J.; Fan, M.; Wang, K.; Chang, L.; Xu, S. Joint effects of physical activity an
41	33083530	Cao, Y.; Baumgartner, K.B.; Visvanathan, K.; Boone, S.D.; Baumgartner, R.N.; Connor, A.E. Ethnic and biological differences in the
42	38901742	Rodríguez-Gutiérrez, E.; Torres-Costoso, A.; Del Pozo Cruz, B.; de Arenas-Arroyo, S.N.; Pascual-Morena, C.; Bizzozero-Peroni, B.;
43	39551081	Shimoda, T.; Tomida, K.; Nakajima, C.; Kawakami, A.;

Ref #	PMID	Citation
		Shimada, H. Dose-Response Relationships of Daily Steps with Disability Incide
44	41612409	Koemel, N.A.; Ahmadi, M.N.; Biswas, R.K.; Thøgersen-Ntoumani, C.; Teixeira-Pinto, A.; Chow, C.K.; Harezlak, J.; Stamatakis, E. Vig
45	42230125	Zhang, Y.; Lee, D.H.; Rezende, L.F.M.; Ma, Y.; Giovannucci, E. Long-term resistance training with all-cause and cause-specific mor
46	38201983	Ma, Y.; Zheng, Z.; Zhuang, L.; Wang, H.; Li, A.; Chen, L.; Liu, L. Dietary Macronutrient Intake and Cardiovascular Disease Risk an
47	32699048	Naghshi, S.; Sadeghi, O.; Willett, W.C.; Esmaillzadeh, A. Dietary intake of total, animal, and plant proteins and risk of all caus
48	32451273	Qi, X.X.; Shen, P. Associations of dietary protein intake with all-cause, cardiovascular disease, and cancer mortality: A systemat
49	33624505	Sun, Y.; Liu, B.; Snetselaar, L.G.; Wallace, R.B.; Shadyab, A.H.;

Ref #	PMID	Citation
		Kroenke, C.H.; Haring, B.; Howard, B.V.; Shikany, J.M.; Valdivie
50	27479196	Song, M.; Fung, T.T.; Hu, F.B.; Willett, W.C.; Longo, V.D.; Chan, A.T.; Giovannucci, E.L. Association of Animal and Plant Protein
51	32076944	Chen, Z.; Glisic, M.; Song, M.; Aliahmad, H.A.; Zhang, X.; Moumdjian, A.C.; Gonzalez-Jaramillo, V.; van der Schaft, N.; Bramer, W.
52	35032733	Wang, J.; Luo, P.; Yang, Y.; Lin, Z.; Wen, Z.; Li, Y.; Huang, Y.; Yang, S.; Lu, Y.; Kong, Y.; et al. Dietary protein intake and th
53	39770971	Han, M.; Woo, K.; Kim, K. Association of Protein Intake with Sarcopenia and Related Indicators Among Korean Older Adults: A System
54	41204398	Mendonça, N.; Rodrigues, A.M.; Henriques, A.R.; Canhão, H.; Raimundo, A.; Celis-Morales, C. Protein intake and transitions between
55	38705583	Katano, S.; Yano, T.; Yamano, K.; Numazawa, R.; Nagaoka, R.; Honma, S.; Fujisawa, Y.; Ohori,

Ref #	PMID	Citation
		K.; Kouzu, H.; Kunihara, H.; et al. A
56	41260173	Huang, C.; Yu, Y.; Liang, W.; Li, J.; Wang, Y.; Wei, F.; Xu, T.; Ning, Y.; Zhen, Z.; Liu, J.; et al. Associations between High Pro
57	42048078	Beberashvili, I.; Baevsky, T.; Shmuel, D.; Yoles, I.; Rosen, M.; Efrati, S. Protein Intake and Kidney Outcomes in Nondialysis Chro
58	40618176	Liu, D.; Li, Q.; Jia, R.; He, W.; Zhao, X.; Pan, M.; Chen, X.; Wang, Y.; Zhu, Y.; Hou, H.; et al. Type 2 diabetes mellitus with ch
59	30428524	Watanabe, D.; Machida, S.; Matsumoto, N.; Shibagaki, Y.; Sakurada, T. Age Modifies the Association of Dietary Protein Intake with
60	36594428	Jiang, S.; Fang, J.; Li, W. Protein restriction for diabetic kidney disease. *Cochrane Database Syst Rev* **2023**, *1*, CD014906.
61	42048978	Summers, M.J.; Bels, J.L.M.; Karahalios, A.; Gunaratne, R.K.; Presneill, J.J.; Plummer, M.P.; Lee, Z.Y.; Heyland, D.K.; Mesotten,

Ref #	PMID	Citation
62	40647234	Pérez-López, P.; López-Gómez, J.J.; Izaola-Jauregui, O.; González-Gutiérrez, J.; Estévez-Asensio, L.; Pérez-Mellen, I.; López-Andr
63	35944294	Kosoku, A.; Iwai, T.; Ishihara, T.; Kabei, K.; Nishide, S.; Maeda, K.; Hanayama, Y.; Ishimura, E.; Uchida, J. Influence of protein
64	28420212	Kang, S.S.; Chang, J.W.; Park, Y. Nutritional Status Predicts 10-Year Mortality in Patients with End-Stage Renal Disease on Hemodi
65	37718370	Schulze, M.B.; Haardt, J.; Amini, A.M.; Kalotai, N.; Lehmann, A.; Schmidt, A.; Buyken, A.E.; Egert, S.; Ellinger, S.; Kroke, A.; e
66	31731672	Fan, M.; Li, Y.; Wang, C.; Mao, Z.; Zhou, W.; Zhang, L.; Yang, X.; Cui, S.; Li, L. Dietary Protein Consumption and the Risk of Typ
67	39796517	Pickering, R.T.; Yiannakou, I.; Lara-Castor, L.; Bradlee, M.L.; Singer, M.R.; Moore, L.L. Individual and Joint Associations Betwee
68	29983752	Ke, Q.; Chen, C.; He, F.; Ye, Y.; Bai, X.; Cai, L.; Xia, M. Association

Ref #	PMID	Citation
		between dietary protein intake and type 2 diabetes varies
69	27299701	Satija, A.; Bhupathiraju, S.N.; Rimm, E.B.; Spiegelman, D.; Chiuve, S.E.; Borgi, L.; Willett, W.C.; Manson, J.E.; Sun, Q.; Hu, F.B
70	20888279	Pounis, G.D.; Tyrovolas, S.; Antonopoulou, M.; Zeimbekis, A.; Anastasiou, F.; Bountztiouka, V.; Metallinos, G.; Gotsis, E.; Liolio
71	34059041	Mirmiran, P.; Bahadoran, Z.; Gaeini, Z.; Azizi, F. Habitual intake of dietary L-arginine in relation to risk of type 2 diabetes: a
72	25979490	Pasin, G.; Comerford, K.B. Dairy foods and dairy proteins in the management of type 2 diabetes: a systematic review of the clinica
73	38320463	Tabaeifard, R.; Moradi, M.; Arzhang, P.; Azadbakht, L. Association between protein intake and risk of gestational diabetes mellitu
74	38215681	Hajhashemy, Z.; Bagherniya, M.; Sadeghi, O.; Askari, G. The relation of dietary

Ref #	PMID	Citation
		protein intake before and during the pregnancy wit
75	37721649	Luo, T.; Chen, H.; Wei, H.; Yang, Y.; Wei, F.; Chen, W. Dietary protein in early pregnancy and gestational diabetes mellitus: a pr
76	39913952	Omoto, T.; Kyozuka, H.; Murata, T.; Fukuda, T.; Isogami, H.; Okoshi, C.; Yasuda, S.; Yamaguchi, A.; Sato, A.; Ogata, Y.; et al. Re
77	33028588	Stensvold, D.; Viken, H.; Steinshamn, S.L.; Dalen, H.; Støylen, A.; Loennechen, J.P.; Reitlo, L.S.; Zisko, N.; Bækkerud, F.H.; Tar
78	34741536	Dibben, G.; Faulkner, J.; Oldridge, N.; Rees, K.; Thompson, D.R.; Zwisler, A.D.; Taylor, R.S. Exercise-based cardiac rehabilitatio
79	38451843	Molloy, C.; Long, L.; Mordi, I.R.; Bridges, C.; Sagar, V.A.; Davies, E.J.; Coats, A.J.; Dalal, H.; Rees, K.; Singh, S.J.; et al. E
80	30255969	Taylor, R.S.; Walker, S.; Smart, N.A.; Piepoli, M.F.; Warren, F.C.; Ciani, O.; O’Connor, C.; Whellan, D.; Keteyian, S.J.; Coats, A
81	30215179	Xia, T.L.; Huang, F.Y.;

Ref #	PMID	Citation
		Peng, Y.; Huang, B.T.; Pu, X.B.; Yang, Y.; Chai, H.; Chen, M. Efficacy of Different Types of Exercise-Base
82	34673936	Kirwan, R.P.; Mazidi, M.; Rodríguez García, C.; Lane, K.E.; Jafari, A.; Butler, T.; Perez de Heredia, F.; Davies, I.G. Protein int
83	36057893	Tagawa, R.; Watanabe, D.; Ito, K.; Otsuyama, T.; Nakayama, K.; Sanbongi, C.; Miyachi, M. Synergistic Effect of Increased Total Pro
84	37907227	Kwon, H.E.; Ko, N.; Yuk, D.; Choi, S.W.; Koh, S.E. Improved Muscle Mass and Function With Protein Supplementation in Older Adults
85	40200135	Zhang, L.; Liu, G.; Huang, X.; He, F. Effects of protein supplementation on muscle mass, muscle strength, and physical performance
86	42289790	Teixeira Silva, P.R.; Santos, P.; de Miranda Silva Nogueira, P.A.; Nogueira, I.D.B.; Vieira, R.P.; Nascimento, M.F.; Tuesta, M.; S
87	30403710	Yan, B.; Su, X.; Xu, B.; Qiao, X.; Wang, L. Effect of diet protein restriction on

Ref #	PMID	Citation
		progression of chronic kidney disease: A systema
88	33118160	Hahn, D.; Hodson, E.M.; Fouque, D. Low protein diets for non-diabetic adults with chronic kidney disease. *Cochrane Database Syst
89	34967847	Bellizzi, V.; Signoriello, S.; Minutolo, R.; Di Iorio, B.; Nazzaro, P.; Garofalo, C.; Calella, P.; Chiodini, P.; De Nicola, L. No
90	30935396	Li, X.F.; Xu, J.; Liu, L.J.; Wang, F.; He, S.L.; Su, Y.; Dong, C.P. Efficacy of low-protein diet in diabetic nephropathy: a meta-a
91	39214675	Terada, T.; Pap, R.; Thomas, A.; Wei, R.; Noda, T.; Visintini, S.; Reed, J.L. Effects of muscle strength training combined with ae
92	40671711	Hua-Rui, L.; Shouliang, H.; Zhengze, Y.; Ning, J.; Peihua, L.; Yifei, Z.; Fenglin, P. Optimal dose of resistance training to impro
93	38830547	Hettiarachchi, J.; Reijnierse, E.M.; Kew, N.; Fetterplace, K.; Tan, S.Y.; Maier, A.B. The effect of dose, frequency, and timing

Ref #	PMID	Citation
		of
94	35445069	Kwon, Y.J.; Lee, H.S.; Park, G.E.; Kim, H.M.; Lee, J.J.; Kim, W.J.; Lee, J.W. The Association Between Total Protein Intake and All
95	36854962	Haghighatdoost, F.; Mohammadifard, N.; Zakeri, P.; Najafian, J.; Sadeghi, M.; Roohafza, H.; Sarrafzadegan, N. Differences in all-c
96	38363105	Shen, R.; Xu, Z.; Lin, L.; Shen, J.; Hu, W.; Lv, L.; Wei, T. Association between adequate dietary protein and all-cause and cardio
97	41465950	Tao, X.; Yi, Y.; Yang, Y.; Zhao, Z. Association between dietary protein intake and all-cause mortality among individuals with diff
98	38555664	Higgins, J.P.T.; Morgan, R.L.; Rooney, A.A.; Taylor, K.W.; Thayer, K.A.; Silva, R.A.; Lemeris, C.; Akl, E.A.; Bateson, T.F.; Berkm
99	40926900	Chu, J.; Zhang, H.; Wu, Y.; Huang, Y.; Zhu, T.; Zhou, Z.; Wang, H. Efficacy of lifestyle modification combined with GLP-1 receptor
100	42303931	Simsek, H.; Ucar, A. GLP-1 Receptor Agonists for Obesity Management in Older

Ref #	PMID	Citation
		Adults: A Scoping Review on the Risk of Sarcopenia an
101	32628589	Ida, S.; Kaneko, R.; Imataka, K.; Okubo, K.; Shirakura, Y.; Azuma, K.; Fujiwara, R.; Murata, K. Effects of Antidiabetic Drugs on M
102	40352260	Johnson, B.; Milstead, M.; Thomas, O.; McGlasson, T.; Green, L.; Kreider, R.; Jones, R. Investigating nutrient intake during use o
103	42280393	Zambrano-Villacres, R.; Campuzano-Donoso, M.; Reytor-González, C.; Rossetti, G.; Cobellis, L.; Cobellis, F.; Pilone, V.; Simancas-
104	39771028	Nilsson, M.I.; Xhuti, D.; de Maat, N.M.; Hettinga, B.P.; Tarnopolsky, M.A. Obesity and Metabolic Disease Impair the Anabolic Respo
105	42020128	Alawadhi, A.A.; Alroudhan, D.; Alsaeed, D.J.; Almarshad, B.W.; Alshehabi, D.H.; Allahou, B.A.; Mashankar, A.; Al Ozairi, E.; Gray,
106	40704320	Yin, H.; Qiu, Y.; Gan, C.; Zhou, Y.; Chen, T.;

Ref #	PMID	Citation
		Liang, M. The association between a dietary index for the gut microbiota and frailt
107	40383993	Xu, X.; Su, H.; Chen, J.; Liu, D.; Chen, A.; Hu, D.; Cao, J.; Huang, Y. Association of the Dietary Index for Gut Microbiota With S
108	37324750	Zhao, J.; Liang, R.; Song, Q.; Song, S.; Yue, J.; Wu, C. Investigating association between gut microbiota and sarcopenia-related t
109	38995073	Zhang, X.; Yang, G.; Jiang, S.; Ji, B.; Xie, W.; Li, H.; Sun, J.; Li, Y. Causal Relationship Between Gut Microbiota, Metabolites,
110	39192550	Lapauw, L.; Rutten, A.; Dupont, J.; Amini, N.; Vercauteren, L.; Derrien, M.; Raes, J.; Gielen, E. Associations between gut microbi
111	36349647	Almeida, H.M.; Sardeli, A.V.; Conway, J.; Duggal, N.A.; Cavaglieri, C.R. Comparison between frail and non-frail older adults' gut
112	35187864	Nunes, E.A.; Colenso-Semple, L.; McKellar, S.R.; Yau, T.; Ali, M.U.; Fitzpatrick-Lewis, D.; Sherifali, D.;

Ref #	PMID	Citation
		Gaudichon, C.; Tomé, D.
113	42208412	Dharmansyah, D.; Rahayuwati, L.; Pramukti, I.; Mutyara, K. Exercise and nutritional interventions for sarcopenia-related fall prev
114	37209044	Zhou, H.H.; Liao, Y.; Peng, Z.; Liu, F.; Wang, Q.; Yang, W. Association of muscle wasting with mortality risk among adults: A syst
115	28647519	Kelley, G.A.; Kelley, K.S. Is sarcopenia associated with an increased risk of all-cause mortality and functional disability?. *Exp
116	41933584	Du, J.; Shu, X.; Lin, T.; Liao, H.; Zhang, L.; Yue, J. Sarcopenia and Mortality, Cardiovascular and Renal Outcomes in Type 2 Diabe
117	37993037	Nakano, Y.; Mandai, S.; Naito, S.; Fujiki, T.; Mori, Y.; Ando, F.; Mori, T.; Susa, K.; Iimori, S.; Sohara, E.; et al. Effect of os
118	36062861	Seo, D.H.; Suh, Y.J.; Cho, Y.; Ahn, S.H.; Seo, S.; Hong, S.; Lee, Y.H.; Choi, Y.J.; Lee, E.; Kim,

Ref #	PMID	Citation
		S.H. Effect of low skeletal musc
119	41201844	Walrand, S.; Le Bacquer, O. Dietary leucine intake and sarcopenia: from isolated supplementation to combined strategies. *Curr Opi
120	36800504	Ni Lochlainn, M.; Bowyer, R.C.E.; Welch, A.A.; Whelan, K.; Steves, C.J. Higher dietary protein intake is associated with sarcopeni
121	37571311	Wu, Y.; Wang, M.; Long, Z.; Ye, J.; Cao, Y.; Pei, B.; Gao, Y.; Yu, Y.; Han, Z.; Wang, F.; et al. How to Keep the Balance between R
122	35458185	Wu, W.; Tang, N.; Zeng, J.; Jing, J.; Cai, L. Dietary Protein Patterns during Pregnancy Are Associated with Risk of Gestational Di
123	41996864	Barrantes-Espinola, G.; Alberti, P.; Jaramillo-Morales, E.; Rodriguez, A.; López-Moreno, M. Isocaloric substitution of animal prot
124	31682257	Budhathoki, S.; Sawada, N.; Iwasaki, M.; Yamaji, T.; Goto, A.; Kotemori, A.; Ishihara, J.; Takachi, R.; Charvat,

Ref #	PMID	Citation
		H.; Mizoue, T.; e
125	40362749	Karpouzi, C.; Kosmidis, I.; Petridou, A.; Voulgaridou, G.; Papadopoulou, S.K.; Bogdanis, G.C.; Mougios, V. Effects of Protein Supp
126	33198824	Dardevet, D.; Mosoni, L.; Savary-Auzeloux, I.; Peyron, M.A.; Polakof, S.; Rémond, D. Important determinants to take into account t
127	42158256	Xia, H.; Xiang, R.; Song, X.; Qu, Y.; Zhao, X.; Liu, T.; Xia, M.; Zhong, Y.; Chen, Z.; Ju, Y.; et al. Dietary protein intake, infl
128	30709690	Granic, A.; Mendonça, N.; Sayer, A.A.; Hill, T.R.; Davies, K.; Siervo, M.; Mathers, J.C.; Jagger, C. Effects of dietary patterns a
129	28642676	Jäger, R.; Kerksick, C.M.; Campbell, B.I.; Cribb, P.J.; Wells, S.D.; Skwiat, T.M.; Purpura, M.; Ziegenfuss, T.N.; Ferrando, A.A.;
130	41841121	Colosio, A.L.; D’hoe, B.; Bourgois, J.G.; Boone, J. Minimum load threshold in resistance training: insights into muscle metabolism
131	25232749	Vezina, J.W.; Der

Ref #	PMID	Citation
		Ananian, C.A.; Greenberg, E.; Kurka, J. Sociodemographic correlates of meeting US Department of Health and Human
132	41183515	Quinn, T.D.; Boyer, W.R. Occupational Physical Activity and Mortality Risk: Evidence From a Nationally Representative US Sample (N
133	27801685	Ko, G.J.; Obi, Y.; Tortorici, A.R.; Kalantar-Zadeh, K. Dietary protein intake and chronic kidney disease. *Curr Opin Clin Nutr Met
134	41580028	Duarte, M.P.; Martins, P.; Leal, D.V.; Nóbrega, O.T.; Ribeiro, H.S.; Viana, J.L. Diabetes and sarcopenia in chronic kidney disease
135	33917987	IJmker-Hemink, V.; Moolhuijzen, N.; Wanten, G.; van den Berg, M. High Frequency Protein-Rich Meal Service to Promote Protein Distr
136	31794597	Hudson, J.L.; Wang, Y.; Bergia Iii, R.E.; Campbell, W.W. Protein Intake Greater than the RDA Differentially Influences Whole-Body

Ref #	PMID	Citation
137	32719809	Ravelli, M.N.; Schoeller, D.A. Traditional Self-Reported Dietary Instruments Are Prone to Inaccuracies and New Approaches Are Need
138	36639712	Thieleking, R.; Schneidewind, L.; Kanyamibwa, A.; Hartmann, H.; Horstmann, A.; Witte, A.V.; Medawar, E. Nutrient scoring for the D
139	38060823	Shonkoff, E.; Cara, K.C.; Pei, X.A.; Chung, M.; Kamath, S.; Panetta, K.; Hennessy, E. AI-based digital image dietary assessment me
140	39608003	Zheng, J.; Wang, J.; Shen, J.; An, R. Artificial Intelligence Applications to Measure Food and Nutrient Intakes: Scoping Review. *
141	38623738	Lee, L.; Hall, R.; Stanley, J.; Krebs, J. Tailored Prompting to Improve Adherence to Image-Based Dietary Assessment: Mixed Methods
142	40218886	Tanabe, H.; Yanai, K. Reasoning-Driven Food Energy Estimation via Multimodal Large

Ref #	PMID	Citation
		Language Models. *Nutrients* **2025**, *17*, 11
143	34144689	Martins, P.; Marques, E.A.; Leal, D.V.; Ferreira, A.; Wilund, K.R.; Viana, J.L. Association between physical activity and mortalit
144	39611889	Zhixiang, Y.; Yongxing, X.; Juan, L.; Qing, Y.; Yangyang, L.; Zhangrui, Z.; Yuehua, G. The effects of soy protein and soy isoflavo
145	35057453	Zheng, J.; Zhu, T.; Yang, G.; Zhao, L.; Li, F.; Park, Y.M.; Tabung, F.K.; Steck, S.E.; Li, X.; Wang, H. The Isocaloric Substitutio
146	34444699	Capurso, C. Whole-Grain Intake in the Mediterranean Diet and a Low Protein to Carbohydrates Ratio Can Help to Reduce Mortality fro

Table S7. ROBINS-E — observational exposure studies entering a synthesis

Domains: **D1** confounding · **D2** measurement of the exposure · **D3** selection of participants · **D4** post-exposure interventions · **D5** missing data · **D6** measurement of the outcome · **D7** selection of the reported result. Ratings: Low / Some concerns / High.

Coverage. Twenty-six studies were appraised in duplicate. One further cohort entering a synthesis — the Women's Health Initiative (PMID 33624505), admitted to the plant-protein stratum by the top-up search at revision — was identified after the appraisal was complete and is not rated here. It is carried in the pooled estimate,

and the risk-of-bias sensitivity analysis is reported under both possible ratings for it, with the same conclusion either way (Section 3.5, Document S1 item 8).

PMID	Study	D1	D2	D3	D4	D5	D6	D7	Overall	What the rating turns on
41360828	Kailuan, Chinese adults, n=109,407	Some concerns	Some concerns	Low	Low	Some concerns	Low	Low	**Some concerns**	D1/D5: median follow-up 6.99 y is short for a mortality endpoint, so reverse causation is not excluded; D2: self-reported activity survey.
37316812	KNHANES 2007–2013,	Some concerns	Some concerns	Low	Low	Low	Low	Low	**Some concerns**	D2: self-reported aero

PMID	Study	D1	D2	D3	D4	D5	D6	D7	Overall	What the rating turns on
	n=34,379									bic, muscle-strengthening and flexibility activity.
39283728	Israel ISGIOH, 20-y follow-up, n=1,210	Some concerns	Some concerns	Some concerns	Low	Some concerns	Low	Low	**Some concerns**	D2: self-reported exercise ‘properties’; D3: single-center convenience cohort; D1: baseline health status likely

PMID	Study	D1	D2	D3	D4	D5	D6	D7	Overall	What the rating turns on
										determines exercise habit in a cohort with mean age 73.
40690774	NHIS diabetes, n=51,650	Some concerns	Some concerns	Low	Low	Low	Low	Low	**Some concerns**	D2: self-reported MVPA and self-reported diabetes status.
40269864	UK Biobank stroke survivors, n=5,507	High	Some concerns	Some concerns	Low	Some concerns	Low	Some concerns	**High**	D1: severe confounding by post-stroke disab

PMID	Study	D1	D2	D3	D4	D5	D6	D7	Overall	What the rating turns on
										ility, which determines both activity and mortality and is not fully measurable; D3: UK Biobank healthy-volunteer selection; D7: estimate taken from a joint PA×sleep

PMID	Study	D1	D2	D3	D4	D5	D6	D7	Overall	What the rating turns on
										analysis.
41165888	NHANES diabetes/prediabetes, n=7,337	Some concerns	Some concerns	Low	Low	Low	Low	High	**High**	D7: the review's target estimate is a secondary component of a mediation analysis whose primary exposure is biological-age acceleration, not physical

PMID	Study	D1	D2	D3	D4	D5	D6	D7	Overall	What the rating turns on
										activity.
42296133	NHANES coronary heart disease, n=4,823	Some concerns	Some concerns	Low	Low	Low	Low	High	**High**	D7: the activity estimate is one component of a Comprehensive Health Assessment Score and is not the paper's primary contrast.
40638069	Lifelines cancer coho	Some concerns	Some concerns	Low	Some concerns	Some concerns	Low	Low	**Some concerns**	D4: pre-diagnosis expo

PMID	Study	D1	D2	D3	D4	D5	D6	D7	Overall	What the rating turns on
	rt, n=152,915 screened									sure with post-diagnosis treatment unmeasured; D2: self-reported guideline adherence.
41579289	German long-term cancer survivors, n=6,057	Some concerns	Some concerns	Some concerns	Some concerns	Some concerns	Low	Some concerns	**Some concerns**	D7: activity is one of four components of a healthy-lifestyle score; D3: survivors

PMID	Study	D1	D2	D3	D4	D5	D6	D7	Overall	What the rating turns on
										of ≥5 years only, a survival-selected sample.
42230125	HPFS + NHS + NHS II, n=147,374, 30 y	Low	Some concerns	Low	Low	Low	Low	Low	**Some concerns**	D2: questionnaire-based, but validated and repeated biennially over three decades—the strongest exposure measurement in

PMID	Study	D1	D2	D3	D4	D5	D6	D7	Overall	What the rating turns on the set.
37558986	Kawasaki Aging & Wellbeing, n=833, age 85–89	Some concerns	High	Some concerns	Low	Some concerns	Low	Some concerns	**High**	D2: brief self-administered diet history questionnaire in adults aged 85+; D1: at this age, appetite and protein intake are markers of health status rathe

PMID	Study	D1	D2	D3	D4	D5	D6	D7	Overall	What the rating turns on
										r than causes of it; D3: participants with MMSE <24 excluded.
38363105	NHANES 1999–2004, glomerular hypofiltration, n=2,168	Some concerns	High	Low	Low	Some concerns	Low	Some concerns	**High**	D2: dietary protein from 24-hour recall at a single baseline, carried forward over a median 204

PMID	Study	D1	D2	D3	D4	D5	D6	D7	Overall	What the rating turns on
										months of follow-up.
41465950	NHANES 1999–2018, n=48,930, by eGFR stratum	Some concerns	High	Low	Low	Some concerns	Low	Some concerns	**High**	D2: as above; D7: twelve intake × eGFR cells reported, of which the review uses one.
36854962	Isfahan Cohort Study, n=5,431, 11.3 y	Some concerns	High	Low	Low	Some concerns	Low	Some concerns	**High**	D2: protein intake estimated from *frequency* of cons

PMID	Study	D1	D2	D3	D4	D5	D6	D7	Overall	What the rating turns on
										umption, not quantified intake; 483 deaths only.
32076944	Rotterdam Study, n=7,786, 13 y, 3,589 deaths	Some concerns	Some concerns	Low	Low	Low	Low	Low	**Some concerns**	D2: FFQ at three time points; otherwise the best-characterized protein cohort in the set.
35445069	KoGES CKD stage 3–5, n=3,8	High	Some concerns	Low	Low	Some concerns	Low	Low	**High**	D1: confounding by indic

PMID	Study	D1	D2	D3	D4	D5	D6	D7	Overall	What the rating turns on
	92									ation—protein restriction in CKD is prescribed to those with worse disease, so the exposure is partly an index of severity.
41260173	UK Biobank ≥1.8 g/kg/day, n=19,420,	Some concerns	Some concerns	Some concerns	Low	Some concerns	Low	Some concerns	**Some concerns**	Rating recalculated at revision. It had

PMID	Study	D1	D2	D3	D4	D5	D6	D7	Overall	What the rating turns on
	PSM									been recorded as High on the strength of indirectness — that MACE is the primary outcome and all-cause mortality a secondary one (HR 1.39, 1.17–1.65, the estimate

PMID	Study	D1	D2	D3	D4	D5	D6	D7	Overall	What the rating turns on
										used here), and that the comparator averages ~1.27 g/kg/day, so the contrast is ‘very high versus adequate’ rather than ‘highest versus lowest’. ROBINS-E has no indire

PMID	Study	D1	D2	D3	D4	D5	D6	D7	Overall	What the rating turns on
										ctness domain, and no domain here is rated High, so the overall rating follows the domains. The indirectness is real and is now carried where the instrume

PMID	Study	D1	D2	D3	D4	D5	D6	D7	Overall	What the rating turns on
										nt for it exists, in the certainty rating (Table S10) and at each point of use. D3: UK Biobank dietary sub-sample, 72.8% women.
39543271	NHANES type 2 diabetes,	Some concerns	High	Low	Low	Some concerns	Low	Low	**High**	D2: 24-hour recall; D1: diabe

PMID	Study	D1	D2	D3	D4	D5	D6	D7	Overall	What the rating turns on
	n=4,646									tes duration and treatment intensity incompletely captured.
42541632	SUN cohort, n=17,989, repeated FFQ	Some concerns	Some concerns	Some concerns	Low	Some concerns	Low	Low	**Some concerns**	D3: Spanish university graduates — a healthy, highly educated cohort with limited transportability.
28715883	Korean	Some	Some	Low	Low	Low	Low	Low	**Some**	D2: self-

PMID	Study	D1	D2	D3	D4	D5	D6	D7	Overall	What the rating turns on
	adults, 8-y prospective (KLoSA)	concerns	concerns						**concerns**	reported activity; otherwise a straightforward whole-cohort guideline-adherence contrast.
39968650	Chinese adults, 47,281 deaths, 12.1 y	Some concerns	Some concerns	Low	Low	Low	Low	Low	**Some concerns**	D2: questionnaire-based activity. The largest and most precisely estim

PMID	Study	D1	D2	D3	D4	D5	D6	D7	Overall	What the rating turns on
										ated cohort in the stratum; it contributes most of the weight, and omitting it lowers s I^2 from 64% to 39% (Section 3.2, Table S14).
39077609	Older adults with heart failure, medi	High	Some concerns	Some concerns	Low	Some concerns	Low	Low	**High**	D1: in heart failure, functional class

PMID	Study	D1	D2	D3	D4	D5	D6	D7	Overall	What the rating turns on
	an 3.2 y									determines both activity and mortality and is only partly captured by covariates; D3: registry-based selection; median follow-up 3.2 y is short for a mortality endp

PMID	Study	D1	D2	D3	D4	D5	D6	D7	Overall	What the rating turns on
										oint in this population, so reverse causation is not excluded.
36902707	NHANES, non-alcoholic fatty liver disease	Some concerns	High	Low	Low	Some concerns	Low	Some concerns	**High**	D2: activity from a single self-report at baseline; D7: leisure-time, transportation and total PA each repor

PMID	Study	D1	D2	D3	D4	D5	D6	D7	Overall	What the rating turns on
										ted against several models, of which the review uses one.
40308924	NHANES, adults with depression	High	Some concerns	Low	Low	Some concerns	Low	Low	**High**	D1: depression severity determines both activity and mortality and is measured only by screening

PMID	Study	D1	D2	D3	D4	D5	D6	D7	Overall	What the rating turns on
										instrument, so confounding by indication is expected to be substantial.
27730174	After COPD hospitalization, 12-month follow-up	High	Some concerns	Low	Some concerns	Some concerns	Low	Low	**High**	D1: activity recorded in routine care before an index admission is a proxy for disease severity;

PMID	Study	D1	D2	D3	D4	D5	D6	D7	Overall	What the rating turns on
										D4: post-discharge treatment unmeasured; the 12-month horizon after hospitalization is exactly where reverse causation is strongest. This is the largest effect in

PMID	Study	D1	D2	D3	D4	D5	D6	D7	Overall	What the rating turns on
										the stratum and the least trustworthy.
33083530	Breast cancer survivors, Multiethnic Cohort	Some concerns	Some concerns	Some concerns	Some concerns	Some concerns	Low	Some concerns	**Some concerns**	D4: post-diagnosis treatment unmeasured; D7: results reported by ethnicity and stage, of which the all-women estimate is used;

PMID	Study	D1	D2	D3	D4	D5	D6	D7	Overall	What the rating turns on
										the ‘active’ cut-point of 6.9 MET-h/week sits below the guideline threshold and is a dichotomy, not a guideline contrast.

Summary. 26 studies appraised; 13 rated High overall, 13 Some concerns, 0 Low. One rating was recalculated at revision (41260173), because it had been assigned on indirectness, which ROBINS-E does not assess. No study was removed on the basis of its rating; the ratings feed the certainty grading in Table S10.

Table S7b. Newcastle–Ottawa Scale (cohort form) — the same 26 observational studies

Coverage. Twenty-six studies were scored by a single rater at revision (Section 2.9); the ROBINS-E appraisal of the same studies was made in duplicate. One further

cohort entering a synthesis — the Women's Health Initiative (PMID 33624505), admitted to the plant-protein stratum by the top-up search at revision — was identified after the appraisal was complete and is not rated here. It is carried in the pooled estimate, and the risk-of-bias sensitivity analysis is reported under both possible ratings for it, with the same conclusion either way (Section 3.5, Document S1 item 8).

Scored at revision as a cross-check on the ROBINS-E grid in Table S7. Nine possible stars: **Selection** S1 representativeness of the exposed cohort · S2 selection of the non-exposed cohort · S3 ascertainment of exposure · S4 outcome not present at start; **Comparability** C1 age · C2 any additional factor; **Outcome** O1 assessment of outcome · O2 follow-up long enough · O3 adequacy of follow-up. Bands: Good ≥ 7, Fair 5–6, Poor ≤ 4.

PMID	Study	S1	S2	S3	S4	C1	C2	O1	O2	O3	Sel	Com	Out	Total	Band	ROBINS-E	What the score turns on
41360828	Kailuan, Chinese adults, n=109,407	—	★	—	★	★	★	★	★	★	2/4	2/2	3/3	**7/9**	Good	Some concerns	S1: occupational cohort of coal-industry

PMID	Study	S1	S2	S3	S4	C1	C2	O1	O2	O3	Sel	Com	Out	Total	Band	ROBINS-E	What the score turns on
																	employees, not a community sample. S3: self-reported baseline surv

PMID	Study	S1	S2	S3	S4	C1	C2	O1	O2	O3	Sel	Com	Out	Total	Band	ROBINS-E	What the score turns on
																	ey. O1: death certificates plus provincial vital registration. O2: med

PMID	Study	S1	S2	S3	S4	C1	C2	O1	O2	O3	Sel	Com	Out	Total	Band	ROBINS-E	What the score turns on
																	ian 6.99y.
37316812	KNHANES 2007–2013, n=34,379	★	★	—	★	★	★	★	★	★	3/4	2/2	3/3	**8/9**	Good	Some concerns	S1: nationally representative survey. S3: activity self-repo

PMID	Study	S1	S2	S3	S4	C1	C2	O1	O2	O3	Sel	Com	Out	Total	Band	ROBINS-E	What the score turns on
																	rted at baseline. O1: death certificates matched on resident registration

PMID	Study	S1	S2	S3	S4	C1	C2	O1	O2	O3	Sel	Com	Out	Total	Band	ROBINS-E	
																	What the score returns on number. O2: up to 12.4 y.
39283728	Israel ISGIOH, 20-y follow-up, n=1,210 [a]	—	★	—	★	—	—	—	★	—	2/4	0/2	1/3	**3/9**	Poor	Some concerns	Abstract only. Nothing beyond design, n and

PMID	Study	S1	S2	S3	S4	C1	C2	O1	O2	O3	Sel	Com	Out	Total	Band	ROBINS-E	What the score turns on
																	20-y horizon is verifiable; six items score no star for want of a report,

PMID	Study	S1	S2	S3	S4	C1	C2	O1	O2	O3	Sel	Com	Out	Total	Band	ROBINS-E	What the score turns on
																	not for want of quality.
40690774	NHIS diabetes, n=51,650[a]	★	★	—	★	—	—	★	★	★	3/4	0/2	3/3	**6/9**	Fair	Some concerns	Abstract only. S1: National Health Intervie

PMID	Study	S1	S2	S3	S4	C1	C2	O1	O2	O3	Sel	Com	Out	Total	Band	ROBINS-E	What the score turns on
																	w Survey. O1: National Death Index linkage. O2: median 9.5 y. C

PMID	Study	S1	S2	S3	S4	C1	C2	O1	O2	O3	Sel	Com	Out	Total	Band	ROBINS-E	What the score turns on
																	1/C2 not verifiable from the abstract.
40269864	UK Biobank stroke survivors,	—	★	—	★	★	★	★	★	★	2/4	2/2	3/3	**7/9**	Good	High	S1: UK Biobank is a 5.5%

PMID	Study	S1	S2	S3	S4	C1	C2	O1	O2	O3	Sel	Com	Out	Total	Band	ROBINS-E	What the score turns on
	n=5,507																-response volunteer cohort, not representative. S3: touch-screen se

PMID	Study	S1	S2	S3	S4	C1	C2	O1	O2	O3	Sel	Com	Out	Total	Band	ROBINS-E	What the score turns on
																	lf-completion. O2: mean 12.55 y.
41165888	NHANES diabetes/prediabetes	★	★	★	★	★	★	★	★	★	4/4	2/2	3/3	**9/9**	Good	High	S3: NHANES exposure instrum

PMID	Study	S1	S2	S3	S4	C1	C2	O1	O2	O3	Sel	Com	Out	Total	Band	ROBINS-E	What the score turns on
	, n=7,337																ents are interviewer-administered. O1: National Death Index. O

PMID	Study	S1	S2	S3	S4	C1	C2	O1	O2	O3	Sel	Com	Out	Total	Band	ROBINS-E	What the score returns on 2: median 94 months. Full marks on NOS while High on ROBINS-E

PMID	Study	S1	S2	S3	S4	C1	C2	O1	O2	O3	Sel	Com	Out	Total	Band	ROBINS-E	What the score returns on
																	— see the disagreement note.
42296133	NHANES coronary heart disease, n=	★	★	★	★	★	★	★	★	★	4/4	2/2	3/3	**9/9**	Good	High	As above. O2: median 6.2 y. The RO

PMID	Study	S1	S2	S3	S4	C1	C2	O1	O2	O3	Sel	Com	Out	Total	Band	ROBINS-E	What the score turns on
	4,823																BINS-E High turns on D7, which NOS has no item for.
40638069	Lifelines canc	★	★	—	★	★	★	★	—	★	3/4	2/2	2/3	**7/9**	Good	Some concer	S3: self-admi

PMID	Study	S1	S2	S3	S4	C1	C2	O1	O2	O3	Sel	Com	Out	Total	Band	ROBINS-E	What the score turns on
	er cohort, n=152,915 screened															ns	nistered baseline questionnaire. O1: Netherlands Cancer Registry lin

PMID	Study	S1	S2	S3	S4	C1	C2	O1	O2	O3	Sel	Com	Out	Total	Band	ROBINS-E	What the score turns on
																	kage. O2: no follow-up duration is given for the reported contrast.
41	G	—	★	—	★	★	★	★	★	—	2/	2/	2/	**6/**	Fa	So	S1

PMID	Study	S1	S2	S3	S4	C1	C2	O1	O2	O3	Sel	Com	Out	Total	Band	ROBINS-E	What the score turns on
57929289	erman long-term cancer survivors, n=6,057										4	2	3	**9**	ir	me concerns	: 42% of those contacted responded, and responders were younger

PMID	Study	S1	S2	S3	S4	C1	C2	O1	O2	O3	Sel	Com	Out	Total	Band	ROBINS-E	What the score turns on
																	and further from diagnosis—the authors say so. S3: postal qu

PMID	Study	S1	S2	S3	S4	C1	C2	O1	O2	O3	Sel	Com	Out	Total	Band	ROBINS-E	What the score turns on
																	estionnaire. O3: losses not described.
42230125	HPFS + NHS + NHS II, n=14	—	★	—	★	★	★	—	★	—	2/4	2/2	1/3	**5/9**	Fair	Some concerns	Abstract only. S1: health prof

PMID	Study	S1	S2	S3	S4	C1	C2	O1	O2	O3	Sel	Com	Out	Total	Band	ROBINS-E	What the score turns on
	7,374, 30y [a]																essionals, an occupational cohort. S3: validated self-administered ques

PMID	Study	S1	S2	S3	S4	C1	C2	O1	O2	O3	Sel	Com	Out	Total	Band	ROBINS-E	What the score turns on
																	tionnaires, biennial. C2: adjusted for aerobic activity, stated in the

PMID	Study	S1	S2	S3	S4	C1	C2	O1	O2	O3	Sel	Com	Out	Total	Band	ROBINS-E	What the score turns on abstract.
37558986	Kawasaki Aging & Wellbeing, n=833, age 85–89	★	★	—	★	★	★	—	—	—	3/4	2/2	0/3	**5/9**	Fair	High	S3: self-administered brief diet history questionnaire. O1: si

PMID	Study	S1	S2	S3	S4	C1	C2	O1	O2	O3	Sel	Com	Out	Total	Band	ROBINS-E	What the score turns on
																	x-monthly telephone survey, not registry linkage. O2: mean 1,218

PMID	Study	S1	S2	S3	S4	C1	C2	O1	O2	O3	Sel	Com	Out	Total	Band	ROBINS-E	What the score turns on
																	days. O3: losses not described.
38363105	NHANES 1999–2004, glomerul	★	★	★	★	—	★	★	★	★	4/4	1/2	3/3	**8/9**	Good	High	Abstract only, but the NHANES

PMID	Study	S1	S2	S3	S4	C1	C2	O1	O2	O3	Sel	Com	Out	Total	Band	ROBINS-E	What the score turns on
	ar hypofiltration, n=2,168 [a]																design fixes S1, S3, O1 and O3. O2: median 204 months. C1 not

PMID	Study	S1	S2	S3	S4	C1	C2	O1	O2	O3	Sel	Com	Out	Total	Band	ROBINS-E	What the score turns on
																	separately verifiable.
41465950	NHANES 1999–2018, n=48,930, by eGFR strat	★	★	★	★	★	★	★	★	★	4/4	2/2	3/3	**9/9**	Good	High	O2: 489,605 person-years over 48,930 participa

PMID	Study	S1	S2	S3	S4	C1	C2	O1	O2	O3	Sel	Com	Out	Total	Band	ROBINS-E	What the score turns on
	um																nts, about 10 y. Full NOS marks against a High ROBINS-E rating.
36	Isf	★	★	—	★	★	★	—	★	—	3/	2/	1/	**6/**	Fa	Hi	S1

PMID	Study	S1	S2	S3	S4	C1	C2	O1	O2	O3	Sel	Com	Out	Total	Band	ROBINS-E	What the score turns on
8549 62	ahan Cohort Study, n=5,431, 11.3 y										4	2	3	**9**	ir	gh	: population-based cohort in Isfahan. S3: validated but self-administ

PMID	Study	S1	S2	S3	S4	C1	C2	O1	O2	O3	Sel	Com	Out	Total	Band	ROBINS-E	What the score turns on
																	ered FFQ. O1: ‘any new case of death was recorded’ — the ascertain

PMID	Study	S1	S2	S3	S4	C1	C2	O1	O2	O3	Sel	Com	Out	Total	Band	ROBINS-E	What the score turns on
																	ment route is not specified. O3: not described.
32076944	Rotterdam Stud	★	★	—	★	★	★	★	★	★	3/4	2/2	3/3	**8/9**	Good	Some concerns	S3: self-administ

PMID	Study	S1	S2	S3	S4	C1	C2	O1	O2	O3	Sel	Com	Out	Total	Band	ROBINS-E	What the score turns on
	y, n=7,786, 13y, 3,589 deaths																ered FFQ. O1: municipal records plus GP and hospital follow-up, ne

PMID	Study	S1	S2	S3	S4	C1	C2	O1	O2	O3	Sel	Com	Out	Total	Band	ROBINS-E	What the score turns on
																	ar-complete. O2: median 13 y.
35445069	KoGES CKD stage 3–5, n=3,892	★	★	—	★	★	★	★	★	★	3/4	2/2	3/3	**8/9**	Good	High	S3: self-administered FFQ. O1: Nati

PMID	Study	S1	S2	S3	S4	C1	C2	O1	O2	O3	Sel	Com	Out	Total	Band	ROBINS-E	What the score returns on
																	onal Death Index, Korean Standard Classification of Diseases. O2: med

PMID	Study	S1	S2	S3	S4	C1	C2	O1	O2	O3	Sel	Com	Out	Total	Band	ROBINS-E	What the score returns on
																	ian 11.1y.
41260173	UK Biobank ≥1.8g/kg/day, n=19,420, PSM	—	★	—	★	★	★	★	★	★	2/4	2/2	3/3	**7/9**	Good	Some concerns	S1: UK Biobank volunteer cohort. S3: self-completed di

PMID	Study	S1	S2	S3	S4	C1	C2	O1	O2	O3	Sel	Com	Out	Total	Band	ROBINS-E	What the score turns on
																	etary questionnaire. C2: propensity-score matched in addition to cova

PMID	Study	S1	S2	S3	S4	C1	C2	O1	O2	O3	Sel	Com	Out	Total	Band	ROBINS-E	What the score turns on
																	riate adjustment. O2: about 13 y.
39543271	NHANES type 2 diabetes, n=4,64	★	★	★	★	—	—	★	★	★	4/4	0/2	3/3	**7/9**	Good	High	Abstract only. NHANES design fix

PMID	Study	S1	S2	S3	S4	C1	C2	O1	O2	O3	Sel	Com	Out	Total	Band	ROBINS-E	What the score turns on
	6 [a]																es S1, S3, O1, O3. O2: mean 9 y. The adjustment set is not itemi

PMID	Study	S1	S2	S3	S4	C1	C2	O1	O2	O3	Sel	Com	Out	Total	Band	ROBINS-E	What the score turns on
																	zed in the abstract.
42541632	SUN cohort, n=17,989, repeated FFQ[a]	—	★	—	★	—	★	—	★	—	2/4	1/2	1/3	**4/9**	Poor	Some concerns	Abstract only. S1: Spanish university graduate

PMID	Study	S1	S2	S3	S4	C1	C2	O1	O2	O3	Sel	Com	Out	Total	Band	ROBINS-E	What the score turns on
																	s, a selected cohort by design. S3: self-administered validated FFQ. O

PMID	Study	S1	S2	S3	S4	C1	C2	O1	O2	O3	Sel	Com	Out	Total	Band	ROBINS-E	What the score turns on
28	Ko	★	★	★	★	★	★	★	★	★	4/	2/	3/	**9/**	G	So	S3
																	1/O3: ascertainment and retention not described in the abstract.

PMID	Study	S1	S2	S3	S4	C1	C2	O1	O2	O3	Sel	Com	Out	Total	Band	ROBINS-E	What the score turns on
7158583	re an adults, 8-y prospective (KLoSA)										4	2	3	**9**	ood	me concerns	: computer-assisted personal interviewing—the only interviewer

PMID	Study	S1	S2	S3	S4	C1	C2	O1	O2	O3	Sel	Com	Out	Total	Band	ROBINS-E	What the score turns on
																	-administered activity measure outside NHANES in this set. O1: deat

PMID	Study	S1	S2	S3	S4	C1	C2	O1	O2	O3	Sel	Com	Out	Total	Band	ROBINS-E	What the score turns on
																	h certificates and family interview. O3: 10.5% excluded and described

PMID	Study	S1	S2	S3	S4	C1	C2	O1	O2	O3	Sel	Com	Out	Total	Band	ROBINS-E	What the score turns on.
39968650	China Kadoorie Biobank, 47,281 deaths, 12.1 y	★	★	—	★	★	★	★	★	★	3/4	2/2	3/3	**8/9**	Good	Some concerns	S1: 10 geographically diverse areas. S3: self-reported MET-h/da

PMID	Study	S1	S2	S3	S4	C1	C2	O1	O2	O3	Sel	Com	Out	Total	Band	ROBINS-E	What the score turns on
																	y. O1: local and national death registries. O3: one participant lost

PMID	Study	S1	S2	S3	S4	C1	C2	O1	O2	O3	Sel	Com	Out	Total	Band	ROBINS-E	What the score turns on
																	to follow-up, stated.
39077609	Older adults with heart failure, median 3.2 y	—	★	—	★	★	★	—	—	—	2/4	2/2	0/3	**4/9**	Poor	High	S1: clinic-based heart-failure sample, source

PMID	Study	S1	S2	S3	S4	C1	C2	O1	O2	O3	Sel	Com	Out	Total	Band	ROBINS-E	What the score turns on
																	population not defined. S3: 7-day recall self-report questionnaire. O

PMID	Study	S1	S2	S3	S4	C1	C2	O1	O2	O3	Sel	Com	Out	Total	Band	ROBINS-E	What the score turns on
																	2: median 3.2 y. O1/O3: ascertainment and retention not describ

PMID	Study	S1	S2	S3	S4	C1	C2	O1	O2	O3	Sel	Com	Out	Total	Band	ROBINS-E	What the score turns on.
36902707	NHANES, non-alcoholic fatty liver disease	★	★	★	★	★	★	★	★	★	4/4	2/2	3/3	**9/9**	Good	High	O1: National Death Index with the enhanced linkage algorithm. O

PMID	Study	S1	S2	S3	S4	C1	C2	O1	O2	O3	Sel	Com	Out	Total	Band	ROBINS-E	What the score turns on
																	2: median 8.6 y.
40308924	NHANES, adults with depression	★	★	★	★	★	★	★	★	★	4/4	2/2	3/3	**9/9**	Good	High	O1: National Death Index linked on SEQN. O2: 13 y

PMID	Study	S1	S2	S3	S4	C1	C2	O1	O2	O3	Sel	Com	Out	Total	Band	ROBINS-E	What the score turns on
																	of follow-up.
27730174	After COPD hospitalization, 12-month follow-up	★	★	★	★	★	★	★	—	★	4/4	2/2	2/3	**8/9**	Good	High	S3: activity routinely recorded in clinical care — a secure

PMID	Study	S1	S2	S3	S4	C1	C2	O1	O2	O3	Sel	Com	Out	Total	Band	ROBINS-E	What the score turns on
																	record, not a research questionnaire. O1: state and national death in

PMID	Study	S1	S2	S3	S4	C1	C2	O1	O2	O3	Sel	Com	Out	Total	Band	ROBINS-E	What the score turns on
																	dices. O2: 12 months only, the shortest horizon in the set and there re

PMID	Study	S1	S2	S3	S4	C1	C2	O1	O2	O3	Sel	Com	Out	Total	Band	ROBINS-E	What the score turns on
																	as on this estimate is the least trustworthy in Table 1.
33083530	Breast canc	★	★	—	★	★	★	★	★	★	3/4	2/2	3/3	**8/9**	Good	Some conc	S1: populat

PMID	Study	S1	S2	S3	S4	C1	C2	O1	O2	O3	Sel	Com	Out	Total	Band	ROBINS-E	What the score turns on
	er survivors, Multiethnic Cohort															erns	ion-based Multiethnic Cohort. S3: self-reported activity in the past m

PMID	Study	S1	S2	S3	S4	C1	C2	O1	O2	O3	Sel	Com	Out	Total	Band	ROBINS-E	What the score turns on
																	onth. O2: median 13 y.

[a] No open-access full text; scored from the abstract and PubMed record alone. Items that the report does not permit to be judged receive no star, so these totals are reporting-limited and should not be read as evidence of poorer conduct.

Scoring conventions, fixed in advance and applied uniformly. S3 is awarded only for a secure record or an interviewer-administered instrument; a self-administered postal, touch-screen or take-home questionnaire, including a self-administered food-frequency questionnaire, receives no star. S4 is awarded to every study, because the outcome is mortality and no participant has died at baseline — it is recorded rather than silently awarded so the reader knows this item does no discriminating work here. O2 requires a median or mean follow-up of at least five years for a mortality endpoint. O3 is awarded for vital-status ascertainment by national or regional registry linkage, or for a described loss to follow-up below 20%.

What the disagreement with ROBINS-E means, in full.

The Newcastle–Ottawa cross-check disagrees with ROBINS-E, and the disagreement is the useful part. Scored on the same 26 studies, NOS returns a median of 7.5 stars out of 9 (IQR 6.0–8.2), with 18 studies in the Good band, five Fair and three Poor (the grid above). Read alone, that is a reassuring picture of a sound literature. It is not the picture ROBINS-E gives, and the two do not merely fail to

agree — in this corpus they point mildly in *opposite* directions. Studies rated High risk of bias on ROBINS-E score a **mean of 7.54 stars** against **6.62** for those rated Some concerns, and **10 of the 13 High-risk studies fall in the NOS Good band**; the rank correlation between the NOS total and ROBINS-E severity is **ρ = +0.30**, where agreement would require it to be negative.

The reason is structural rather than accidental, and it is worth stating because it generalizes beyond this review. The ROBINS-E High ratings in this corpus are driven by two things: selective reporting (D7), where the estimate we use is a by-product of an analysis designed for something else, and confounding by indication or reverse causation (D1), where the exposure is partly an index of how ill the participant already was. NOS has **no item for either**. What it does reward — a nationally representative sampling frame, an interviewer-administered instrument, death ascertainment by registry linkage, near-complete follow-up — is precisely the profile of the NHANES-derived analyses that account for most of our High ratings. NHANES collects nine stars almost by construction, and the failures that concern us are invisible to the instrument that awards them.

Two consequences follow. The NOS totals are reported for transparency and comparability with the existing literature, and they carry **no weight in any certainty grade or conclusion** in this review. And a reader encountering a protein or activity meta-analysis whose quality appraisal rests on Newcastle–Ottawa alone should treat a high star count as evidence about sampling and follow-up, not about confounding — which, in this literature, is where the bias lives. One further caveat belongs with the table: only **9 of the 26** studies earn the exposure star, because a self-administered food-frequency or activity questionnaire does not qualify as a secure record, and six studies without open-access full text were scored from the abstract, so their totals are limited by reporting rather than by conduct.

Distribution. Fair 5 · Good 18 · Poor 3 · median total 7.5/9 (IQR 6.0–8.2).

Agreement with ROBINS-E.

ROBINS-E overall	NOS Good	NOS Fair	NOS Poor	n
Some concerns	8	3	2	13
High	10	2	1	13

Table S8. RoB 2 — randomized trials

Domains: **D1** randomization process · **D2** deviations from intended interventions · **D3** missing outcome data · **D4** measurement of the outcome · **D5** selection of the reported result. Domain 2 is assessed against the *effect of assignment to intervention*; absence of participant blinding is not by itself treated as high risk where the outcome is objectively measured.

PMID	Trial	D1	D2	D3	D4	D5	Overall	What the rating turns on
33028588	Generation 100 (5-year exercise training, n=1,567)	Low	Some concerns	Low	Low	Low	**Some concerns**	Deviations: 'control' participants were free to exercise and many did, and adherence to the assigned modality was partial; the trial therefore estimates the effect of assignment under substantial contamination. Outco

PMID	Trial	D1	D2	D3	D4	D5	Overall	What the rating turns on
								me (death via national registry) and randomization are sound.
34967847	Supplemented very-low-protein vs low-protein diet in advanced CKD (median 74 months)	Low	High	Some concerns	Low	Low	**High**	Deviations: adherence to a very-low-protein diet over six years is difficult to sustain and was incomplete; missing data over that horizon are substantial.

Table S9. AMSTAR 2 — included systematic reviews and meta-analyses

Seven critical domains; ✓ = adequately addressed, ✗ = not. Overall confidence follows AMSTAR 2 rules (one critical flaw → Low; more than one → Critically low).

PMID	Review	C2 protocol	C4 search	C7 exclusions	C9 RoB in studies	C11 method	C13 RoB in interpretation	C15 publication bias	Confidence	Note
34741536	Cochrane — exercise CR for coronary heart disease (2021)	✓	✓	✓	✓	✓	✓	✓	**High**	
38451843	Cochrane — exercise CR for heart failure (2024)	✓	✓	✓	✓	✓	✓	✓	**High**	
33118160	Cochrane — low-	✓	✓	✓	✓	✓	✓	✓	**High**	

PMID	Review	C2 protocol	C4 search	C7 exclusions	C9 RoB in studies	C11 method	C13 RoB in interpretation	C15 publication bias	Confidence	Note
	protein diets in non-diabetic CKD (2020)									
36594428	Cochrane — protein restriction for diabetic kidney disease (2023)	✓	✓	✓	✓	✓	✓	✓	**High**	
30255969	ExTraMATCH II — IPD meta-analysis in heart failur	✓	✓	✗	✓	✓	✓	✗	**Critically low**	C7: exclusions not listed individually; C15: publicatio

PMID	Review	C2 protocol	C4 search	C7 exclusions	C9 RoB in studies	C11 method	C13 RoB in interpretation	C15 publication bias	Confidence	Note
	e (2018)									n bias not formally assessed in the IPD framework.
30215179	Network meta-analysis of cardiac rehabilitation modes (2018)	✗	✓	✗	✓	✓	✗	✓	**Critically low**	C2: no registered protocol; C7: exclusions not justified individually; C13: risk of bias not carried into the netw

PMID	Review	C2 protocol	C4 search	C7 exclusions	C9 RoB in studies	C11 method	C13 RoB in interpretation	C15 publication bias	Confidence	Note
										ork interpretation.
42289790	High-protein diets and renal function without CKD (2026)	✓	✓	✓	✓	✓	✓	✓	**High**	RoB 2 applied to every included trial and GRADE reported by the authors.
30403710	Protein restriction and CKD progression (2018)	✗	✓	✗	✓	✓	✗	✓	**Critically low**	C2: no protocol; C7: exclusions not justified individually; C13: substantial

PMID	Review	C2 protocol	C4 search	C7 exclusions	C9 RoB in studies	C11 method	C13 RoB in interpretation	C15 publication bias	Confidence	Note
										heterogeneity (I^2=76%) not carried into the conclusion.
30935396	Low-protein diet in diabetic nephropathy (2019)	✗	✓	✗	✓	✗	✗	✓	**Critically low**	C11: standardized mean differences pooled across incommensurable proteinuria assays; C2/C7/C13

PMID	Review	C2 protocol	C4 search	C7 exclusions	C9 RoB in studies	C11 method	C13 RoB in interpretation	C15 publication bias	Confidence	Note
										absent.
34673936	Protein interventions and lean mass with/without resistance exercise (2022)	✓	✓	✓	✓	✓	✓	✓	**High**	I^2 of 89–95% is reported and carried into the interpretation rather than hidden.
36057893	Dose–response of added protein and strength training (2022)	✓	✓	✓	✓	✓	✓	✓	**High**	

PMID	Review	C2 protocol	C4 search	C7 exclusions	C9 RoB in studies	C11 method	C13 RoB in interpretation	C15 publication bias	Confidence	Note
37907227	Protein supplementation in sarcopenia (2023)	✗	✓	✗	✓	✓	✗	✗	**Critically low**	C13: handgrip and SPPB results with I^2=94% are described as ‘a tendency for improvement’ despite crossing the null.
40200135	Protein supplementation by physical-activity stratum	✓	✓	✓	✓	✓	✓	✗	**Low**	C15: publication bias not assessed.

PMID	Review	C2 protocol	C4 search	C7 exclusions	C9 RoB in studies	C11 method	C13 RoB in interpretation	C15 publication bias	Confidence	Note
	(2025)									
39214675	Combined strength + aerobic vs aerobic alone in CAD (2024)	✓	✓	✓	✓	✓	✓	✓	**High**	
40671711	Optimal resistance-training dose for handgrip strength in sarcopenia (2025)	✓	✓	✗	✓	✓	✓	✓	**Low**	C7: excluded studies not individually justified.

Summary. 8 High, 0 Moderate, 2 Low, 5 Critically low.

Ratings recalculated at revision. Five overall ratings were recalculated at revision by applying the rule printed above to the critical-domain marks already recorded in

this grid; no domain judgment was changed. 30255969 and 30403710 and 30215179 move from Moderate or Low to **Critically low** (two, three and three critical flaws respectively), and 40200135 and 40671711 move from Moderate to **Low** (one critical flaw each). The earlier ratings had been assigned as global impressions rather than by the stated rule, and the discrepancy was found during an internal audit of this revision. How a Critically low rating is used is set out in Section 2.9: the pooled magnitude is not treated as reliable and no conclusion rests on such a review alone, the estimate is reported with its rating stated where it is used, and the certainty of any body of evidence to which it contributes is rated down. Two of the five (30935396, 37907227) are critically flawed in the pooling itself and support nothing; the reason is stated wherever they appear.

Table S10. GRADE summary of findings

Observational bodies of evidence start at Low certainty and are rated up only for a large magnitude of effect or a demonstrated dose–response gradient; randomized bodies start at High and are rated down for risk of bias, inconsistency, indirectness, imprecision or suspected publication bias. Every up- or down-grade is justified explicitly.

Outcome	Effect	Certainty	Reasoning
Meeting physical activity guidelines → all-cause mortality (observational)	pooled HR 0.76 (0.72–0.79), k=16, I^2=64%	**Low**	Starts Low (observational). Not rated up: the dose–response gradient comes from other analyses (Section 3.3) rather than from this pooled contrast, and stability under leave-one-out (0.747–0.770) is not a GRADE upgrading domain. Seven of the sixteen cohorts contributing to this estimate are High risk of bias on ROBINS-E (thirteen of the twenty-six

Outcome	Effect	Certainty	Reasoning
			appraised observational studies overall), exposure was self-reported in fifteen of the sixteen cohorts and the sixteenth names no instrument, I^2 of 64% (95% CI 29–89%) is not explained by any measured study characteristic (Table S14), and four estimates share NHANES cycles.
Structured exercise → all-cause mortality (randomized)	RR 0.87–0.93 in two pairwise syntheses, both intervals crossing 1; HR 0.83 (0.67–1.04) in an individual-participant meta-analysis; RR 0.76 (0.64–0.90) in a network meta-analysis restricted to center-based cardiac rehabilitation	**Low**	Starts High (randomized). Rated **down** one level for imprecision — three of four pooled confidence intervals include the null, the one that does not is confined to a single delivery format in a single disease and rests partly on indirect comparisons, and the one long trial with mortality as its endpoint (Generation 100) was underpowered and contaminated. Rated **down** a second level for

Outcome	Effect	Certainty	Reasoning
			risk of bias in the contributing syntheses: the individual-participant meta-analysis and the network meta-analysis, which supply the two estimates furthest from the null, are both rated Critically low on AMSTAR 2 after the recalculation described in Table S9, while the two pairwise Cochrane reviews are rated High. Not rated down for inconsistency: the estimates are mutually compatible.
Exercise → cardiovascular mortality (randomized)	RR 0.58 (0.43–0.78) at long-term follow-up	**Moderate**	Starts High. Rated **down** one level for imprecision: eight contributing trials, and the interval is wide on the lower-risk side. Precise in direction and reproduced across Cochrane updates.
Exercise → all-cause hospitalization (randomized)	RR 0.69 (0.56–0.86) in heart failure	**High**	Starts High. Not rated down: the estimate is precise, consistent across updates, and the outcome is directly measured

Outcome	Effect	Certainty	Reasoning
			in the population to which it is applied.
Total protein intake → all-cause mortality (observational)	null across three independent syntheses; our pool HR 0.91 (0.64–1.27), k=5, I^2=81.0%, prediction interval 0.43–1.89	**Very low**	Starts Low. Rated **down** for inconsistency (the cohorts disagree in direction, I^2=80%) and for indirectness of the exposure metric — percentage of energy and quantile contrasts do not identify an absolute intake. A further consideration recorded rather than converted into a downgrade, the rating already being Very low: restricting to studies not rated High risk of bias leaves one cohort, the Rotterdam Study, which reports HR 1.12 (1.01–1.25) — the opposite direction to the pooled null (Table 2, Section 3.5).
Plant protein → all-cause mortality (observational)	pooled HR 0.76 (0.63–0.93), k=4, I^2=65.1%, 95% PI 0.54–1.09	**Very low**	Starts Low; **downgraded to Very low at revision**. The top-up search added the Women's Health Initiative,

Outcome	Effect	Certainty	Reasoning
			which superseded the previous k=3 estimate of 0.69 (0.58–0.82) with I^2=0.0% and removed the homogeneity that was the strongest part of that rating. Rated **down** for imprecision: the 95% prediction interval crosses 1, so a new cohort is not expected to reproduce the effect. Not rated up: k=4, all FFQ-based, residual confounding by overall diet quality and socioeconomic position unresolved, and the stratum falls to a single appraised cohort (SUN, 0.65, 0.45–0.93) when studies rated High risk are removed. The direction is consistent across all four cohorts; the magnitude is not defensible.
Incretin therapy → lean body mass (randomized)	WMD −1.02 kg (−1.46 to −0.57) across 19 trials	**Moderate**	Starts High. Rated **down** one level for indirectness: the outcome is tissue mass, not function, and no

Outcome	Effect	Certainty	Reasoning
			trial in the set measured strength. Not rated down for imprecision — the interval excludes the null and is narrow — nor for inconsistency.
Protein + resistance training during weight loss → fat-free mass, extrapolated to incretin therapy (randomized)	MD +0.30 kg (0.09–0.52)	**Low**	Starts High. Rated **down** twice: imprecision, the interval running from 0.09 kg to 0.52 kg on a body-composition endpoint; and indirectness, because no trial has tested the combination during incretin therapy and the evidence is carried across from weight loss achieved by other means.
Protein supplementation alone → lean mass in older adults (randomized)	WMD 0.34 kg (−0.21 to 0.89); MD −0.01 kg (−3.23 to 3.20)	**Moderate**	Starts High. Rated **down** one level for imprecision. Consistent null across independent syntheses.
Protein supplementation **with** resistance training → strength and lean mass (randomized)	ALM WMD 0.54 kg (0.03–1.05); strength MD +2.01% (1.09–2.93); dose-dependent above 0.5 g/kg/day added protein	**Moderate**	Starts High. Rated **down** one level for very high heterogeneity (I^2 89–95%). Not rated down for imprecision: the direction is

Outcome	Effect	Certainty	Reasoning
			consistent and the dose–response is monotonic.
High-protein diet → kidney function in adults without CKD (randomized)	eGFR SMD 0.39 (0.09–0.69); serum creatinine SMD 0.08 (−0.09 to 0.25)	**Moderate**	Starts High. Rated **down** one level for indirectness: trials are short and measure filtration, not kidney outcomes. The direction of the eGFR change is inconsistent with injury.
Protein restriction in CKD → mortality (randomized)	RR 0.77 (0.51–1.18); RR 1.26 (0.62–2.54); OR 1.17 (0.67–2.06); HR 0.95 (0.62–1.45)	**Low**	Starts High. Rated **down** one level for imprecision: four independent estimates, no signal, and two point estimates above 1. Rated **down** a second level for risk of bias in the contributing evidence: the OR of 1.17 comes from a review rated Critically low on AMSTAR 2 (Table S9), and the hazard ratio of 0.95 comes from the six-year very-low-protein trial rated High risk of bias on RoB 2 (Table S8). The two Cochrane estimates are from a review rated High.
Protein restriction	very-low vs low	**Moderate**	Starts High. Rated

Outcome	Effect	Certainty	Reasoning
in CKD → progression to end-stage kidney disease (randomized)	protein RR 0.65 (0.49–0.85); low vs normal RR 1.05 (0.73–1.53)		**down** one level for risk of bias — adherence to a very-low-protein diet over years is incomplete and unblinded (RoB 2 domain 2, High in the one long trial).

Table S11. Randomized evidence table (full)

Sixteen sources, 32 estimates. Not pooled — the exercise-and-mortality rows are successive updates of overlapping trial sets. Every estimate was located by the deterministic extractor and read in its published context before admission.

Domain	PMID	Year	Design	Population	Contrast	Estimate (95% CI)	Note
PA → all-cause mortality	33028588	2020	RCT (5 y, n=1,567)	community-dwelling adults aged 70–77, generally healthy	HIIT vs control	HR 0.63 (0.33 to 1.2)	Generation 100. The only long randomized trial powered on mortality in a healthy older cohort; MICT vs control showed an absolute increase of 1.2 percentage

Domain	PMID	Year	Design	Population	Contrast	Estimate (95% CI)	Note
							points.
PA → all-cause mortality	34741536	2021	Cochrane MA (25 trials)	coronary heart disease	exercise-based cardiac rehabilitation vs usual care, 6–12 mo	RR 0.87 (0.73 to 1.04)	moderate-certainty (authors' GRADE)
PA → all-cause mortality	34741536	2021	Cochrane MA (15 trials)	coronary heart disease	same, medium-term follow-up	RR 0.9 (0.8 to 1.02)	
PA → all-cause mortality	34741536	2021	Cochrane MA (long term)	coronary heart disease	same, long-term follow-up	RR 0.91 (0.75 to 1.1)	cardiovascular mortality in the same review IS reduced: RR 0.58 (0.43–0.78), 8 trials
PA → all-cause mortality	38451843	2024	Cochrane MA (34 trials)	heart failure	exercise-based cardiac rehabilitation vs usual care, ≤12 mo	RR 0.93 (0.71 to 1.21)	all-cause hospital admission in the same review IS reduced: RR 0.69 (0.56–

Domain	PMID	Year	Design	Population	Contrast	Estimate (95% CI)	Note
							0.86)
PA → all-cause mortality	30255969	2018	IPD meta-analysis	heart failure	exercise cardiac rehabilitation vs control	HR 0.83 (0.67 to 1.04)	ExTraMATCH II
PA → all-cause mortality	30215179	2018	network MA (n=19,411)	coronary heart disease	center-based cardiac rehabilitation vs usual care	RR 0.76 (0.64 to 0.9)	the only randomized all-cause-mortality estimate in this domain that excludes 1
Protein → muscle (with/without training)	34673936	2022	MA of RCTs	older adults	protein vs lower-protein control — total lean body mass	WMD (kg) 0.34 (−0.21 to 0.89)	I^2=90%; null
Protein → muscle (with/without training)	34673936	2022	MA of RCTs	older adults	protein + resistance exercise — appendicular lean mass	WMD (kg) 0.54 (0.03 to 1.05)	I^2=90%
Protein →	34673936	2022	MA of RCTs	older adults	protein +	WMD (kg) 1.71	I^2=89%

Domain	PMID	Year	Design	Population	Contrast	Estimate (95% CI)	Note
muscle (with/without training)					resistance exercise — handgrip strength	(0.12 to 3.3)	
Protein → muscle (with/without training)	34673936	2022	MA of RCTs	sarcopenic / frail only	protein ± exercise — appendicular lean mass	WMD (kg) 0.88 (0.51 to 1.25)	effect confined to this subgroup
Protein → muscle (with/without training)	36057893	2022	dose–response MA (69 articles)	adults	added protein WITH resistance training — muscle strength	MD (%) 2.01 (1.09 to 2.93)	
Protein → muscle (with/without training)	36057893	2022	dose–response MA (69 articles)	adults	added protein WITHOUT resistance training — muscle strength	MD (%) 0.13 (−1.53 to 1.79)	null
Protein → muscle (with/without	36057893	2022	dose–response MA	adults	added protein <0.5 g/kg/day + resistan	MD (%) 1.22 (0.41 to 2.03)	

Domain	PMID	Year	Design	Population	Contrast	Estimate (95% CI)	Note
training)					ce training — strength		
Protein → muscle (with/without training)	36057893	2022	dose–response MA	adults	added protein >0.5 g/kg/day + resistance training — strength	MD (%) 4.29 (1.99 to 6.6)	3.5× the lower-dose effect
Protein → muscle (with/without training)	37907227	2023	MA of RCTs	older adults with sarcopenia	protein supplementation — appendicular skeletal muscle mass	SMD 0.41 (0.24 to 0.58)	I^2=1%
Protein → muscle (with/without training)	37907227	2023	MA of RCTs	older adults with sarcopenia	protein supplementation — handgrip strength	SMD 0.37 (−0.32 to 1.07)	I^2=94%; null
Protein → muscle (with/without training)	40200135	2025	MA of RCTs	older adults, low physical activity	protein supplementation alone — total lean body mass	MD (kg) −0.01 (−3.23 to 3.2)	I^2=0%; null

Domain	PMID	Year	Design	Population	Contrast	Estimate (95% CI)	Note
High protein → kidney (no CKD)	42289790	2026	MA of 22 RCTs (RoB 2 + GRADE)	adults without chronic kidney disease	high-protein vs control diet — eGFR	SMD 0.39 (0.09 to 0.69)	eGFR rises; the authors read this as short-term hemodynamic adaptation, not injury
High protein → kidney (no CKD)	42289790	2026	MA of 19 RCTs	adults without chronic kidney disease	high-protein vs control diet — serum creatinine	SMD 0.08 (−0.09 to 0.25)	I^2=21%; null
Protein restriction in CKD	30403710	2018	MA of 19 RCTs (n=2,492)	chronic kidney disease	low-protein vs control — kidney failure	OR 0.59 (0.41 to 0.85)	
Protein restriction in CKD	30403710	2018	MA of 19 RCTs	chronic kidney disease	low-protein vs control — end-stage renal disease	OR 0.64 (0.43 to 0.96)	
Protein restriction in	30403710	2018	MA of 19 RCTs	chronic kidney disease	low-protein vs	OR 1.17 (0.67 to 2.06)	null, and the point

Domain	PMID	Year	Design	Population	Contrast	Estimate (95% CI)	Note
CKD					control — all-cause death		estimate is above 1
Protein restriction in CKD	33118160	2020	Cochrane MA (5 trials, n=1,680)	non-diabetic CKD	low-protein vs normal-protein — death	RR 0.77 (0.51 to 1.18)	moderate certainty (authors' GRADE)
Protein restriction in CKD	33118160	2020	Cochrane MA (6 trials, n=1,814)	non-diabetic CKD	low-protein vs normal-protein — end-stage kidney disease	RR 1.05 (0.73 to 1.53)	null
Protein restriction in CKD	33118160	2020	Cochrane MA (10 trials, n=1,010)	non-diabetic CKD	very-low-protein vs low-protein — end-stage kidney disease	RR 0.65 (0.49 to 0.85)	moderate certainty
Protein restriction in CKD	33118160	2020	Cochrane MA (6 trials, n=681)	non-diabetic CKD	very-low-protein vs low-protein — death	RR 1.26 (0.62 to 2.54)	null; point estimate above 1
Protein restriction in CKD	34967847	2022	RCT (median 74 months)	advanced CKD	supplemented very-low-	HR 0.95 (0.62 to 1.45)	renal death HR 1.17 (0.88–

Domain	PMID	Year	Design	Population	Contrast	Estimate (95% CI)	Note
					protein vs low-protein — mortality		1.57); ESRD HR 1.12 (0.81–1.56)
Protein restriction in CKD	30935396	2019	MA of RCTs	diabetic nephropathy	low-protein diet — proteinuria	SMD 0.69 (0.22 to 1.16)	
Protein restriction in CKD	30935396	2019	MA of RCTs	diabetic nephropathy	low-protein diet — glycated hemoglobin	SMD 0.17 (−0.18 to 0.51)	null
Training composition and dose	39214675	2024	MA of 23 RCTs (n=916)	coronary artery disease	strength + aerobic vs aerobic alone — cardiorespiratory fitness	SMD 0.26 (0.02 to 0.49)	0.36 (0.05–0.68) when aerobic volume is not reduced
Training composition and dose	40671711	2025	MA of 42 RCTs (n=3,728)	older adults with sarcopenia	resistance training vs control — handgrip strength	MD (kg) 2.69 (1.78 to 3.61)	

Table S12. Measurement error in dietary exposure instruments, and what image-based assessment does and does not fix

Supporting Section 5.8 item 6. Every figure is the one reported by the cited source; nothing here is derived by us.

A. Error in the instruments the field currently uses

Instrument	Reference standard	Reported error	Source
24-hour recall	doubly labeled water	energy under-reported by ~16% (range 10–28%)	PMID 32719809
Food-frequency questionnaire	doubly labeled water	energy under-reported by 26–32%	PMID 32719809
Self-report, population surveys	plausibility screening	31% under-reporting in NHANES II; 46% among women in the UK National Diet and Nutrition Survey	PMID 36639712

An exposure carrying roughly 30% measurement error is being used to argue about hazard ratios of 1.1. That is the first problem, and it is the one the field already acknowledges.

B. Error in automated image-based assessment

Method class	Reported accuracy	Note	Source
Fully automated AI, systematic review against ground truth	average relative error **0.10–38.3%** for calories, **0.09–33%** for volume	**errors were lower for single, simple foods**	PMID 38060823
Image-based assessment, scoping review	typical estimation error **10–15%**	across mixed method families	PMID 39608003
Image-based assessment, meta-analysis	energy still under-reported by **20%** against doubly	the residual bias is not eliminated, only reduced	PMID 38623738

Method class	Reported accuracy	Note	Source
	labeled water		
Reasoning-capable multimodal models fine-tuned on food-energy data	outperform general-purpose vision-language models and some supervised expert systems on the same benchmarks	recent, and benchmark-bound	PMID 40218886

C. Why the two tables have to be read together

The second problem is not the size of the error but its structure. Automated assessment is **least accurate precisely on the mixed plates that carry the exposure of interest** — the accompaniment, not the protein food, is what Section 4.4 identifies as potentially decisive, and the accompaniment is what makes a plate hard to segment. A pooled accuracy figure hides this, which is why the validation requirement below is stated per meal-complexity stratum rather than in aggregate.

A food-frequency questionnaire asks how often red meat is eaten. It does not ask what was on the rest of the plate. The protein food is frequently the smaller part of the meal in both mass and energy — the side, the sauce, the bread, the soup and the dessert routinely contribute several times the calories of the steak — and Section 4.4 reports a dietary pattern defined by *how the protein was accompanied* carrying an odds ratio of 4.40 (1.58–12.22), larger than any single protein-source estimate in this review. This is a limitation of the instrument, not of the analysis, and no statistical adjustment repairs it, because the accompaniment was never recorded.

D. What funders should require, in full

New prospective nutrition cohorts should be required to deliver:

1. **Image capture of the whole eating occasion**, rather than food-level recall.
2. **Validation against weighed food records *and* doubly labeled water, reported per meal-complexity stratum** rather than pooled, since the pooled figure hides the failure mode identified in section C above.
3. **Protein reported in grams and grams per kilogram alongside percentage of energy**, so that the discrepancy documented in Section 3.11 — the same patients adequate on the relative metric and deficient on the absolute one — becomes visible rather than invisible.
4. **Retention of the images, or of a derived plate-composition vector**, so that the food-matrix hypothesis can be tested retrospectively rather than only invoked as a limitation.

5. **Prespecified handling of algorithmic error as measurement error** in the analysis, with the seriousness now applied to self-report bias.

This is a proposal for an instrument, not a result. The claim is only that the question in Section 4.4 — *does the accompaniment matter more than the meat?* — is currently unanswerable with the data the field collects, and becomes answerable with data the field could now collect.

Table S13. Conduct against the Cochrane Rapid Reviews Methods Group recommendations

PRISMA 2020 is a reporting guideline. The conduct standard that applies to a rapid review is the Cochrane Rapid Reviews Methods Group (RRMG) guidance (Garritty et al., *J Clin Epidemiol* 2021; PMID 33068715), updated for reviews of effectiveness in 2024 (PMID 38320771). This table maps the review against the recommendations that bear on the two criticisms it is most exposed to — single-reviewer screening and a single-database analytic corpus — and against the extraction and appraisal recommendations that travel with them. Recommendation numbers are those of the 2021 guidance.

Verdicts are not graded on a curve. *Met* means the recommendation was followed as written. *Exceeded* means more was done than the recommendation asks. *Partly met* and *Not met* are stated for what they are, and each carries the consequence rather than a justification alone.

#	What the guidance recommends	What this review did	Verdict
**R10**	Limit main database searching to CENTRAL, MEDLINE (e.g. via PubMed) and Embase, where access allows	**MEDLINE via PubMed** was the analytic corpus: 58 logged queries in ten tranches, 7,544 unique records (67 distinct query strings once the nine date-floor re-runs of the revision are counted; those re-runs lie outside the 7,544), from which every extracted estimate	**Partly met.** One of three recommended databases was searched. The consequence is stated in Section 2.8 item 3 and is not softened: the analytic corpus is single-database and the coverage check is multi-database. Trials indexed in CENTRAL or

#	What the guidance recommends	What this review did	Verdict
		and every pooled estimate comes. **CENTRAL and Embase were not searched** — no institutional Embase license was available. Coverage was instead tested against **Europe PMC, Semantic Scholar and ClinicalTrials.gov** (211 logged executions, Tables S1 and S1b) and by backward and forward citation chasing on every included reference. Europe PMC returned 3,405 records seen in no other source.	Embase but not MEDLINE could not enter a pooled estimate.
**R10**	Involve an information specialist	K.S. acted as the review's information specialist: the search architecture, the ten tranches, every query string and every re-run were designed and executed by him against the NCBI E-utilities, Europe PMC, Semantic Scholar and ClinicalTrials.gov	**Met.** The role was filled from within the review team rather than by an external librarian, which is what the recommendation permits; the reproducibility of the log is what makes the claim checkable.

#	What the guidance recommends	What this review did	Verdict
		APIs. All 211 executions are reproduced verbatim with execution timestamps, true match counts and retrieved counts (Tables S1, S1b), so the strategy can be audited and re-run rather than taken on trust.	
**R14**	Dual-screen **at least 20%** (ideally more) of titles and abstracts, with conflict resolution	The sampling frame was the enlarged multi-source corpus assembled at revision (Table S1b), not the 7,544-record PubMed analytic corpus, so the strata below total more records than that corpus; the sample was drawn after a title-level exposure filter, and the agreement statistics therefore describe screening decisions on records already naming an exposure in their title (Section 2.9). The frame was stratified before sampling. **Stratum A** — topical	**Met, and exceeded in one respect.** The 20% applies to the stratum where inclusion decisions are made, and the guidance's target is met there exactly. Sampling two further strata to estimate the false-negative rate of the screening gates is not asked for by the guidance and is reported in Section 2.9.

#	What the guidance recommends	What this review did	Verdict
		records carrying a ratio estimate with a 95% confidence interval, i.e. the only records that can move a pooled estimate — contains **2,019 records, of which 406 (20.1%) were dual-screened**. Two further strata were sampled to measure the false-negative rate of the automated gates: **B** (topical, no extractable estimate) 308 of 16,230, and **C** (not topical) 174 of 27,504. One further record, admitted by the top-up search after sampling, was screened by both reviewers, giving **889 in duplicate**. All **149 disagreements** were adjudicated by a third reviewer.	
**R14**	One reviewer screens the remaining abstracts; **a second reviewer screens all excluded abstracts**	The remaining abstracts were screened by one reviewer (K.S.). **No second reviewer screened all exclusions.** What was done instead	**Not met.** This is the single largest conduct shortfall in the review, and it is the reason Section 2.8 item 2 tells a reader to treat the corpus as

#	What the guidance recommends	What this review did	Verdict
		is the stratified false-negative measurement above, which estimates the exclusion error rate rather than eliminating it.	single-reviewer-screened with a validation sample, not as duplicate-screened.
**R15**	Full-text screening: one reviewer screens included articles, a second screens all excluded	Full-text screening and the admission of individual estimates were performed by one author (K.S.), each estimate read in its source context. **No second reviewer screened full-text exclusions.**	**Not met.** Stated in Section 2.8 item 2.
**R16**	Single reviewer extracts data using a piloted form; **a second reviewer checks correctness and completeness**	Extraction was **deterministic and language-model-free** — regular-expression parsers over full text and abstracts, in two measure families never merged (Section 2.4) — so the extraction step is reproducible by re-running the code in File S1 rather than by a second pair of eyes. Every estimate admitted to an analysis was then read in its source context and adjudicated by one	**Not met as written.** Determinism answers *correctness of transcription*, which is what a second checker usually catches; it does not answer *completeness*, and Section 4.6 documents two extraction defects found by internal audit that a second reviewer might have found earlier. Section 4.7 states the completeness limit: estimates reported only in

#	What the guidance recommends	What this review did	Verdict
		author. **No second reviewer independently checked the extracted set.**	figures or in tables whose layout does not survive text extraction were missed.
**R19**	Single reviewer rates risk of bias, with **full verification** of all judgments by a second	Risk of bias was rated **in full duplicate** — every study rated independently by V.G.-O. and C.D. with K.S. adjudicating disagreements — using ROBINS-E (26 observational studies), RoB 2 (2 trials) and AMSTAR 2 (15 reviews), with a Newcastle–Ottawa cross-check on the same 26 and the disagreement between instruments reported rather than reconciled (Section 3.10, Tables S7, S7b).	**Exceeded**, with one exception recorded in Document S1 item 8: the Women's Health Initiative, admitted at revision, has not yet been rated.
**R19**	Limit risk-of-bias rating to the most important outcomes	Rating covered every observational study entering a synthesis and every trial, not a subset.	**Exceeded.**

What this table is for

The two criticisms this review will attract are that screening was single-reviewer outside a validation sample and that the analytic corpus is one database. Both are true, both are declared in Section 2.8, and both are permitted abbreviations under a named rapid-review standard — but not without cost, and the cost is what the right-hand column states. Four rows are *Not met* or *Partly met*. A reader who concludes from them that this review does not meet systematic-review conduct standards is correct, and is reaching the conclusion the Methods intend: it is a rapid review, conducted and reported as one.

Table S14. Heterogeneity and sensitivity analyses of the physical activity stratum (k = 16)

Added at revision in response to peer review. All models are random-effects (REML, `metafor` 5.0.1, R 4.6) on the log hazard ratio, standard errors derived from the reported 95% confidence limits (Section 2.6). The meta-regressions are exploratory: several moderators were tested at k = 16, no correction for multiple testing was applied, and none is used to support a conclusion. Generated by the heterogeneity script supplied in File S1.

A. Overall model

Statistic	Value
Pooled HR (95% CI), Wald	0.76 (0.72–0.79)
Pooled HR (95% CI), Knapp–Hartung	0.76 (0.72–0.80)
95% prediction interval	0.65–0.87
Cochran's Q (df = 15)	60.45, p <0.001
τ^2 (95% CI, Q-profile)	0.0048 (0.0011–0.0221)
I^2 (95% CI, Q-profile)	63.9% (28.8–89.1%)
Point estimates below 1 / confidence intervals excluding 1	16 of 16 / 16 of 16

B. Subgroups

Subgroup	k	HR (95% CI)	I^2
Generally healthy	5	0.73 (0.68–0.78)	51.4%
Established chronic disease	11	0.77 (0.73–0.82)	47.2%
Guideline-threshold contrast	11	0.75 (0.71–0.80)	41.8%
Extreme-category contrast	5	0.76 (0.70–0.83)	82.4%
East Asia	9	0.74 (0.70–0.79)	66.4%

Subgroup	k	HR (95% CI)	I^2
Other regions	7	0.79 (0.75–0.84)	17.3%

C. Univariable meta-regression

Moderator	k	Q_M (df)	p	R^2	Slope / contrast (95% CI)
Setting (chronic disease vs generally healthy), log HR	16	1.71 (1)	0.191	27%	0.0624 (-0.0312 to 0.1560)
Exposure definition (extreme category vs guideline threshold), log HR	16	0.08 (1)	0.772	0%	0.0155 (-0.0892 to 0.1201)
Region (East Asia vs other), log HR	16	0.97 (1)	0.326	13%	-0.0494 (-0.1480 to 0.0491)
Publication year, log HR per year	16	0.91 (1)	0.339	0%	0.0107 (-0.0112 to 0.0326)
Standard error of the estimate	16	2.13 (1)	0.144	0%	-1.0355 (-2.4259 to 0.3550)
Length of follow-up, log HR per year	16	1.37 (1)	0.242 (permutation 0.291)	13%	-0.0079 (-0.0210 to 0.0053)

Follow-up duration could be established for 14 of 16 cohorts (range 3.2–20.0 years). Activity was measured by self-report in every cohort for which the method could be established (10 of 16); no cohort used a device-based measure, so measurement method could not be examined as a moderator.

D. Influence and leave-one-out

Cohort omitted	Weight (%)	HR without it	I^2 without it	Cook's D	DFFITS	Baujat: contribution to Q	Baujat: influence on the pooled estimate
[34]	12.7	0.770	39.3%	0.546	-0.940	2.70	0.6116
[37]	11.4	0.747	53.7%	0.224	+0.506	1.57	0.2160
[39]	8.4	0.753	66.2%	0.019	+0.133	0.13	0.0164
[32]	8.3	0.754	66.5%	0.009	+0.089	0.04	0.0073
[20]	8.3	0.748	59.8%	0.191	+0.458	2.17	0.1917
[21]	7.5	0.760	65.0%	0.037	-0.191	0.47	0.0336
[40]	6.9	0.758	66.2%	0.008	-0.088	0.14	0.0072
[31]	5.8	0.752	65.7%	0.045	+0.209	0.61	0.0409
[38]	5.8	0.753	66.0%	0.034	+0.182	0.44	0.0308
[36]	5.8	0.751	65.0%	0.068	+0.260	1.01	0.0633
[35]	5.3	0.761	64.5%	0.056	-0.236	0.94	0.0530
[33]	4.2	0.758	66.1%	0.006	-0.075	0.16	0.0054
[22]	4.1	0.757	66.4%	0.001	-0.025	0.03	0.0006
[23]	3.2	0.762	62.9%	0.096	-0.313	2.70	0.0982
[41]	1.3	0.757	65.5%	0.005	-0.068	0.37	0.0045
[19]	1.1	0.759	64.8%	0.025	-0.159	2.18	0.0252

Standardized influence diagnostics (`metafor::influence.rma.uni`) and Baujat coordinates. Reference thresholds for k = 16: Cook's D > 0.455 (the median of χ^2_1) and |DFFITS| > 0.775 ($3\sqrt{(1/(k-p))}$). Only [34] exceeds either, and it exceeds both; the Baujat map agrees, placing it at the largest influence on the pooled estimate (0.61). The largest contributors to heterogeneity are [23] (2.70), [19] (2.18) and [20] (2.17), none of which is influential on the estimate itself — the pattern of a precise cohort disagreeing with imprecise ones rather than of a single study driving the result.

E. Small-study effects

Test	Result
Egger's regression (predictor: standard error)	z = -1.46, p = 0.144
Trim-and-fill	3 studies imputed; adjusted HR 0.77

Test	Result
	(0.73–0.81)

With 16 studies neither test has adequate power, and funnel asymmetry can reflect heterogeneity rather than selective publication; a non-significant result is not evidence that publication bias is absent.

F. Overlap of parent cohorts (NHANES)

Four chronic-disease estimates derive from overlapping NHANES cycles: [20] (1999–2018), [21] (1999–2018), [22] (2007–2014) and [23] (2005–2018).

Analysis	k	Pooled HR (95% CI)	I^2	Chronic-disease subgroup HR (95% CI), k	Setting moderator Q_M, p
As reported (all 16)	16	0.76 (0.72–0.79)	63.9%	0.77 (0.73–0.82), 11	1.71, 0.191
All four NHANES estimates removed	12	0.76 (0.72–0.80)	63.4%	0.80 (0.76–0.84), 7	3.87, 0.049
Only [20] retained	13	0.77 (0.73–0.81)	66.4%	0.81 (0.78–0.85), 8	6.23, 0.013
Only [21] retained	13	0.75 (0.72–0.79)	61.9%	0.78 (0.73–0.83), 8	2.10, 0.148
Only [22] retained	13	0.76 (0.72–0.80)	60.6%	0.79 (0.75–0.84), 8	3.66, 0.056
Only [23] retained	13	0.75 (0.71–0.79)	64.3%	0.77 (0.72–0.83), 8	1.57, 0.211
Four NHANES estimates combined (fixed effect) into one	13	0.76 (0.72–0.80)	62.3%	0.79 (0.75–0.83), 8	4.27, 0.039

Table S15. Analyses planned at first draft, added at internal revision, and added in response to peer review

This table separates what the review set out to do from what was added later, so that no addition can be read as pre-specified. The protocol of record (Document S2) is retrospective, so "planned at first draft" means present in the first complete draft of the analysis and not described anywhere in the manuscript or its supplements as a later addition. "Internal revision" refers to the revision carried out by the authors before submission; "peer review" refers to changes made in response to the reviewers of this submission. Where an addition changed a reported estimate, the change is stated.

A. Planned at first draft

Component	Where reported
PubMed search, 58 logged queries in ten tranches (the hypertrophy and mediator tranches excepted; see B)	Section 2.2, Table S1
Deterministic regular-expression extraction of ratio estimates, with contextual reading by an author before admission	Section 2.4
Separate pooling by effect measure and by single contrast definition; one estimate per cohort per stratum; primary cohorts and published meta-analyses in separate layers	Sections 2.5–2.6
Random-effects REML models; I^2, τ^2, prediction intervals; leave-one-out; Egger's test where $k \geq 10$	Section 2.6
Generally healthy versus established chronic disease as a subgroup contrast for physical activity, and clinical population as a subgroup dimension	Section 3.2; Document S2
Tabulation, without pooling, of population-stratified protein estimates in absolute units	Sections 2.7, 3.6; Table S4
Risk of bias with ROBINS-E, RoB 2 and AMSTAR 2, rated in duplicate with third-author adjudication; GRADE	Section 2.9; Tables S7–S10

Prespecified but not performed. Two subgroup dimensions named in the protocol (Document S2, field 27) were not analyzed and are recorded here rather than left unaccounted for: *age stratum*, because the cohorts report age bands that are not comparable across studies and too few estimates share a band to support a moderator test; and *exposure metric (absolute units versus percentage of energy)*, which cannot be tested in this corpus because Section 2.7 excludes percentage-of-energy contrasts from the population-stratified analysis and no pooled stratum contains estimates of both kinds.

B. Added at internal revision (before submission)

Addition	Reason	Effect on reported results	Where reported
Capped queries re-executed without a retrieval cap; nine core queries re-run with a 1990 date floor	Completeness	Two pooled analyses recovered [10], [17]	Section 2.2; Document S4
Europe PMC, Semantic Scholar, ClinicalTrials.gov and citation chasing	Test the coverage of the PubMed corpus	Used to test coverage and supply the screening sample, not to extract estimates	Section 2.2; Table S1b
Rule-based exclusion of conference abstracts	Required once sources indexing abstracts were added	3,275 records excluded	Section 2.3
Hypertrophy dose–response and mediator tranches	Source the muscle-accrual block of Figure 5 and the second link of the calculation in Section 4.5	Inform one figure block and one Discussion calculation; no pooled estimate	Document S3
Physical-activity expansion (2010–2026), adding 7 of the 16 cohorts in the activity stratum	The original core search began in 2023	HR and I^2 changed from the k = 9 analysis (I^2 34%) to k = 16 (HR 0.76, I^2 64%)	Section 3.2; Document S4
Top-up search on plant-versus-	Contrast-specific completeness	Plant-protein estimate re-	Section 3.5; Document S1

Addition	Reason	Effect on reported results	Where reported
animal protein contrasts, adding the Women's Health Initiative		estimated; the added cohort is unrated and is handled by bounding	
Setting moderator applied to the total-protein stratum	Symmetric extension of the pre-specified activity subgroup	Exploratory; after the population recoding and the estimate removal described in Section C it shows no difference between settings (0.93 vs 0.83, Q_M p = 0.79), and the chronic-disease side has k = 2 and is reported as a subgroup description rather than a pooled result	Section 3.5; Document S4
Duplicate screening of a stratified random sample (888 records, 889 with the record admitted after the sample was drawn)	Estimate the reproducibility of single-reviewer screening	κ = 0.50, PABAK = 0.67	Section 2.9; Document S4
Newcastle–Ottawa cross-check (single rater)	Compare with ROBINS-E	Informs no grade and no conclusion	Section 2.9; Table S7b
GRADE for plant protein downgraded from Low to Very low	k = 4, all food-frequency questionnaires, I^2 65%, prediction interval crossing 1	Certainty rating lowered	Section 3.10; Table S10
Randomized	Compare	New evidence	Sections 2.5, 3.9;

Addition	Reason	Effect on reported results	Where reported
tranche and randomized evidence table	observational with randomized evidence	layer, not pooled	Table S11
Four population rows in Figure 5 (adults aged ≥85 years, disease-related malnutrition, kidney transplant recipients, critical illness including acute kidney injury)	Evidence introduced at revision	New rows in Figure 5	Section 5.5

C. Added in response to peer review (this revision)

Addition	Reviewer comment	Effect on reported results	Where reported
Dedicated statistical-analysis subsection, including pooling eligibility, contrast compatibility and the rationale for the three-study minimum	R2.7; R1.m3; R1.m4	None (description of rules already applied); Egger threshold corrected from $k \geq 9$ to $k \geq 10$, which is the threshold the pooling script applied	Section 2.6
95% confidence intervals for τ^2 and I^2	R1.M2	I^2 64% (29–89%)	Section 3.2; Table S14
Exploratory meta-regression (setting, exposure definition, region, publication year, standard error, follow-up)	R1.M2	No moderator significant, each tested at $k = 16$	Section 3.2; Table S14
Standardized influence diagnostics	R1.M2; R2.7	One influential cohort [34], exceeding both	Table S14

Addition	Reviewer comment	Effect on reported results	Where reported
(Cook's D, DFFITS) and Baujat coordinates		thresholds; the largest contributors to heterogeneity are not the influential ones; pooled estimate unchanged	
Knapp–Hartung and trim-and-fill sensitivity analyses	R1.m6; R2.7	HR 0.76 (0.72–0.80) and 0.77 (0.73–0.81)	Section 3.2; Table S14
Parent-cohort (NHANES) overlap sensitivity analyses	Identified by the authors while addressing R1.M2	Overall estimate unchanged; general-versus-clinical difference becomes nominally significant in some specifications, so the claim of equal magnitude was withdrawn	Sections 2.6, 3.2; Table S14
Correction of citations attached to the wrong statement, including two unrelated PMIDs in Section 3.13	R1.m9	None to any pooled estimate; the statements now match their sources	Section 3.13; Document S1
Red meat and type 2 diabetes relabeled to the per-50 g/day contrast, RR 1.11 (1.06–1.16)	R1.m9	The cited value changes; the estimate enters no pool	Sections 3.7, 4.4, 5.7; Table 8
Population recoding of the KoGES cohort (to CKD stage 3–5),	Identified by the authors while re-reading source populations for	Total protein 0.91 (0.64–1.27), k = 5, I^2 = 81%, prediction interval	Sections 2.6, 3.5, 4.2; Tables 2, S3, S4

Addition	Reviewer comment	Effect on reported results	Where reported
and removal of the NHANES estimate from the pooled stratum because the source reports it only within an eGFR stratum and only as a cut-point contrast, which Section 2.6 admits in neither respect	R1.M2	0.43–1.89; the exploratory setting split does not differ (Q_M $p = 0.79$); the NHANES estimate is retained in the population table only	
GRADE certainty for the activity–mortality association lowered from Moderate to Low	R1.M7; R2.8	Certainty rating lowered; the certainty labels on the recommendations in Sections 5.1 and 5.4 change with it	Section 3.10; Tables 5, S10
Confidence labels in Table 6 aligned to GRADE wording	R1.M7; R2.8	Labels only; no window changes	Table 6
Correction of three transcribed confidence intervals and one population label in Table 1	Identified by the authors while verifying estimates for R1.m9	None: the analysis files held the correct values	Section 3.2, Table 1

Document S1. Log of citation and pipeline errors identified during this review

Every defect found in our own tooling or in the published literature during this synthesis, with what it would have cost had it gone undetected and what was done about it. Section 4.6 of the manuscript summarizes the first three; this document is the complete log, including defects found after that section was written.

1. Silent extraction failure — `pdftotext` returning success on empty output

What happened. The utility was invoked in a form that failed silently in our environment, returning exit code 0 with empty output. All 825 PDFs in the first harvest fell back to abstract-only text without raising an error.

Cost if undetected. The analysis would have rested on abstracts alone for a third of the corpus, with no signal that anything was wrong.

Correction. After the invocation was fixed, the extracted set grew from 638 to **2,566 estimates**. Found by validating output against sentences read by hand, not by any internal check the pipeline performs on itself. That figure is the corpus as it stood at that point; with the search tranches added later the final extracted set is 8,304 estimates (Table S2).

---

2. Sign-absorbing regular expression in the mean-difference extractor

What happened. The pattern written for the randomized layer allowed the character gap preceding a confidence limit to absorb a minus sign, so a published `95% CI: -0.21, 0.89` was captured as `0.21 to 0.89` — turning a null result into a significant one, and in the wrong direction for our own argument.

Cost if undetected. Sign errors on an unknown number of difference-measure estimates, biased toward supporting our thesis.

Correction. Surfaced on a single estimate whose source sentence was being read for another purpose (PMID 34673936). The pattern was rewritten to exclude every dash character from that position, a six-case regression check was added, and the extraction was re-run from scratch. The difference-measure set went from 841 to **1,634 estimates** with the signs correct.

We assume there are others we have not found. Both defects were caught by hand-checking, not by the pipeline auditing itself.

---

3. Misattributed effect estimate in a published review

What happened. PMID 42158256 cites the Newcastle 85+ study as showing “higher sarcopenia incidence over 3 years among those consuming ≥1 g/kg body weight per day of protein (OR = 2.57, 95% CI = 1.26–5.26)”.

What the source actually says. We retrieved it (Granic *et al.*, *Clin Nutr* 2020;39:166–173; PMID 30709690, PMC6961212) and verified that **OR 2.57 is the comparison of dietary pattern DP2 ("Traditional British") versus DP3 ("Low Butter") for three-year prevalent sarcopenia**, adjusted Model 3. The figure ≥1 g/kg aBW/day appears in that paper only as the cut-point defining the "good protein intake" stratum. The source concludes the opposite of the citation: the harmful dietary pattern raised sarcopenia risk **even when protein intake was good** (OR 5.45, 1.81–16.39).

Cost if undetected. It was the sole apparent contradiction to an otherwise consistent finding in older adults, and would have been reported as such.

Correction. The estimate was excluded from our analysis.

---

4. A date floor, not a database, excluded two pivotal cohorts

What happened. Nine core queries carried a 2023 date floor. Two of the most important cohorts in this field — Arem 2015 (PMID 25844730) and Stamatakis 2018 (PMID 29099919) — were therefore never retrieved, and the omission looked like a database-coverage problem rather than a filter problem.

Correction. The nine queries were re-executed with the floor removed (the `-1990` variants in Table S1b), both cohorts were recovered, and they are now references in Sections 2.2, 3.3, 5.1 and 5.2.

---

5. A pooled estimate that did not survive a missing cohort

What happened. The plant-protein stratum was reported as **HR 0.69 (0.58–0.82), k = 3, I^2 = 0.0%**. A top-up search at revision found a cohort that met our contrast definition exactly and that the original search had missed: the Women's Health Initiative (PMID 33624505), 102,521 women and 25,976 deaths, the largest cohort in the stratum by a wide margin.

Cost if undetected. The homogeneity was the more persuasive half of the original claim, and it was an artifact of which cohorts we happened to have.

Correction. With four cohorts the estimate is **HR 0.76 (0.63–0.93), I^2 = 65.1%**, prediction interval 0.54–1.09. Table 2 carries the superseded value so the change is visible rather than silent. Section 3.5 states it explicitly. The bounds of the risk-of-bias-restricted plant-protein result under both possible ratings for the unrated cohort are given in item 8 below; Section 3.5 states only that the conclusion is unchanged under either.

---

6. A screening rule whose safety calibration had gone stale

What happened. A title-level exposure rule removed 21,699 corpus records on the strength of a calibration showing it discarded **0 of 101** cited references. That calibration predates the review's sarcopenia and microbiome material. Against the current 146-reference list the same rule discards **12** — every sarcopenia, muscle-mass, muscle-wasting and gut-microbiota title, which is precisely the evidence Sections 3.12 and 3.13 now rest on.

Cost if undetected. A screening filter presented as calibrated and safe while systematically removing one of the review's own evidence streams.

Correction. Disclosed in Section 2.9: the duplicate-screened validation sample was drawn after this filter was applied, so the agreement statistics describe records that already name an exposure in their title and would not transfer unchanged to an unfiltered corpus.

---

7. A projection withdrawn after publication-internal review

What happened. An earlier draft carried a Monte Carlo projection multiplying the randomized effect on muscle by the observational hazard of low muscle mass. It returned implied hazard ratios near 0.97 — roughly an order of magnitude smaller than the 20–30% mortality differences the cohorts of Section 3.2 report, because it modeled only the muscle-mass pathway rather than the total effect of activity.

Cost if undetected. Printed beside those cohorts it invited the reading that the whole prescription is worth about three per cent, at a cost of hundreds of thousands of participants.

Correction. Withdrawn. Section 4.5 states the withdrawal and the reason, and makes the qualitative case instead. The corresponding supplementary table and figure were removed.

8. A cohort added at revision that the risk-of-bias appraisal never reached

What happened. The top-up search run at revision on plant-versus-animal protein contrasts identified the Women's Health Initiative (PMID 33624505; 102,521 postmenopausal women, 25,976 deaths) and it was admitted to the plant-protein stratum, where it is by a wide margin the largest cohort and where it moved the pooled estimate from HR 0.69 (I^2 = 0.0%) to HR 0.76 (I^2 = 65.1%). The duplicate ROBINS-E and Newcastle–Ottawa appraisal had been completed before that search was run, and the cohort was never routed back through it. Tables S7 and S7b therefore cover twenty-six studies, while the plant-protein synthesis rests on four.

How it was found. A cross-reference audit of the manuscript against the appraisal tables, run while checking that every in-text figure and table reference resolved. The sensitivity paragraph in Section 3.5 still read “two of the three plant-protein cohorts”, a count that had been correct before the top-up search and was not updated with the pool.

Why it matters. The risk-of-bias-restricted sensitivity analysis is one of the central honesty claims of this review: it is what establishes that the protein pooling layer would not survive a restriction to low-risk exposure measurement. An unrated cohort inside the stratum being tested weakens that claim, and doing the arithmetic over four cohorts while only three had been appraised would have overstated the coverage of the analysis.

What was done. The cohort is carried in the pooled estimate, where it was pre-specified to belong, and is reported as *unrated* in the sensitivity analysis, which is stated in Section 3.5 to be an analysis of the three appraised cohorts rather than of the four that produce the headline estimate. The alternatives — dropping the largest cohort in the stratum, or generating a rating outside the duplicate procedure the Methods describe — would each have been worse: the first discards evidence, and the second would report an appraisal that the stated process did not produce.

Inclusion is settled; appraisal is not. The cohort was screened independently by V.G.-O. and C.D. at revision and admitted by both, and that decision is logged separately from the 888-record duplicate-screening frame so that it cannot alter the agreement statistics computed on it. Inclusion and risk of bias are separate instruments, and a unanimous inclusion decision is not a ROBINS-E rating.

Bounded rather than left open. Section 3.5 states that the conclusion is unchanged under either possible rating for this cohort; the two bounds are given here. If it were rated High, the restricted set is one cohort, HR 0.65 (0.45–0.93), not poolable. If it were rated Some concerns or Low, the set is two cohorts, HR 0.81 (0.59–1.11), I^2 = 69% — below our three-study minimum and no longer excluding the null. No conclusion in the review turns on which of the two is correct, so the missing rating is disclosed but not load-bearing. The code is `protein_setting_moderator.R` in File S1.

Status. This cohort is reported as unrated, and the review makes no claim that it was appraised. The bound above is what the manuscript relies on; a rating, if added later, would replace a bound with a value and change the counts in Section 3.10 and Tables S7 and S7b, but would not change a result.

Document S2. Protocol — PROSPERO registration form (all fields)

Status of this document. This protocol is written in the PROSPERO field structure so that it can be submitted verbatim. It is **retrospective**: the searches were executed on 22–23 August 2026 and data extraction was complete before the protocol was written down. PROSPERO does not accept registrations for reviews in which data extraction has been completed, and we will not misrepresent the timeline in order to obtain a registration number. **Handling:** the review is reported as *not registered* under PRISMA 2020 item 24a, and this document is the protocol of record. It is supplied with the manuscript as Supplementary Document S2 rather than deposited in an external repository, so it carries no independent time stamp and should not be read as though it did — its authority rests on being complete and on marking, field by field, what was decided before the data were seen and what after.

---

1. Review title

Protein Intake and Resistance Training Across Clinical Populations: A PRISMA-Guided Rapid Review of Observational and Randomized Evidence.

(The working title at the time this retrospective protocol was written was "Physical activity thresholds and population-stratified dietary protein targets for healthspan and lifespan". The change reflects the shift of the review's axis onto protein and sarcopenia at revision; the questions, eligibility criteria and analysis plan below are unchanged. The title was changed again at peer review to name the design explicitly.)

2. Original language title

Not applicable — the review is written in English.

3. Anticipated or actual start date

2026-08-22 (first logged search).

4. Anticipated completion date

2026-09-30.

5. Stage of review at time of this registration

At the time this protocol was written down: data extraction **completed**; formal risk-of-bias assessment and GRADE in progress; synthesis complete for the

observational layer. Declared, not concealed. All of these steps were completed before submission, and the risk-of-bias and certainty assessments are reported in full in Tables S7–S10.

6. Named contact

Károly Szili, MD MBA PhD — szili.karoly@sze.hu (corresponding author for this protocol). The manuscript has two corresponding authors: Sándor Nagy (nsandor@sze.hu) and Károly Szili.

7. Named contact email / address

Department of Obstetrics and Gynecology, Faculty of Health and Sport Science, Széchenyi István University, Egyetem tér 1, 9026 Győr, Hungary.

8. Organizational affiliation of the review

Széchenyi István University, Győr, Hungary; University of Szeged, Albert Szent-Györgyi Medical School, Szeged, Hungary.

9. Review team members and their organizational affiliations

Affiliations as given in the manuscript byline (superscripts 1 = Department of Obstetrics and Gynecology, Faculty of Health and Sport Science, Széchenyi István University; 2 = Department of Obstetrics and Gynecology, Albert Szent-Györgyi Medical School, University of Szeged; 3 = Doctoral School of Regional Sciences and Business Administration, Széchenyi István University): Csilla Dézsi (1, 3; joint first author); Viktor Gulyás-Oldal (1, 2, 3; joint first author); Ekaterine Paschali (1, 3); Imre Gálóczi (1, 3); Sándor Nagy (1, 3; corresponding author); Károly Szili (1, 2, 3; corresponding author).

10. Funding sources / sponsors

None. No external funding was received.

11. Conflicts of interest

None declared by any author. No author holds a commercial relationship with a protein supplement, sports nutrition, meat industry, or physical-activity technology manufacturer.

12. Collaborators

None.

13. Review question

1. Where does the physical-activity dose–response curve for all-cause mortality actually bend, and does the association differ between generally healthy populations and populations with established chronic disease?
2. Is the protein–mortality relationship better described by amount, by source, or by neither?
3. When protein intake is expressed in **absolute units** (g/kg body weight per day, g/kg ideal or adjusted body weight per day, g/day) rather than as a percentage of total energy, and the population is specified (generally healthy, obesity, type 2 diabetes, cardiovascular disease, chronic kidney disease, pregnancy), does a single population-wide recommendation remain tenable?
4. Does randomized evidence agree with the observational evidence on each of these questions, and where it does not, which divergences are systematic?
5. How far does the chain from protein, through muscle and sarcopenia, to mortality actually hold, and at which link does it stop being demonstrated? *(Added at revision, when the review's axis moved onto protein and sarcopenia; recorded as a later addition in Table S15.)*

14. Searches

PubMed via the NCBI E-utilities API. Each query string was logged verbatim at execution time together with the database, UTC timestamp, PubMed's true match count and the number of records retrieved (the machine-readable search log, supplied as the PRISMA-S appendix). Ten tranches, 58 queries: - **Core** (8 queries, 2023-01-01 to 2026-12-31): physical-activity dose–response and mortality; activity guidelines and healthy aging; protein and longevity; protein dose and sarcopenia; combined resistance training and protein; sports nutrition and healthspan; steps/VILPA and mortality; protein restriction, mTOR and IGF-1. - **Supplementary** (4 queries, 2015-01-01 to 2026-12-31): protein–mortality meta-analyses; high-protein safety and cardiovascular outcomes; protein and mortality in older adults; protein in UK Biobank and the US National Health and Nutrition Examination Survey (NHANES). - **Population** (7 queries, 2015–2026), framed in absolute intake units: generally healthy adults, obesity, type 2 diabetes (×2), cardiovascular disease, chronic kidney disease, and an explicit upper-intake / safety query. - **Microbiome** (5 queries, 2015–2026): gut microbiota and short-chain fatty acids against sarcopenia and muscle outcomes; against dietary protein and proteolytic fermentation metabolites; against exercise and lactate; against longevity; and an explicit gut–muscle-axis query. **Declared as a relevance-ranked sample, not a search**: the queries matched 20,872 records and retrieved 1,250. - **Dietary assessment** (4 queries, 2018–2026): AI and image-based dietary assessment; validation against weighed records and doubly labeled water; automated protein monitoring; and self-report measurement error. Also a sample rather than a search. - **Incretin** (6 queries, 2015–2026): GLP-1 receptor agonist and dual incretin exposure against lean mass,

sarcopenia and muscle strength; against dietary protein and supplementation; against resistance training; muscle-preserving weight loss; clinician guidance; and a randomized-design-filtered query. - **Physical-activity expansion** (5 queries, 2010–2026): guideline adherence; most-active-versus-inactive quantile contrasts; device-measured activity; clinical populations; pooled and consortium cohort analyses. The window was widened because the cohort literature defining the pooled activity contrast predates the core tranche. - **Hypertrophy dose–response** (4 queries, 2010–2026): protein supplementation with resistance training against dose–response, plateau and break-point terms; athlete and resistance-trained protein requirements including position stands; indicator amino acid oxidation and nitrogen-balance requirement studies; and very-high or supraphysiological intakes in randomized designs. Added at revision to source the muscle-accrual block of Figure 5, which is a different endpoint from every other bar in that figure; matched 870 records, retrieved 568, contributes to no pooled estimate. - **Mediator** (3 queries, 2010–2026): muscle strength, muscle mass and sarcopenia against all-cause and cardiovascular mortality, restricted to syntheses reporting a hazard ratio or a dose–response slope. Added at revision to supply the second link of the calculation in Section 4.5; matched 897 records, retrieved 376, contributes to no pooled estimate. - **Randomized** (12 queries, 2015–2026), design-filtered on `randomized controlled trial[pt] OR controlled clinical trial[pt]` plus text words, covering protein in older adults, high-protein renal safety, low-protein diets in CKD, protein in type 2 diabetes, protein and lean mass in obesity, protein dose–response, protein plus resistance training (pooled), protein and cardiovascular endpoints, exercise and hard outcomes in older adults, exercise in clinical populations (pooled), exercise dose (pooled), and multidomain interventions. No language restriction was applied at the query level. No publication-type exclusion filter was applied, so guidelines, consensus statements, umbrella reviews and meta-analyses were retained.

15. URL to search strategy

Supplied as Supplementary Table S1, with the machine-readable search log and the query scripts in File S1.

16. Condition or domain being studied

Healthspan and lifespan in adults; all-cause and cause-specific mortality; sarcopenia, frailty and physical function; incident type 2 diabetes and gestational diabetes; chronic kidney disease progression.

17. Participants / population

Adults (≥18 y), any sex. Prespecified strata: generally healthy adults; older adults (≥60 y); obesity; type 2 diabetes; cardiovascular disease (cardiac, vascular, cerebrovascular); chronic kidney disease including dialysis; pregnancy. Exclusions: non-human studies; pediatric populations.

18. Intervention(s) / exposure(s)

(i) Physical activity — volume, intensity, pattern (MVPA minutes/week, MET-minutes, steps/day, VILPA bouts, resistance-training sessions/week), self-reported or device-measured; structured exercise interventions in trials.

(ii) Dietary protein — total, animal and plant protein, expressed in absolute units where available; protein supplementation and protein-restricted diets in trials.

19. Comparator(s) / control

Lowest exposure category, non-guideline-meeting, zero or usual care, placebo or isocaloric control, as reported by the source. **Contrast definitions were not harmonized across studies**; pooling was restricted to strata sharing a single homogeneous contrast definition.

20. Types of study to be included

Prospective cohort studies; randomized controlled trials; systematic reviews and meta-analyses; umbrella reviews; Cochrane reviews. Cross-sectional studies were admitted only to the descriptive population-stratified table and were never pooled. Excluded: animal and in-vitro studies; studies reporting no effect estimate with a 95% confidence interval.

21. Context

No geographic restriction. Publication window 2015–2026 (core tranche 2023–2026).

22. Main outcome(s)

All-cause mortality. **Measures of effect:** hazard ratio, risk ratio, incidence rate ratio and odds ratio, each pooled **separately**; no conversion between measures.

23. Additional outcome(s)

Cardiovascular and cancer mortality; incident sarcopenia and frailty; physical function (grip strength, gait speed, SPPB, major mobility disability); lean and fat-free mass; incident type 2 diabetes and gestational diabetes; kidney outcomes (eGFR slope, progression to end-stage kidney disease, albuminuria); adverse events in high-protein trials.

24. Data extraction (selection and coding)

Full texts retrieved through the PMC Open Access Web Service and Europe PMC render endpoints (no web-interface scraping). Effect estimates located by a deterministic regular-expression pipeline capturing measure, point estimate, both confidence limits and a ±180-character provenance window; **no language-model**

component participates in extraction. Every estimate admitted to an analysis was subsequently read in its full-text context and adjudicated by at least one author. Screening was performed by a single reviewer (declared deviation), with a duplicate-screened stratified random sample at revision to estimate its reproducibility (§2.9).

25. Risk of bias (quality) assessment

- **RoB 2** (Cochrane) for randomized trials.
- **ROBINS-E** (Risk Of Bias In Non-randomized Studies — of Exposure) for observational exposure studies, with the Newcastle–Ottawa Scale as a cross-check for cohort studies.
- **AMSTAR 2** for included systematic reviews and meta-analyses.
- **GRADE** for certainty of the body of evidence, per outcome and per population. **TRIPOD and PROBAST were considered and are not applicable**: both are instruments for multivariable *prediction-model* studies (development, validation, updating). This review synthesises association and effect estimates, not prediction models, and no included study reports a prediction model as its target of inference. Applying PROBAST here would be a category error, and claiming to have applied it would misdescribe the appraisal actually performed.

26. Strategy for data synthesis

Random-effects models fitted by restricted maximum likelihood (`metafor` 5.0.1, R 4.6). Standard errors derived from reported confidence limits on the log scale. Pooling restricted (a) to a single effect measure and (b) to a single homogeneous contrast definition. Primary cohorts and published meta-analyses held in separate layers to prevent double counting; one estimate per cohort per stratum. Heterogeneity reported as I^2 and τ^2; 95% prediction intervals where $k \geq 3$; Egger's regression where $k \geq 10$; leave-one-out influence analysis. Randomized and observational evidence are synthesized in **parallel layers** and compared, never pooled together.

27. Analysis of subgroups or subsets

Prespecified: generally healthy vs. established chronic disease (physical activity); protein source (total / animal / plant); clinical population (healthy, obesity, T2DM, CVD, CKD/dialysis, pregnancy); age stratum; exposure metric (absolute units vs. percentage of energy).

28. Type and method of review

Rapid review with meta-analysis, reported to PRISMA 2020 and PRISMA-S. Submitted as a **Review**, not a Systematic Review, because the conduct standards

of a systematic review (prospective registration, dual independent screening, multiple databases) were not met.

29. Anticipated or actual start date

2026-08-22.

30. Anticipated completion date

2026-09-30.

31. Country

Hungary.

32. Other registration details

None. Not registered on PROSPERO for the reason in the header.

33. Reference / URL for published protocol

Not applicable — the protocol is not deposited in an external repository; it is Supplementary Document S2.

34. Dissemination plans

Submission to *Nutrients* (MDPI). Full search log, extraction pipeline and analysis code released as Supplementary Material under an open license.

35. Keywords

physical activity; dietary protein; healthspan; all-cause mortality; sarcopenia; dose–response; plant protein; processed meat; nutrition policy; PRISMA.

36. Details of any existing review of the same topic by the same authors

None.

37. Current review status

Completed. Risk-of-bias assessment (RoB 2, ROBINS-E with a Newcastle–Ottawa cross-check, AMSTAR 2) and GRADE certainty assessment are complete and reported in Tables S7–S10; the manuscript is under peer review at *Nutrients*.

38. Additional information

A recent single-author Perspective in *Frontiers in Nutrition* argues the same general direction ("beyond the bare minimum") on narrative grounds. The present review overlaps that paper in framing and **diverges from it in findings**: our quantitative synthesis does not support a uniform recommendation for higher protein intake. The relationship with that paper is stated explicitly in the manuscript rather than left for a reader to discover.

39. Details of final report / publication(s)

To be added.

40. Language

English. # PRISMA 2020 checklist

Reporting is claimed at the **checklist** level. Conduct deviations from a full systematic review are declared in Manuscript §2.8 and are repeated in the "Location / status" column wherever they affect an item. An item marked **DEVIATION** is one where this review does not meet the systematic-review standard and says so in the manuscript rather than claiming compliance.

#	Item	Checklist item	Location / status
1	Title	Identify the report as a systematic review	**DEVIATION — deliberate.** The report is identified as a *Review* reporting a PRISMA-guided rapid review (§2.1). It is not titled a systematic review because it does not meet systematic-review conduct standards.
2	Abstract	PRISMA 2020 for Abstracts checklist	Structured abstract (Background/Objectives, Methods, Results, Conclusions).

#	Item	Checklist item	Location / status
3	Rationale	Rationale in the context of existing knowledge	§1, paragraphs 1–4.
4	Objectives	Explicit statement of objectives / questions	§1, numbered questions 1–5 (question 5 added at revision; protocol field 13).
5	Eligibility criteria	Inclusion and exclusion criteria and grouping	§2.3; grouping for synthesis in §2.5–§2.7.
6	Information sources	All databases, registers, other sources, and dates	§2.2, §2.8. **DEVIATION:** analytic corpus from PubMed only; Europe PMC, Semantic Scholar and ClinicalTrials.gov searched at internal revision to test coverage, not to extract estimates (Table S1b); no Embase, Web of Science, SPORTDiscus, CINAHL or gray literature.
7	Search strategy	Full search strategies for all sources, including filters	§2.2 and Supplementary Table S1 — all 58 PubMed query strings verbatim (the 153 further executions are in Table S1b) with UTC execution timestamps, true match counts and retrieved counts

#	Item	Checklist item	Location / status
			(PRISMA-S appendix).
8	Selection process	How many reviewers screened, independently, tools used	§2.8, §2.9. **DEVIATION:** single reviewer outside a duplicate-screened stratified random sample of 888 records, 889 with the one record admitted by the top-up search after the sample had been drawn (κ = 0.50, PABAK = 0.67).
9	Data collection process	How many reviewers collected data, independently, tools	§2.4. Deterministic regular-expression extraction (no language-model component), then contextual adjudication by at least one author.
10a	Data items — outcomes	All outcomes sought; which results sought from each	§2.3, §2.5. All-cause mortality primary; secondary outcomes listed in protocol field 23.
10b	Data items — other variables	Other variables sought; assumptions about missing data	§2.5, §2.7. Exposure metric, contrast definition, population stratum and effect measure were recorded for every estimate; estimates with missing confidence limits were excluded rather than

#	Item	Checklist item	Location / status
			imputed.
11	Risk of bias	Tools used, how many assessors, independence	§2.9. RoB 2 for randomized trials; ROBINS-E (with Newcastle–Ottawa cross-check) for cohorts; AMSTAR 2 for included reviews. ROBINS-E, RoB 2 and AMSTAR 2 were assessed by two authors independently, disagreements resolved by a third; the Newcastle–Ottawa cross-check was scored at revision by a single rater and informs no rating (Table S7b). **TRIPOD/PROBAST considered and not applicable — no prediction-model studies (§2.9).**
12	Effect measures	Effect measure(s) used for each outcome	§2.5, §2.6. HR, RR, IRR and OR pooled separately; **no conversion between measures**.
13a	Synthesis — eligibility	Processes to decide which studies enter each synthesis	§2.6. Pooling restricted to a single effect measure, a single homogeneous contrast definition, the adjusted

#	Item	Checklist item	Location / status
			whole-cohort estimate, one estimate per cohort, and at least three estimates.
13b	Synthesis — preparation	Data preparation, conversions	§2.6. Standard errors derived from reported confidence limits on the log scale (normal approximation). No cross-measure conversion.
13c	Synthesis — display	Tabulation / visual display methods	§3.2, §3.5, §3.6; Figures 2–3 (forest plots); Tables 1–4.
13d	Synthesis — methods	Methods and model, heterogeneity, software	§2.6. Random effects, REML, `metafor` 5.0.1, R 4.6; Q, τ^2 and I^2 with 95% CIs; 95% prediction intervals; Knapp–Hartung sensitivity analysis (added at revision).
13e	Synthesis — heterogeneity	Subgroup analysis, meta-regression	§2.6, §3.2 (general vs. clinical populations, Q_M test; exploratory meta-regression added at revision, Table S14); §3.6 (population strata, tabulated not pooled).
13f	Synthesis — sensitivity	Sensitivity analyses	§2.6, §3.2. Leave-one-out and influence

#	Item	Checklist item	Location / status
			diagnostics; restriction to studies not at high risk of bias; parent-cohort (NHANES) overlap and Knapp–Hartung sensitivity analyses (added at revision).
14	Reporting bias	Methods to assess risk of bias due to missing results	§2.6, §3.2. Egger's regression where k ≥ 10, with its low power stated; trim-and-fill sensitivity analysis (added at revision). **DEVIATION:** the open-access constraint (§2.8 item 6) is an unquantifiable source of missing results.
15	Certainty assessment	Methods to assess certainty in the body of evidence	§2.9. GRADE, per outcome and per population; summary-of-findings table (Table 5).
16a	Study selection results	Numbers screened, included, excluded, with flow diagram	§3.1 and Figure 1 (PRISMA 2020 flow diagram).
16b	Excluded studies	Studies that appeared to meet criteria but were excluded, with reasons	§3.1 and Supplementary Table S3 (per-estimate exclusion rationale).
17	Study	Characteristics of	Supplementary

#	Item	Checklist item	Location / status
	characteristics	each included study	Tables S3–S5.
18	Risk of bias in studies	Risk-of-bias assessments for each included study	§3.10 and Supplementary Tables S7 (ROBINS-E), S7b (Newcastle–Ottawa), S8 (RoB 2), S9 (AMSTAR 2).
19	Results of individual studies	Effect estimate and precision for each study	Figures 2–3; Tables 1–4; Supplementary Table S2 (all 8,304 extracted estimates with provenance: 6,005 ratio estimates and 2,299 mean differences).
20a	Results of syntheses — summary	Summary of contributing studies per synthesis	§3.2, §3.5, §3.6, §3.9.
20b	Results of syntheses — statistical	Summary estimate, precision, heterogeneity, direction	§3.2, §3.5.
20c	Results of syntheses — heterogeneity	Investigations of causes of heterogeneity	§3.2, §3.5, §4.2 (contrast-definition heterogeneity).
20d	Results of syntheses — sensitivity	Sensitivity analyses and robustness	§3.2 (leave-one-out).
21	Reporting biases	Risk of bias due to missing results	§3.2 (Egger), §2.8 item 6, §4.7.
22	Certainty of evidence	Certainty for each outcome assessed	§3.10 and Table 5 (GRADE summary of findings).

#	Item	Checklist item	Location / status
23a	Discussion — interpretation	General interpretation in the context of other evidence	§4.1–§4.5.
23b	Discussion — limitations of evidence	Limitations of the evidence included	§4.2, §4.3, §4.7.
23c	Discussion — limitations of review	Limitations of the review processes used	§2.8, §4.6, §4.7.
23d	Discussion — implications	Implications for practice, policy and research	§5.1–§5.10.
24a	Registration	Registration information / statement of non-registration	§2.8 item 1. **Not registered, and no registration will be sought.** PROSPERO does not accept reviews whose data extraction is complete, and a retrospective entry would misdate the protocol. A retrospective protocol in PROSPERO field format is provided instead as Supplementary Document S2, with its retrospective status stated in its first line.
24b	Protocol	Where the protocol can be accessed	Supplementary Document S2, supplied with this manuscript.
24c	Protocol amendments	Amendments to the protocol	Not applicable — the protocol is

#	Item	Checklist item	Location / status
			retrospective and describes what was done, not what was planned. Stated as such.
25	Support	Financial and non-financial support, role of funders	Funding statement — none.
26	Competing interests	Competing interests of review authors	Conflicts of Interest statement — none.
27	Availability of data, code, materials	Which are publicly available and where	Data Availability Statement; Supplementary File S1 (extraction pipeline and pooling script), Table S1 (search log).

PRISMA-S checklist (search reporting)

#	Item	Status	Where
1	Database name	Reported	PubMed, for every query, in the machine-readable log.
2	Multi-database searching	Analytic corpus single-database (PubMed); Europe PMC, Semantic Scholar and ClinicalTrials.gov searched for coverage. Declared as a deviation.	§2.2, §2.8 item 3, Table S1b.
3	Study registries	ClinicalTrials.gov searched for coverage at internal revision;	§2.8 item 3, Table S1b.

#	Item	Status	Where
		not used for extraction.	
4	Online resources and browsing	Not used. Full text was retrieved only through the PMC Open Access Web Service and Europe PMC render endpoints; the PMC web interface was not scraped, in compliance with NCBI policy.	§2.4.
5	Citation searching	Performed at internal revision on included references, for coverage.	§2.2, Document S4.
6	Contacts	No authors were contacted.	§2.8.
7	Other methods	None.	—
8	Full search strategies	Reported verbatim for all 58 queries, with UTC timestamps	Supplementary Table S1; the machine-readable log and the query scripts are in File S1.
9	Limits and restrictions	Publication-date limits only (2023–2026 core; 2015–2026 supplementary, population, randomized, incretin and microbiome tranches; 2018–2026 dietary assessment; 2010–2026 physical-activity	§2.2.

#	Item	Status	Where
		expansion and hypertrophy tranches). No language limit. No publication-type exclusion filter. Design filters applied *inside the query string* for the randomized tranche only, and printed verbatim.	
10	Search filters	The randomized tranche used `randomized controlled trial[pt] OR controlled clinical trial[pt]` plus text-word variants; the filter is not a validated published filter and is printed in full.	§2.2, Table S1.
11	Prior work	Search terms were developed by the review team; no published strategy was adapted.	§2.2.
12	Updates	Capped queries re-executed without a retrieval cap and core queries re-run with a 1990 date floor at internal revision.	§2.2, Document S4.
13	Dates of searches	22–23 August 2026, logged to the second in UTC for every query.	Table S1.

#	Item	Status	Where
14	Peer review of the search	**Not peer reviewed (no PRESS appraisal).** Declared.	§2.8.
15	Total records	Reported per query (true PubMed match count and records retrieved) and in aggregate; all twenty-three capped queries are named.	§2.8 item 5, §3.1, Table S1.
16	Deduplication	Cross-query duplicates removed on PMID; the number removed is reported.	§3.1.

Document S3. Methods in full — search architecture, declared deviations and appraisal

The Methods of the manuscript state each decision once and point here for the detail. Nothing in this document contradicts the main text; it is the same material at the length a reader who wants to reproduce or contest the work will need. Section numbers refer to the manuscript.

S3.1 The ten search tranches, one by one

Expands Section 2.2. Every query string, its execution timestamp, its true match count and the number retrieved are in Table S1.

Queries fell into ten tranches. Each is named below with its count, its publication window and the reason it exists. Every query string, its execution timestamp, its true match count and the number retrieved are given verbatim in **Table S1**.

- **Core** (8, 2023–2026) — eight themes: activity dose–response and mortality; activity guidelines and healthy aging; protein and longevity; protein dose and sarcopenia; combined resistance training and protein; sports nutrition; steps

and vigorous intermittent lifestyle physical activity; and protein restriction with mTOR and IGF-1.

- **Supplementary** (4, 2015–2026) — the pivotal pooled analyses of protein and mortality predate 2023, so the window was widened for them.
- **Population** (7, 2015–2026) — healthy adults, obesity, type 2 diabetes (two), cardiovascular disease, chronic kidney disease and an upper-intake safety query. All seven are framed in **absolute intake units** rather than percentage of energy.
- **Randomized** (12, 2015–2026) — each query carried an explicit design filter inside the query string: `randomized controlled trial[pt] OR controlled clinical trial[pt]`, plus text-word variants. Both primary trials and trial-restricted syntheses were therefore retrieved. This tranche exists because the first three were dominated by prospective cohorts and because the divergences in Section 4.3 can only be adjudicated by randomized evidence.
- **Incretin** (6, 2015–2026) — added because the review identified one clinical population whose protein exposure is being altered by prescribing faster than guidance is being written (Section 3.11).
- **Microbiome** (5, 2015–2026) — added because Section 3.5 found that protein *source* carries a signal that protein *amount* does not. The gut is the mechanism most often proposed for it. **Declared a sample, not a search** (Section 2.8 item 5).
- **Dietary assessment** (4, 2018–2026) — added because Section 4.4 identifies the exposure instrument, not the analysis, as the binding constraint on the food-matrix question. **Also a sample.**
- **Physical-activity expansion** (5, 2010–2026) — the window was widened to 2010 because the cohort literature defining the single pooled contrast behind Table 1 predates the core cut-off. A dedicated device-measured query was included because every cohort in that stratum uses self-report, its weakest ROBINS-E domain.
- **Hypertrophy dose–response** (4, 2010–2026) — added at revision because Figure 5 draws a muscle-accrual window. A window on a different endpoint from every other bar in that figure must be sourced from a search of its own.
- **Mediator** (3, 2010–2026) — added at revision to supply the second link of the calculation in Section 4.5. The review had a randomized estimate of what the intervention does to muscle, and none of what muscle does to mortality.

S3.2 Declared deviations from PRISMA 2020, in full

Expands Section 2.8.

We state these plainly rather than burying them:

1. **No prospective protocol registration, and none will be sought.** The review question evolved across the analysis, so a prospective record does not exist, and a retrospective PROSPERO entry would misrepresent when the protocol was fixed. PROSPERO is a register of intentions; registering one after the analysis is complete converts it into a description. We have therefore written the protocol out in full, in PROSPERO field format, with every element dated and every deviation from the original plan marked. It is supplied as **Document S2**. It is a retrospective protocol and is labeled as one. A reader who wants to know what was decided before the data were seen, and what was decided after, can read it there rather than take our word for it.

2. **Single reviewer for screening.** Screening of records and adjudication of extracted estimates were performed by one author (K.S.), and screening outside the validation sample was single-reviewer. At revision a stratified random sample of 888 records was screened in duplicate, together with one record admitted by the top-up search after the sample had been drawn: 889 in all ($\kappa = 0.50$, PABAK = 0.67). This does *not* apply to the risk-of-bias appraisal, which was rated in duplicate by V.G.-O. and C.D., with K.S. resolving disagreements (Section 2.9). The asymmetry follows from the appraisal being added after selection was complete. It means the screening step, not the appraisal, is the one a reader should treat as unreplicated.

3. **Single-database analytic corpus.** Every admitted estimate came from PubMed; Europe PMC, Semantic Scholar and ClinicalTrials.gov were searched at revision to test coverage only. Embase, Web of Science, SPORTDiscus, CINAHL and gray literature were not searched.

4. **Risk of bias was appraised after the fact, not as part of screening.** The instruments in Section 2.9 were applied to the studies that had already entered a synthesis. They did not drive study selection, because selection was complete before the appraisal began. No study was removed on the basis of its rating. Ratings therefore inform the certainty grading in Section 3.10 and the confidence labels in Section 5, and nothing else.

5. **Twenty-three searches were capped, and two tranches are samples rather than searches.** Of fifty-eight queries, thirty-five retrieved every matching record. The remaining twenty-three were truncated by the retrieval cap, and are flagged individually, with their true match counts, in Table S1. The truncation is severe in two places. The **microbiome tranche** is the extreme case: its five queries matched 20,872 records and retrieved 1,250, a relevance-ranked sample of roughly 6% (1,090 of them unique). The **dietary-assessment tranche** is next, at 1,700 matched against 597 retrieved across its four queries, three of which were capped (1,553 matched against 450 retrieved). Everything else is mild. The remaining ten capped queries lost 3,138 records between them. The hypertrophy and mediator tranches were

added at revision; they matched 870 and 897 records, retrieved 568 and 376, and lost a further 823 between their five capped queries. They inform one figure block and one Discussion calculation respectively, and no pooled estimate.

This matters most for Sections 3.11 and 3.12, and we want to be blunt about it. The microbiome and dietary-assessment literatures are enormous, and PubMed's relevance ordering is not random. We cannot characterize the direction of what was missed. Those two sections are therefore written as **orientation over a large sample, not as a synthesis of a field**, and they contribute nothing to any pooled estimate. Section 3.12 is deliberately excluded from the policy recommendations for exactly this reason. The remaining capped queries feed the incretin section, which should be read the same way.

6. **Open-access constraint.** 4,074 of 7,544 records (54.0%) were screened on abstract alone because no open-access full text existed. Estimates reported only in paywalled full texts are therefore systematically under-represented, and the magnitude of this selection pressure cannot be quantified from within the review.
7. **A second extraction defect was found and corrected during this revision.** The mean-difference pattern added for the randomized layer initially allowed the gap before a confidence limit to consume a minus sign. "95% CI: −0.21, 0.89" was therefore read as "0.21 to 0.89", converting a null result into a significant one. It was caught on a single estimate whose published text we happened to be reading (Section 4.6). The pattern was rewritten to forbid any dash in that position, and the extraction was re-run from scratch. Reporting this rather than quietly fixing it is the point: an extraction pipeline that is never shown to have failed has probably not been checked.

S3.3 Risk of bias and certainty of evidence — domains and instrument selection

Expands Section 2.9.

Three instruments were applied, each to the design it was built for, and one pair of widely cited instruments was considered and rejected.

- **Randomized trials — RoB 2** (Cochrane risk-of-bias tool for randomized trials). Its five domains are the randomization process; deviations from intended interventions; missing outcome data; measurement of the outcome; and selection of the reported result. Trials of dietary protein are almost never participant-blinded, so domain 2 was assessed against the

effect of assignment to intervention rather than adherence. The absence of blinding was not by itself treated as high risk where the outcome was objectively measured.

- **Observational exposure studies — ROBINS-E.** Its seven domains are confounding; measurement of the exposure; selection of participants; post-exposure interventions; missing data; measurement of the outcome; and selection of the reported result. The **Newcastle–Ottawa Scale** (cohort form, nine stars) was scored for the same 26 studies as a cross-check, because it remains the instrument most readers of this literature will recognize. Its scoring conventions were fixed before scoring and are printed with the table, since NOS is applied differently by different groups and an unstated convention makes the totals incomparable. The complete star-level grid is Table S7b. Where the two instruments disagree, the ROBINS-E judgment is the one reported. Section 3.10 gives the disagreement rather than burying it: it is substantial, and it is the reason for the choice.
- **Systematic reviews and meta-analyses — AMSTAR 2.** Seven critical domains determine the overall confidence rating: protocol registration, search adequacy, justification of exclusions, risk-of-bias assessment in the included studies, appropriateness of the meta-analytic method, consideration of risk of bias in interpretation, and assessment of publication bias.
- **Certainty of the body of evidence — GRADE**, applied per outcome and, for protein, per clinical population. Observational bodies of evidence start at low certainty and were rated up only for large magnitude of effect or a demonstrated dose–response gradient. Randomized bodies start high and were rated down for risk of bias, inconsistency, indirectness, imprecision and suspected publication bias.

Each study appraised with ROBINS-E, RoB 2 or AMSTAR 2 was rated independently from the published report by two authors (V.G.-O. and C.D.). Disagreements were resolved by discussion with a third (K.S.). Note the asymmetry with Section 2.8 item 2: screening was single-reviewer, the appraisal was not. The Newcastle–Ottawa cross-check was added later, during revision, and was scored by a single rater. It informs no grade and no conclusion, and is reported only for the comparison in Section 3.10. Ratings are the review team's judgment of the published record, not of the underlying data. The completed domain-level grids are supplied in full (Tables S7–S9), with the GRADE summary in Table S10, so that a reader who disagrees with a judgment can see exactly which domain it turns on.

TRIPOD and PROBAST were considered and are not applicable here. Both are instruments for multivariable *prediction-model* studies: TRIPOD for their reporting, PROBAST for their risk of bias and applicability. Both are organized around participants, predictors, outcome and analysis *as they bear on a model that produces individual-level predicted risks*. This review synthesizes association and

effect estimates. No included study reports development, validation or updating of a prediction model, and none of our own analyses produces a predicted risk for an individual. Scoring PROBAST's analysis domain against a Cox model fitted for aetiological inference would not measure anything meaningful, and reporting that we had done so would misdescribe the appraisal actually performed. We record the decision here rather than omitting it silently, because the two instruments are increasingly requested by default and their scope is often misunderstood.

Document S4. Extended methods and results

Material moved here from the main text to keep the manuscript within the length a *Nutrients* Review is normally granted. Each passage appears under the section of the main text it came from, and the main text carries a summary and a pointer at the place it was taken from. Two qualifications apply. Where a passage has been corrected since the submitted version — the setting moderator of Section 3.5 above all — this document carries the corrected text. It also says what the earlier version claimed. And a few passages appear twice: in a long form under the section they came from, and in a short form under the recommendation that uses them. Where the two differ in wording, the long form governs. Numbers quoted in the main text can be traced to their full derivation here.

Citations. Reference numbers below are those of the main manuscript's reference list, not a separate one. Every source cited here is also cited at the corresponding place in the main text, so the two documents share one numbering scheme and one list.

Section 4.5 — combined activity and protein

Protein alone is not uniformly inert, and the exception is population-specific. The conditionality above was established on *strength* in community-dwelling older adults. It does not generalize to every endpoint or every population. On *muscle mass*, protein supplementation without a training co-intervention carries a small but significant effect in community-dwelling older adults: SMD 0.116 (0.032–0.200), 38 articles, I^2 = 15.3%. Neither dose (≥30 versus <30 g/day), frequency nor timing modified it [93]. Distribution tracks function even where no training is prescribed. Preoperative patients reaching 20 g of protein at two or more meals had higher handgrip strength than those who did not [135]. That is the practical expression of the anabolic-resistance argument for raising intake above the adult reference value in old age [126]. The isolated-nutrient version of the strategy remains the weakest of the three. Leucine alone does not improve muscle mass or strength in older adults; its benefit emerges only in combination with resistance training or microbiota-targeted co-intervention [119].

The exception has a hard boundary, and it lies in acute illness. In critically ill adults, delivering enteral protein at the doses international guidelines recommend (1.2–2.0 g/kg/day) rather than below them did not affect mortality across 14 randomized trials and 6,553 patients (RR 1.01, 0.92–1.12, $I^2 = 0\%$). In the subgroup with acute kidney injury it was associated with *higher* mortality (1.29, 1.05–1.58) [61]. A recommendation to raise protein irrespective of loading therefore does not extend to acute illness, and specifically not to acute kidney injury.

Who stands to gain is close to the inverse of who is usually addressed. Section 3.13 sets out the estimates; their joint implication is what matters here. Sarcopenia carries a substantial mortality association: an odds ratio for all-cause mortality of 3.64 (2.94–4.51) in adults aged 60 and over [115]. The penalty is several times larger where organ disease and muscle loss coincide than in healthy or athletic populations. Two details in those estimates deserve emphasis because they are actionable. It is the *combined* phenotypes that carry the risk: osteosarcopenia rather than low bone density or sarcopenia alone in CKD [117], and sarcopenic obesity rather than low muscle mass alone in type 2 diabetes [118]. Screening on a single body-composition measure will therefore miss the group that matters. And the only measure whose *improvement* tracked lower risk in the CKD cohort was handgrip strength, not muscle mass, body mass index or bone density [117]. That points the intervention at function rather than at bulk. Because the outcome penalty is concentrated in the old, the diseased and the malnourished, so is the expected absolute benefit of protecting muscle. Those are precisely the groups from whom protein is most often withheld on organ-protective grounds.

Would the combination extend life, and what would it take to show it? No trial has tested it, and this review does not try to supply the answer by calculation. An earlier version of this section carried a Monte Carlo projection that multiplied the randomized effect of the intervention on muscle by the observational hazard that low muscle mass carries. **We have withdrawn it.** The projection answered only how much benefit would flow through the *muscle-mass pathway alone*, and returned implied hazard ratios near 0.97. That is roughly an order of magnitude smaller than the 20–30% mortality differences observed between active and inactive people in the cohorts of Section 3.2. Those cohorts capture the total effect of activity by every pathway. Set beside those cohorts, it invites precisely the misreading it was built to prevent. That misreading is that the whole prescription is worth about three per cent, at a cost of hundreds of thousands of participants. The qualitative position does not depend on the arithmetic and is not weakened by dropping it. The effect of the combination on function is established and dose-quantified; its effect on mortality is untested. The outcome penalty attached to muscle loss is several times larger in the old, the diseased and the malnourished than in healthy or athletic populations (Section 3.13). A hard-endpoint trial is therefore far more likely to be informative in those strata than in a general older population. Section 5.8 item 4 acts on that conclusion, and now states it without a costing we could not defend.

We should be explicit about the inferential step this licenses, and the one it does not. Section 5.6 gives the incretin recommendation particular weight. A reader is entitled to ask how that weight can rest on an interaction whose mortality effect is untested. It does not rest on it. The recommendation is an argument from **harm avoidance under a known exposure**, not from a demonstrated survival benefit. Lean mass is being lost, at a measured 1.02 kg (0.57–1.46) more than control across 19 trials. The only intervention shown to move that endpoint requires loading alongside the protein. And issuing the protein target alone is what current practice does when it does anything. That showed no effect on the one endpoint where it has been tested (+0.13%, −1.53 to 1.79). The claim is therefore that the *cheaper, better-evidenced half of the intervention is being omitted*, not that adding it will extend life. Nothing in this review shows that it will, and Section 5.6 does not say so.

Section 5.9 — the general adult population

On protein amount, there is nothing to chase — but there is something to avoid, and it is the low end. Total protein intake is null for all-cause mortality in the general population across three independent syntheses. No threshold is detectable in dose–response modeling. Our own five-cohort pool is null too, and splitting it by setting does not separate it. The healthy cohorts give 0.93 (0.55–1.57) against 0.83 (0.44–1.56) in chronic disease, Q_M p = 0.79 (Section 3.5). The one cohort in the stratum not rated at high risk of bias, the Rotterdam Study, reports higher mortality at higher intake, 1.12 (1.01–1.25). Nothing here supports raising intake in this group. That null is an average over an asymmetric curve: across the populations of Table 7, higher intake is the better direction in nine and the worse direction in two. For a healthy adult the practical consequence is not to raise intake. It is to stop worrying about the upper half of the adequacy range and to make sure of the lower half. For a healthy adult under 65 the defensible statement is a wide adequacy range: 0.8–1.6 g/kg of actual body weight per day. Within it, no position has been shown to be better than another. The widely quoted 1.6–2.2 g/kg/day is not a range of increasing benefit. It is a hypertrophy break-point estimate carried to the upper bound of its confidence interval, above which the lean-mass curve is flat (Section 5.5). Intakes in that range have not been shown to harm kidney function in people with normal kidneys, but “not harmful” is not “better”.

2. **Unprocessed red meat is not the target, but it is not unconditional either.** The evidence associates it with higher all-cause mortality at the population level (HR 1.12, 1.04–1.20) [121]. That is a small effect relative to the processed fraction and relative to what accompanies it, and it does not apply uniformly. Three groups should treat it differently. The first is people with established cardiovascular disease, where habitual intake at or above 1.8 g/kg/day of total protein was associated with all-cause mortality HR 1.39 (1.17–1.65) in one disputed cohort [56]. The second is pregnancy, where the direction of the protein–outcome relationship inverts (Section 3.8). The third

is anyone in whom a red-meat-dominant *pattern* has replaced a mixed one, since the protein–diabetes association reverses sign between a legumes-and-seafood pattern (OR 0.58, 0.33–0.96) and a red-meat pattern [68].

3. **What it is eaten with can matter more than the meat.** This is the least-appreciated finding in the corpus and the one with the most direct kitchen consequence. A pattern combining baked or fried food with white meat was associated with gestational diabetes at aOR 4.40 (1.58–12.22) relative to a prudent pattern [122]. That is several times larger than any single protein-source estimate in this review. In 1,190 adults aged 65–100, a 5% increase in protein from meat carried an odds ratio for diabetes of 1.34 (1.02–1.76) while protein from vegetables and cereals carried none [70]. The operational advice is therefore about the plate, not the portion. Unprocessed meat, cooked without deep-frying, should be eaten with vegetables, legumes and whole grains rather than with refined starch fried in fat.

"Plant-based" is not self-justifying, and the split is measurable. In 200,727 US adults followed for 4.1 million person-years, a *healthful* plant-based index was inversely associated with type 2 diabetes after adjustment for body mass index (HR 0.66, 0.61–0.72). An *unhealthful* one — built on refined grains, potatoes, sweetened beverages and desserts, all of which are plant foods — was positively associated (1.16, 1.08–1.25) [69]. Advice to "eat more plants" that does not distinguish the two is capable of moving risk in the wrong direction.

One thing worth doing before 65 that is usually left until after it. The muscle a person carries into old age is the substrate every recommendation in Section 5.5 operates on. The outcome penalty attached to losing it is large (Section 3.13). Strength training in middle age is the cheapest available way of raising that starting point. Unlike the protein question, it has a demonstrated effect on function at every age tested. We state it here rather than in the older-adult section because by the time the older-adult section applies, the opportunity is smaller.

Section 3.9 — the randomized layer

Randomized exercise evidence on all-cause mortality is imprecise. Across three independent syntheses in clinical populations the point estimate sits between 0.83 and 0.93, and every confidence interval crosses the null. Exercise-based cardiac rehabilitation in coronary heart disease gives RR **0.87 (0.73–1.04)** at 6–12 months, **0.90 (0.80–1.02)** at medium term and **0.91 (0.75–1.10)** at long term [78]. Heart failure gives RR **0.93 (0.71–1.21)** [79] and, on individual participant data, HR **0.83 (0.67–1.04)** [80]. A fourth synthesis is the exception, and we give it its due rather than filing it under imprecision. A network meta-analysis of 19,411 participants restricted to center-based cardiac rehabilitation returns RR **0.76 (0.64–0.90)** [81], the only randomized all-cause-mortality estimate in this domain that excludes 1. It is also the weakest of the four as a general claim. It rests partly on indirect

comparisons between program formats, and speaks to one delivery model in one disease. In generally healthy older adults, the single long trial designed around mortality is Generation 100: five years, 1,567 participants aged 70–77. It returned HR **0.63 (0.33–1.20)** for high-intensity training against control, with an absolute risk difference of 1.7 percentage points that did not reach significance [77].

Protein supplementation increased strength with resistance training but not without it, and the effect scaled with the dose added above habitual intake. Protein supplementation against a lower-protein control did not change total lean body mass in older adults (WMD **0.34 kg, −0.21 to 0.89**, $I^2 = 90\%$) [82]. A 2025 synthesis stratified by activity level found the same (MD **−0.01 kg, −3.23 to 3.20**, $I^2 = 0\%$) [85]. In interventions that also used resistance exercise, the same review found appendicular lean mass WMD **0.54 kg (0.03–1.05)** and handgrip strength WMD **1.71 kg (0.12–3.30)**. The lean-mass effect was confined to sarcopenic or frail participants (WMD **0.88, 0.51–1.25**) [82]. A dose–response meta-analysis of 69 articles makes the interaction explicit. Muscle strength increased with added protein **only** when resistance training was present (MD **+2.01%, 1.09–2.93**) and not without it (MD **+0.13%, −1.53 to 1.79**). Within the trained groups, added protein above 0.5 g/kg/day produced **+4.29% (1.99–6.60)** against **+1.22% (0.41–2.03)** below that threshold, a 3.5-fold difference [83]. A recent triple-crossover trial delivering 0.6 g/kg/day of supplemental protein on a 1.0 g/kg/day background during high-intensity functional training sits inside that window [125].

High-protein diets do not damage normal kidneys over trial horizons. Twenty-two randomized trials of adults without chronic kidney disease were appraised by their authors with RoB 2 and GRADE. High-protein diets raised eGFR (SMD **0.39, 0.09–0.69**) and left serum creatinine unchanged (SMD **0.08, −0.09 to 0.25**, $I^2 = 21\%$) [86]. A rise in filtration with no rise in creatinine is the signature of hemodynamic adaptation, not of injury, and it is the opposite of what the renal-harm argument predicts. The trials are short. That is a real limitation, and the reason the certainty is graded Moderate rather than High (Table 5). The trials available nonetheless do not support the renal-harm claim in people without kidney disease.

In established kidney disease, the effects of protein restriction differ by endpoint. Restriction reduced kidney failure (OR **0.59, 0.41–0.85**) and end-stage renal disease (OR **0.64, 0.43–0.96**). It did not reduce all-cause death (OR **1.17, 0.67–2.06**) [87]. Cochrane found death RR **0.77 (0.51–1.18)** and end-stage kidney disease RR **1.05 (0.73–1.53)** for low versus normal protein. For very-low versus low protein, end-stage kidney disease fell (RR **0.65, 0.49–0.85**) while death did not (RR **1.26, 0.62–2.54**) [88]. A six-year trial of a supplemented very-low-protein diet in advanced chronic kidney disease found mortality HR **0.95 (0.62–1.45)** and renal death HR **1.17 (0.88–1.57)** [89]. In diabetic nephropathy, restriction improved proteinuria (SMD **0.69, 0.22–1.16**) and did nothing to glycated hemoglobin (SMD **0.17, −0.18 to 0.51**) [90].

Training composition. Adding strength work to aerobic training improved cardiorespiratory fitness in coronary artery disease (SMD **0.26, 0.02–0.49**, and **0.36, 0.05–0.68** where aerobic volume was preserved) [91]. Resistance training raised handgrip strength in sarcopenia by **2.69 kg (1.78–3.61)** across 42 trials [92]. The floor for a strengthening prescription is a randomized quantity, not an extrapolation.

Section 5 — reading Figure 5

The reference value is one number. Panel (a) contains a single red line and eleven bars. That is the disagreement in its entirety. The 0.83 figure is not wrong. It is a *safe level*, derived from nitrogen balance, designed to prevent deficiency in 97.5% of a population. It has nonetheless come to be read as a target, and it is applied unchanged to a 25-year-old, an 85-year-old, a patient on dialysis and a patient losing 15% of body weight on an incretin agonist. Nine of the eleven windows we propose have a lower bound at or above it and an upper bound well above it. Only the non-dialysis chronic kidney disease row sits substantially against it, and there the randomized evidence shows an effect on kidney endpoints but not on mortality (Section 3.9).

The denominator column is not decoration. Three rows use ideal or adjusted body weight rather than actual weight. A guideline that omits this is not merely imprecise. At a BMI of 35 the same instruction produces intakes differing by roughly 40%, and expressing intake per kilogram of actual weight is what manufactures the spurious "high protein is harmful" findings dissected in Section 4.2.

Panel (b) shows a threshold facing a distribution. The WHO aerobic recommendation is a band from 150 to 300 min/week. The evidence in Section 3.3 is that the curve has already bent well before 150, hence a floor at 10 min/week and a gradient running to roughly 500. Above that, the returns flatten. For muscle strengthening, WHO states a frequency (≥2 days per week) and no volume at all. We propose 60–120 min/week *with the ceiling published*, because the absence of an upper bound is itself a barrier for people who assume strength training means a gym membership.

The green block is a different endpoint, and it converges on the same number. The eleven upper bars answer a question about survival, function and disease progression. The three lower bars answer a question about muscle, and we have drawn them apart rather than together. Pooling a mortality contrast with a lean-mass contrast is precisely the error Section 4.1 objects to elsewhere. Read on their own terms, they are quantitatively well supported. Across 49 randomized trials in 1,863 participants, protein supplementation added to resistance training increased one-repetition-maximum strength by **2.49 kg (0.64–4.33)** and fat-free mass by **0.30 kg (0.09–0.52)**. The break-point was **1.62 g/kg/day (1.03–2.20)**, beyond which no further fat-free-mass gain was observed [12]. A three-level synthesis of 74 trials reproduces both the effect and its age structure. Lean body mass gave **SMD 0.22**

(0.14–0.30) where resistance exercise was present, significant at **1.2–1.59 g/kg/day** in those aged ≥ 65 and only at **≥ 1.6 g/kg/day** in those younger [112]. Intakes above the recommended allowance rather than at it add **0.32 kg (0.01–0.64)** of lean mass across 22 comparisons [136]. What determines the size of the effect is not the absolute intake but the *increment*. In trained adults, supplementation adding more than 0.5 g/kg/day above habitual intake improved strength by **4.29% (1.99–6.60)**, against **1.22% (0.41–2.03)** for smaller increments [83].

Three cautions travel with that block, and the figure states them rather than hiding them. First, the effects are real but small in absolute terms: a third of a kilogram of fat-free mass, two and a half kilograms on a one-repetition maximum. No trial has shown that optimizing them extends life, so the endpoint separation is not a formality. Second, the athlete bar is not a range of increasing benefit but the break-point estimate carried out to the upper limit of its own confidence interval. The plateau lies somewhere between 1.03 and 2.20 g/kg/day and is best estimated at 1.62. The range 1.6–2.2 therefore marks where the plateau plausibly sits, not a dose that outperforms 1.6. It is consistent with the International Society of Sports Nutrition position stand of 1.4–2.0 g/kg/day [129]. That is reassuring for practice, but a consensus document cannot be counted as independent confirmation. The honest reading of everything above roughly 1.6 g/kg/day is *not demonstrated to be harmful*, not *demonstrated to be better*. Third, and shown as an explicit null row, the whole block is conditional: without resistance training, protein changes strength by **0.13% (−1.53 to 1.79)** [83]. The bars are a dose. They are not, on their own, an intervention.

Section 3.5 — protein amount and source

What survives is not uniformly reassuring, and one stratum is worse than the pooled result suggests. For plant and animal protein the surviving estimates point the same way as the pools they came from: plant protein HR 0.65 (0.45–0.93) in the SUN cohort, animal protein 0.94 (0.70–1.26) in the same cohort. The *direction* of the plant-protein finding therefore does not depend on the high-risk studies. Its *precision* does, and with it our entitlement to call it a pooled estimate. **Total protein is the exception, and it runs against us.** Its one surviving cohort is the Rotterdam Study, rated Some concerns and described in our own appraisal as the best-characterized protein cohort in the set. It reports **HR 1.12 (1.01–1.25)** for highest versus lowest total protein: a statistically significant association with *higher* mortality, where the five-cohort pool returns a null of 0.91 (0.64–1.27). We report this rather than leaving it in the supplement, because it is the single result in this review most likely to be turned against our own framing. The honest reading is uncomfortable in a specific way. The general-population null for total protein, which we use to argue that amount alone does not determine risk, rests on a stratum whose only well-measured cohort does not find a null. It does not overturn the null: one cohort, an FFQ exposure, and a confidence interval whose lower bound sits at 1.01. A reader is nonetheless entitled to know that the pooled null and the low-risk-

of-bias evidence are not saying the same thing, and Table 2 now shows both side by side. We report the pooled values as the primary result because they were pre-specified. We state here, rather than in the supplement, that the protein pooling layer of this review would not survive a restriction to low-risk exposure measurement. That is the single strongest argument in this paper for the measurement recommendation of Section 5.8.

Section 5.5 — the population windows

A note on the muscle target, which this table deliberately does not contain. Readers will come to Table 6 looking for the number that maximizes muscle, and they will not find it here. Every row above is graded against mortality, function or disease progression. That number exists and is well estimated: a break-point at 1.62 g/kg/day (1.03–2.20) across 49 trials [12], reproduced with an age split at 1.2–1.59 in those ≥ 65 and ≥ 1.6 in those younger [112]. It is drawn as a separate block in Figure 5 for exactly that reason. Two consequences follow for guideline writers. The upper bound this review proposes for most populations, 1.6 g/kg/day, turns out to coincide with an independently measured hypertrophy break-point. That is a convergence worth stating rather than a coincidence worth hiding. And the widely quoted athletic range of roughly 1.6–2.2 g/kg/day is not, on this evidence, a range of increasing benefit. It is the break-point estimate carried out to the upper bound of its confidence interval, above which the lean-mass curve is flat. The defensible claim is that such intakes are not demonstrably harmful, not that they are better.

Section 2.2 — the search update at revision

Search update at revision. Three changes were made after the first draft and are reported here rather than folded silently into the counts above. First, every capped query was re-executed with no retrieval cap, so the 24,686 records that PubMed matched but did not return are now retrieved. Second, the nine core-tranche queries, which had been bounded at 2023, were re-executed with a 1990 floor. This applied to physical activity the same reasoning that had already widened the supplementary tranche for protein. It recovered two pooled analyses that no query in any database had previously returned [10], [17], both now reported in Sections 3.3, 5.1 and 5.2. The date bound, not the choice of database, was what had excluded them: the same core queries return both once the floor is removed. Third, the search was extended beyond PubMed to **Europe PMC** and **Semantic Scholar**, to **ClinicalTrials.gov** as a trial register, and to **backward and forward citation chasing** on every included reference. Europe PMC returned 3,656 records in total, 3,405 of them seen in no other source. Screening of the enlarged corpus is by two independent reviewers with a third adjudicating (Section 2.9). It is stratified so that the stratum in which inclusion decisions are actually made is dual-screened at the fraction the Cochrane rapid-review standard specifies. Two further strata are sampled to measure the false-negative rate of each automated gate.

Section 3.10 — the appraisal-instrument disagreement

The Newcastle–Ottawa cross-check disagrees with ROBINS-E, and the disagreement is the useful part. Scored on the same 26 studies, NOS returns a median of 7.5 stars out of 9 (IQR 6.0–8.2). Eighteen studies fall in the Good band, five Fair and three Poor (Table S7b). Read alone, that is a reassuring picture of a sound literature. It is not the picture ROBINS-E gives, and the two do not merely fail to agree: in this corpus they point mildly in *opposite* directions. Studies rated High risk of bias average **7.54 stars**, against **6.62** for those rated Some concerns. **Ten of the 13 High-risk studies fall in the NOS Good band.** The rank correlation between NOS total and ROBINS-E severity is **ρ = +0.30**, where agreement would require it to be negative. The cause is structural. The High ratings here are driven by selective reporting (D7) and by confounding by indication or reverse causation (D1), and **NOS has no item for either**. What it does reward — a representative sampling frame, an interviewer-administered instrument, registry death ascertainment, near-complete follow-up — is the profile of the NHANES analyses that account for most of those ratings. NOS totals are reported for comparability with the existing literature and carry **no weight in any certainty grade or conclusion here**. The generalizable lesson, and the scoring conventions behind it, are set out with Table S7b.

Remarkably, three independent meta-analyses by three different author groups report the identical point estimate for plant protein and all-cause mortality: **0.92** (BMJ 2020, [47]; NMCD 2020, [48]; *Nutrients* 2024, [46]). The two numbers are not in conflict, and the difference is worth stating precisely because it is a contrast artifact rather than a disagreement. The published syntheses pool graded or per-increment exposures across dozens of cohorts, so their estimate is the average effect of a small shift in plant-protein intake. Ours admits only extreme-quantile contrasts, Q4 or Q5 versus Q1 in four cohorts. That separates the exposure far more widely, and therefore returns a larger effect per comparison rather than a stronger effect per gram. Our estimate should be read as the size of the difference between the top and bottom of the intake distribution in four cohorts. The 0.92 figure is the one to use if a per-increment number is wanted, and with the Women's Health Initiative included the two are close rather than discrepant.

The single most-cited primary analysis in this area reports on the per-increment scale and belongs beside them. In 131,342 participants of the Nurses' Health Study and the Health Professionals Follow-up Study, plant protein carried **HR 0.90 (0.86–0.95) per 3% of energy**. Animal protein was not associated with all-cause mortality (1.02, 0.98–1.05 per 10% of energy) [50]. One qualification matters and is discussed in Section 4.4. Both associations were confined to participants carrying at least one of smoking, heavy alcohol use, overweight or inactivity, and were not evident in those carrying none. Neither is evidence that plant protein *amount* drives the association, since the top quantile of plant protein also marks a different whole diet (Section 4.4).

For the **older-adult window**, the randomized evidence attaches a condition that the table alone does not convey. On strength, and on appendicular lean mass, protein at these intakes does nothing measurable unless resistance training accompanies it [82], [83], [85]. The qualification is endpoint-specific and should be stated as such, because protein alone is not wholly without effect. It raises total muscle mass by a small margin in community-dwelling older adults, SMD 0.116 (0.032–0.200), independently of dose, frequency and timing [93]. Two rules follow rather than one. **A protein target for older adults should always be issued with a loading prescription attached**, because that is where the effect on strength and function lies. **But it should not be withheld where loading cannot be delivered.** In frailty, in disease-related malnutrition, in the immobilized and the institutionalized, protein remains the deliverable half of the intervention. Declining to prescribe it on the grounds that the trials also included training is not what those trials showed. The one setting in which that permission does not extend is acute illness with acute kidney injury, where higher protein delivery was associated with increased mortality (Section 4.5) [61].

For the **kidney rows**, randomized trials consistently move renal endpoints and consistently fail to move mortality [87], [88], [89]. Restriction can therefore be offered as a way of delaying dialysis, which is a real and patient-relevant goal. It must not be presented as a way of living longer, and in patients over 75 or with any sign of protein-energy wasting the balance tips against it. The clinical literature in nephrology has begun to make the same argument from the other direction, against one-size-fits-all management of the diabetic–sarcopenic CKD patient [134]. Physical activity is also associated with lower mortality in end-stage kidney disease [143], which strengthens the case for pairing any dietary prescription in this group with an activity one. Three further considerations bear on these rows. The mechanism invoked for restriction — raised intraglomerular pressure and hyperfiltration — is an argument for individualization rather than for a uniform ceiling [133]. Source matters within the restricted range, with soy protein in place of animal protein improving renal parameters in CKD on meta-analysis [144]. And restriction must be released once the kidney recovers. In transplant recipients, the change in skeletal muscle mass index over the first post-transplant year tracked protein intake after adjustment ($p = 0.015$); the intake predicted to hold muscle at its pre-transplant value was estimated at 0.72 g/kg ideal body weight per day [63]. In maintenance hemodialysis, ten-year survivors had higher protein (0.91 ± 0.21 versus 0.82 ± 0.24 g/kg) and energy intake than decedents. Only energy intake below 25 kcal/kg (HR 1.86, 1.02–3.40) and the malnutrition–inflammation score above 5 (HR 2.15, 1.17–3.93) were independent predictors of all-cause mortality [64]. That negative detail is worth reporting, because it locates the modifiable risk in total energy and inflammation rather than in protein quantity alone.

Section 2.2 — the search tranches

Queries fell into **ten tranches**, grouped by tranche in Table S1 and described one by one, with the reason each exists, in Document S3. Four were planned from the outset. The **core** tranche (8 queries, 2023–2026) covers activity dose–response, protein and longevity, sarcopenia and protein restriction. The **supplementary** tranche (4, 2015–2026) was widened because the pivotal pooled analyses of protein and mortality predate 2023. The **population** tranche (7, 2015–2026) is framed throughout in **absolute intake units** rather than percentage of energy. The **randomized** tranche (12, 2015–2026) carries an explicit design filter inside each query string; it was added because the first three were dominated by prospective cohorts, and because the divergences in Section 4.3 can only be adjudicated by randomized evidence. Six were added as the analysis raised questions the planned searches could not answer. These are **incretin** (6, 2015–2026), **microbiome** (5, 2015–2026), **dietary assessment** (4, 2018–2026), **physical-activity expansion** (5, 2010–2026), **hypertrophy dose–response** (4, 2010–2026) and **mediator** (3, 2010–2026). The last two were added at revision, to source the muscle-accrual window proposed in Section 5.5 and the second link of the calculation in Section 4.5 respectively. The microbiome and dietary-assessment tranches are declared samples rather than searches (Section 2.8 item 5).

Section 2.9 — screening agreement

Raw agreement was **p_o = 0.834**, Cohen's **κ = 0.50 (95% CI 0.42–0.57)**, and **PABAK = 0.67**. We report PABAK alongside κ deliberately. With 83% agreement and a heavily skewed marginal distribution — most records are rejections — κ is depressed by the prevalence paradox rather than by poor agreement, and quoting κ alone would misdescribe the result. Agreement was stable across strata (κ 0.45, 0.52 and 0.53) and under a stricter mapping of the four options to include/exclude (0.50). The conclusion therefore does not turn on the mapping convention. The **149 discrepancies** were adjudicated by K.S., blind to neither reviewer's identity, yielding **38 inclusions and 111 rejections**. The adjudicator agreed with V.G.-O. on 89 and with C.D. on 60, so the adjudication did not simply defer to one reviewer. Three of the 38 were then excluded on the formal grounds declared in Section 2.3: one congress abstract and two preprints. They are shown as such in Figure 1.

Section 3.13 — the sarcopenia penalty

The penalty carried by the sarcopenic state, and how much it depends on the definition. How large the association looks depends on how completely muscle loss is defined. The three available estimates should be read as three different questions rather than as one dose–response curve, because they use different phenotypes, different effect measures and different populations. **Low muscle mass alone**, against normal, carries all-cause mortality **RR 1.36 (1.28–1.44)** across 49 prospective studies, 878,349 participants and 61,055 deaths [114]. Requiring

function as well as *mass* gives a much larger figure. In adults aged 60 and over, sarcopenia was associated with all-cause mortality at **OR 3.64 (2.94–4.51)** and with functional decline at **OR 2.58 (1.33–4.99)**, estimated under an inverse-heterogeneity model across 12 and 6 prospective studies (14,169 and 8,561 participants). Influence analysis leaving out each study once left both significant [115]. **The two are not directly comparable.** An odds ratio exceeds the corresponding risk ratio when the outcome is common, and death over long follow-up is common. The contrast between 1.36 and 3.64 should therefore be read as a difference in what was measured, not as a quantified effect of adding a functional criterion. In type 2 diabetes, sarcopenia carried **HR 1.70 (1.21–2.40)** across 21 cohorts and 580,277 participants [116], again on a different measure and in a different population.

The penalty is larger where organ disease and muscle loss coincide. The study followed 251 outpatients aged 65 and over with CKD not on kidney replacement therapy, for a median 5.2 years. **Osteosarcopenia** — sarcopenia together with low bone mineral density — carried **HR 3.28 (1.52–7.08)** for the composite of death, kidney replacement therapy and major adverse cardiovascular and cerebrovascular events, and **HR 2.07 (1.10–3.89)** for the kidney composite. Neither low bone density alone nor sarcopenia alone reached that risk. Low handgrip strength was the strongest single component (**HR 2.44, 1.46–4.08**). Rising handgrip strength was also the only body-composition measure associated with lower risk (**HR 0.93, 0.89–0.98**), where body mass index, skeletal muscle mass index and bone density were not [117]. A further 3,123 patients with type 2 diabetes and preserved renal function were followed for 8.9 years. **Sarcopenic obesity**, and not low muscle mass alone, predicted incident CKD (**adjusted HR 1.77, 1.24–2.51**; 530 incident cases) [118].

Section 4.4 — the food matrix

First, **isocaloric substitution analyses are the most interpretable design available**, because they hold total energy constant and specify what replaces what. Substituting 3% of energy from plant protein for **processed meat** protein was associated with all-cause mortality HR **0.54 (0.38–0.75)** and cancer mortality HR **0.50 (0.30–0.85)**. The corresponding substitution for **unprocessed red meat** gave HR **0.66 (0.55–0.80)** [124], corroborated by [145]. Even these, however, are silent on cooking method and on what else is on the plate. A 2026 meta-analysis of nine cohorts, 1,036,799 participants and 19.9 million person-years now pools the substitution question directly. Replacing 3% or 5% of total caloric value from animal protein with plant protein was associated with all-cause mortality **HR 0.91 (0.87–0.94)**, with no difference between the two substitution levels. Cardiovascular mortality was 0.88 (0.83–0.93) at 3% and 0.77 (0.70–0.86) at 5% [123]. That is a smaller effect than the single-cohort substitutions above, which is what one expects when the contrast is defined by energy replacement rather than by quantile extremes. The same body of evidence carries a warning against reading it as a general prescription. In the largest primary analysis, the plant-protein association

was **confined to participants with at least one unhealthy lifestyle factor**, and was not detectable in those with none [50]. If that effect modification is real, the observed association with substitution is concentrated in exactly the population that dietary advice reaches least well. A population-average estimate then understates it in one group and overstates it in another.

A contrary reading of the same territory should be acknowledged. A narrative review of the Mediterranean and Okinawan patterns argues that a **protein-to-carbohydrate ratio well below 1** is itself part of what slows aging, delivered through whole grains and plant foods [146]. That claim is neither tested nor refuted by anything in our corpus. Our finding that total protein is null with respect to mortality is compatible with a macronutrient *ratio* mattering, because a ratio is not an amount. It does however cut against any recommendation framed as "more protein for everyone", from whichever direction.

Section 3.11 — incretin therapy

The gap, and what makes it visible. A cross-sectional study followed 69 adults on a GLP-1 RA for at least a month, with three-day food records. Mean protein intake was **77.3 g/day (95% CI 70–84)**, against a calculated requirement of **74–169 g/day** at 1.2–2.0 g/kg [102]. The finding we want to draw attention to is not the shortfall itself but *how it was hidden*. **Protein as a percentage of total energy was within the acceptable macronutrient distribution range.** The same intake is adequate on the relative metric and deficient on the absolute one, in the same people, on the same day. This is the denominator problem of Section 1 and Section 4.2, occurring prospectively in a population of millions. It is the clearest single argument in this review for reporting protein in grams per kilogram rather than as a share of energy.

Anabolic resistance. One retrospective analysis examined a randomized trial of home-based resistance exercise plus protein-based multi-ingredient supplementation in older males. **Obesity and metabolic-syndrome risk were the strongest negative predictors of the gain in lean mass** (r = −0.36 to −0.68), while age, kidney function and activity level were only weakly associated [104]. The population most exposed to incretin-associated lean-mass loss is the population in which protein-plus-loading works least well. That is an argument for the upper end of the range and for early initiation, not for abandoning the intervention.

Section 5.8 — the measurement agenda

6. **Adopt image-based, AI-assisted dietary assessment as the exposure instrument for the next generation of protein cohorts — and fund the validation that would make it credible.** This is the most consequential methodological recommendation we can make. It follows directly from Section 4.4. The instruments in use carry roughly 30% error against doubly labeled water: the 24-hour recall under-reports energy by about 16%, the food-frequency questionnaire by 26–32% [137], [138]. They are being used to

argue about hazard ratios of 1.1. But the deeper problem is what the instrument is built to see. A questionnaire asks how often red meat was eaten and never asks what was on the rest of the plate, although Section 4.4 shows that the accompaniment may carry more of the signal than the meat. Image-based assessment records the plate rather than the food name. It is now good enough to be worth validating and not yet good enough to deploy uncritically: reported errors run from 0.10–38.3% for calories, with a 20% residual under-report against doubly labeled water. The finding that matters is that **accuracy is worst on exactly the mixed plates that carry the exposure of interest** [139], [140], [141], [142]. That is an argument for funding the validation, not for waiting. Funders should require five things in new cohorts. First, whole-eating-occasion image capture. Second, validation against weighed records **and** doubly labeled water, reported **per meal-complexity stratum** rather than pooled, since the pooled figure hides the failure mode. Third, protein reported in grams and g/kg alongside percentage of energy. Fourth, retention of the images or a derived plate-composition vector. Fifth, prespecified handling of algorithmic error as measurement error. The evidence behind each figure, and the five requirements in full, are Table S12.

7. **A combined activity-and-protein trial against hard endpoints — in a high-risk stratum, and nowhere else.** The synergy is established on function, and dose-quantified above 0.5 g/kg/day of added protein [83]. What is missing is any trial carrying it through to mortality or disability-free survival. Where such a trial should be sited is the recommendation. The mortality and morbidity penalty attached to muscle loss is several times larger in high-risk strata than in the general older population (Section 3.13). The same design that would be hopelessly underpowered in community-dwelling older adults is therefore on the scale of a cardiovascular outcome trial in sarcopenic obesity, type 2 diabetes or incretin therapy. Funding this as a general-population question is therefore unlikely to be efficient. The tractable version is a hard-endpoint trial of protein plus supervised loading, added to incretin therapy, powered on all-cause mortality or disability-free survival, in a population already enrolled in weight-management services. It should be designed now, while the exposure is being created at scale, rather than after the prescribing wave has passed. A trial powered instead on physical function would answer a smaller question at a tenth of the size and is the fallback if the mortality version is unfundable.

The red-flag checklist in full

Red flags in a protein recommendation

1. **A protein number with no denominator.** “1.2 g/kg/day” is not a specification until it says whether the kilograms are actual, ideal or adjusted

body weight. In obesity, heart failure and dialysis the choice changes the prescribed amount by tens of grams a day, and silence on it generates the spurious harm signals of Section 4.2.

2. **A single floor applied across populations.** The direction of the protein–outcome relationship reverses across the populations of Table 3. Any document carrying one number for all of them is wrong for most of them.

3. **A protein target issued without a loading prescription.** On strength and appendicular lean mass, protein at these intakes does nothing measurable without resistance training: +0.13% (−1.53 to 1.79) without training, against +2.01% (1.09–2.93) with it [83].

4. **The converse: protein withheld because loading is not feasible.** In frailty, disease-related malnutrition, immobility and institutional care, protein remains the deliverable half of the intervention. It has a small independent effect on muscle mass (SMD 0.116, Section 3.13). "The trials included exercise" is not a reason to prescribe nothing.

5. **Restriction in the old or the wasting.** Above roughly 75 years, or with any sign of protein-energy wasting, age should override organ status. In CKD over 65, moderate versus low intake was associated with all-cause mortality HR 0.14 (0.02–0.69) [59].

6. **Chronic-disease reasoning carried into acute illness.** In critical illness, protein at 1.2–2.0 g/kg/day versus below did not move mortality (RR 1.01, 14 randomized trials). In acute kidney injury it was associated with *higher* mortality (1.29, 1.05–1.58) [61]. This is the one place in the review where more protein was associated with harm in randomized data.

7. **Athletic ranges presented as a benefit gradient.** 1.6–2.2 g/kg/day is the upper reach of a break-point confidence interval, not a dose–response curve (Section 5.5).

8. **Longevity or healthspan claims for protein supplements resting on biomarkers or mechanism.** Nothing in this corpus supports them on human outcome data. One cohort in cardiovascular-risk populations points the other way.

Red flags in an activity recommendation

9. **Threshold framing.** "150 minutes per week" stated as a pass/fail target misrepresents a curve whose steepest segment lies below it. It also discourages precisely the inactive people who would gain most (Section 5.1).

10. **Strength work treated as an optional addition to aerobic activity.** The two guidelines are separately and additively associated with survival. Meeting

the strength guideline alone gave HR 0.79 (0.66–0.94), against 0.84 (0.78–0.90) for aerobic alone [17].

11. **A strength recommendation with no ceiling.** No additional reduction in mortality is observed above 120 min/week. Publishing “2 days per week” without saying so leaves an open-ended demand in front of the least-adherent groups [131].

Red flags in the evidence being cited

12. **Sarcopenia screened on a single body-composition measure.** The risk was carried by osteosarcopenia rather than sarcopenia or low bone density alone, and by sarcopenic obesity rather than low muscle mass alone (Section 3.13). Single-measure screening misses the group the recommendation is for.

13. **“Plant-based” used without a healthful/unhealthful split** (HR 0.66 versus 1.16 in the same cohorts) [69].

14. **“Eat less red meat” offered where a processing-targeted measure is available.** The processing-targeted version is better supported and less vulnerable to food-matrix confounding (Sections 4.4, 5.7).

15. **A cohort estimate of protein intake presented without its denominator problem**, or an observational muscle–mortality association presented as if it were causal. Section 4.2 sets out the first. Section 3.13 states the reverse-causation problem attaching to the second, and Section 4.5 explains why we declined to convert it into a projected survival benefit.

16. **An effect on muscle or on function presented as an effect on survival.** Every randomized estimate in this review has a body-composition or functional endpoint; none has mortality. A recommendation that slides from “improves strength” to “extends life” has crossed the one gap this review could not close, and Section 4.5 says so explicitly. Table 8 applies this test to our own recommendations, one row at a time.

Section 1 — the competing Perspective in full

The case for revising both numbers upward has already been made in narrative form. A 2026 Perspective in *Frontiers in Nutrition* argues, under the heading “beyond the bare minimum”, that national physical activity and protein guidelines are anchored to deficiency prevention rather than to functional capacity. It concludes that both should be raised [16]. We agree with that framing and adopt it as our starting point. We depart from it in three respects, and the departures are the reason for this paper. First, that argument is narrative. No search was logged, no estimates were pooled and no risk of bias was appraised, so a reader cannot tell how much of the evidence

base was seen. Second, it expresses protein intake per unit of body weight without confronting what that denominator does to studies in which body size is itself on the causal path. Third, and most consequentially, it treats “more protein” as a single recommendation for a broad population. Our synthesis does not support that: once intake is expressed in absolute units and the population is named, the direction of the protein–outcome association is not constant. The same Perspective introduces the useful notion of the “protein wrapper” — the nutritional package a protein is delivered in — but leaves it qualitative. We quantify it, and it turns out to carry more of the signal than protein quantity does.

Section 3.2 — the activity stratum

Seven of the sixteen were added at revision. The original core search covered 2023–2026 only, and the cohort literature that defines this contrast is older; a dedicated 2010–2026 tranche was therefore run against this stratum specifically (Section 2.2). Every added estimate was read in full text and admitted only on the adjusted, whole-cohort figure. In one case (COPD) the adjusted HR of 0.53 was taken and the paper’s more striking unadjusted 0.35 discarded.

Heterogeneity. I^2 moved from 34% at k = 9 to 64% (95% CI 29–89%) at k = 16. It does not concern direction: every one of the sixteen point estimates lies between 0.53 and 0.86, and all sixteen confidence intervals exclude unity. Part of it reflects one very precise estimate. Removing the single largest cohort — 47,281 deaths, confidence interval 0.67–0.71 — lowers I^2 from 64% to 39% and moves the pooled estimate to 0.770. The rest is unexplained: none of exposure definition, region, publication year, the standard error of the estimate or length of follow-up accounted for it in exploratory meta-regression (Table S14). The prediction interval, 0.65–0.87, describes the range within which the association in a new comparable cohort would be expected to fall.

Section 3.3 — the dose–response curve

The largest estimate of this curve is a pooled analysis of six prospective cohorts, **661,137 adults** over a median 14.2 years [10]. Against no leisure-time activity, adults doing **less than the recommended minimum** already had **HR 0.80 (0.78–0.82)**. One to two times the minimum gave **0.69 (0.67–0.70)** and two to three times **0.63 (0.62–0.65)**. The curve reached its floor at **three to five times the recommendation** (**HR 0.61, 0.59–0.62**), with no excess risk at ten times or more (HR 0.69, 0.59–0.78). Being a published synthesis, it is reported beside our pooled cohort estimate and not inside it (Section 2.5).

It also carries a qualification this review states rather than absorbs. The authors’ own reading is that meeting the guideline minimum captures *nearly* all of the observed reduction in mortality: the marginal gain from the minimum to the optimum is 31% against 39%. The case made in Section 5.1 is therefore an

argument about where the observed reduction in risk **begins**, and about the cost of a pass/fail line. It is not a claim that the 150-minute figure is set at the wrong level.

A caution against over-reading the floor. In the largest single cohort ([32], n = 109,407), the lowest non-zero activity quartile (0–75 min/week) showed **no** reduction in mortality versus complete inactivity (HR 1.08, 0.98–1.19). The 75–360 min/week band did (HR 0.89, 0.81–0.98). A trivial dose is not a proportionally small reduction in risk; in that cohort no reduction was observed. Any "start with ten minutes" message must therefore be framed as an entry point on a gradient, not as a terminus.

Section 2.8 — the rapid-review standard

The standard these deviations are declared against. PRISMA 2020 is a reporting guideline, not a conduct standard, and this review was conducted as a **rapid review**. The conduct standard that applies to it is therefore the Cochrane Rapid Reviews Methods Group guidance [29], updated for reviews of effectiveness in 2024 [30]. Naming it is what turns the items below from omissions into declared method choices. That guidance makes four relevant recommendations; the numbering below is its own. It limits the main database search to CENTRAL, MEDLINE (for example via PubMed) and Embase where access allows (R10). It asks for dual screening of **at least 20%** of titles and abstracts with conflict resolution, and single-reviewer screening of the remainder (R14). It asks for single-reviewer data extraction with a second reviewer checking correctness and completeness (R16). And it asks for single-reviewer risk-of-bias rating with full verification by a second (R19). **Table S13** maps this review against those recommendations one by one. It records three places where the review falls short: CENTRAL and Embase were not searched, no second reviewer screened the excluded records at title or full text, and extraction was not independently checked by a second reviewer. It records two where the review goes beyond the guidance: risk of bias was rated in full duplicate rather than rated once and verified, and corpus coverage was tested against three further sources rather than left untested.

Section 5.2 — the muscle-strengthening dose

Two further findings bear on how this recommendation should be written. An umbrella review of 41 systematic reviews covered roughly 4,475 unique primary studies. It found moderate-to-high-certainty evidence that resistance exercise combined with protein supplementation at ≥1.2 g/kg/day outperforms either alone on muscle mass (SMD 0.22) and handgrip strength (MD 4.19 kg). It also found high-certainty evidence that balance and multicomponent exercise reduce fall rates by 23–34% (rate ratios 0.66–0.77) [113]. Falls are the endpoint that converts the strength recommendation from a physiological one into a clinical one, given the observational sarcopenia–falls association (OR 1.60–1.89) reported in the same synthesis. And the recommendation is, at present, largely unmet in exactly the

groups it would most benefit. Among 333,507 respondents to the 2011 Behavioral Risk Factor Surveillance System aged over 45, only 23.7% met the DHHS muscle-strengthening recommendation. The lowest odds were among those with less than a high-school education (OR 0.32, 0.30–0.35), a body mass index of 30 kg/m^2 or more (0.47, 0.45–0.49), and age 85 or over (0.63, 0.58–0.68) [131]. A ceiling that removes the gym-scale assumption is therefore not a minor editorial point; it addresses the specific barrier reported by the least-adherent groups.

Section 3.7 — type 2 diabetes

Three further datasets sharpen the source argument and one qualifies it. Three US cohorts totaled 200,727 participants and 16,162 incident cases over 4,102,369 person-years. A *healthful* plant-based index was inversely associated with T2D after adjustment for body mass index (HR 0.66, 0.61–0.72, extreme deciles), while an *unhealthful* plant-based index was positively associated (1.16, 1.08–1.25) [69]. "Plant" is therefore not the operative variable, and should not be written into guidance as though it were. In 1,190 adults aged 65–100 in the Mediterranean Islands study, a 5% increase in protein from meat carried an odds ratio for diabetes of 1.34 (1.02–1.76), and a 5% increase in total protein 1.29 (1.00–1.69). There was no association for protein from vegetables or cereals [70]. The signal is detectable even at the level of single amino acids. Higher habitual L-arginine intake was associated with T2DM incidence (HR 2.71, 1.20–6.09, >5.4 versus 2.69 g/day), while total protein intake and the arginine-to-protein ratio were not [71]. The result is hypothesis-generating rather than actionable; we report it because it illustrates how much of the apparent "amount" effect may be compositional. The qualification concerns dairy, the one animal source with a favorable epidemiological signal. The clinical evidence for dairy proteins in type 2 diabetes is confined to acute studies of insulin secretion, with no long-term trial reporting prevention, management or treatment endpoints [72]. The yogurt association above should not yet be converted into a dairy-protein recommendation.

Section 4.3 — the four divergences

4. **Physical activity and all-cause mortality — the divergence that runs the other way.** Here the observational literature is the *more* certain of the two. Cohorts give a precise pooled HR of 0.76 (0.72–0.79) across sixteen studies. Three randomized syntheses give 0.83–0.93, with every interval crossing the null [78], [79], [80], and a fourth, restricted to center-based cardiac rehabilitation, gives 0.76 (0.64–0.90) [81]. The single long trial powered on mortality in healthy older adults is also imprecise, HR 0.63 (0.33–1.20) [77]. The estimates agree in sign and are statistically compatible; what differs is precision, and the reason is structural rather than substantive. Exercise trials cannot blind, cannot prevent control participants from exercising, and cannot run for the decades over which the observational gradient

accumulates. Generation 100 is the clearest illustration: five years, 1,567 participants, and a control group that exercised anyway [77].

Section 4.7 — limitations in full

One limitation is specific to the recommendations rather than to the evidence. The synergy between protein and loading is established on muscle mass, strength and physical function, and on nothing beyond that. No trial in this corpus carries the combination through to mortality or disability-free survival, and the observational layer cannot supply the interaction because no cohort reports it. Sections 5.5 and 5.6 nonetheless make forceful recommendations that invoke the combination. They are framed as harm avoidance under a documented exposure rather than as survival claims (Section 4.5), and each states its own endpoint. The distance between a demonstrated function effect and a mortality endpoint is nonetheless real, and a reader should hold us to it. Section 4.5 states what would be required to close that gap, and Section 5.8 item 4 sets out where such a trial should be sited. Neither supplies a projected effect, for the reason given under Section 4.5 above. The distance between a demonstrated function effect and a mortality endpoint is real and a reader should hold us to it. This limitation is specific to the recommendations rather than to the evidence, and it is the reason each of them names its own endpoint. Formerly this paragraph ended with a costed design. That calculation multiplies a randomized link by an observational one, and its second factor carries exactly the reverse-causation problem this review criticizes elsewhere. It is reported with a causal-fraction sensitivity for that reason, and it belongs to the Discussion rather than the Results. It must not be read as evidence that the combination extends life.

Section 3.5 — reading across populations

Read across populations rather than pooled across them, the direction is not null but asymmetric: in most populations higher intake is better than lower. This review assembles sixteen populations in absolute intake units. They are set out with the recommendations in Section 5.5, because that is where the windows they support are proposed. **Nine** carry a net direction of *more rather than less*: healthy adults ≥65, the very old, obesity and sarcopenic obesity, CKD over 65, diabetic kidney disease, kidney transplant recipients, cardiac inpatients and heart failure, adults on incretin therapy, and disease-related malnutrition. Only **two** run the other way — community cardiovascular disease at habitual intakes ≥1.8 g/kg/day, and the acute phase of critical illness. Two more are bounded windows in which both tails carry risk (maintenance hemodialysis, non-dialysis CKD 3–4), and in three the operative variable is source or dietary pattern rather than amount. The general-population null is therefore not a statement that intake does not matter. It is an average taken over a population in which most people sit near adequacy. It averages a deficiency tail that is steep and common with an excess tail that is

shallow and rare. **Where a person's position on that curve is unknown, the base rate favors too little rather than too much.**

Section 3.5 — the risk-of-bias sensitivity analysis

A risk-of-bias sensitivity analysis, and the limitation it exposes. The ROBINS-E appraisal (Section 3.10) rated thirteen of twenty-six observational studies at High risk overall. We therefore re-ran every pooled stratum restricted to studies **not** rated High. The activity stratum is robust: **HR 0.762 (0.716–0.811), k = 9**, against 0.756 (0.720–0.794) with all sixteen. **The protein strata are not.** All three fall to a single study and cannot be pooled under our own three-study minimum. Four of the five total-protein cohorts are rated High, as are two of the plant-protein cohorts and one of the two animal-protein cohorts. **One qualification belongs with that sentence rather than in a supplement.** The Women's Health Initiative was added to the plant-protein stratum by the top-up search at revision, after the duplicate risk-of-bias appraisal had been completed. It has therefore not been rated. It is the largest cohort in the stratum, it is carried in the pooled estimate, and it is carried as *unrated* here. The appraised plant-protein set remains the original three, of which two are High. We state this rather than silently reporting the sensitivity analysis over four cohorts, and it is logged in Document S1. It does not change the conclusion of this paragraph, since the surviving non-High estimate still points the same way. It does mean that the RoB-restricted plant-protein analysis is an analysis of three cohorts, not of the four that produce the headline estimate. In every case the reason is the same ROBINS-E domain, **D2, measurement of the exposure**. The instruments are food-frequency questionnaires that record frequency rather than quantity, or a single 24-hour recall standing in for habitual intake.

Section 3.5 — the setting moderator

What this contrast looked like before the population codings were corrected, and what it looks like now. In the version of this analysis carried in the submitted manuscript, the Rotterdam Study held **83.1% of the weight** in the generally healthy subgroup. The moderator test returned $Q_M = 12.45$, $p = 4 \times 10^{-4}$, and removing Rotterdam alone destroyed it (Q_M 0.92, $p = 0.34$). That version rested on two population codings that the revision audit found to be wrong (Document S1). The KoGES cohort is restricted to eGFR < 60 and belongs on the chronic-disease side; the NHANES estimate comes from the eGFR ≥ 90 group and belongs on the generally healthy side. A third change followed from the same audit. The estimate admitted from the NHANES analysis is reported only for an eGFR stratum and only as a cut-point contrast (≥1.2 versus <0.6 g/kg/day), and Section 2.6 admits neither a subgroup estimate nor a contrast other than highest-versus-lowest quantile. It was therefore removed from the stratum altogether, and is retained in the population table alone. With the recoding and that removal, the stratum is **k = 5**, pooling to **HR 0.91 (0.64–1.27), I^2 = 81.0%**, with a 95% prediction interval of 0.43–1.89. Rotterdam carries **26.7%** of the weight, and the two sides of the setting split are **0.93 (0.55–1.57)**

and **0.83 (0.44–1.56)**. **The moderator test returns Q_M = 0.07, p = 0.79**: no difference between settings. The chronic-disease side remains **k = 2, below the three-study minimum we impose on pooled results**, and is reported as a subgroup description rather than a pooled estimate by our own rule. Neither subgroup contains more than one cohort not rated High risk of bias, and the chronic-disease subgroup contains none.

Section 3.10 — the appraisal in full

Thirteen of the twenty-six observational studies were rated High overall and thirteen Some concerns; none was rated Low. The reasons cluster into three recognizable failures rather than being scattered. **Selective reporting (ROBINS-E domain D7)** drives two of the High ratings. In [20] the activity estimate is a secondary component of a mediation analysis whose actual exposure is biological-age acceleration; in [21] it is one element of a composite health score. Neither set out to estimate what we used it for. Two further cohorts raise the same concern without it determining the overall rating. In [39], rated Some concerns overall, the estimate is one of four components of a lifestyle score. In [22], rated High on exposure measurement, several activity domains are reported against several models, of which we use one. **Exposure measurement (D2)** drives six of the High ratings, four of them among protein cohorts. There, intake is a single baseline 24-hour recall carried forward over as much as seventeen years [96], [97]; or it is estimated from consumption *frequency* rather than quantity [95]; or it is a single baseline dietary measure in an older cohort (the Kawasaki Aging and Wellbeing cohort, PMID 37558986, cited in Tables S3, S4 and S7 but in no numbered reference of the main text). **Confounding by indication and reverse causation (D1)** account for the rest. In the CKD cohort [94], protein restriction is prescribed to those with worse disease, so the exposure is partly an index of severity. In the stroke cohort [40], post-stroke disability determines both activity and death. The same problem affects three cohorts added at revision: heart failure [38], depression [23] and the twelve months after a COPD admission [19]. The last of these produces the largest effect in Table 1 and is the least trustworthy estimate in it. Activity recorded in routine care before an index admission is in large part a measure of how ill the patient already was.

Section 4.6 — the pipeline defects in full

Our pipeline. (i) The `pdftotext` utility was invoked in a form that failed silently in our environment, returning exit code 0 with empty output. All 825 PDFs in the first harvest therefore fell back to abstract-only text without raising an error. After correction the extracted set grew from 638 to 2,566 estimates. We report this because a silent tool failure that degrades data without raising an error is a foreseeable hazard of automated extraction pipelines. Reviews that use them should validate against a known-good sample. (ii) The mean-difference pattern written for the randomized layer allowed the character gap preceding a confidence limit to absorb a minus sign. A published “95% CI: −0.21, 0.89” was therefore

captured as 0.21 to 0.89, turning a null result into a significant one, and in the wrong direction for our argument. It surfaced on a single estimate whose source sentence we were reading for another purpose [82]. The pattern was rewritten to exclude every dash character from that position, and a six-case regression check was added. The extraction was then re-run from scratch, taking the difference-measure set from 841 to 1,634 estimates with the signs correct. Both were found by validating output against sentences read by hand, not by any internal check the pipeline performs on itself. We assume there are others we have not found.

Section 6 — the conclusions in full

For physical activity, the evidence supports a floor set far lower than 150 min/week, combined with an explicit gradient and a stated ceiling for resistance training. It also supports treating activity prescription in chronic disease as clinical care rather than lifestyle advice, because the association with lower mortality is present there too. Whether it is as large as in primary prevention is uncertain. For protein, no single population-wide number survives contact with absolute-intake data. The optimum is a window; the window differs by population; and in at least one population both tails carry higher risk. That conclusion is drawn from population-stratified evidence read across studies, not from a large pooled estimate. The protein strata pool four to six cohorts each, and none survives restriction to studies not rated high risk on exposure measurement. The total-protein stratum illustrates the problem rather than solving it. Its pooled null separates into opposite directions in health and in disease, under the same moderator that returns nothing for physical activity. That separation rests on a single cohort and does not survive its removal. We therefore report it as an explanation worth testing, and not as evidence anything should be built on. It is the direction, read population by population, that carries this part of the review; the pooled magnitudes do not, and we have not written them as though they did.

On movement, the number to remember is the first step, not the target. The steepest mortality gradient is between doing nothing and doing a little. It is largely complete before the guideline threshold is reached (Section 5.1). Two additions carry most of the remaining gain. The first is 60–120 minutes per week of muscle-strengthening activity, with no further reduction in mortality observed above 120 minutes (Section 5.2). The second is counting incidental and intermittent activity — stairs, carrying, brisk walking in bouts of any length — toward the total (Section 5.3). Adherence, not intensity, is the binding constraint. Only 23.7% of US adults over 45 met the muscle-strengthening recommendation, with the lowest odds among those with less than a high-school education (OR 0.32) and a body mass index of 30 or more (0.47) [131].

Section 5.6 — the incretin recommendation

The recommendation is issued on the strength of the first three rungs and is framed accordingly. It says not to issue, on its own, the component without a demonstrated effect on strength, to a population documented to be losing the tissue the intervention protects. It does **not** say that the combination extends life, and no reader should take the word “urgent” to mean that it does. The urgency comes from the rate at which the exposure is being created by prescribing, not from the size of a demonstrated survival benefit. Guidance issued now should be explicitly provisional and revisited when the function trial reports.

Section 3.5 — the plant-protein re-estimation

Plant protein: an inverse association of very low certainty. A top-up search at revision, run specifically on plant-versus-animal protein contrasts, identified a cohort that met our contrast definition exactly and that the original search had missed. The **Women’s Health Initiative** covers 102,521 postmenopausal women and 25,976 deaths over 1,876,205 person-years, giving plant protein **HR 0.91 (0.86–0.96)** for the highest versus lowest quintile [49]. It was screened on title and abstract by V.G.-O. and C.D. independently, and included by both. It is the one record outside the stratified random sample that enters the duplicate-screening set of Section 2.9, which is why that set is 889 rather than 888. It is by a wide margin the largest cohort in this stratum, and adding it changes the result materially. The pooled estimate across **four** cohorts is **HR 0.76 (95% CI 0.63–0.93), k = 4, I^2 = 65.1%, $p = 5.7 \times 10^{-3}$**. Its 95% prediction interval is **0.54–1.09** (Table 2, Figure 3). Our earlier three-cohort estimate — 0.69 (0.58–0.82) with I^2 = 0.0% — is superseded. **We report the change rather than the corrected value alone, because the homogeneity was the more persuasive part of the original claim and it did not survive.** The direction is unchanged and remains significant. The apparent consistency was an artifact of which cohorts we had.

The total-protein null does not separate by setting, and an earlier version of this analysis said that it did. The protocol pre-specified a generally-healthy-versus-established-chronic-disease contrast for physical activity, and named clinical population as a subgroup dimension. Applying the same moderator to the total-protein stratum at revision is a symmetric extension of that, and we label it as such rather than as pre-specified. When the split was first run it appeared to separate the stratum sharply, with the generally healthy cohorts above unity and the chronic-disease cohorts below it. During the revision audit we re-read the source populations of every cohort in the stratum, and found two coding errors. The KoGES cohort [94] is restricted to participants with eGFR < 60, that is chronic kidney disease stages 3–5, and had been coded as generally healthy. The estimate admitted from the NHANES analysis by eGFR group [97] had been coded as clinical, although it comes from the eGFR ≥ 90 group. Re-reading that source settled the second case differently. It reports estimates only within eGFR strata and only as

cut-point contrasts (≥1.2 versus <0.6 g/kg/day), and Section 2.6 admits neither a subgroup estimate nor a contrast other than highest-versus-lowest quantile. The estimate was therefore removed from the pooled stratum, and is retained in the population table alone. With the KoGES cohort recoded and that estimate removed, the split gives **HR 0.93 (0.55–1.57), I^2 = 87.9%**, in the three generally healthy cohorts and **HR 0.83 (0.44–1.56), I^2 = 76.9%**, in the two chronic-disease cohorts. The moderator test gives **Q_M = 0.07, df = 1, p = 0.79**: no difference at all. The pooled estimate over all five is **HR 0.91 (0.64–1.27)**. We report the correction rather than the corrected numbers alone, because the earlier version of this paragraph carried an argument that the data do not support. The total-protein null is not a mixture of two opposite associations, and the heterogeneity of this stratum is not explained by setting. It is worth setting this beside the activity result, where the same moderator also returns nothing (Q_M = 1.71, p = 0.19, Section 3.2).

What this analysis is, and what it is not. It is an **exploratory** analysis that failed to explain the heterogeneity of the stratum. It is reported because it was run, and because an earlier version of this document drew a conclusion from it. It is **not** load-bearing evidence, and the recommendation of Section 5.5 does not rest on it. Delete this paragraph and the preceding one entirely, and nothing in Section 5.5 changes. The population-specific windows are built from the per-population estimates of Tables 3 and 7, each in its own absolute units with its own denominator. They rest on the randomized evidence against protein restriction in diabetic kidney disease, the mortality association with restriction in chronic kidney disease over 65, the bounded window in maintenance hemodialysis where both tails carry risk, the acute-illness signal, and the incretin population. None of these passes through the total-protein pool or its subgroups. A subgroup analysis of six cohorts cannot carry a guideline change, and this one is not being asked to.

Section 5.5 — what the windows stand on

What this recommendation stands on, in order. Its load-bearing evidence is randomized where randomized evidence exists, and per-population where it does not. It comprises the randomized trials against protein restriction in diabetic kidney disease; the mortality association with restriction in chronic kidney disease over 65; the maintenance-hemodialysis window, the only one in the table whose two bounds come from a common reference; the acute-illness signal, including higher mortality in acute kidney injury; and the incretin population, where lean-mass loss is measured in nineteen randomized trials. The setting moderator of Section 3.5 is **not** on that list. It is exploratory, it shows no difference between settings once the population codings are corrected, and we have deliberately built nothing on it. If a reader deletes it, every row of Table 6 stands where it stood. The *bounds* of each window are magnitudes. They come from the population-stratified estimates of Tables 3 and 7, each read in its own units with its own denominator, and not from Table 2. What this review cannot offer is a precise pooled magnitude for protein and

mortality as a single quantity. The argument of Section 4.2 is that no such quantity exists to be estimated.

Section 5.5 — the trade-off table

The trade-off has to be stated explicitly, because a window on its own does not show it. A reader given only the range in Table 6 cannot see what is being traded against what. In several populations the two directions of error carry very different penalties. The principle the table enforces is the one that the kidney literature illustrates most sharply. In CKD, sarcopenia predicts mortality and CKD progression, protein deficiency produces sarcopenia, and protein excess accelerates filtration loss. Neither "restrict" nor "supplement" is therefore a complete instruction, and both costs must be stated at every level. Empty cells are not reassurance; they mark directions in which this corpus contains no estimate.

Section 6 — the combination

Where this leaves the combination. Movement and protein delivered together, at the levels this review specifies, are among the most promising non-pharmacological candidates in the healthspan literature. Four things recommend them. The two exposures act on the same tissue. Their interaction is established and dose-quantified on function. The outcome they protect carries one of the largest observational penalties in the corpus. And the intervention is cheap, scalable and free of the safety questions that attach to pharmacological longevity candidates. **It is a candidate and not a demonstrated longevity intervention.** That distinction is the whole of Section 4.5. Not one randomized estimate in this review carries a mortality endpoint, and we declined to close that gap by calculation. It is also applied recommendation by recommendation in Table 8. No number in Section 5 can therefore be lifted out of the paper without the endpoint it was measured on. What would close it is larger and longer trials with hard endpoints. They should be sited where the outcome penalty is concentrated rather than where recruitment is easiest, and designed to test the combination against its components rather than either alone. The field should also stop testing the two in isolation from everything else that moves the same pathway. Sleep, incretin prescribing, the processed fraction of the diet and the gut-microbial route of Section 3.12 all plausibly modify the same signal. A trial that holds them uncontrolled will answer a smaller question than the one worth asking.

Section 4.5 — the function evidence

On function, the interaction is established and quantified. Added protein raises muscle strength only in the presence of resistance training (MD +2.01%, 1.09–2.93, with training; +0.13%, −1.53 to 1.79, without). Within trained groups the effect scales with dose: +4.29% (1.99–6.60) when more than 0.5 g/kg/day is added, against +1.22% (0.41–2.03) below that [83]. The same conditionality appears on

body composition. There is no effect of protein on lean mass overall, a significant effect on appendicular lean mass when resistance exercise is co-prescribed, and the largest effect in sarcopenic and frail participants [82]. Trials continue to populate this window. One recent example is a triple-crossover design delivering 0.6 g/kg/day of supplemental protein on a 1.0 g/kg/day background during high-intensity functional training [125]. A synthesis stratified explicitly by activity level confirms the null in the low-activity stratum [85].

What this does not establish is a survival benefit. No trial has tested protein, with or without loading, against mortality in any of these strata. The association between sarcopenia and death is observational, and carries the reverse-causation problem this review criticizes elsewhere. The claim licensed here is narrower and, we think, more useful. The populations in which the muscle pathway carries the largest outcome penalty are not the healthy adults and athletes in whom protein requirements are usually debated. A mortality trial is therefore worth mounting where the penalty is largest rather than where the literature is densest — the design conclusion Section 5.8 item 4 acts on.

The three numbers in full

1. Steps, counted outside work: aim past ~3,000 a day, and keep going. An inverse association becomes detectable in the pooled step literature at approximately **3,143 steps/day**. Each additional 1,000 steps carries **HR 0.91 (0.87–0.95)** [42]. Above 75 years the optimal cut-point falls to about **1,824 steps/day**, and below 75 to about **4,128** [43]. The 10,000-step figure is not supported as a threshold by this evidence, and its practical effect is to make the target look unreachable to the people furthest below it. Two qualifications belong with the number. The gradient does not stop at the threshold — each further 1,000 steps carries its own 9% — so this is a floor, not a target. And the exposure that carries the strongest cohort evidence is **leisure-time** activity: the 661,137-adult pooled analysis measured leisure-time activity specifically [10], and the step estimates come from device-measured daily totals. **Whether the same association holds for steps accumulated at work was not addressed by this corpus and should not be assumed.** We state the target as non-occupational because that is what the evidence measured, not because occupational activity has been shown not to count.

Section 3.13 — what the exposures do

What the exposures do to that outcome. Protein supplementation raises muscle mass in community-dwelling older adults **without** a training co-intervention: **SMD 0.116 (0.032–0.200)**, 38 articles, I^2 = 15.3%, with no significant subgroup difference by dose (≥30 versus <30 g/day), frequency or timing. Data in hospitalized and institutionalized populations were too sparse to pool [93]. Combined with resistance exercise the effect is roughly twice as large and extends to strength. An

umbrella review of 41 systematic reviews reported **SMD 0.22** for muscle mass and **MD 4.19 kg** for handgrip strength at protein intakes ≥1.2 g/kg/day with exercise, at moderate-to-high certainty. The same synthesis reported high-certainty evidence that balance and multicomponent exercise cut fall rates by **23–34%** (rate ratios 0.66–0.77), against an observational sarcopenia–falls association of **OR 1.60–1.89** [113]. Where intake falls short, the association runs the other way. In 118 outpatients with disease-related malnutrition, intake below 70% of calculated requirement carried **OR 4.27 (1.30–14.03)** for sarcopenia and **OR 5.17 (1.63–16.42)** for severe malnutrition [62].

Two qualifications belong here rather than in a later caveat. First, the same meta-analysis that reports the absolute loss also reports that **lean mass as a *percentage* of body mass did not differ** between users and controls [9]. The tissue is lost roughly in proportion, which is what happens in any substantial weight loss. Second, none of these trials measured function. Kilograms of lean mass are not sarcopenia. Sarcopenia is defined by strength and performance, and on those endpoints the evidence in this population is, in the words of the scoping review, "limited and inconsistent" [100].

Section 2.1 — article type and reporting standard

This manuscript is submitted as a Review, not as a Systematic Review, and the distinction is deliberate. MDPI requires systematic reviews to conform to PRISMA and to include a completed checklist and flow diagram. It also strongly encourages prospective protocol registration. Our synthesis satisfies the *reporting architecture* of PRISMA 2020: the search was logged at execution time, identification and screening counts are reported in full, and the flow is auditable. It does **not** satisfy the *conduct* standards of a systematic review. There was no prospective protocol registration. Screening was single-reviewer outside a stratified duplicate-screened sample of 889 records (Section 2.9). The analytic corpus from which every estimate was extracted is a single database, with three further sources used to test its coverage (Section 2.8 item 3). And risk of bias was appraised after selection rather than as part of screening. Labelling the work a systematic review would claim a methodological standard it does not meet. We describe it in the abstract as a PRISMA-guided **rapid review**, and as a rapid review here as well, within the Review article type: the same object under a plain-language name and a methodological one. Every deviation is declared in Section 2.8 rather than implying compliance we did not achieve, and our own confidence is graded accordingly in Section 5. The design was chosen because the review question spans two exposures, several outcome families and multiple clinical populations. A conventional single-question systematic review is poorly suited to that scope.

Section 2.9 — the appraisal instruments

Each design was appraised with the instrument built for it. **RoB 2** was used for randomized trials, across its five domains, and **ROBINS-E** for observational exposure studies, across its seven. **AMSTAR 2** was used for systematic reviews and meta-analyses, with the seven critical domains determining the overall rating. **GRADE** was used for the certainty of each body of evidence, applied per outcome and, for protein, per clinical population. Observational bodies start at low certainty and were rated up only for a large magnitude of effect or a demonstrated dose–response gradient. Randomized bodies start high and were rated down for risk of bias, inconsistency, indirectness, imprecision and suspected publication bias. Two decisions that shape the ratings are worth stating here rather than in the appendix. Protein trials are almost never participant-blinded, so RoB 2 domain 2 was assessed against the *effect of assignment* rather than adherence. Absent blinding was not by itself treated as high risk where the outcome was objectively measured. The **Newcastle–Ottawa Scale** was scored for the same 26 observational studies by a single rater as a cross-check, with conventions fixed before scoring and printed with Table S7b. Where the two disagree, the ROBINS-E judgment is reported, and Section 3.10 gives the disagreement rather than burying it.

Section 2.9 — the screening validation sample

Reproducibility of the screening step. Because screening was otherwise single-reviewer (Section 2.8 item 2), a stratified random sample of **888 records** was screened independently on title and abstract by V.G.-O. and C.D. One record was added to it: the cohort identified by the top-up search at revision (Section 3.5), screened by both reviewers after the sample was drawn and included by both. That gives **889 screened in duplicate**. The sample was drawn after a title-level exposure filter had been applied to the revision corpus. The agreement statistics below therefore describe screening decisions on records that already name one of the review's exposures in their title. They would not transfer unchanged to an unfiltered corpus, where the rejection rate would be higher and chance agreement with it. Each record was rated against a four-option decision (include, reject, re-review, more data needed), and both reviewers completed all 889. One reviewer (V.G.-O.) read the sample offline and transcribed the decisions afterwards, which is why the recorded timestamps cluster.

Section 2.2 — the PubMed search

PubMed was searched via the NCBI E-utilities API. Fifty-eight queries were executed on 22 and 23 August 2026, and are reproduced verbatim in **Table S1**. At revision the search was extended in two directions, both logged at execution time and reported in full in **Table S1b**. Nine of the queries were re-run with their date floor removed (the `-1990` variants, for the reason given below), giving **67 distinct query strings**. All 67 were then run against **Europe PMC** and **Semantic Scholar**, with ten further

queries against **ClinicalTrials.gov** as a trial register. Across the two tables the record therefore covers 211 logged executions against four sources: 67 in PubMed, 67 in Europe PMC, 67 in Semantic Scholar and 10 in ClinicalTrials.gov. That is what PRISMA-S requires, and what the first version of this manuscript reported only for PubMed. Each query string was passed to `esearch` unmodified, with no narrative-review filter and no publication-type exclusion. Guidelines, consensus statements, umbrella reviews and meta-analyses — the substantive backbone of an update — were therefore retained rather than filtered out.

Section 4.2 — the denominator problem in full

When protein is expressed per kilogram of **actual** body weight, the exposure and several outcomes share body size. [120] (TwinsUK) reported that high intake (>1.3 g/kg/day) was associated with low muscle mass (OR 1.76, 1.39–2.24) and sarcopenia (OR 2.04, 1.21–3.44), while low intake was associated with lower odds (OR 0.52, 0.40–0.67). The paper's own baseline table shows the group with low muscle mass weighed **60.2 kg versus 74.5 kg** (BMI 22.9 versus 28.1). They consumed **statistically indistinguishable energy** (1,744 versus 1,770 kcal/day, p = 0.23). Protein was expressed per kilogram of actual body weight; low muscle mass was defined as appendicular lean mass divided by height squared. Body size therefore sits in the denominator of the exposure and in the definition of the outcome. At equal absolute intake, a lighter person is mechanically assigned a higher g/kg value and is mechanically more likely to cross a low-lean-mass threshold. That the association reverses so cleanly — low intake "protective" — is the signature of a denominator artifact, since it would otherwise require low protein intake to build muscle.

Section 4.6 — the citation error in full

In the literature. [127] cites the Newcastle 85+ study as showing "higher sarcopenia incidence over 3 years among those consuming ≥1 g/kg body weight per day of protein (OR = 2.57, 95% CI = 1.26–5.26)". We retrieved the source (Granic *et al.*, *Clin Nutr* 2020;39:166–173; [128], PMC6961212). **OR 2.57 is the comparison of dietary pattern DP2 ("Traditional British") versus DP3 ("Low Butter") for three-year prevalent sarcopenia**, adjusted Model 3. The figure ≥1 g/kg aBW/day appears in that paper only as the cut-point defining the "good protein intake" stratum. The source concludes the opposite of the citation: the harmful dietary pattern raised sarcopenia risk **even when protein intake was good** (OR 5.45, 1.81–16.39). The estimate was excluded from our analysis. It had been the sole apparent contradiction to an otherwise consistent finding in older adults.

Section 3.2 — general versus clinical populations

General versus clinical populations. General-population cohorts pooled to HR 0.73 (0.68–0.78), k = 5, I^2 = 51%; clinical cohorts to HR 0.77 (0.73–0.82), k = 11, I^2 =

47%. The moderator test is not significant as reported (Q_M = 1.71, p = 0.191), but that result depends on how overlapping cohorts are handled. Four clinical estimates derive from overlapping NHANES cycles. With all four removed the test gives p = 0.049, and with the four combined into one estimate p = 0.039. With one retained at a time, p ranges from 0.013 to 0.211, the clinical subgroup moving to HR 0.77–0.81 while the overall estimate stays at 0.75–0.77 (Table S14). Higher activity is therefore associated with lower mortality in people with diabetes, cancer, stroke, heart failure, liver disease, depression or COPD, as well as in the general population. The data do not establish that the association is of the same magnitude, and a modestly weaker association in clinical cohorts cannot be excluded. The single largest point estimate, in patients discharged after a COPD admission, comes from the least precise cohort in the stratum (HR 0.53, 0.34–0.84). It is also among the estimates most exposed to reverse causation, as set out above for the clinical cohorts added at revision.

Section 2.8 — the screening deviation in full

2. **Screening was single-reviewer, with a duplicate-screened validation sample.** Screening and adjudication of extracted estimates were performed by one author (K.S.). At revision, a **stratified random sample of 888 records** was screened independently on title and abstract by two further authors (V.G.-O. and C.D.). One further record, admitted by the top-up search after the sample had been drawn, was also decided by both, giving **889 records in all**. An inter-rater statistic is reported for that set (Section 2.9, Figure 1). The main corpus outside the sample remains single-reviewer, and that is the limitation a reader should carry forward. The validation sample estimates how reproducible the screening step was; it does not make the whole corpus duplicate-screened. This deviation does **not** apply to the risk-of-bias appraisal. ROBINS-E, RoB 2 and AMSTAR 2 were rated in duplicate throughout, with K.S. resolving disagreements. The one exception is the Newcastle–Ottawa cross-check, scored at revision by a single rater and informing no rating (Section 2.9, Table S7b).

Section 3.2 — the activity pool

Sixteen prospective cohorts compared the most-active or guideline-meeting category with an inactive or below-guideline reference. The pooled association with all-cause mortality was **HR 0.76 (95% CI 0.72–0.79), k = 16, I^2 = 64%, τ^2 = 0.0048, p = 1.1×10^{-28}**, with a 95% prediction interval of 0.65–0.87 (Table 1, Figure 2).

The largest estimate of this curve is the pooled analysis of 661,137 adults. Leisure-time activity **below** the recommended minimum already carried **HR 0.80 (0.78–0.82)** against none, with the optimum at three to five times the recommendation and no further gain beyond [10]. Two qualifications travel with it, and we state them rather than bury them. The same analysis found no lower mortality at a trivial dose, so the

floor is a way in and not a license to do almost nothing. And self-reported leisure-time activity and device-measured MVPA are not the same exposure, and are not interchangeable on a minutes axis (Document S4).

Individual cohorts locate the steep segment of the curve substantially below 150 min/week. They report a detectable association from 10 min/week of device-measured MVPA [15]; an inverse association from about 3,143 steps/day [42]; optimal step cut-points of about 1,824/day above age 75 and 4,128/day below it [43]; and lower mortality at a median of 5.3 daily bouts of vigorous intermittent activity [44].

The consistent shape is L-shaped rather than linear. The largest marginal return occurs at the transition from zero to any activity, and the curve is close to flat well before the volumes most guidelines advertise.

Section 3.4 — odds ratios from primary 2x2 data

Of 688 papers carrying raw event counts, one published a genuine exposure-stratified 2×2 table for a relevant exposure and mortality. The remainder reported deaths per person-year, yielding incidence rate ratios rather than odds ratios, or counts unrelated to the exposure. From [32] (n = 109,407) we computed crude odds ratios against the authors' adjusted hazard ratios (Table S4.1 below).

Crude ORs are uniformly more extreme than the adjusted HRs. With a 4.6% event rate the odds ratio closely approximates the risk ratio, so the gap reflects confounder adjustment rather than non-collapsibility. Reverse causation — illness reducing activity — remains unaddressed in the crude figures, which is why the adjusted estimates, not these, should anchor policy. The crude table is presented to make the underlying counts auditable.

Derived contrasts: any activity versus none, **OR 0.81 (0.76–0.86)**; approximately meeting the WHO guideline (≥150 min/week, Q2–Q4) versus none, **OR 0.74 (0.69–0.79)**.

Table S4.1. Total physical activity and all-cause mortality: crude ORs computed from published cell counts.

TPA group	Deaths	N	Crude OR (95% CI)	Published adjusted HR
—	—:	—:	—	—
No TPA (reference)	2,456	53,240	1.00	1.00
No TPA (reference)	2,456	53,240	1.00	1.00
Q1: 0–75	592	11,973	1.08 (0.98–	1.08 (0.98–

TPA group	Deaths	N	Crude OR (95% CI)	Published adjusted HR
min/week			1.18)	1.19)
Q1: 0–75 min/week	592	11,973	1.08 (0.98–1.18)	1.08 (0.98–1.19)
Q2: 75–360 min/week	530	15,043	0.76 (0.69–0.83)	0.89 (0.81–0.98)
Q2: 75–360 min/week	530	15,043	0.76 (0.69–0.83)	0.89 (0.81–0.98)
Q3: 360–720 min/week	524	14,366	0.78 (0.71–0.86)	0.88 (0.79–0.96)
Q3: 360–720 min/week	524	14,366	0.78 (0.71–0.86)	0.88 (0.79–0.96)
Q4: >720 min/week	469	14,785	0.68 (0.61–0.75)	0.77 (0.69–0.85)
Q4: >720 min/week	469	14,785	0.68 (0.61–0.75)	0.77 (0.69–0.85)

Section 5 — how to read Figure 5

Three things in this figure carry the argument.

The reference value is one number. Panel (a) sets a single red line against eleven population windows, nine of which have a lower bound at or above it. That line is the WHO/FAO/UNU safe level of 0.83 g/kg/day, applied unchanged to a 25-year-old, an 85-year-old, a dialysis patient and a patient losing 15% of body weight on an incretin agonist. That is the disagreement in its entirety (Document S4).

The denominator column is not decoration. Three rows use ideal or adjusted body weight. At a BMI of 35 the choice changes the prescribed intake by roughly 40%, and expressing intake per kilogram of actual weight is what manufactures the spurious harm signals of Section 4.2.

Panel (b) shows a threshold facing a distribution. The WHO aerobic band is 150–300 min/week. The curve has already bent well below 150, which is why we propose a floor at 10 min/week and a gradient to roughly 500. For muscle strengthening WHO states a frequency and no volume at all (Document S4).

The green block is a different endpoint, and it converges on the same number. The three lower bars answer a question about muscle rather than survival. They are drawn apart for that reason. Read on their own terms they are well supported. Protein added to resistance training raises one-repetition-maximum strength by 2.49 kg (0.64–4.33) and fat-free mass by 0.30 kg (0.09–0.52), with a break-point at

1.62 g/kg/day (1.03–2.20) beyond which no further gain was observed [12], [136]. Document S4 gives the supporting syntheses.

Three cautions travel with that block, and the figure states them rather than hiding them. The effects are real but small in absolute terms, and no trial has shown that optimizing them extends life. The athlete bar is a break-point confidence interval carried to its upper limit, not a dose that outperforms 1.6 g/kg/day. The honest reading above roughly 1.6 is *not demonstrated to be harmful*, not *demonstrated to be better*. And the whole block is conditional — without resistance training, protein changes strength by **0.13% (−1.53 to 1.79)** [83]. It is consistent with the International Society of Sports Nutrition position stand of 1.4–2.0 g/kg/day [129], which is reassuring for practice but is a consensus document and not independent confirmation. The bars are a dose; they are not, on their own, an intervention.

The three passages below formed Sections 5.9–5.11 of the submitted manuscript and were moved here at revision to shorten the main text (Section 5.9 now summarizes them). Section numbers inside them refer to the revised manuscript; the former Section 5.12 is now Section 5.10.

Former Section 5.9 — the general adult population, in full — *the average, not the exception*

Sections 5.1–5.8 are addressed to the populations in which the evidence separates. Most people are in none of them, and a review that only stratifies leaves the largest group without an answer. This section states what the same evidence supports for a healthy adult under 65 with normal kidney function, no heart failure and no incretin prescription. **Where a person belongs to one of the strata in Table 6, that row overrides everything below.**

On movement, the number to remember is the first step, not the target. The steepest mortality gradient lies between doing nothing and doing a little. It is largely complete before the guideline threshold (Section 5.1). Two additions carry most of the rest. They are 60–120 min/week of muscle strengthening, with no further reduction in mortality above 120 (Section 5.2), and counting incidental and intermittent activity toward the total (Section 5.3). Adherence, not intensity, is the binding constraint [131] (Document S4).

On protein amount, there is nothing to chase — but there is something to avoid, and it is the low end. For a healthy adult under 65 the defensible statement is a wide adequacy range: 0.8–1.6 g/kg of actual body weight per day, within which no position has been shown to be better than another. The widely quoted 1.6–2.2 g/kg/day is a hypertrophy break-point estimate carried to the upper bound of its confidence interval, not a benefit gradient (Section 5.5, Document S4).

On protein source, the evidence is observational but consistent. Three practical statements follow from Sections 3.5, 3.7, 4.4 and 5.7.

1. **Replace ultra-processed meat products with traditionally prepared ones.** This rests on observational and substitution evidence. Processed meat is more strongly associated than unprocessed red meat for type 2 diabetes (RR 1.41, 1.24–1.60 per 50 g/day versus 1.11, 1.06–1.16) [66]. Substituting 3% of energy from plant protein is also associated with a larger reduction when it replaces processed meat (all-cause mortality HR 0.54, 0.38–0.75) than unprocessed red meat (0.66, 0.55–0.80) [124]. The risk tracks the *processing* — nitrite curing, sodium load, emulsified fat — which is a property of the product and not of the meal it sits in.
2. **Unprocessed red meat is not the target, but it is not unconditional either.** Its association with all-cause mortality is small (HR 1.12, 1.04–1.20) [121] relative to the processed fraction. Three groups should nonetheless treat it differently: established cardiovascular disease, pregnancy, and anyone in whom a red-meat-dominant pattern has replaced a mixed one (Document S4).
3. **What it is eaten with can matter more than the meat.** A pattern combining baked or fried food with white meat was associated with gestational diabetes at aOR 4.40 (1.58–12.22) against a prudent pattern [122]. That is larger than any single protein-source estimate here. The operational advice is about the plate, not the portion: unprocessed meat, not deep-fried, eaten with vegetables, legumes and whole grains.

"Plant-based" is not self-justifying, and the split is measurable. A healthful plant-based index was inversely associated with type 2 diabetes (HR 0.66, 0.61–0.72). An unhealthful one — refined grains, potatoes, sweetened beverages, desserts — was positively associated (1.16, 1.08–1.25) in the same cohorts [69]. Advice that does not distinguish the two can move risk the wrong way.

One thing worth doing before 65 that is usually left until after it. Strength training in middle age raises the muscle a person carries into old age, which is the substrate every recommendation in Section 5.5 operates on. Unlike the protein question, it has a demonstrated effect on function at every age tested.

Former Section 5.10 — red flags: how to recognize a recommendation this evidence does not support

The failure modes below are not hypothetical; each was observed in the guidance, trials or reviews assembled for this synthesis, and each has a specific anchor in the evidence. They are offered as a checklist for anyone drafting, appraising or implementing a recommendation on activity or protein.

Sixteen failure modes are set out one by one in this document, each with the anchor in this review that identifies it. In summary, a protein recommendation is unsafe under any of eight conditions. It gives a number with no denominator; applies one floor across populations; issues a target without a loading prescription, or,

conversely, withholds the target because loading is not feasible; restricts intake in the old or the wasting; carries chronic-disease reasoning into acute illness; presents athletic ranges as a benefit gradient; or rests a longevity claim on biomarkers. An activity recommendation is unsafe if it frames 150 min/week as a pass/fail threshold, treats strength work as optional, or publishes a strength requirement with no ceiling. And the evidence being cited is unsafe under five further conditions. Sarcopenia was screened on a single body-composition measure; "plant-based" is used without a healthful/unhealthful split; "eat less red meat" is offered where a processing-targeted measure exists; a cohort protein estimate is quoted without its denominator problem; or **an effect on muscle or function is presented as an effect on survival**, the one gap this review could not close (Section 4.5). Table 8 applies that last test to our own recommendations, one row at a time.

Former Section 5.11 — the real-life version: three numbers a person can act on

Everything above is written for the people who draft guidance. This section is written for the consultation, and it reduces the review to the smallest set of numbers that survives it. **The numbers are for a healthy adult; any row of Table 6 or Table 7 overrides them.**

1. Steps, counted outside work: aim past ~3,000 a day, and keep going. An inverse association is detectable from approximately **3,143 steps/day**, with **HR 0.91 (0.87–0.95) per additional 1,000 steps** [42]. Above age 75 the optimum falls to about 1,824, and below 75 it sits near 4,128 [43]. The 10,000-step target is not supported as a threshold by this evidence. Two qualifications apply. The gradient does not stop at the threshold, so this is a floor and not a target. And **whether the same association holds for steps accumulated at work was not addressed by this corpus and should not be assumed** [10] (Document S4).

2. Minutes per week: ten to start, 300–500 to finish, and 60–120 of them against resistance. A statistically significant lower risk was detectable from **10 minutes per week** of accelerometer-measured MVPA, with the curve flattening beyond roughly **500 min/week** [15]; the self-reported optimum sits at three to five times the 150-minute recommendation, i.e. **450–750 min/week** [10] — the two are not in conflict, because self-reported and device-measured activity are not the same exposure. Separately from all of that, **60–120 minutes per week of muscle-strengthening work**, with **HR 0.87 (0.81–0.95) at 90–119 min/week** and **no further reduction in mortality above 120** [45]. Brief incidental bursts were also associated with lower mortality. A median of **5.3 bouts/day** of vigorous intermittent activity carried **HR 0.56 (0.39–0.82)** [44], and such bursts require no gym, no equipment and no change of clothes.

3. Protein: more is better than less only until adequacy. Below the floor, higher intake is associated with better outcomes, and the effect sizes are large. In older adults, intake under 0.8 g/kg/day against ≥1.2 carried sarcopenia **OR 1.87 (1.46–2.38)** [53]. In disease-related malnutrition, falling below 70% of requirement carried **OR 4.27 (1.30–14.03)** [62]. Above adequacy, the gradient disappears. Total protein intake is null for all-cause mortality in three independent syntheses and in our own five-cohort pool, and the best-measured cohort in that stratum reports higher rather than lower mortality at the top of the distribution (Section 3.5). In three defined groups it reverses (Table 7). The operational form is therefore:

- **Under 65 and healthy:** anywhere in **0.8–1.6 g/kg of actual body weight per day**. There is nothing to chase inside that range, and the athletic 1.6–2.2 g/kg/day is the upper reach of a break-point confidence interval, not a benefit gradient.
- **65 and over: 1.2–1.6 g/kg/day**, delivered as **25–30 g at each of two or three meals** rather than concentrated at dinner; reaching 20 g at two or more meals was associated with higher handgrip strength [135]. It should be **paired with the resistance work of point 2**, because that is where the effect on strength and function lies.
- **Never below 0.8 g/kg/day** without a specific clinical indication. Protein should **not** be raised in the acute phase of a serious illness, where doing so did not help and, with acute kidney injury, was associated with higher mortality.

And the one thing not to do with these numbers. They describe where the observed association begins, not a score to maximize. The activity curve is L-shaped and the protein curve is a window. A person already meeting the floors gains far less from doubling them than an inactive person gains from starting.

The passages below were moved from the main text at revision to shorten it; the main text retains a summary and a pointer at each location.

Section 3.12 — the gut microbiome, in full

Section 3.5 found that protein *source* carries a signal that protein *amount* does not. The gut is the obvious place to look for why, and it is the one part of this review where the mechanism is more convincing than the evidence.

The dietary signal is reproducible. Two independent NHANES analyses used the Dietary Index for Gut Microbiota, a score built from foods known to shift the microbiome rather than from sequencing. Both return the same number for the highest versus lowest tertile: odds ratio **0.70 (0.53–0.91)** for frailty in 4,578 older adults [106], and **0.70 (0.57–0.87)** for sarcopenia in 2,169 adults with chronic kidney disease [107]. The foods scoring well on that index are, almost entirely, the plant-protein and fiber sources Section 3.5 found to be associated with lower risk.

The causal-inference evidence is directional but small. Mendelian randomization implicates specific taxa in low grip strength (*Alcaligenaceae* OR **1.17, 1.05–1.30**) [108] and in sarcopenia (*Porphyromonadaceae* OR **1.05, 1.005–1.09**). The microbiota-derived metabolite creatine is associated with lower odds (OR **0.95, 0.91–0.995**) [109].

The sequencing evidence does not yet support a signature. A systematic review of nineteen studies found taxa associated with sarcopenia in most of them, but with directions "heterogeneous and also conflicting" [110]. A meta-analysis of frail versus non-frail older adults found no difference in species richness (SMD **−0.15, −0.39 to 0.10**) [111].

The summary is uncomfortable but clean. **The diet that feeds the microbiome predicts muscle outcomes; the microbiome itself does not yet.** Section 4.5 sets out the mechanism this would run through. It is worth testing, not legislating on, and we have deliberately kept it out of Section 5.

Section 3.13 — sarcopenia, in full (as submitted, before condensation)

Both exposures in this review, activity and protein, act on the same tissue. The estimates that quantify what is at stake therefore belong in the results rather than only in the interpretation.

The chain this review depends on, link by link, with the endpoint and the design at each step. It is set out here because the strength of the argument is the strength of its weakest link. The link that fails is the last one:

- **Protein → muscle.** *Randomized.* Added protein raises muscle mass by a small margin without training (SMD 0.116, 0.032–0.200) [93]. It raises strength only alongside resistance training (+2.01% versus +0.13%) [83], [112]. **Established, and conditional.**
- **Loading → muscle.** *Randomized.* Resistance training raises handgrip strength in sarcopenia by 2.69 kg (1.78–3.61) across 42 trials [92], and protein with training outperforms either alone [113]. **Established.**
- **Muscle → sarcopenia.** *Definitional and measured.* Which phenotype is measured determines the estimate. Low mass alone, mass-plus-function, and sarcopenic obesity are not the same exposure, and the last carries the most risk. **Established, and definition-dependent.**
- **Sarcopenia → mortality.** *Observational.* The association is well away from the null in every estimate assembled here, and larger where organ disease and muscle loss coincide. It remains observational, and carries the reverse-causation problem that early illness lowers both intake and muscle. **Consistent, not causal.**

- **Protein-plus-loading → mortality.** *Untested.* No randomized estimate in this corpus carries a mortality endpoint, in any population (Section 4.5). **This is where the chain breaks, and no recommendation in Section 5 is written as though it does not.**

The penalty carried by the sarcopenic state depends on how the state is defined. Low muscle mass alone carries all-cause mortality RR 1.36 (1.28–1.44) across 49 studies. Requiring function as well gives a much larger figure, OR 3.64 (2.94–4.51) in adults 60 and over. In type 2 diabetes, sarcopenia carried HR 1.70 (1.21–2.40) across 21 cohorts [114], [115], [116]. The three are not directly comparable, because an odds ratio exceeds the corresponding risk ratio when the outcome is common. Document S4 sets out why no single number should be quoted as the hazard of muscle loss.

Two things survive that caution. Every estimate is well away from the null and the direction is consistent. And **the phenotype definition determines what a screening program will find.** An approach that measures mass alone is working with the weakest of the three definitions, which is the practical form of the point made below about osteosarcopenia and sarcopenic obesity. No single number should be quoted as *the* hazard of muscle loss.

The penalty is larger where organ disease and muscle loss coincide, and it is the *combined* phenotype that carries it. In older CKD outpatients it was osteosarcopenia rather than sarcopenia or low bone density alone. In type 2 diabetes with preserved renal function it was sarcopenic obesity rather than low muscle mass alone, with low handgrip strength the strongest single component (HR 2.44, 1.46–4.08) [117], [118]. Single-measure screening misses the group the recommendation is for [115], [118], [114], [117] [93] (Document S4).

What the exposures do to that outcome. Protein supplementation raises muscle mass in community-dwelling older adults, and resistance training raises handgrip strength in sarcopenia by 2.69 kg (1.78–3.61) across 42 trials [92]. The two together outperform either alone on mass and strength [113], the same conditional structure as Section 3.9 (Document S4).

Three qualifications belong with these numbers. The mortality and CKD estimates are observational, and carry the reverse-causation problem set out in Section 4.2. Muscle mass in a cohort is partly an index of the illness that kills the participant. The isolated-nutrient version of the intervention is the weakest. Leucine alone does not improve muscle mass or strength in older adults; benefit appears only in combination with resistance training or microbiota-targeted co-intervention [119]. And none of these estimates has an all-cause mortality endpoint on the intervention side; what they establish is that the *outcome* is consequential, not that moving it extends life. Section 4.5 states what would be required to close that gap.

Section 3.5 — bounding the unrated Women's Health Initiative cohort

One plant-protein cohort, the Women's Health Initiative, was admitted by the top-up search at revision, after the duplicate appraisal was complete, and has not been rated. Its possible ratings were therefore bounded. The restricted result is the same whichever way it would be rated. If it were rated High, the non-High plant-protein set is the SUN cohort alone — **k = 1, HR 0.65 (0.45–0.93)**, not poolable. If it were rated Some concerns or Low, the set is SUN plus the Women's Health Initiative: **k = 2, HR 0.81 (0.59–1.11), I^2 = 69%**. That is below our three-study minimum and, in any case, no longer excludes the null. There is therefore no assumption about this cohort under which the restricted plant-protein stratum yields a poolable or a significant estimate. No conclusion in this review turns on the missing rating. No rating was assigned outside the duplicate-appraisal procedure of Section 2.9; the appraisal is logged as an open item in Document S1.

Section 4.4 — the contrary reading and cooking method

A contrary reading of the same territory should be acknowledged rather than argued away. The association between unprocessed red meat and mortality is small, sensitive to adjustment, and confounded by everything that travels with a meat-heavy diet; some read it as null. Our recommendation is deliberately narrower for that reason — it targets the processed fraction, which is a property of the product rather than of the meal.

Our corpus contains almost no direct evidence on cooking method: a systematic search for cooking-method, food-matrix, heterocyclic-amine and co-consumption effects on protein-related outcomes returned two usable estimates. Section 5.8 identifies this as a research priority.

Section 4.5 — the mortality query and trial design

On mortality, the interaction remains untested. Our dedicated query on combined resistance training and protein returned 128 records, and **not one yielded an estimate meeting a mortality stratum's contrast definition**. [121] frames meat intake and physical activity as jointly modifiable, reporting in the same analysis red meat HR 1.12 (1.04–1.20) against daily steps HR 0.58 (0.45–0.74) and muscle-strengthening activity HR 0.91 (0.84–0.98). This is descriptive, not an interaction test.

Section 4.5 — what a survival trial would require

Would the combination extend life, and what would it take to show it? No trial has tested it. What would settle the question is a long, hard-endpoint trial, sited where the outcome penalty is concentrated and powered on disability-free survival

rather than on a body-composition surrogate. Document S4 sets out the design parameters, and why the existing trial portfolio cannot answer it.

Section 5.2 — falls and adherence (short form; full form above)

Two further findings bear on how this recommendation should be written. An umbrella review of 41 systematic reviews found high-certainty evidence that balance and multicomponent exercise reduce fall rates by 23–34% [113]. Adherence to the recommendation is low in some groups. Only 23.7% of US adults over 45 met it, with the lowest odds among those with less than a high-school education (OR 0.32) and a BMI of 30 or more (0.47) [131]. (Document S4).

Section 3.10 — the two RoB 2 ratings

Of the two randomized trials appraised with RoB 2, Generation 100 carries Some concerns: control participants were free to exercise and many did, so the trial estimates an effect of assignment under substantial contamination [77]. The six-year very-low-protein trial carries High risk of bias, because adherence to that diet over that horizon is neither sustainable nor verifiable [89].

Section 3.10 — the five Critically low AMSTAR 2 reviews

Five reviews are rated Critically low, and they do not all fail in the same way. **Two are critically flawed in the pooling itself and support nothing.** One pools standardized mean differences across incommensurable proteinuria assays [90]. The other describes handgrip and physical-performance results with I^2 of 94% and confidence intervals crossing the null as “a tendency for improvement” [84].

Three were recalculated at revision, when an internal audit found that their overall ratings had been assigned as global impressions rather than by the rule Table S9 prints. Applying that rule to the critical-domain judgments already recorded moves three reviews to Critically low. The individual-participant meta-analysis in heart failure [80] has two critical flaws: exclusions not listed individually, and publication bias not formally assessed. The network meta-analysis of cardiac rehabilitation modes [81] and the meta-analysis of protein restriction and kidney disease progression [87] have three each: no registered protocol, exclusions not justified individually, and risk of bias not carried into the interpretation. No domain judgment was changed.

The distinction matters for how the three are handled. Their flaws are in protocol registration, in the justification of exclusions and in the treatment of publication bias: failures of conduct and reporting rather than of the arithmetic. The estimates are therefore reported with the rating stated at each point of use, and the certainty of the bodies of evidence to which they contribute is rated down instead of the estimates being discarded (Section 2.9). Two certainty ratings change as a result: structured exercise against all-cause mortality, and protein restriction in chronic

kidney disease against mortality, both from Moderate to Low (Table 5, Table S10). It is worth saying plainly which way this cuts. The two randomized estimates furthest from the null in the exercise layer, RR 0.76 and HR 0.83, are the two that come from the least reliable reviews. The recalculation therefore weakens the randomized case for exercise rather than strengthening it.
